# Ligand-Controlled Chemodivergent Bismuth Catalysis

**DOI:** 10.1021/jacs.5c11854

**Published:** 2025-11-07

**Authors:** Lucas Mele, Philipp D. Engel, Jamie A. Cadge, Vytautas Peciukenas, Hoonchul Choi, Matthew S. Sigman, Josep Cornella

**Affiliations:** † 28314 Max-Planck-Institut für Kohlenforschung, Kaiser-Wilhelm-Platz 1, Mülheim an der Ruhr 45470, Germany; ‡ Department of Chemistry, 7060 University of Utah, 315 1400 E, Salt Lake City, Utah 84112, United States; § Catalysis Research Laboratory (CaRLa), Im Neuenheimer Feld 271, Heidelberg 69120, Germany; ∥ BASF SE, Carl-Bosch-Str. 38, Ludwigshafen 67056, Germany

## Abstract

Herein, we report a ligand-controlled chemodivergent bismuth-catalyzed coupling between arylboronic acids and *N*-fluorosulfonimide derivatives that enables the selective formation of either C­(sp^2^)–N or C­(sp^2^)–O bonds. Selectivity is achieved by the modulation of the electronic and steric properties of a common ligand framework for bismuth, thus establishing an unusual ligand-controlled chemodivergent platform in main group catalysis. Specifically, the use of an electron-enrich sulfone ligand led to the major formation of sulfonimide with selectivities ranging from 2:1 to more than 20:1. Conversely, a bismuth catalyst supported by an electron-deficient sulfoximine predominantly promoted the sulfonimidate product with ratios ranging between 5:1 and 15:1. To understand the underlying principles that control the selectivity, a comprehensive mechanistic investigation was conducted by combining experimental stoichiometric studies, DFT calculations, and statistical modeling. These studies support a catalytic high-valent bismuth redox cycle, where Bi­(V) intermediates dictate product selectivity through either a three- or five-membered reductive elimination–ligand coupling event. By means of statistical modeling, we identified that the charge of the coordinating heteroatom through hypervalency, together with a steric parameter around the bismuth, is the key parameter responsible for the stabilization of the relevant transition states that lead to control over the reductive elimination process.

## Graphical abstract

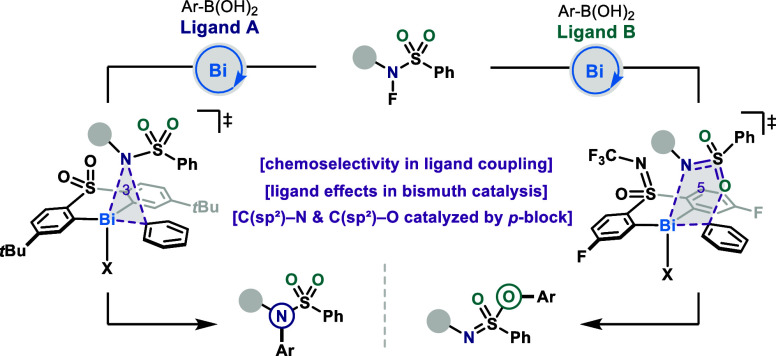

## Introduction

The realization that the electronic and steric properties of the ligands attached to transition metal centers dictate the outcome of a given catalytic transformation revolutionized transition metal catalysis.^1^ Consequently, it is no surprise that the pursuit of ligand design opportunities has been the cornerstone of method development: from shunting undesired pathways to stabilizing the metal center. A pinnacle of such interplay is highlighted in ligand-controlled chemodivergent reactions, in which two distinct products can be formed originating from the same material under similar conditions (metal catalyst, additive, solvent, temperature, time, etc.) except for the ligand.^2^ Therefore, from a common platform, selective pivotal bonds such as C­(sp^2^)–N, C­(sp^2^)–O, or C­(sp^2^)–C­(sp^2^), among others, can be forged (Figure 1A).

**Figure 1:**
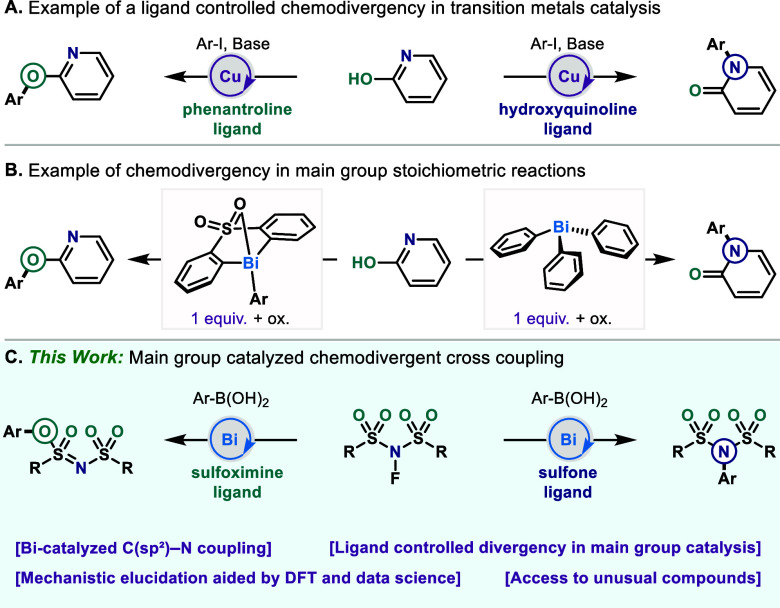
(A) Cu-catalyzed ligand-controlled chemodivergent coupling of 2-hydroxypyridine with aryl iodide.^5^ (B) Stoichiometric oxidative arylbismuth chemodivergent coupling with 2-hydroxypyridine. (C) Bismuth-catalyzed ligand-controlled chemodivergent coupling of NFSI with arylboronic acids.

Although C­(sp^2^) functionalization is traditionally the reserved domain of transition metals armed with their supporting ligands, the past decade has seen an uptick of main group chemistry and catalysis, thus achieving similar transformations using electrons in frontier p-orbitals.^3^ In the absence of d-orbitals participating in bonding, main group catalysts often rely on distorted geometries enforced by their ligand frameworks to dictate the reactivity. Due to these restrictions, fine-tuning of the ligand structure to achieve selective and efficient reactions in main group chemistry has been challenging. Although some chemodivergent transformations using main group reagents have been reported, this has mainly been dependent on the nature of the additives or substrate type;^4^ only a few stoichiometric examples of chemodivergence have been controlled by ligand structure.

Seminal studies by Barton, Finet, and Motherwell on organobismuth­(V) compounds demonstrated that phenol arylation yields either C­(sp^2^)–O or C­(sp^2^)–C­(sp^2^) products, depending on the acidity of the reaction medium or the nature of the substituents bound to the Bi center.^6^ Using similar high-valent bismuth reagents derived from homoleptic triarylbismuthine, Mukaiyama developed a selective *N*-arylation of 2-hydroxypyridine.^7^ More recently, the Ball group showed that the selectivity on the same substrate could be reversed in favor of the *O*-arylation product by employing a stoichiometric amount of a diarylsulfone-tethered bismuth reagent (Figure 1B).^8^ The authors suggested that intramolecular hydrogen bonding between the sulfone moiety and a C­(sp^2^)–H bond of the nucleophile could be responsible for the ambivalent nucleophile orientation, leading to the observed chemoselectivity.

Hypervalent halogen reagents were also employed in chemodivergent transformations, such as in the cycloisomerization of *o*-alkenylbenzamide, where a combined effect of solvent, ligand, and additive could influence the relative nucleophilicity of the ambident partner.^9^ Unfortunately, the reaction outcome could not be tuned by ligand replacement alone.

Considering these precedents, among the p-block candidates to develop a catalytic ligand-controlled chemodivergent transformation, bismuth is an attractive choice as a result of its ability to control and tune elementary organometallic steps. Specifically, transmetalation of Bi­(III) intermediates with organoboron- and organosilicon-nucleophiles has enabled catalytic processes building C­(sp^2^)–S bonds in a redox-neutral strategy,^10^ as well as C­(sp^2^)–F^11^ and C­(sp^2^)–O^12^ bonds by cycling between various oxidation states.^13^ High-valent bismuth intermediates have also been suggested to undergo pseudo-reductive elimination processes via a variety of modes: e.g., three-membered ring, five-membered ring, or outer-sphere mechanisms.^14^ Interplay between these mechanisms provides a possible framework for developing reactions with ligand-controlled chemodivergence.

Building on our continued interest in high-valent bismuth catalysis, we looked to expand the range of transformations achievable with bismuth catalysts derived from bis-aryl sulfone and sulfoximine ligands to an unprecedented catalytic C­(sp^2^)–N bond formation.^15^ Capitalizing on the ability of bismuth to couple weak nucleophiles such as triflates (^−^OTf) and nonaflates (^−^ONf),^12^ as well as the feasibility of accessing Bi­(V) intermediates with electrophilic fluorine sources, we investigated the coupling between Bi­(III)–Ar derivatives and *N*-fluorobenzenesulfonimide (NFSI), which could be rendered catalytic when using arylboronic acids as the aryl source. In contrast to our prior reports on high-valent Bi catalysis, the oxidant and the nucleophilic partner are combined into a single reagent.^16^ Moreover, selectivity favoring C­(sp^2^)–N or C­(sp^2^)–O products can be achieved by a change of the ligand substitution; an example of a unique ligand-controlled chemodivergence in main group catalysis (Figure 1C). Owing to the variety of bismuth catalysts amenable to this reaction, a distinct mechanistic rationale is postulated, which differs from prior work in similar transformations based on high-valent bismuth catalysis.^17^ Finally, we sought to rationalize the ligand-controlled selective outcome of this transformation through a combination of computational studies and statistical modeling. Key molecular descriptors were identified for these main group catalysts, providing insights into crucial selectivity-determining reductive elimination. This information serves as a framework for the design of new transformations based on heavy main group catalysts.

## Results and Discussion

### Stoichiometric Coupling

We initiated our investigations by treating NFSI **1a** in CDCl_3_ at 90 °C with a stoichiometric amount of the selected Bi complex **Bi-1·4-*t*BuPh**, which had previously displayed activity for various reactions.^8,10,12,17^ Gratifyingly, an 88% yield of a mixture of two compounds with a 3:1 ratio in favor of C­(sp^2^)–N coupled product **2a** was observed (Figure 2). In this reaction, in addition to its oxidizing function, NFSI also served as a pro-ambident nucleophile, which could successfully couple with the untethered aryl moiety borne by the bismuth center. This C­(sp^2^)–N coupling giving **2a** as the major product can also be accessed via Pd-catalyzed aryl C–H imidation, as described by Ritter.^18^ However, no direct cross-coupling using a transition metal has been reported to synthesize this moiety. The structure of the second product was confirmed by single crystal X-ray diffraction analysis (SC-XRD) as sulfonimidate **3a**.^19^ Reports of this class of compounds in the literature are scarce. Indeed, although a SuFEx approach has been developed to synthesize related sulfonimidates,^20^ aryl derivatives were only obtained from complex phenoxenium ions.^21^ It is noteworthy that replacing complex **Bi-1·4-*t*BuPh** with simple triphenylbismuth did not afford any coupling product. Moreover, similar chemodivergence can be observed in the reaction of a vinylbromonium reagent with potassium bis­(triflyl)­imide,^22^ in the decomposition of aryl diazonium compounds,^23^ or in the reaction of methylenecyclopropane with NFSI.^24^

**Figure 2:**
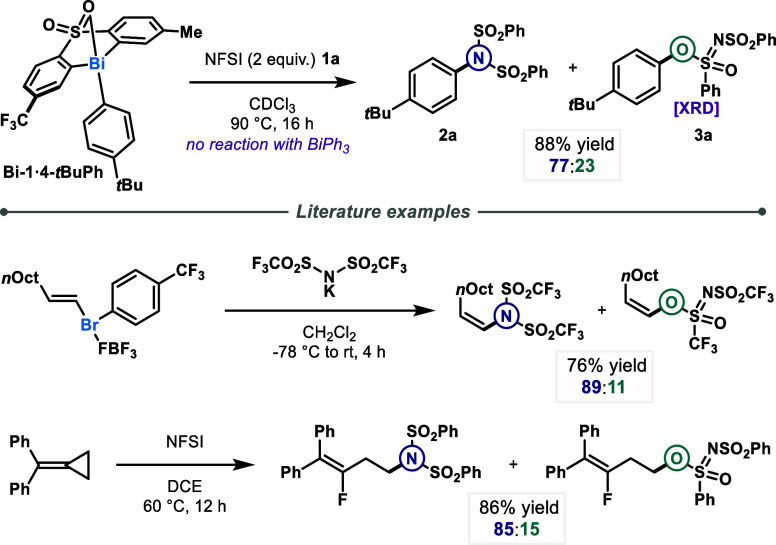
Stoichiometric reactivity of the Bi–Ar complex **Bi-1·4-*t*BuPh** when exposed to NFSI **1a** and comparison with literature examples. Yield was determined by quantitative ^1^H NMR using dibromomethane as an internal standard. Selectivity was determined by HPLC analysis on the crude reaction mixture (see HPLC procedure details in Supporting Information).

### Optimization of Chemodivergent Catalysis

Translating from stoichiometric conditions to catalysis proceeded smoothly, as the use of arylboronic acid **4** in combination with a catalytic amount of sulfone-supported Bi­(III) salt in CDCl_3_ afforded the desired product mixture (see Supporting Information for detailed optimization tables). Further optimization was then conducted to increase the yield as well as the selectivity outcome (see the optimization details in Supporting Information). We evaluated the addition of 20 mol % of 4,4′-bis­(trifluoromethyl)­stilbene (*p*CF_3_-stb), which proved beneficial to the reaction, increasing yield by 20%. In the presence of this additive, enhanced stability of the catalyst under heating was observed (see details in Supporting Information). This can be ascribed to a possible olefin interaction with the Bi center, as previously described.^25^ Control experiments revealed the requirement for an oxidant, as replacing NFSI with its N–H analogue (NSIH) did not lead to any observed product. The sulfone framework is also essential, since other reported ligand frameworks or simple BiCl_3_ were not effective.

The highest selectivity toward the C­(sp^2^)–N product **2a** was obtained using CDCl_3_ as the solvent.^26^ We then proceeded to evaluate a series of catalysts by changing substitutions on the sulfone backbone as well as on the counterion. The use of more electron-donating groups led to improved selectivity toward the C­(sp^2^)–N product **2a**. **Bi-2** bearing a 3-*t*Bu-substituted backbone and Cl as a counterion, proved to be the most selective catalyst (77:23 favoring the sulfonimide product), while maintaining an 84% yield when the reaction was performed at 100 °C (Figure 3, Conditions A).

**Figure 3:**
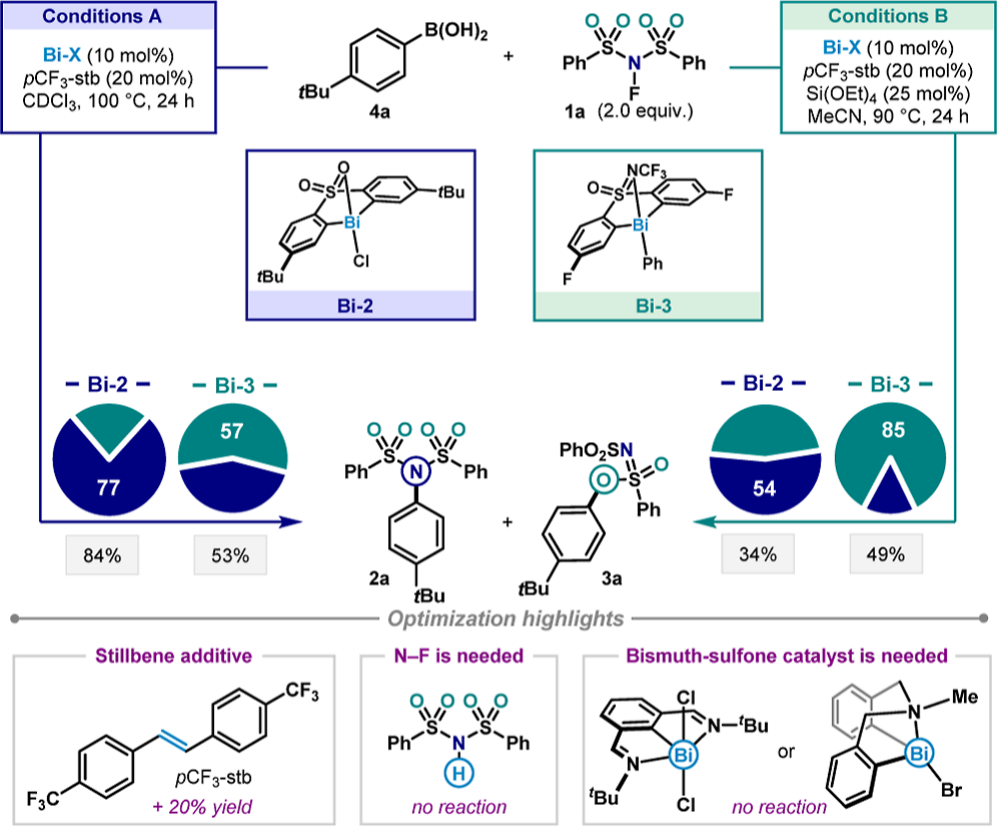
Optimization results of Bi-catalyzed ligand-controlled chemodivergent coupling. Reaction performed on 0.075 mmol of **4a**. Selectivities were determined by HPLC analysis on the crude reaction mixture (see HPLC procedure details in Supporting Information). Pie chart legend: blue = fraction of C­(sp^2^)–N product **2a**; green = fraction of C­(sp^2^)–O product **3a**; and gray box = overall yield (**2a** + **3a**).

Evaluation of the ligand substituents revealed that the presence of electron-withdrawing groups in the sulfone-ligand scaffold leads to the preferential formation of **3a**. Replacing the sulfone with a sulfoximine moiety also increased the selectivity for **3a**. Previous studies have shown that this functional group can play a significant role in stabilizing high-valent bismuth intermediates via intramolecular electronic donation.^17,27^ Catalyst **Bi-3**, which combines both a sulfoximine and electron-withdrawing groups, emerged as the catalyst of choice to favor C­(sp^2^)–O product **3a**. Using the sulfoximine **Bi-3** as a catalyst in Condition A led to the formation of the C­(sp^2^)–O coupling as the major product **3a** (57%) with a 53% yield, thus reversing the selectivity preference by simply changing the ligand.

We next targeted a means to improve the yield of sulfonimidate **3a**, which revealed MeCN as the most suitable solvent. Surprisingly, the use of analogous solvents such as nitromethane, pivalonitrile, or benzonitrile did not show a similar effect. Moreover, the use of MeCN as a solvent in the literature for related reactions did not impact the selectivity outcome.^22–24^ To further enhance the reaction yields, we hypothesized that the formation of fluoride anions may affect the catalyst capabilities, thus reducing efficiency. Therefore, we explored the use of tetraethylorthosilicate as a fluoride scavenger additive, which was found to increase the reaction yield (see additive screening in Supporting Information).^28^ A final set of reaction conditions (Conditions B) using 10 mol % of the sulfoximine **Bi-3** gave 49% yield and selectivity up to nearly 6:1, favoring the C­(sp^2^)–O **3a** product over the C­(sp^2^)–N **2a**. Importantly, the selectivity could also be reversed in Conditions B by solely changing the catalyst to **Bi-2** (54:46 in favor of sulfonamide **2a**), validating the ligand-controlled chemodivergence in this catalytic transformation.

### Scope Evaluation

Having in hand two sets of conditions, we investigated their application to other substrates. Conditions A favor C–N coupling and sulfonimide **2** formation (**Bi-2** as catalyst in CDCl_3_), while Conditions B favor C–O and sulfonimidate **3** formation (catalyzed by **Bi-3** in MeCN). Gratifyingly, the identified conditions proved to be general for other reaction partners, including diversely substituted arylboronic acids **4** and electronically modified NFSIs **1**, thereby ruling out substrate-controlled chemodivergence (Figure 4). More precisely, electron-neutral and electron-rich aryl boronic acids were tolerated (**4a**–**4e**), with the C­(sp^2^)–O product (**3a**–**3e**) being slightly favored as electron density increased. For example, when using Conditions B with phenylboronic acid **4b**, the sulfonimidate product **3b** could be obtained with an 84:16 selectivity, as the more electron-rich 4-MeO derivative **4e** resulted in a 96:4 ratio. The lower yield (45%) observed for the 4-MeO derivative (**4e**) can be rationalized via the direct reactivity of this substrate upon exposure of the electron-rich arene to NFSI.^29^ Electron-poor aryl boronic acids (**4f**–**4h**) also performed well in catalysis under Conditions A, with sulfonimide **2** selectivity increasing in parallel with electron-withdrawing group strength. This effect was most pronounced with a *para*-trifluoromethyl substituent in moiety **4h**, where complete C–N selectivity was observed and virtually no C–O sulfonimidate product **3h** was detected. Due to the lack of product formation, ligand control chemodivergence could not be achieved under Conditions B in the case of electron-poor arylboronic acids. Such peculiar reactivity has also been observed in other bismuth-promoted reactions when applied to electron-deficient aryl sources (vide infra).^6c,17^ Finally, two modified oxidants, **1b** and **1c**, bearing electron-withdrawing groups were prepared and satisfactorily tested in catalysis, affording the desired products in both conditions with a slight decrease in the sulfonimide **2** ratio compared to the standard NFSI (using Conditions A, 65:35 for **2ab** compared to 77:23 for **2a**).

**Figure 4:**
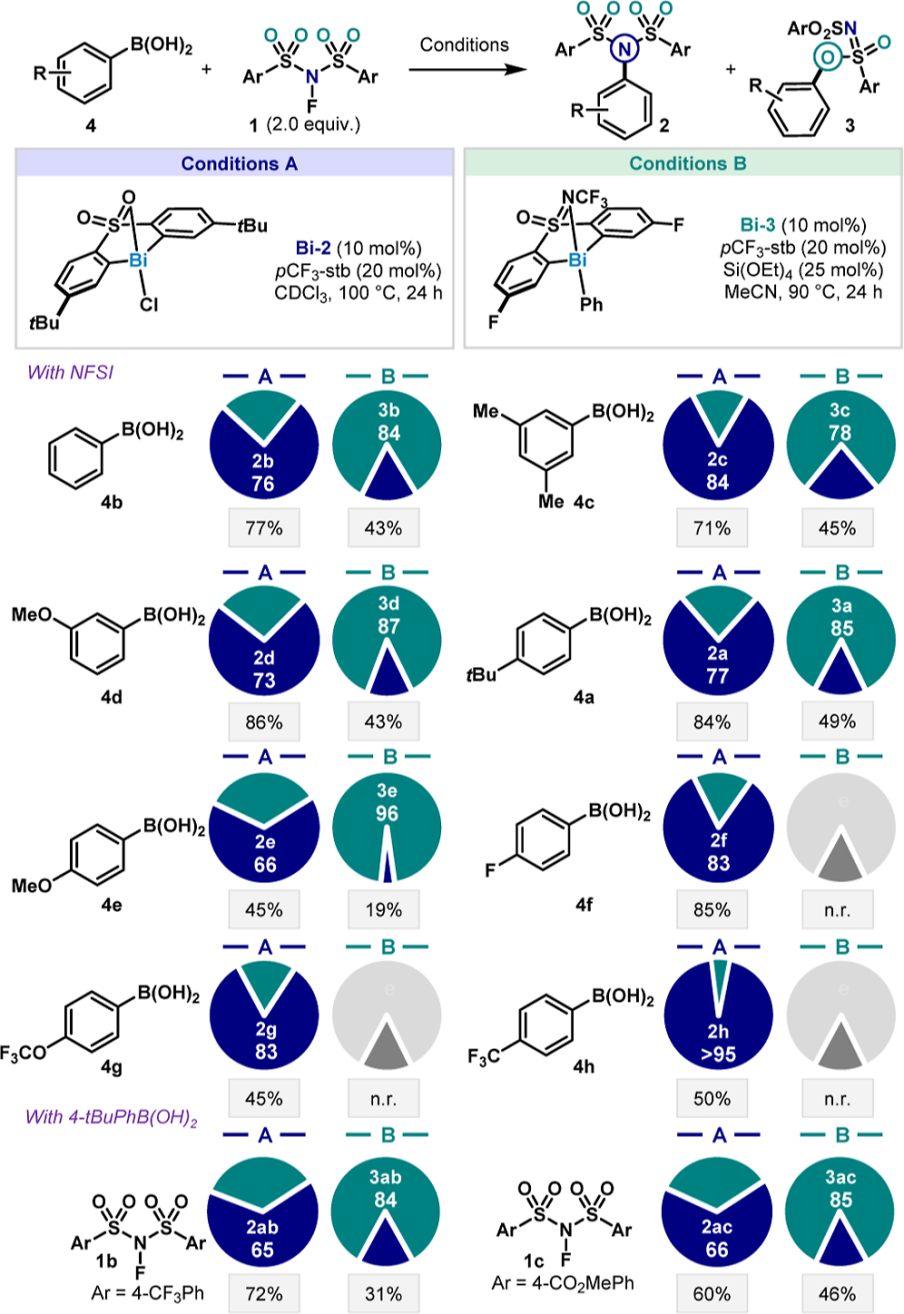
Scope of the ligand-controlled chemodivergent coupling with Bi catalysts. Reaction performed on 0.075 mmol of **4a**. Selectivities were determined by HPLC analysis on the crude reaction mixture (see HPLC procedure details in Supporting Information). Pie chart legend: blue = fraction of C­(sp^2^)–N product **2**; green = fraction of C­(sp^2^)–O product **3**; and gray box = overall yield.

### Mechanistic Investigations

To better understand how this coupling proceeds with a broad range of diarylsulfone bismuth catalysts as well as the factors governing product selectivity, we investigated the mechanism. We began by monitoring the reaction using ^1^H and ^19^F NMR at 90 °C with **Bi-1·OTFA** as catalyst and CDCl_3_ as solvent (see Supporting Information). It was clear that the resting state of the catalyst was a Ar_2_Bi­(III)–X, where Ar_2_ corresponds to the bis-arylsulfone backbone and X evolves from any initial counterion to NSI. To validate this assignment, **Bi-1·NSI** bearing the NSI counterion was independently synthesized by treating Bi–Ph with NSIH (see Supporting Information); indeed, the NMR chemical shifts correspond to the signals observed during monitoring. Similar observations were made when using Ar_2_Bi­(III)–Cl, **Bi-1·Cl** as the precatalyst. Additionally, **Bi-1·NSI** was also able to catalyze the reaction under both conditions. It is important to mention that as the reaction advances, slow catalyst degradation was observed. This is exacerbated when MeCN is used as the solvent, leading to the lower observed yields (Figure 4). Product stability was validated by heating the isolated products **2a** and **3a** separately under various sets of conditions (Figure 5A).^30^ In every case, no interconversion or degradation was observed, thus validating that the observed ratio did not result from a subsequent reaction of the products.

**Figure 5:**
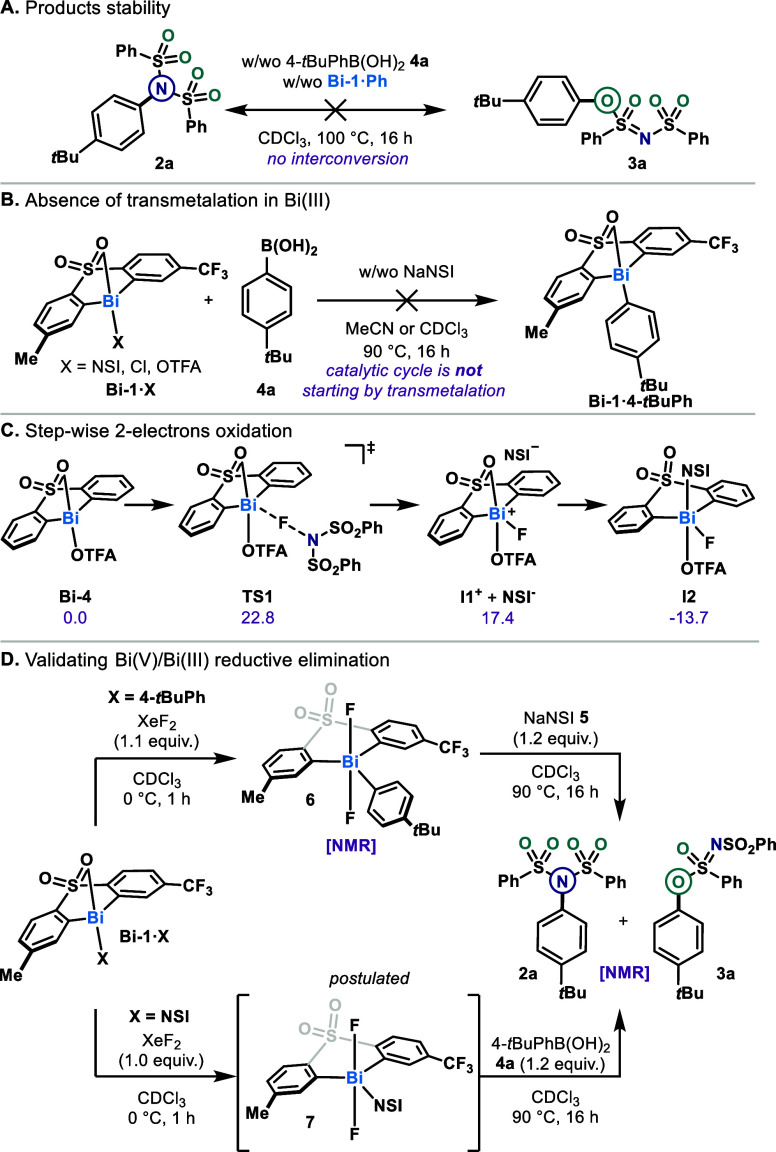
Mechanistic investigations. (A) Product stability under the reaction conditions. (B) Discarding the transmetalation pathway of Ar_2_Bi­(III)–X. (C) Computational validation of oxidative addition. (D) Stoichiometric reductive elimination from high-valent bismuth species.

In previously reported Bi­(III)/Bi­(V) catalysis, transmetalation has been postulated as the first step of the catalytic cycle.^11,12^ This was validated by the facile stoichiometric transmetalation between Bi­(III) precatalysts and the boronic acid derivative. In this case, however, when various active catalysts (**Bi-1·X**) were combined in this transformation with 4-*tert*-butylphenyl boronic acid **4a** in CDCl_3_ or MeCN at 90 °C, no transmetalation was observed (Figure 5B). Addition of sodium bis­(phenylsulfonyl)­amide (NaNSI) **5** did not change the outcome. The absence of reactivity between various Bi­(III) precatalysts **Bi-1·X** and the boronic acid **4a** suggested that transmetalation might not be the first step of the catalytic cycle. However, the pioneering work from Matano has defined a base-free transmetalation from Bi­(V)–F even at room temperature.^31^ With these precedents in mind, we investigated if oxidation of the precatalysts could precede the transmetalation step. Unfortunately, cyclic voltammetry performed on **Bi-1·Cl** (−1.5 to +2.5 V, 100 mV/s, TBAPF_6_ 0.1 M in acetonitrile was used as electrolyte) did not reveal a one-electron oxidation event. Exposure of **Bi-1·X** to a stoichiometric amount of NFSI at 90 °C in either CDCl_3_ or MeCN-*d*_3_ did not lead to the identification of new bismuth species, even if a loss of mass balance was observed (see Supporting Information for details). This data led us to speculate that initial oxidation is occurring through a 2-electron event from a fluoronium species.^32,33^

To investigate this hypothesis further, we conducted density functional theory (DFT) calculations with the model catalyst **Bi-4** at the B3LYP-D4/def2-TZVP//cosmo(∞)-BP86-D4/def2-TZVP level of theory (see computational details for more information), including solvation treatment with CHCl_3_ as solvent (COSMO-RS) (Figure 5C). For all species, extensive screening of coordination and conformational isomers was conducted. These calculations revealed a plausible stepwise two-electron oxidative addition process. After the transfer of a fluoronium (Δ*G* ^‡^ = 22.8 kcal·mol^–1^), the separated ions (**I1** ^**+**^ and **NSI** ^**–**^ 17.4 kcal·mol^–1^) can recombine in a strongly exergonic (Δ*G* = −13.7 kcal·mol^–1^), barrierless process giving the Bi­(V) intermediate **I2** (Figure S39). Overall, this step of the catalytic cycle appears to be rate limiting, validating the Bi­(III) resting state observed by NMR. Following oxidation to Bi­(V) **I2**, transmetalation of boronic acid **4** is proposed as reported in the literature (Figure S40).^31^

The final reductive elimination step was investigated by performing a series of stoichiometric experiments on bismuth complexes (Figure 5D). The transmetalated Bi­(III) complex **Bi-1·4-*t*BuPh** was prepared and oxidized by using XeF_2_ at 0 °C, affording **6** quantitatively. After drying under high vacuum in order to suppress any trace of the oxidant, complex **6** was mixed with NaNSI as a surrogate nucleophile of NFSI for 16 h at 90 °C. This mixture resulted in the expected combination of products (**2a** and **3a**) with yield and selectivity identical to those of the stoichiometric reaction of **Bi-1·4-*t*BuPh** (Figure 2), thus validating a reductive elimination step from high-valent bismuth. It is noteworthy that a combination of Ph_3_BiCl_2_ with NaNSI did not promote product formation, consistent with the requirement of a sulfone-type ligand backbone. Next, to model more accurately the catalytic cycle, the identified resting state **Bi-1·NSI** was oxidized to putative intermediate **7** prior to the addition of boronic acid **4a**. Gratifyingly, both C­(sp^2^)–N **2a** and C­(sp^2^)–O **3a** products could be detected by ^1^H NMR, consistent with our hypothesis of high-valent transmetalation followed by reductive elimination. Additional stoichiometric reactions to probe for parallel mechanistic pathways, including outer sphere generation of aryl radical or *N*-centered radical, ruled out such hypothesis (see details in Supporting Information).^34–36^

### Computational Analysis of Reductive Elimination Modes

Having defined a plausible catalytic cycle and limiting the putative pathways for reductive elimination, we next interrogated the origin of chemodivergence. Building on literature reports using NFSI as well as high-valent Bi reactivity, we considered three plausible scenarios to explain the ligand-controlled chemodivergency. First, a polar vs radical pathway was investigated (Figure 6A).^37^ In this case, a neutral Bi­(V) intermediate **I3** could either afford one product by the reported 5-membered ring reductive elimination or the other product after homolysis to **I4** and *ipso*-substitution by the formed *N*-centered radical (or in cage recombination of radicals after aryl radical formation from the Bi­(IV) species).^37^ Such a pathway was ruled out by DFT, as the resulting radical pair after homolysis is not feasible with +47.7 kcal·mol^–1^ relative to closed-shell Bi­(V) intermediate ***N*-I3** (Figure S43).

**Figure 6:**
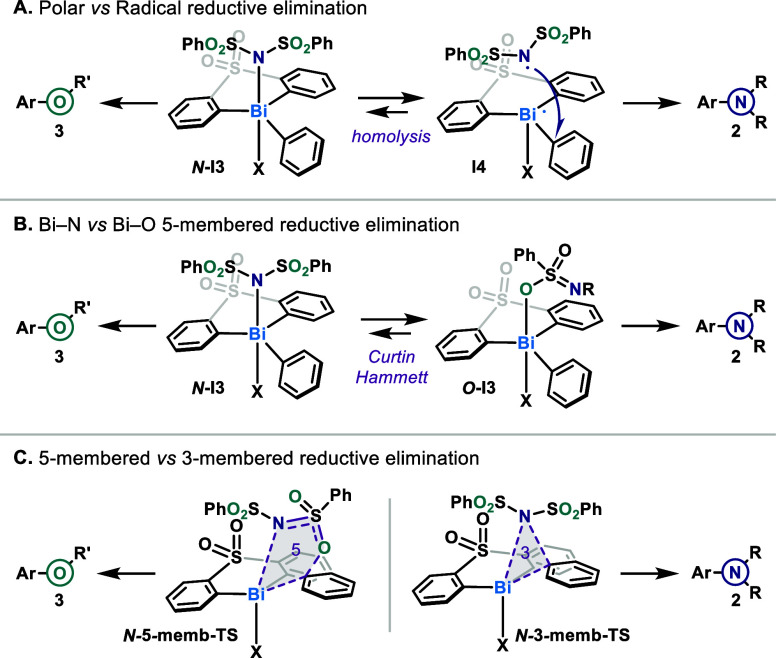
Selected reductive elimination pathways that lead to the observed chemodivergence. R = SO_2_Ph; R′ = S­(O)­NSO_2_Ph.

The second hypothesis relies exclusively on a previously reported 5-membered ring reductive elimination/sigmatropic rearrangement from a high-valent bismuth center (Figure 6B). Two complexes, one *O*-bound ***O*-I3** and one *N*-bound ***N*-I3**, could be in equilibrium, both leading to their corresponding products after reductive elimination in a Curtin-Hammett scenario. Finally, we considered a competition between a 3-membered and 5-membered ring reductive elimination from the same reaction intermediate (Figure 6C). Three-membered ring reductive elimination has been computationally shown to be feasible for Bi if performed in an asynchronous manner, thus circumventing the problematic symmetry-forbidden ligand–ligand coupling for pentacoordinated pnictogens.^12,17,38,39^ In a seminal report, Barton suggested that diarylether formation from phenol and tetraarylbismuthonium could result from an outer-sphere S_N_Ar-type reaction,^6c^ which can be seen as an extreme case of an asynchronous 3-membered ring reductive elimination (through prior dissociation of the nucleophile).^40,41^ Similar asynchronous apical-equatorial coupling has also been described for phosphonium intermediates.^42^

To interrogate scenarios B and C, DFT analysis of the various 3-membered and 5-membered ring transition states from *O*-bound and *N*-bound Bi­(V) intermediates ***O*-I3** and ***N*-I3** was performed (Figure S46 for more details). The sulfone-bearing ligands for the model catalyst **Bi-4** (Figure 7A), the sulfoximine model catalyst **Bi-5** (Figure 7B), and the most selective catalyst for the C­(sp^2^)–N coupling (**Bi-2**) (Figure 7C) were investigated. The five-membered ring transition states proceed in a concerted and synchronous manner (Figures S45 and S48). This agrees with our previous studies in C–F bond formation.^17^ In the latter case, because of the destabilization of charge separation, electron-deficient arenes were reluctant to undergo such ligand coupling. Due to the analogous 5-membered ring pathway in the present C–O coupling, electron-poor aryl groups should also exhibit low reactivity through this reductive elimination mode. In the three-membered ring reductive elimination scenario, asynchronous transition states were found leading to both products **2** and **3** depending on NSI coordination in intermediate **I3**. Here, the Bi–N or Bi–O bond is broken first, then the subsequent coupling occurs (Figures S48 and S51).^43^

**Figure 7:**
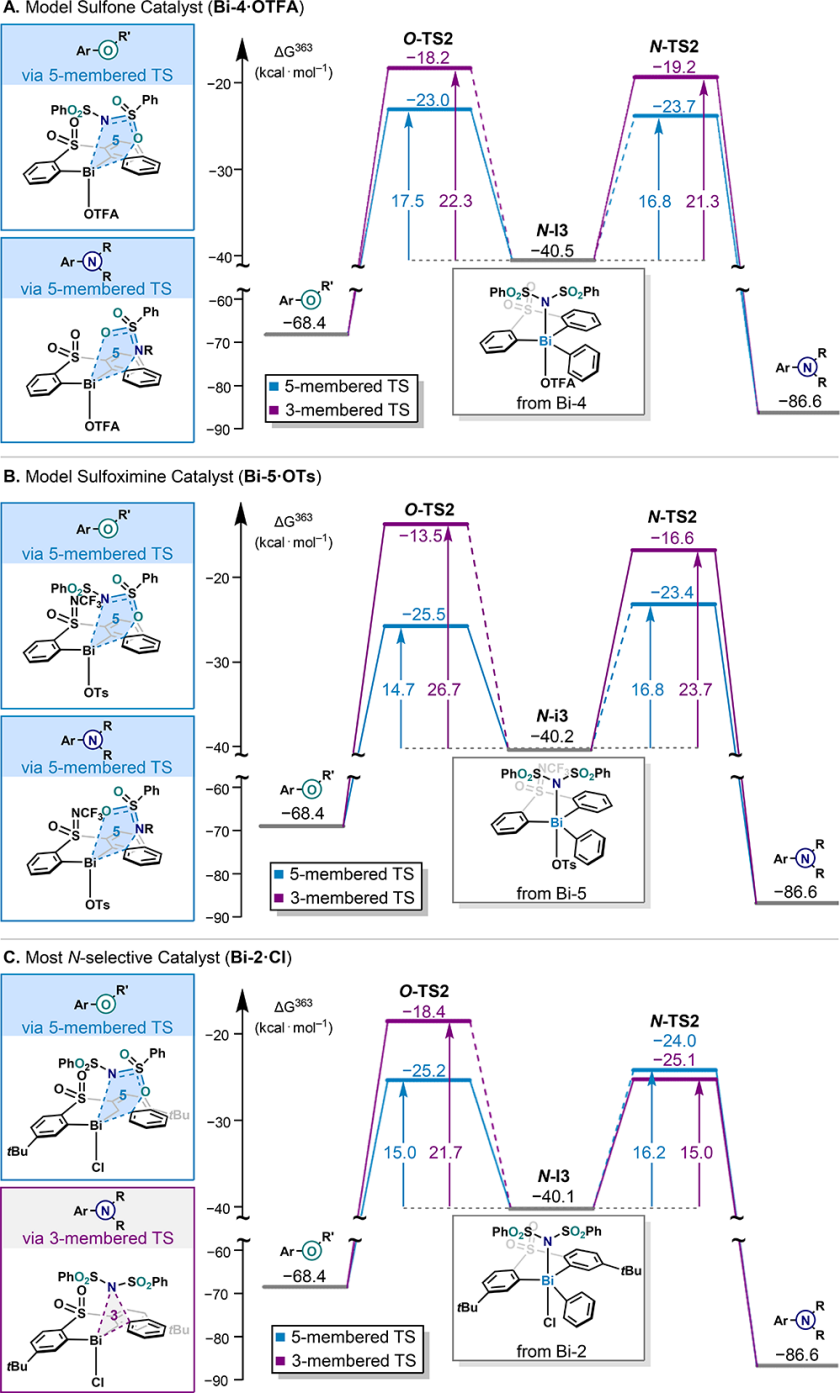
Free energy diagram for the key step of the reductive elimination for model sulfone catalyst (A), model sulfoximine catalyst (B), and catalyst with the optimal C–N selectivity (C). For simplification, the equilibria to ***O*-I3** are not shown in the Figure and indicated by a dotted line, see Supporting Information Figures S44–S54 for the full picture; the *t*Bu group at the aryl substituent is not shown for clarity; Δ*G* ^363^ in kcal·mol^–1^; B3LYP-D4/def2-TZVP//cosmo(∞)-BP86-D4/def2-TZVP; COSMO-RS (chloroform). R = SO_2_Ph; R′ = S­(O)­NSO_2_Ph.

For both sulfone catalysts examined in Figure 7 (**Bi-2** and **Bi-4**), the *N*-bound Bi­(V) intermediate ***N*-I3** is significantly more stable than the *O*-bound intermediate ***O*-I3** (by 8.0 kcal·mol^–1^ for **Bi-4** and 3.9 kcal·mol^–1^ for **Bi-2**). Thus, an initial equilibration of the *N*-bound intermediate is expected. Conversely, the *N*- and *O*-bound Bi­(V) intermediates derived from the sulfoximine **Bi-5** are almost isoenergetic (within 1 kcal·mol^–1^). In every case, the C­(sp^2^)–O product **3** was preferentially obtained through a five-membered reductive elimination transition state (effective activation Gibbs free energy, Δ*G* ^‡^_eff_ = 17.5 kcal·mol^–1^ for **Bi-4**, Δ*G* ^‡^_eff_ = 14.7 kcal·mol^–1^ for **Bi-5**, and Δ*G* ^‡^_eff_ = 15.0 kcal·mol^–1^ for **Bi-2**). Sulfoximine catalysts experimentally showed a greater selectivity toward the C­(sp^2^)–O product **3** than the sulfone analogues (see Tables S3 and S5). This experimental trend is also observed computationally in the energy profiles of **Bi-4** and **Bi-5**.

Although both catalysts share identical backbone substitutions with a similar anion, sulfoximine **Bi-5** leads to a greater selectivity for the C­(sp^2^)–O product with ΔΔ*G* ^‡^ = 2.1 kcal·mol^–1^ compared to ΔΔ*G* ^‡^ = –0.7 kcal·mol^–1^ with **Bi-4**. By contrast, investigation of the C­(sp^2^)–N bond formation showed different behaviors between catalysts. In the case of catalysts **Bi-4** and **Bi-5**, the C­(sp^2^)–N coupling proceeds via a five-membered transition state akin to that observed with the C­(sp^2^)–O coupling (Δ*G* ^‡^_eff_ = 16.8 kcal·mol^–1^ for **Bi-4**), in preference by 4.5 kcal·mol^–1^ compared to the parent three-membered transition state. With catalyst **Bi-2**, there is less difference between the effective Gibbs free energy of activation, and the three-membered ring transition state is preferred now by 1.2 kcal·mol^–1^. Consequently, for catalyst **Bi-2** the five- and three-membered transition states leading to reductive elimination are predicted to be competitive. Overall, this analysis of the reductive elimination potential energy surfaces for catalysts **Bi-4**, **Bi-5,** and **Bi-2** validates the feasibility of both five- and three-membered transition states described in Figure 6C. However, depending on the catalyst, a competition with scenario B is expected.

### Statistical Modeling

The complexity of the mechanistic calculations using DFT for this reaction considering multiple competing pathways, including one with possible charge separation, approaches the limits of computational analysis. To overcome these challenges and to gain more insights into the observed ligand-dependent selectivity, we elected to investigate the effect of ligand structure on reaction outcome using statistical modeling techniques. For this analysis, the experimental selectivities in chloroform were selected, since this data set was most extensive. Using multivariate linear regression (MLR), the ratio of the measured C­(sp^2^)–N and C­(sp^2^)–C coupling products formed was converted into a (measured) ΔΔ*G* ^‡^ in kcal·mol^–1^. Here, a larger negative ΔΔ*G* ^‡^ would translate to a higher selectivity for the C­(sp^2^)–N coupled product. To define the molecular features for MLR-modeling, a conformational search was performed on each complex followed by DFT optimization and free energy ranking using the same level of theory as for the mechanistic analysis (B3LYP-D4/def2-TZVP//cosmo(∞)-BP86-D4/def2-TZVP; COSMO-RS CHCl_3_; for full details on the computational workflow, see Supporting Information). The lowest energy conformer was chosen for the calculation of geometrical, steric, and electronic features. In this process, the ligands bearing sulfoximine backbones (including **Bi-3**) were discarded due to the significant structural and feature differences compared with the sulfone-containing ligands. For all models, a training/validation split of 12:3 was employed.

Initially, we sought to generate MLR models (using up to two features and searching all possible feature combinations) with the Ar_2_Bi­(III)–X complexes with observed selectivity. However, no satisfactory models were found. This suggested that the features of the Bi­(III) resting state were unable to capture the complex nature of the selectivity determining reductive elimination step (vide infra, Figure S25, see the Supporting Information for details on the different MLR models). Therefore, we explored the use of a series of Bi­(V) complexes with the substrate *N*-bound to extract molecular features, as this step may better represent the reductive elimination event. Unfortunately, this analysis did not result in readily interpretable models (see Figure S27 for examples). We then turned to utilization of the bismuthonium cation complex (Figure 8, **I3** ^**+**^) to extract molecular features. The descriptors derived from this complex produce a good MLR model with adequate training and cross-validation statistics (*R* ^2^ = 0.78; mean absolute error, MAE = 0.06 kcal·mol^–1^, leave-one-out cross-validation *Q* ^2^ = 0.59). Furthermore, the validation set was predicted relatively well (*R* ^2^ = 0.62 and MAE = 0.07 kcal·mol^–1^). At first glance, the link between such an intermediate, in which the ambident nucleophile is dissociated, and the selectivity is difficult to rationalize. However, the bismuthonium intermediates may reflect a three-membered ring asynchronous reductive elimination, where a positive charge is first formed on the Bi center (Figure 8A).

**Figure 8:**
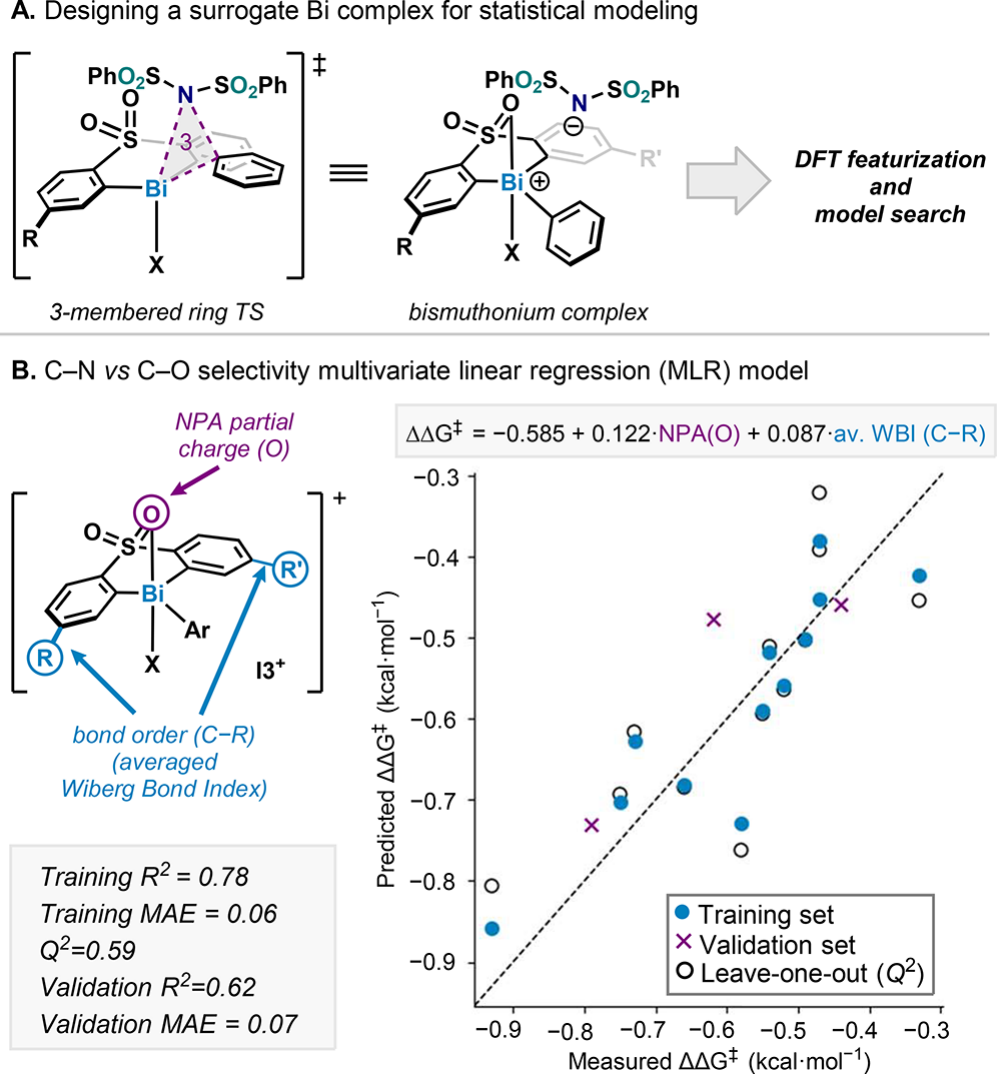
Descriptor modeling workflow (A) for the modeling of the bismuthonium (**I3** ^**+**^) as well as the MLR model (B); the *t*Bu group at the aryl substituent is not shown for clarity.

To explore this in more detail, we analyzed the molecular features determined by the MLR model: the atomic partial charge of the O atom coordinated to Bi (derived from a natural population analysis calculation) and the average (of both sides of the ligand) Wiberg bond index (WBI) for the C–R bond “meta” to Bi on the ligand aryls.

Examining the feature coefficients of the regression equation, a more negative partial charge of the O and a smaller WBI lead to a higher selectivity for formation of the C­(sp^2^)–N coupled product **2**. Aligning these findings with the computational studies in Figure 7, the more negative partial charge on O, which leads to enhanced coordination of this group to Bi, is proposed to impart greater stability to a three-membered reductive elimination transition state.^44^ Interpretation of the WBI, however, was less straightforward. Probing the relationship between this feature and ΔΔ*G* ^‡^ indicated that it was acting as a classifier for different ligand arene substitution patterns on the ligand (Figure S32). As an additional step, the WBI was regressed with two other ligand features to investigate what this feature reads out. A good correlation (*R* ^2^ = 0.92) was found with the two Sterimol parameters. Both features are the *L*-axis of the Sterimol, the longest length measured from the starting atom. The L component of the C–R to the substituted position at the ligand arene (averaged for C–R and C–R′ if R≠R′) shows a positive correlation and the L component of the C^2^–C^3^ bond (maximal value of both ligand substitution positions) a negative correlation. From this, the interpretation of the WBI can be aided such that it describes the length and width of the substituents on the “meta” position of the ligand. The use of these two ligand-based^45^ features in the MLR statistical modeling above supports the hypothesized of ligand-controlled C­(sp^2^)–N vs C­(sp^2^)–O selectivity. The success of the bismuthonium (**I3** ^**+**^) species featurization and statistical modeling is most consistent with a favored three-membered ring reductive elimination transition state, which we speculate favors the C­(sp^2^)–N coupling product **2** generation.

The combination of the statistical modeling, computational studies, and mechanistic experiments provides us with the basis to suggest a catalytic cycle (Figure 9). First, the Bi­(III) catalyst resting state, Ar_2_Bi­(III)–X, is oxidized in a two-electrons stepwise mechanism by NFSI **1**. The counterion can exchange at this point in the cycle to NSI. The resultant fleeting intermediate **I2** undergoes rapid transmetalation with the boron-based nucleophile **4** affording Bi­(V) complex ***N*-I3** where the nucleophile is *N*-bound. From this intermediate, the product-determining step occurs with a competition between a 5-membered ring reductive elimination producing the C­(sp^2^)–O product **3** or a 3-membered ring reductive elimination leading to the C­(sp^2^)–N product **2**. Such divergence can be explained by stabilization from the backbone to a transient positive charge as well as steric factors. Nonetheless, other pathways, including outer-sphere substitution or 5-membered ring reductive elimination from an *O*-bound intermediate, cannot be fully excluded.

**Figure 9:**
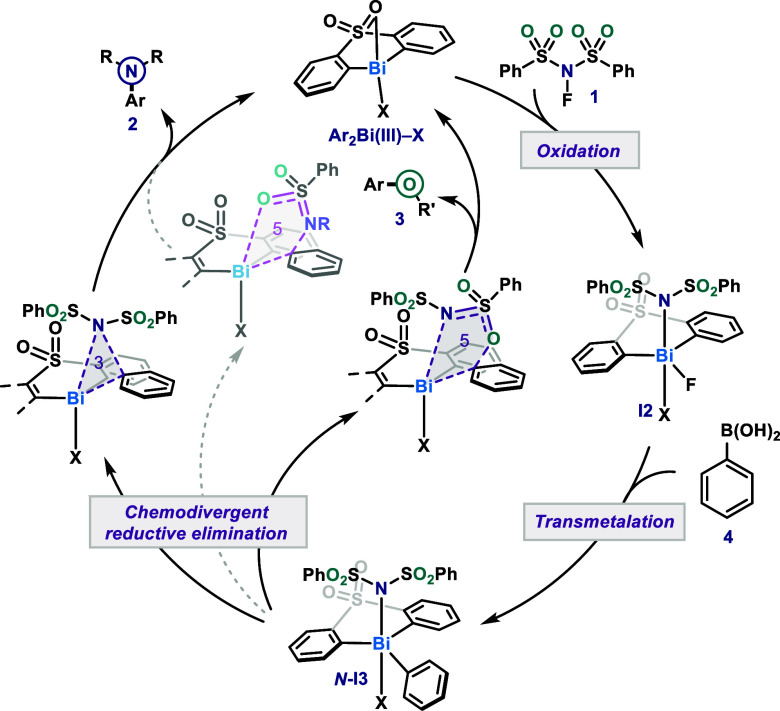
Suggested mechanistic cycle for Bi­(III)/Bi­(V) C­(sp^2^)–N/C­(sp^2^)–O chemodivergent coupling. *R* = SO_2_Ph; *R*′ = S­(O)­NSO_2_Ph.

## Conclusion

In summary, we reported a C­(sp^2^)–N/C­(sp^2^)–O chemodivergent coupling that operates through a catalytic high-valent bismuth redox cycle. The selectivity of the reaction could be switched by simple changes to a sulfone-like ligand framework, hence achieving ligand-controlled chemodivergence in main group catalysis. The transformation could be performed using a range of *N*-fluorosulfonimide derivatives (**1**) with diversely substituted boronic acids **4**. The catalytic process is postulated to proceed through an initial oxidation step followed by a transmetalation at the Bi­(V) center.^31^ The origin of the chemodivergence was then analyzed using a combination of computational methods wherein statistical analysis supports the formation of a bismuthonium cation as a crucial intermediate. We were able to successfully correlate the properties of such intermediates to the reaction outcomes. This proposal was further supported by DFT studies demonstrating that a competition between 3-membered and 5-membered ring ligand coupling/reductive elimination was responsible for the observed divergence. Ligand stabilization of this high-valent bismuthonium intermediate allows access to the two chemodivergent bond-forming modes of reductive elimination. These novel pathways in bismuth catalysis should provide the blueprint to exploit new catalyst design strategies as they are applied to future reactions of interest.

## Supplementary Material





## Notes

Experimental procedures, analytical data (^1^H, ^19^F, and ^13^C NMR, HRMS) for all new compounds, optimization details, supplementary mechanistic investigations, and computational details (PDF)
*Xyz* coordinates of the computed structures and a spreadsheet with all calculated descriptors and absolute free energies (ZIP)

## #

Department of Chemistry, University of Bath, Bath BA2 7AY, U.K
