# Supplementary material for: Ligand-Controlled Chemodivergent Bismuth Catalysis
Source: J Am Chem Soc. 2025 Nov 7;147(46):42406–15. doi: 10.1021/jacs.5c11854 (PMC12636031; doi:10.1021/jacs.5c11854)
Supplement: Supplementary file 1 [file ja5c11854_si_001.pdf]

Supporting Information

Ligand-Controlled Chemodivergent Bismuth Catalysis

Lucas Mele,[a,†] Philipp D. Engel,[b,c,d,†] Jamie A. Cadge,[b,e] Vytautas Peciukenas,[a] Hoonchul Choi,[a] Matthew S. Sigman,[*b] and Josep Cornella[*a]

[a] Max-Planck-Institut für Kohlenforschung, Kaiser-Wilhelm-Platz 1, Mülheim an der Ruhr, 45470, Germany.

[b] Department of Chemistry, University of Utah, 315 1400 E, Salt Lake City, Utah 84112, United States.

[c] Catalysis Research Laboratory (CaRLa), Im Neuenheimer Feld 271, 69120 Heidelberg, Germany.

[d] BASF SE, Carl-Bosch-Str. 38, 67056 Ludwigshafen, Germany.

[e] Department of Chemistry, University of Bath, Bath BA2 AY, United Kingdom.

cornella@kofo.mpg.de

Supporting Information

1. General considerations 4
2. Catalysts preparation 5
   2.1. Synthesis of phenylbismines 5
   2.2. Synthesis of bismines trifluoroacetate 7
   2.3. Synthesis of bismines tosylate 9
   2.4. Synthesis of chlorobismines 9
   2.5. Synthesis of tetrafluorobismine **Bi-1·BF$_4$** 10
   2.6. Synthesis of bromobismine **Bi-1·Br** 11
   2.7. Preparation of sulfinimidobismine **Bi-1·NSI** 11
   2.8. Other catalysts 12
3. HPLC method 13
4. Reaction optimization 20
   4.1. C–N selectivity, optimization 20
   4.2. C–O selectivity, optimization 23
5. Reaction scope 24
   5.1. Product characterization 24
   5.2. Unsuccessful substrates 31
6. Reproducibility issues 32
7. Additives effects 33
   7.1. Stilbene 33
   7.2. Si(OEt)$_4$ 34
8. Monitoring 35
9. Stoichiometric experiments 40
   9.1. Stoichiometric reactivity of Bi(III)–Ar with NFSI 40
   9.2. Products stability 41
   9.3. Transmetalation studies 44
   9.4. Oxidative addition 45
   9.5. Reaction with an external oxidant 46
   9.6. Stoichiometric reductive elimination 46
   9.7. Other reaction pathways 50
10. Electrochemical data 52
11. Computational Chemistry 54
   11.1. Computational Details 54

11.2. Feature Calculation 54
11.3. Statistical Modeling 55
11.4. Oxidative Addition 66
11.5. Transmetallation 66
11.6. Bi(V) Intermediate **I3**: Coordination Isomers 67
11.7. Reductive Elimination: Pathway B 68
11.8. Reductive Elimination: Pathway A and C 68
12. Crystallographic Data 79
13. NMR Spectra 84
14. References 166

1. General considerations

Unless otherwise stated, all manipulations were performed under argon using standard Schlenk-line techniques or in an argon-filled glovebox.

Instruments

NMR data were recorded using Bruker AVIII HD 300 MHz, Bruker AVIII HD 400 MHz, Bruker AVIII 500 MHz, or Bruker AVNeo 600 MHz NMR spectrometers (at 298-300 K, unless stated otherwise). ^{1}H and ^{13}C chemical shifts are reported in ppm relative to the solvent residual peaks as an internal reference. For ^{1}H NMR the following residual proton peaks of the deuterated solvents were used: $CDCl_3$, $\delta_H(CHCl_3)$ 7.260; CD_3CN, $\delta_H(CHD_2CN)$ 1.940. For ^{13}C NMR: $CDCl_3$, δ 77.16; CD_3CN, δ 1.32. ^{13}C spectra were acquired with broadband ^{1}H decoupling unless mentioned otherwise. Chemical shifts (δ) are given in ppm, relative to deuterated solvent residual peak, and coupling constants (*J*) provided in Hz. ^{19}F NMR shifts are reported relative to the ^{19}F resonances of $CFCl_3$. ^{19}F data at 282 MHz NMR is generally reported with ^{1}H decoupling. Chromatographic purifications were performed by flash column chromatography using Merck silica gel 60 (40-63 μm). High-resolution mass spectra were obtained using Bruker APEX III FT-MS (ESI ionization source), Finnigan MAT 95 (EI ionization source), Thermo Scientific Q Exactive G Orbitrap GC-MS/MS (GC-MS with EI ionization source), or Thermo Scientific Q Exactive Plus (APPI ionization source).

Solvents and reagents

Anhydrous MeCN was purchased from Sigma-Aldrich, opened and stored in an argon-filled glovebox. Under argon, $CDCl_3$ was pass through aluminium oxide 90 active neutral (70 – 230 mesh) and dried on 3 Å molecular sieves for at least 72 h prior to use. Smaller amounts of MeCN-d_3 and THF-d_8 were purchased and submitted to 3 freeze-pump-thaw cycles, vacuum distilled over CaH_2, introduced into an argon-filled glovebox, and stored under activated 3 Å molecular sieves. Molecular sieves were activated at 250 °C under high vacuum for 1 day (pressure reading of vacuum line remained steady at 2×10^{-3} mbar for at least 5 h). Unless otherwise stated, solvents used for the preparation of the ligands, catalysts or starting materials were also anhydrous, but not deoxygenated nor stored over molecular sieves prior to use.

Anhydrous $BiBr_3$ (99.9%, trace metal basis) was purchased from Sigma-Aldrich and stored in the glovebox. N-Fluorobenzenesulfonimide (98%) was purchased from BLD Pharm and used without further purification. Unless otherwise noted, all reagents were obtained from commercial suppliers and used without further purification.

2. Catalysts preparation

2.1. Synthesis of phenylbismines

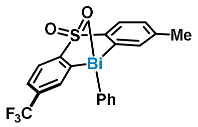
Bi-1·Ph

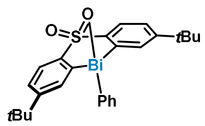
Bi-2·Ph

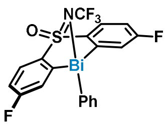
Bi-3·Ph

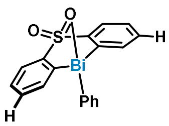
Bi-4·Ph

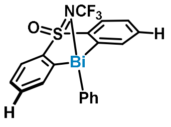
Bi-5·Ph

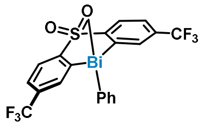
Bi-6·Ph

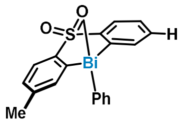
Bi-7·Ph

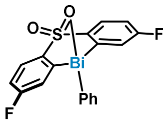
Bi-8·Ph

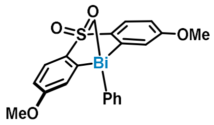
Bi-9·Ph

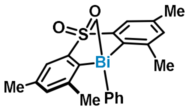
Bi-10·Ph

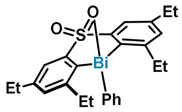
Bi-11·Ph

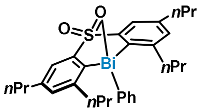
Bi-12·Ph

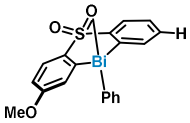
Bi-13·Ph

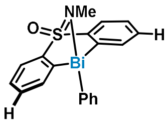
Bi-14·Ph

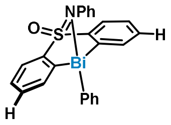
Bi-15·Ph

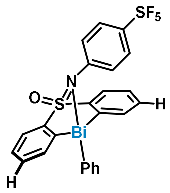
Bi-16·Ph

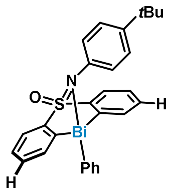
Bi-17·Ph

Phenylbismines **Bi-1·Ph**, **Bi-2·Ph**, **Bi-3·Ph**, **Bi-4·Ph**, **Bi-5·Ph**, **Bi-6·Ph**, **Bi-7·Ph**, **Bi-9·Ph**, **Bi-10·Ph**, **Bi-13·Ph**, **Bi-14·Ph**, **Bi-15·Ph**, **Bi-16·Ph** and **Bi-17·Ph** were already described and were prepared according to the reported procedure.[1,2,3]

General procedure for the synthesis of phenylbismines Bi·Ph.

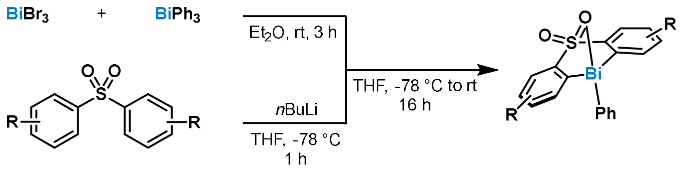

While working in an argon-filled glovebox, a first flame-dried Schlenk flask **A** equipped with a Teflon-coated stir bar was charged with $BiBr_3$ (0.66 equiv) and $BiPh_3$ (0.33 equiv.). The flask was ported out of the glovebox, connected to an argon/vacuum Schlenk line, and placed under an atmosphere of argon. Anhydrous and deoxygenated diethyl ether (0.7 M) was added to the flask **A** and the reaction was stirred at room temperature for 3 h. *Note: formation of a yellow precipitate was observed.* In parallel, a second flame-dried Schlenk flask **B** equipped with a Teflon-coated stir bar was charged with the corresponding diarylsulfone ligand (1.00 equiv.). The flask was connected to an argon/vacuum Schlenk line and was evacuated and refilled with argon (3 cycles). Anhydrous and degassed THF (0.2 M) was added to the flask **B** and the reaction was cooled to –78 °C (dry ice/acetone cooling bath). Under stirring, *n*-butyllithium (1.6 M solution in hexanes, 2.1 equiv.) was slowly added to the flask **B** while keeping the reaction temperature below –60 °C. After addition, the flask **B** was stirred at –78 °C for 1 h to prepare the dilithiated ligand. Anhydrous and degassed THF (*ca.* 0.6 M) was added to the flask **A** to completely dissolve the yellow precipitate, and this solution was added dropwise at –78 °C to the flask **B** containing the dilithiated ligand. Following the addition of the bismuth compound, the reaction was stirred

overnight while being allowed to slowly warm up to room temperature. Then, the reaction was quenched with brine (50 volumes), transferred to a separatory funnel, extracted with ethyl acetate (50 volumes), and re-extracted with CH_2Cl_2 (2 × 50 volumes). The combined organic layers were dried over $MgSO_4$, filtered on Celite and the filtrate cake was extracted with CH_2Cl_2. The filtrate was concentrated with a rotary evaporator to give a thick yellow residue. The crude material was purified by flash column chromatography (silica gel, hexanes/ethyl acetate) to give the corresponding arylbismine as a white solid.

2,8-difluoro-10-phenyl-10H-dibenzo[b,e][1,4]thiabismine 5,5-dioxide (Bi-8·Ph)

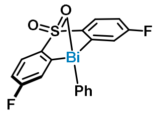

Following the general procedure, starting from 0.98 g of 4,4'-sulfonylbis(fluorobenzene) (3.85 mmol), **Bi-8·Ph** (1.35 g, 34% yield) was isolated as a white solid after purification by column chromatography (hexanes/EtOAc = 4:1).

^{1}H NMR (300 MHz, $CDCl_3$) δ 8.38 (dd, J = 8.6, 4.7 Hz, 2H), 7.78 (dd, J = 7.9, 1.4 Hz, 2H), 7.58 (dd, J = 7.0, 2.5 Hz, 2H), 7.54 – 7.37 (m, 3H), 7.08 (td, J = 8.5, 2.5 Hz, 2H).

^{13}C NMR (101 MHz, $CDCl_3$) δ 168.0, 164.6 (d, J = 260.7 Hz), 164.5, 138.7, 137.6 (d, J = 2.8 Hz), 131.5, 129.6 (d, J = 8.4 Hz), 129.3, 124.7 (d, J = 20.9 Hz), 115.5 (d, J = 22.8 Hz).

^{19}F NMR (565 MHz, $CDCl_3$) δ –105.9 (td, J = 7.8, 4.9 Hz, 2F).

HRMS (ESI-Orbitrap) calc'd for $C_{18}H_{11}BiF_2SO_2Na$ $[M+Na]^+$: 561.0144, found: 561.0144.

1,3,7,9-tetraethyl-10-phenyl-10H-dibenzo[b,e][1,4]thiabismine 5,5-dioxide (Bi-11·Ph)

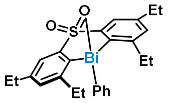

Following the general procedure, starting from 2.4 g of 5,5'-sulfonylbis(1,3-diethylbenzene) (7.2 mmol), **Bi-11·Ph** (2.5 g, 57% yield) was isolated as a white solid after purification by column chromatography (hexanes/EtOAc = 4:1).

^{1}H NMR (300 MHz, $CDCl_3$) δ 8.17 (d, J = 1.8 Hz, 2H), 7.70 (dd, J = 6.4, 2.9 Hz, 2H), 7.24 (dd, J = 5.0, 2.0 Hz, 3H), 7.19 (d, J = 1.8 Hz, 2H), 2.69 (q, J = 7.6 Hz, 4H), 2.35 (dq, J = 15.0, 7.5 Hz, 2H), 2.10 (dq, J = 14.9, 7.5 Hz, 2H), 1.26 (t, J = 7.6 Hz, 6H), 0.86 (t, J = 7.5 Hz, 6H).

^{13}C NMR (151 MHz, $CDCl_3$) δ 160.4, 154.0, 150.9, 145.0, 141.2, 138.6, 133.1, 130.7, 127.7, 124.2, 32.3, 28.6, 15.4, 15.0.

HRMS (ESI-Orbitrap) calc'd for $C_{26}H_{29}BiSO_2Na$ $[M+Na]^+$: 637.15942, found: 637.15845.

1,3,7,9-tetrapropyl-10-phenyl-10H-dibenzo[b,e][1,4]thiabismine 5,5-dioxide (Bi-12·Ph)

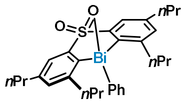

Following the general procedure, starting from 0.42 g of 5,5'-sulfonylbis(1,3-dipropylbenzene) (1.09 mmol), **Bi-12·Ph** (0.30 g, 41% yield) was isolated as a white solid after purification by column chromatography (hexanes/EtOAc = 4:1).

^{1}H NMR (600 MHz, $CDCl_3$) δ 8.14 (d, J = 1.7 Hz, 2H), 7.74 – 7.69 (m, 2H), 7.25 (dd, J = 4.5, 2.0 Hz, 4H), 7.14 (d, J = 1.8 Hz, 2H), 2.61 (td, J = 7.4, 2.2 Hz, 4H), 2.26 (ddd, J = 14.1, 10.1, 5.8 Hz, 2H), 2.04 (ddd, J = 14.1, 10.0, 5.8 Hz, 2H), 1.69 – 1.62 (m, 4H), 1.52 – 1.40 (m, 2H), 1.15 – 1.06 (m, 2H), 0.94 (t, J = 7.3 Hz, 6H), 0.69 (t, J = 7.3 Hz, 6H).

^{13}C NMR (151 MHz, $CDCl_3$) δ 160.6, 154.5, 149.5, 143.3, 141.3, 138.6, 134.3, 130.7, 127.8, 124.7, 41.1, 37.7, 24.4, 24.3, 14.1, 13.9.

HRMS (ESI-Orbitrap) calc'd for $C_{30}H_{37}BiSO_2Na$ $[M+Na]^+$: 693.2208, found: 693.2211.

2.2. Synthesis of bismines trifluoroacetate

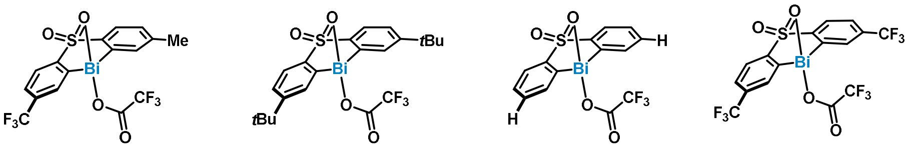

Bi-1·OTFA Bi-2·OTFA Bi-4·OTFA Bi-6·OTFA

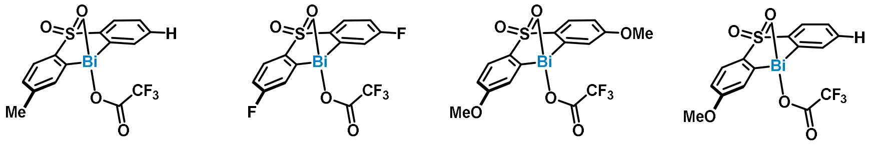

Bi-7·OTFA Bi-8·OTFA Bi-9·OTFA Bi-13·OTFA

Bismines trifluoroacetate **Bi-1·OTFA** and **Bi-6·OTFA** were already described and were prepared according to the reported procedure.[3]

General procedure for the synthesis of bismines trifluoroacetate Bi·OTFA.

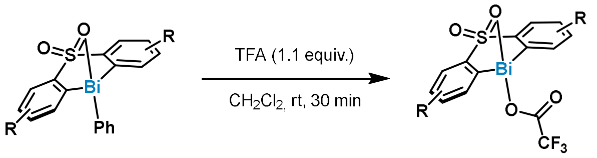

A flame dried culture tube equipped with a Teflon-coated stir bar was charged with phenylbismine (1.0 equiv.). The culture tube was sealed with a Teflon screw-cap, connected to an argon/vacuum Schlenk line and evacuated and refilled with argon (3 cycles). Anhydrous and degassed CH_2Cl_2 (0.1 M) was added through the septa followed by trifluoroacetic acid (1.1 equiv.). The reaction was stirred (600 rpm) for 30 min at room temperature. Then, the solvent was removed under vacuum, and the remaining solid was dried under high-vacuum (pressure reading on vacuum gauge stayed at maximum 1×10^{-2} mbar) for 2 h.

2,8-di-tert-butyl-5,5-dioxido-10H-dibenzo[b,e][1,4]thiabismin-10-yl 2,2,2-trifluoroacetate (Bi-2·OTFA)

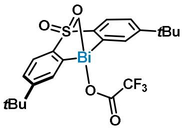

Following the general procedure, starting from 200 mg of 2,8-di-tert-butyl-10-phenyl-10H-dibenzo[b,e][1,4]thiabismine 5,5-dioxide **Bi-2·Ph** (0.325 mmol), **Bi-2·OTFA** (209 mg, 99% yield) was isolated as a white solid.

^{1}H NMR (400 MHz, $CDCl_3$) δ 8.80 (d, *J* = 1.8 Hz, 2H), 8.34 (d, *J* = 8.1 Hz, 2H), 7.49 (dd, *J* = 8.1, 1.8 Hz, 2H), 1.33 (s, 18H).

^{13}C NMR (101 MHz, $CDCl_3$) δ 185.9, 163.5 (q, *J* = 39.2 Hz), 159.6, 137.5, 132.8, 128.9, 126.1, 116.4 (q, *J* = 288.5 Hz), 36.0, 31.2.

^{19}F NMR (282 MHz, $CDCl_3$) δ –74.3 (s, 3F).

HRMS (ESI-Orbitrap) calc'd for $C_{22}H_{24}BiF_3SO_4Na$ $[M+Na]^+$: 673.1043, found: 673.1044.

5,5-dioxido-10H-dibenzo[b,e][1,4]thiabismin-10-yl 2,2,2-trifluoroacetate (Bi-4·OTFA)

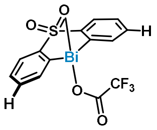

Following the general procedure, starting from 204 mg of 10-phenyl-10H-dibenzo[b,e][1,4]thiabismine 5,5-dioxide **Bi-4·Ph** (0.406 mmol), **Bi-4·OTFA** (214 mg, 98% yield) was isolated as a white solid.

^{1}H NMR (600 MHz, $CDCl_3$) δ 8.73 (dd, *J* = 7.4, 1.1 Hz, 2H), 8.41 (dd, *J* = 7.8, 1.2 Hz, 2H), 7.79 (td, *J* = 7.4, 1.3 Hz, 2H), 7.51 (td, *J* = 7.6, 1.1 Hz, 2H).

^{13}C NMR (151 MHz, $CDCl_3$) δ 186.1, 164.0 (q, *J* = 35.5 Hz), 140.2, 136.1, 135.6, 129.4, 129.2, 116.3 (q, *J* = 288.3 Hz).

^{19}F NMR (564 MHz, $CDCl_3$) δ –74.0 (s, 3F).

HRMS (ESI-Orbitrap) calc'd for $C_{14}H_8BiF_3SO_4Na$ $[M+Na]^+$: 560.9796, found: 560.9792.

2-methyl-5,5-dioxido-10H-dibenzo[b,e][1,4]thiabismin-10-yl 2,2,2-trifluoroacetate (Bi-7·OTFA)

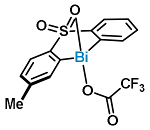

Following the general procedure, starting from 201 mg of 2-(tert-butyl)-10-phenyl-10H-dibenzo[b,e][1,4]thiabismine 5,5-dioxide **Bi-7·Ph** (0.389 mmol), **Bi-7·OTFA** (204 mg, 95% yield) was isolated as a white solid.

^{1}H NMR (300 MHz, $CDCl_3$) δ 8.70 (dd, *J* = 7.4, 1.1 Hz, 1H), 8.52 (dd, *J* = 1.5, 0.7 Hz, 1H), 8.38 (dd, *J* = 7.7, 1.2 Hz, 1H), 8.29 (d, *J* = 7.9 Hz, 1H), 7.77 (td, *J* = 7.5, 1.3 Hz, 1H), 7.49 (td, *J* = 7.6, 1.1 Hz, 1H), 7.28 (ddd, *J* = 7.8, 1.6, 0.8 Hz, 1H) 2.43 (s, 3H).

^{13}C NMR (75 MHz, $CDCl_3$) δ 185.9, 185.8, 163.7 (q, *J* = 39.2 Hz), 147.4, 140.7, 137.1, 136.1, 136.0, 135.5, 129.8, 129.5, 129.2, 129.1, 116.3 (q, *J* = 288.6 Hz), 22.1.

^{19}F NMR (282 MHz, $CDCl_3$) δ –74.0 (s, 3F).

HRMS (ESI-Orbitrap) calc'd for $C_{15}H_{10}BiF_3SO_4Na$ $[M+Na]^+$: 574.9947, found: 574.9948.

2,8-difluoro-5,5-dioxido-10H-dibenzo[b,e][1,4]thiabismin-10-yl 2,2,2-trifluoroacetate (Bi-8·OTFA)

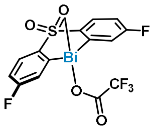

Following the general procedure, starting from 200 mg of 2,8-difluoro-10-phenyl-10H-dibenzo[b,e][1,4]thiabismine 5,5-dioxide **Bi-8·Ph** (0.372 mmol), **Bi-8·OTFA** (207 mg, 97% yield) was isolated as a white solid.

^{1}H NMR (400 MHz, $CDCl_3$) δ 8.53 (dd, *J* = 6.7, 2.4 Hz, 2H), 8.44 (dd, *J* = 8.5, 4.5 Hz, 2H), 7.16 (td, *J* = 8.4, 2.5 Hz, 2H).

^{13}C NMR (101 MHz, $CDCl_3$) δ 189.3 (d, *J* = 3.9 Hz), 168.9 (d, *J* = 263.5 Hz), 164.0 (q, *J* = 40.2 Hz), 135.9 (d, *J* = 3.0 Hz), 132.2 (d, *J* = 8.9 Hz), 123.6 (d, *J* = 22.3 Hz), 116.54 (d, *J* = 23.4 Hz), 116.50 (q, *J* = 287.7 Hz).

^{19}F NMR (282 MHz, $CDCl_3$) δ –75.0 (s, 3F), –105.4 (s, 2F).

HRMS (ESI-Orbitrap) calc'd for $C_{14}H_7BiF_5SO_4$ $[M+H]^+$: 574.9788, found: 574.9784.

2,8-dimethoxy-5,5-dioxido-10H-dibenzo[b,e][1,4]thiabismin-10-yl 2,2,2-trifluoroacetate (Bi-9·OTFA)

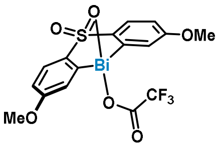

Following the general procedure, starting from 199 mg of 2,8-dimethoxy-10-phenyl-10H-dibenzo[b,e][1,4]thiabismine 5,5-dioxide **Bi-9·Ph** (0.354 mmol), **Bi-9·OTFA** (193 mg, 92% yield) was isolated as a white solid.

^{1}H NMR (300 MHz, $CDCl_3$) δ 8.37 (m, 4H), 7.13 (td, J = 8.7, 2.5 Hz, 2H), 3.86 (s, 6H).

^{13}C NMR (101 MHz, $CDCl_3$) δ 187.8, 166.3, 163.7 (q, J = 39.1 Hz), 131.9, 131.0, 120.7, 116.3 (q, J = 288.5 Hz), 114.5, 56.0.

^{19}F NMR (282 MHz, $CDCl_3$) δ –74.0 (s, 3F).

HRMS (ESI-Orbitrap) calc'd for $C_{16}H_{12}BiF_3SO_6Na$ $[M+Na]^+$: 621.0001, found: 621.0003.

2-methoxy-5,5-dioxido-10H-dibenzo[b,e][1,4]thiabismin-10-yl 2,2,2-trifluoroacetate (Bi-13·OTFA)

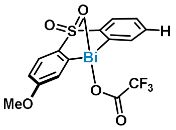

Following the general procedure, starting from 197 mg of 2-methoxy-10-phenyl-10H-dibenzo[b,e][1,4]thiabismine 5,5-dioxide **Bi-13·Ph** (0.370 mmol), **Bi-13·OTFA** (206 mg, 98% yield) was isolated as a white solid.

^{1}H NMR (300 MHz, $CDCl_3$) δ 8.70 (dd, J = 7.3, 1.1 Hz, 1H), 8.50 – 8.28 (m, 3H), 7.77 (td, J = 7.5, 1.3 Hz, 1H), 7.48 (td, J = 7.6, 1.1 Hz, 1H), 6.91 (dd, J = 8.6, 2.4 Hz, 1H), 3.86 (s, 3H).

^{13}C NMR (75 MHz, $CDCl_3$) δ 188.3, 185.7, 166.5, 163.9, 163.5 (q, J = 39.6 Hz), 141.3, 135.8, 135.6, 131.6, 130.8, 129.1, 128.9, 120.7, 116.3 (q, J = 288.5 Hz), 56.0.

^{19}F NMR (282 MHz, $CDCl_3$) δ –74.0 (s, 3F).

HRMS (ESI-Orbitrap) calc'd for $C_{15}H_{10}BiF_3SO_5Na$ $[M+Na]^+$: 590.9896, found: 590.9897.

2.3. Synthesis of bismines tosylate

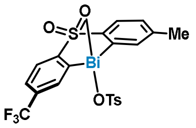

Bi-1·OTs

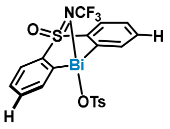

Bi-5·OTs

Bismines tosylate **Bi-1·OTs** and **Bi-5·OTs** were already described and were prepared according to the reported procedure.[3]

2.4. Synthesis of chlorobismines

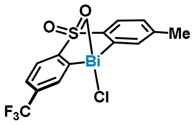

Bi-1·Cl

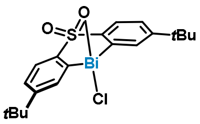

Bi-2·Cl

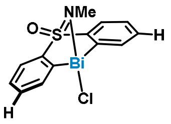

Bi-14·Cl

Chlorobismine **Bi-1·Cl** was already described and was prepared according to the reported procedure.[4]

General procedure for the preparation of chlorobismine Bi·Cl.

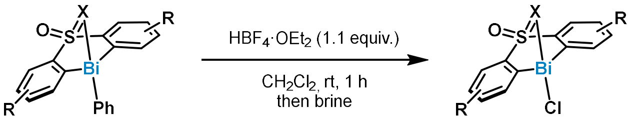

A flame dried culture tube equipped with a Teflon-coated stir bar was charged with phenylbismine (1.0 equiv.). The culture tube was sealed with a Teflon screw-cap, connected to an argon/vacuum Schlenk line and evacuated and refilled with argon (3 cycles). Anhydrous and deoxygenated CH_2Cl_2 (0.1 M) was added through the septa followed by tetrafluoroborate diethylether complex (1.1 equiv.). The reaction was stirred (600 rpm) for 1 h at room temperature. *Note: depending on the bismine substituents a white precipitate can appear.* After quantitative formation of the tetrafluoroborate salt, an equal volume of brine in respect to the solvent was added and the reaction was vigorously stirred (1200 rpm) until disappearance of the white precipitate (*ca* 10 min). Then, the reaction was transferred to a separatory funnel and the organic layer was collected. The aqueous layer was extracted with CH_2Cl_2 (2 × 10 volumes) and the combined organic layers was concentrated under reduced pressure. The obtained solid was dried under high-vacuum (pressure reading on vacuum gauge stayed at maximum 1 × 10^{-2} mbar) for 2 h.

2,8-di-tert-butyl-10-chloro-10H-dibenzo[b,e][1,4]thiabismine 5,5-dioxide (Bi-2·Cl)

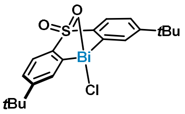

Following the general procedure, starting from 200 mg of 2,8-di-tert-butyl-10-phenyl-10H-dibenzo[b,e][1,4]thiabismine 5,5-dioxide **Bi-2·Ph** (0.326 mmol), **Bi-2·Cl** (175 mg, 94% yield) was isolated as a white solid.

^{1}H NMR (600 MHz, $CDCl_3$) δ 8.89 (dd, *J* = 1.9, 0.4 Hz, 2H), 8.28 (dd, *J* = 8.2, 0.4 Hz, 2H), 7.46 (dd, *J* = 8.1, 1.8 Hz, 2H), 1.35 (s, 18H).

^{13}C NMR (151 MHz, $CDCl_3$) δ 178.7, 159.1, 137.7, 133.4, 128.1, 125.9, 36.0, 31.3.

HRMS (ESI-Orbitrap) calc'd for $C_{20}H_{24}BiClSO_2Na$ $[M+Na]^+$: 595.0882, found: 595.0887.

10-chloro-5-(methylimino)-5,10-dihydro-5l4-dibenzo[b,e][1,4]thiabismine 5-oxide (Bi-14·Cl)

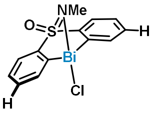

Following the general procedure, starting from 516 mg of 5-(methylimino)-10-phenyl-5,10-dihydro-5l4-dibenzo[b,e][1,4]thiabismine 5-oxide **Bi-14·Ph** (1.00 mmol), **Bi-14·Cl** (152 mg, 32% yield) was isolated as a white solid.

^{1}H NMR (600 MHz, DMSO) δ 8.81 (d, *J* = 7.3 Hz, 2H), 8.14 (dd, *J* = 7.7, 1.2 Hz, 2H), 7.78 (td, *J* = 7.3, 1.2 Hz, 2H), 7.56 (td, *J* = 7.6, 1.2 Hz, 2H), 2.62 (s, 3H).

^{13}C NMR (151 MHz, DMSO) δ 182.3, 139.1, 136.2, 134.5, 129.5, 128.1, 28.1.

HRMS (ESI-Orbitrap) calc'd for $C_{13}H_{12}BiClNSO$ $[M+H]^+$: 474.0127, found: 474.0130.

2.5. Synthesis of tetrafluorobismine **Bi-1·BF4**

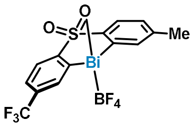

Bi-1·BF4

Tetrafluorobismine **Bi-1·BF4** was already described and was prepared according to the reported procedure.[2]

2.6. Synthesis of bromobismine **Bi-1·Br**

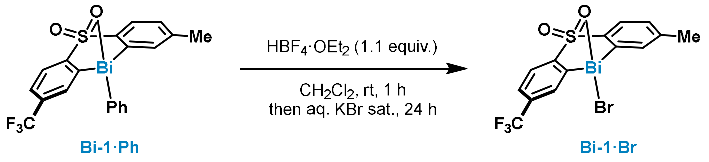

A flame dried culture tube equipped with a Teflon-coated stir bar was charged with phenylbismine **Bi-1·Ph** (500 mg, 0.856 mmol, 1.0 equiv.). The culture tube was sealed with a Teflon screw-cap, connected to an argon/vacuum Schlenk line and evacuated and refilled with argon (3 cycles). Anhydrous and degassed CH_2Cl_2 (8.5 mL, 0.1 M) was added through the septa followed by tetrafluoroborate diethylether complex (128 µL, 0.941 mmol, 1.1 equiv.). The reaction was stirred (600 rpm) for 1 h at room temperature. *Note: a white precipitate was observed.* After quantitative formation of the tetrafluoroborate salt, an aqueous saturated KBr solution (8 mL) was added and the reaction was vigorously stirred (1200 rpm) until disappearance of the white precipitate (*ca.* 24 h). Then, the reaction was transferred to a separatory funnel and the organic layer was collected. The aqueous layer was extracted with MTBE (3 × 10 mL) and the combined organic layers was concentrated under reduced pressure. The obtained white solid **Bi-1·Br** (500 mg, 99% yield) was dried under high-vacuum (pressure reading on vacuum gauge stayed at maximum 1 × 10^{-2} mbar) for 2 h.

^{1}H NMR (600 MHz, $CDCl_3$) δ 9.18 (dt, *J* = 1.8, 0.6 Hz, 1H), 8.82 – 8.80 (m, 1H), 8.39 (dt, *J* = 8.0, 0.7 Hz, 1H), 8.25 (dt, *J* = 7.9, 0.4 Hz, 1H), 7.73 (ddd, *J* = 8.0, 1.7, 0.7 Hz, 1H), 7.30 (ddd, *J* = 7.9, 1.6, 0.8 Hz, 1H), 2.46 (s, 3H).

^{13}C NMR (151 MHz, $CDCl_3$) δ 174.1, 173.3, 147.7, 144.4, 138.2, 137.0 (q, *J* = 33.4 Hz), 136.4, 134.6 (q, *J* = 3.6 Hz), 129.9, 128.9, 127.9, 126.2 (q, *J* = 3.7 Hz), 123.6 (q, *J* = 273.9 Hz), 22.0.

^{19}F NMR (565 MHz, $CDCl_3$) δ –62.8 (s, 3F).

HRMS (APPI-Orbitrap) calc'd for $C_{14}H_9BiBrSO_2F_3$ $[M]^{•+}$: 585.9257, found: 585.9256.

2.7. Preparation of sulfinimidobismine **Bi-1·NSI**

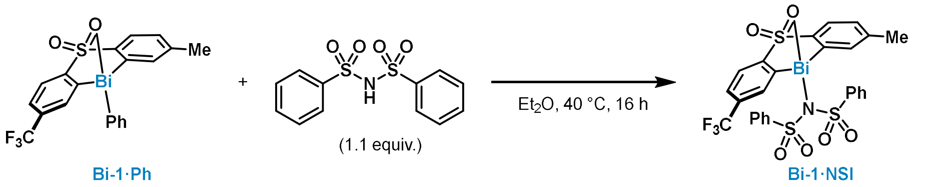

A flame dried culture tube equipped with a Teflon-coated stir bar was charged with phenylbismine **Bi-1·Ph** (200 mg, 0.342 mmol, 1.0 equiv.) and dibenzensulfonimide (112 mg, 0.378 mmol, 1.1 equiv.). The culture tube was sealed with a Teflon screw-cap, connected to an argon/vacuum Schlenk line and evacuated and refilled with argon (3 cycles). Anhydrous and deoxygenated Et_2O (1.5 mL) was added through the septa and the reaction was stirred (600 rpm) for 1 h at 40 °C. The crude reaction mixture was concentrated under reduced pressure and the obtained solid was washed with pentane (3 × 1 mL) The obtained white solid **Bi-1·NSI** (173 mg, 63% yield) was dried under high-vacuum (pressure reading on vacuum gauge stayed at maximum 1 × 10^{-2} mbar) for 2 h.

^{1}H NMR (300 MHz, $CDCl_3$) δ 9.20 (s, 1H), 8.85 (s, 1H), 8.52 (d, *J* = 8.0 Hz, 1H), 8.39 (d, *J* = 7.8 Hz, 1H), 7.80 (d, *J* = 7.8 Hz, 4H), 7.74 (d, *J* = 8.3 Hz, 1H), 7.50 (t, *J* = 7.4 Hz, 2H), 7.38 (t, *J* = 6.7 Hz, 4H), 7.31 (d, *J* = 8.1 Hz, 1H), 2.35 (s, 3H).

^{13}**C NMR** (101 MHz, $CDCl_3$) δ 193.7, 192.5, 147.9, 144.1, 141.1, 137.6 – 137.3 (m), 136.8, 135.9, 133.7, 132.6, 130.7, 129.9, 129.8, 129.1, 128.7, 128.2, 127.5, 126.1, 123.8 (q, J = 274.4 Hz), 22.1.

^{19}**F NMR** (282 MHz, $CDCl_3$) δ -62.7.

HRMS (ESI-Orbitrap) calc'd for $C_{26}H_{19}BiF_3NS_3O_6Na$ $[M+Na]^+$: 826.0025, found: 826.0023.

2.8. Other catalysts

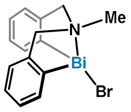

Bi-18

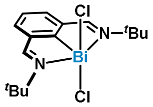

Bi-19

Complexes **Bi-18** and **Bi-19** were already described and were prepared according to the reported procedure.[5,6]

3. HPLC method

In order to determine the selectivity between the C(sp²)–N product **2** and the C(sp²)–O product **3**, the crude reaction mixtures were analyzed by RP-HPLC. First, an optimized method was determined by analyzing the pure reactions reagents and products in various conditions, aiming to avoid overlap in the different chromatogram. Chromatograms of the constituents of a model reaction in optimized condition are presented on **Figure S1**.

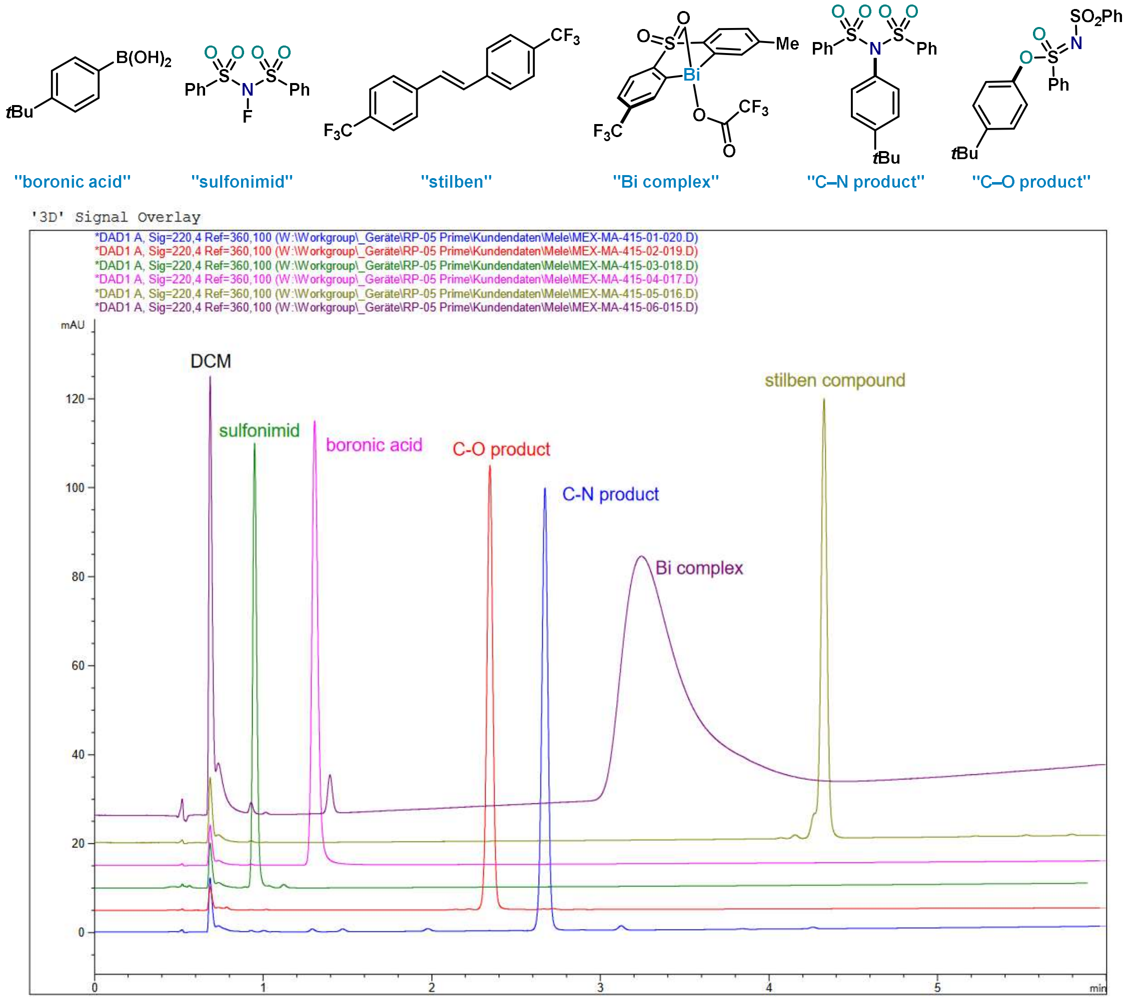

Figure S1 Chromatograms of the pure reagents and products in a model bismuth catalyzed reaction. Conditions: Zorbax Eclipse Plus, C18 1.8 µm, 4.6 x 50 mm; gradient 70% to 90% (5 min) MeOH/H_2O, 1.0 mL/min, 220 nm.

The optimized HPLC method was then try on a crude reaction mixture resulting from a typical catalysis (*vide infra* for detailed protocol). At the end of the reaction, an aliquot was filtered through a syringe filter (Carl Roth PTFE, 45 µm, 13 mm), concentrated at the rotary evaporator to afford about 1 mg of dry material. The dry extract was then dissolved in 400 µL of MeCN and 100 µL of DCM to afford a clear solution that was then use for HPLC analysis. Chromatogram of a typical crude mixture using the optimized HPLC condition is depicted in **Figure S2**.

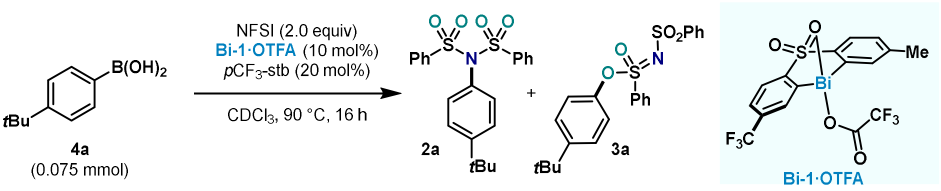

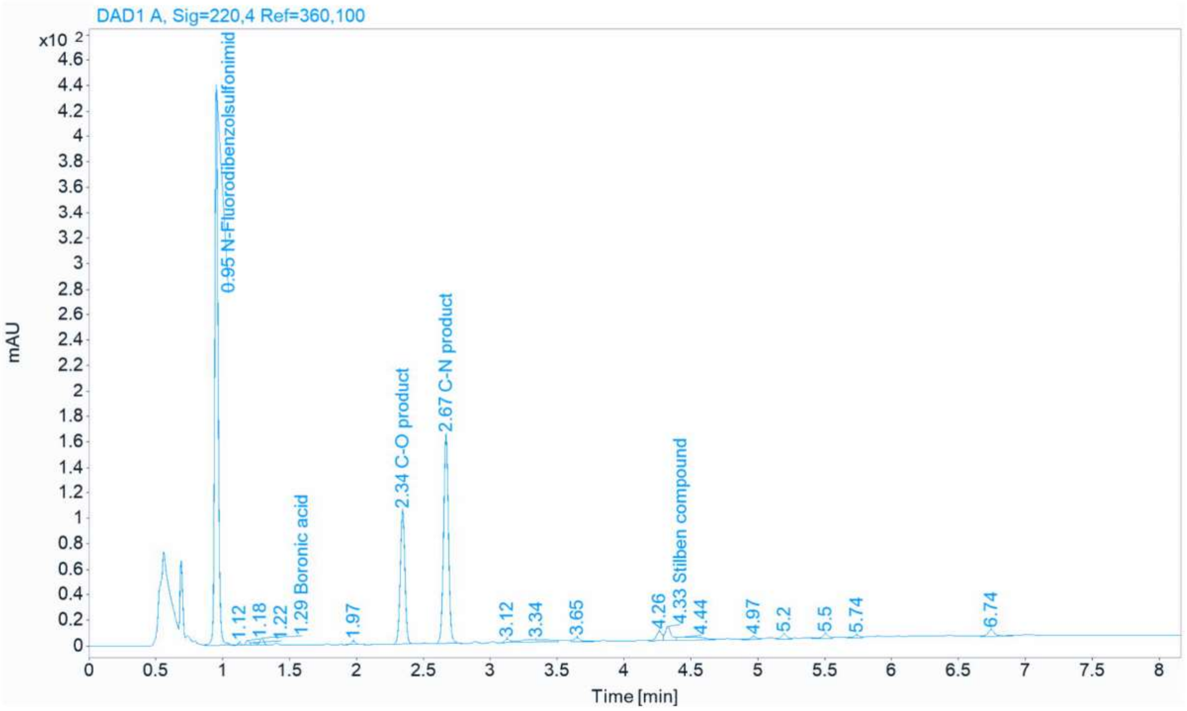

Figure S2 Chromatogram of the crude material of a model bismuth catalyzed reaction. Conditions: Zorbax Eclipse Plus, C18 1.8 µm, 4.6 x 50 mm; gradient 70% to 90% (5 min) MeOH/H_2O, 1.0 mL/min, 220 nm.

The ratios between the two products were then determined by relative integration of their corresponding signals after correction of their molar extinction coefficient.

Correction of molar extinction coefficient

Independently, for each example of the reaction scope, aliquots of both isolated pure products were combined using a pipette and dissolved in $CDCl_3$ with a known amount of CH_2Br_2 as an internal standard. The products relative concentrations (C_0) were then determined by quantitative ^{1}H NMR on clean signals. The solution was then analyzed by HPLC and the absorption were measured at $\lambda = 220$ nm. The procedure was then repeated two additional times at different concentration, typically aiming for $C_0/2$ and $C_0/4$. Finally, following non corrected Beer-Lambert law, the absorption integral was plotted versus the relative concentration and linearly fitted. The ratio of the two slopes obtained is the ratio of the two molar extinction coefficients and was used to correct the absorption in the chromatogram of the crude reaction mixture.

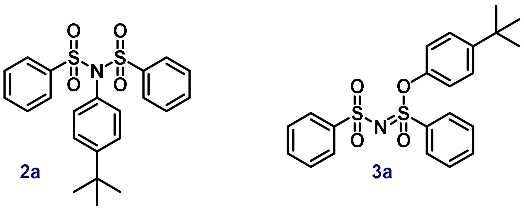

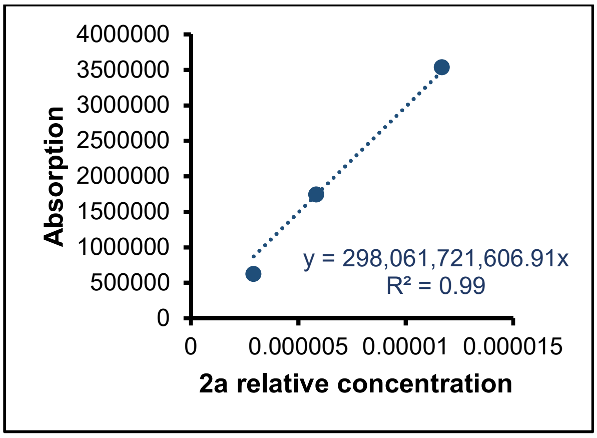

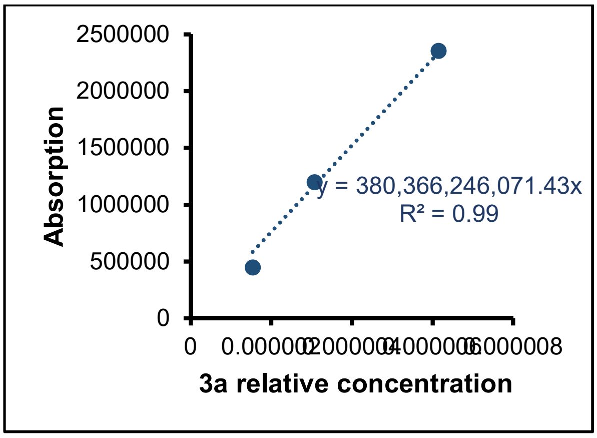

$$\varepsilon(3a) = 1.276\,\varepsilon(2a)$$

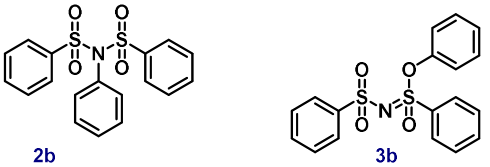

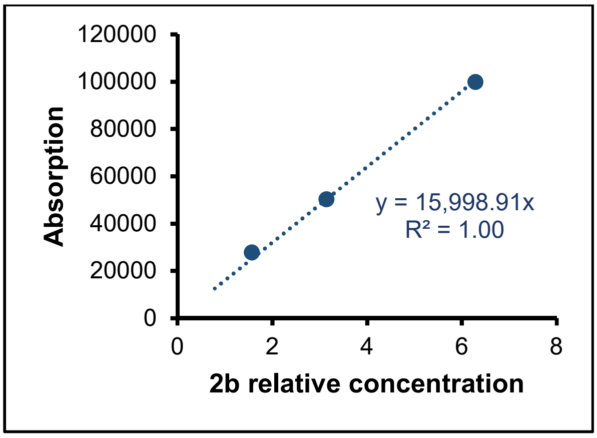

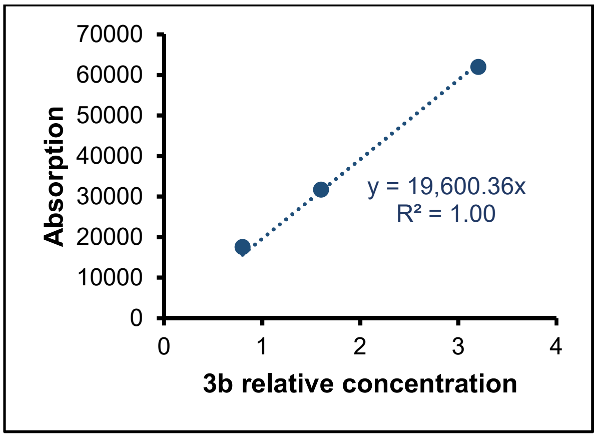

$$\varepsilon(3b) = 1.225\,\varepsilon(2b)$$

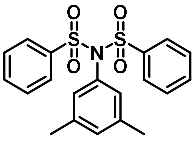

2c

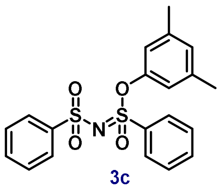

3c

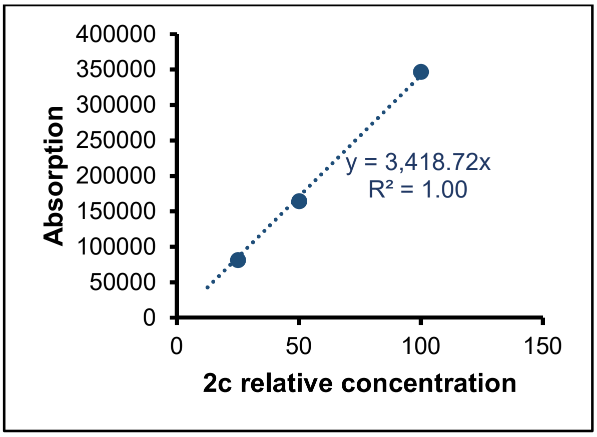

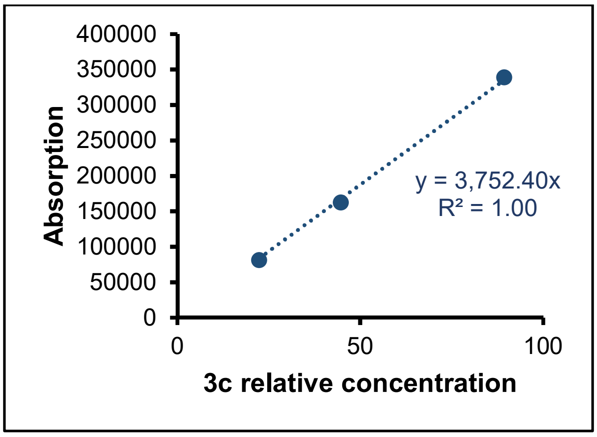

$$\varepsilon(3c) = 1.097\ \varepsilon(2c)$$

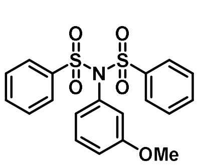

2d

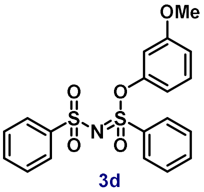

3d

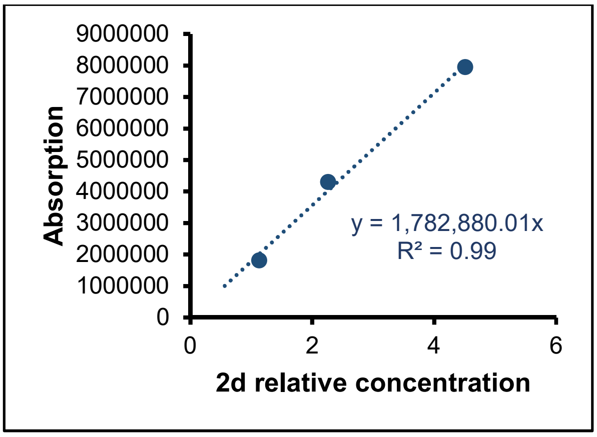

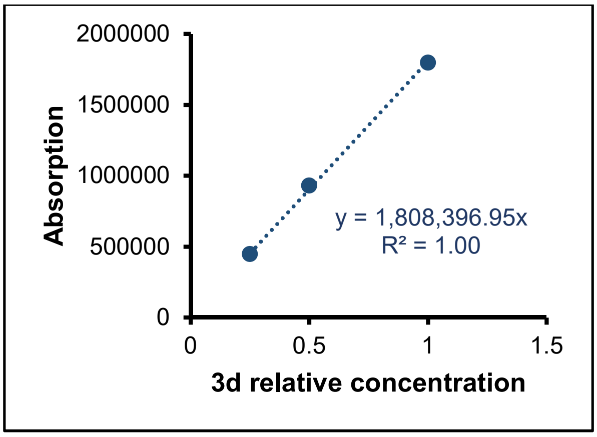

$$\varepsilon(3d) = 1.014\ \varepsilon(2d)$$

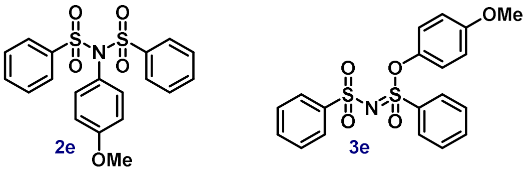

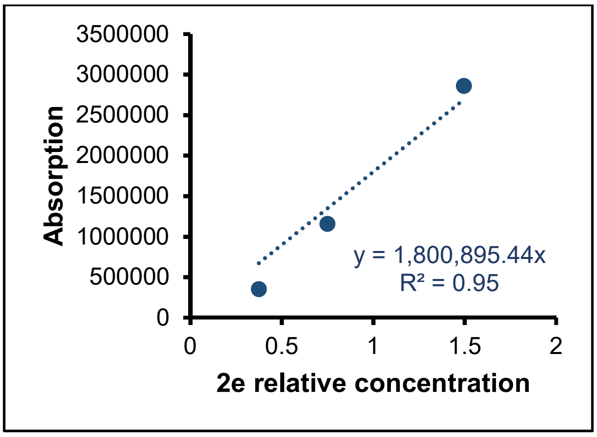

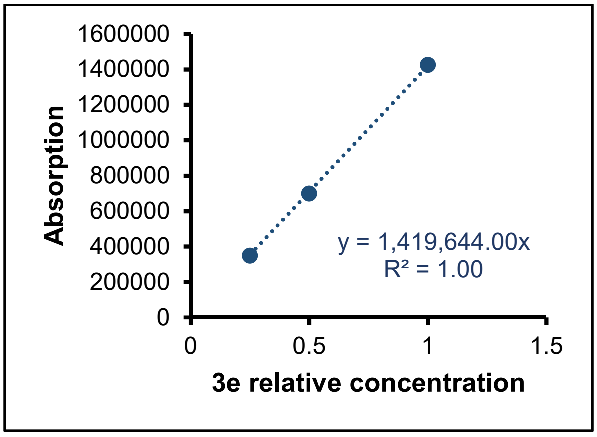

$$\varepsilon(3e) = 0.788\ \varepsilon(2e)$$

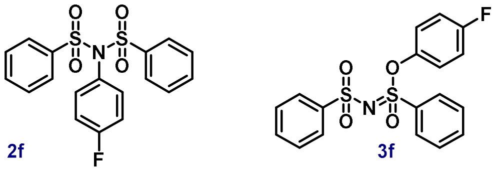

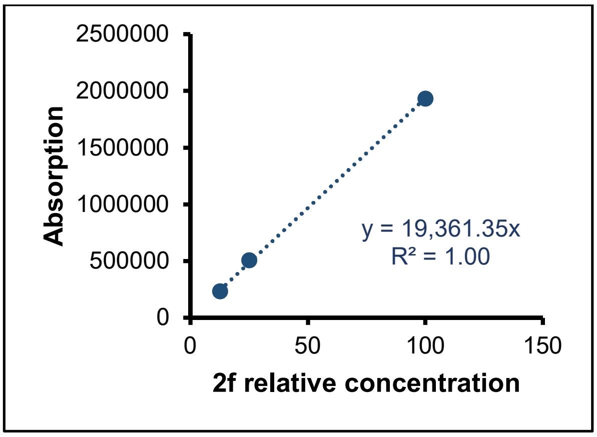

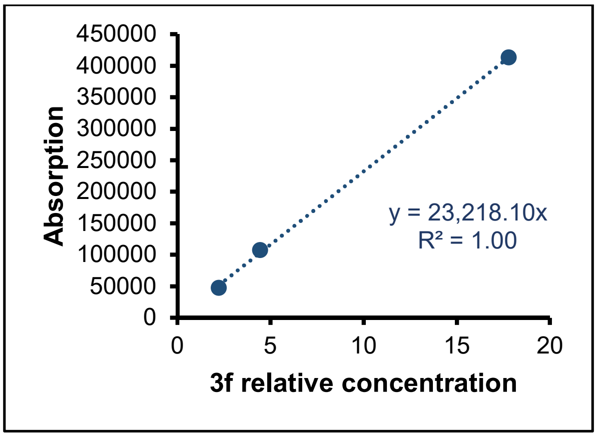

$$\varepsilon(3f) = 1.199\ \varepsilon(2f)$$

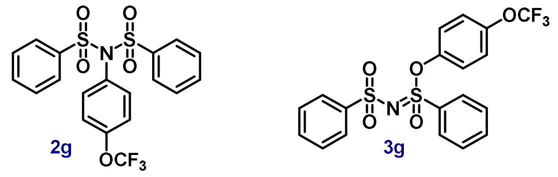

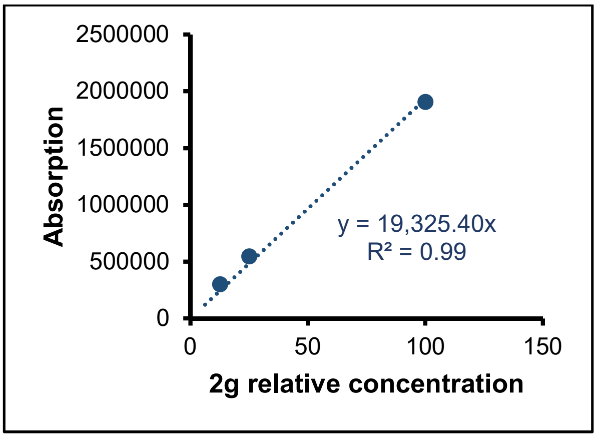

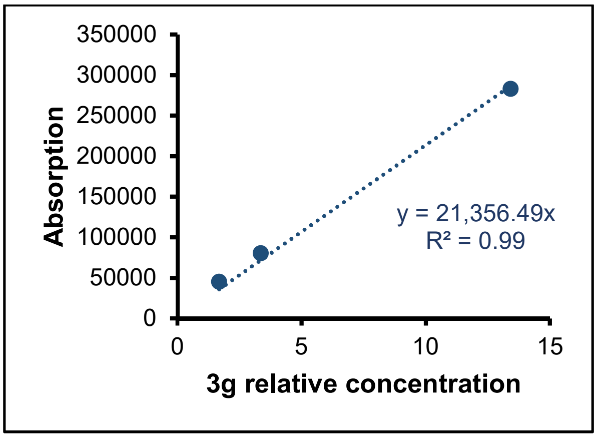

$$\varepsilon(3\mathrm{g}) = 1.105\ \varepsilon(2\mathrm{g})$$

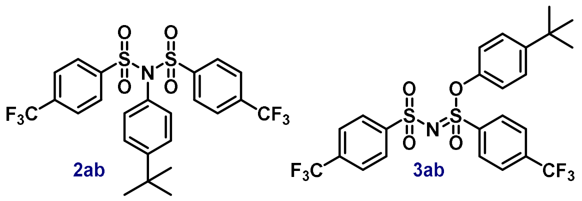

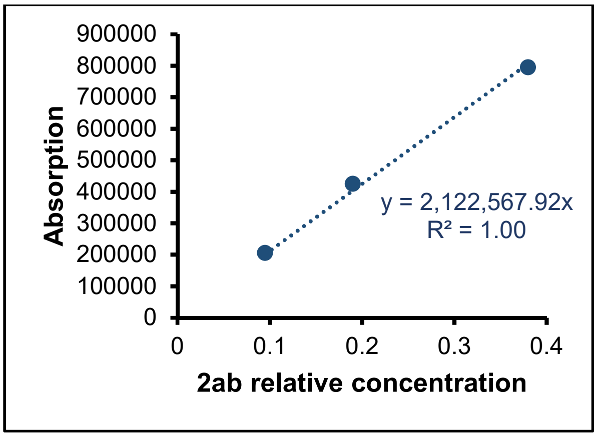

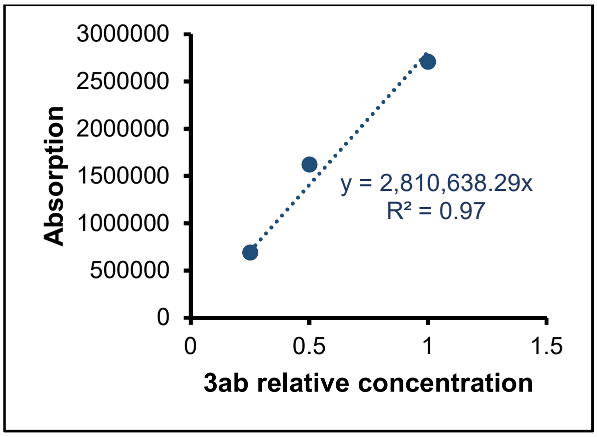

$$\varepsilon(3\mathrm{ab}) = 1.324\ \varepsilon(2\mathrm{ab})$$

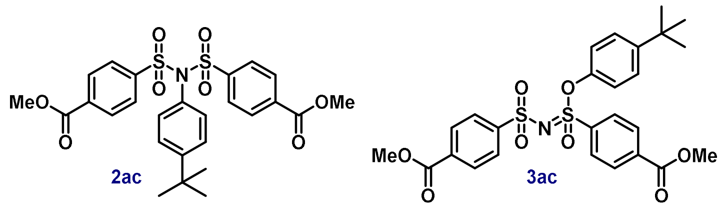

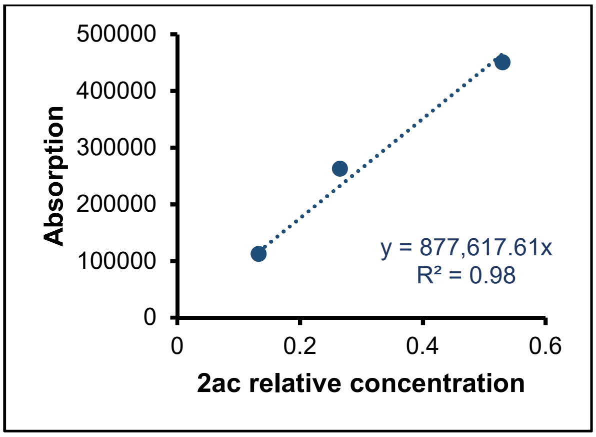

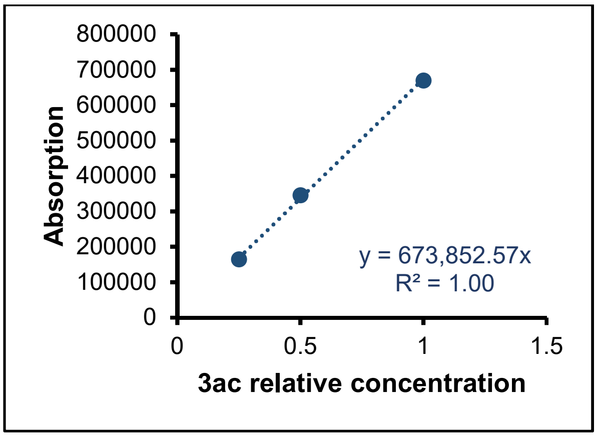

$$\varepsilon(3\text{ac}) = 0.880\ \varepsilon(2\text{ac})$$

4. Reaction optimization

General procedure for reaction optimization

A flame dried culture tube equipped with a Teflon-coated stir bar was charged with the appropriate bismuth catalyst (0.0075 mmol, 10 mol%), NFSI (47.3 mg, 0.15 mmol, 2.0 equiv.), (4-tertbutyl)phenyl boronic acid **4a** (13.4 mg, 0.075 mmol, 1.0 equiv.) and optional solid additives (*e.g.* inorganic base, stilbene). The culture tube was closed with a Teflon screw-cap, connected to an argon/vacuum Schlenk line and evacuated and refilled with argon (3 cycles). Anhydrous and deoxygenated appropriate solvent (0.75 mL, 0.1 M) was added through the septa followed by optional liquid additive (*e.g.*, tetraethylorthosilicate). The reaction vessel was then sealed using two pieces (*ca.* 2 cm) of electrical tape, the first one covering needle punctures and the second one around the cap thread. The reaction was stirred (600 rpm) for 16 h at the appropriate temperature in a pre-heated oil bath. After the reaction time, the crude solution was allowed to cool down to room temperature, concentrated in the culture tube at reduced pressure and dissolved in $CDCl_3$ with a known amount of CH_2Br_2 as an internal standard. Quantitative ^1H NMR was then recorded to determine the yield of the transformation, as well as HPLC following the aforementioned procedure to determine the selectivity of the reaction.

4.1. C–N selectivity, optimization

Table S1 Solvent optimization following the general procedure for optimization. Yields were determined by quantitative ^1H NMR using CH_2Br_2 as an internal standard. Selectivities were roughly estimated using quantitative ^1H NMR analysis and MestReNova line fitting feature.

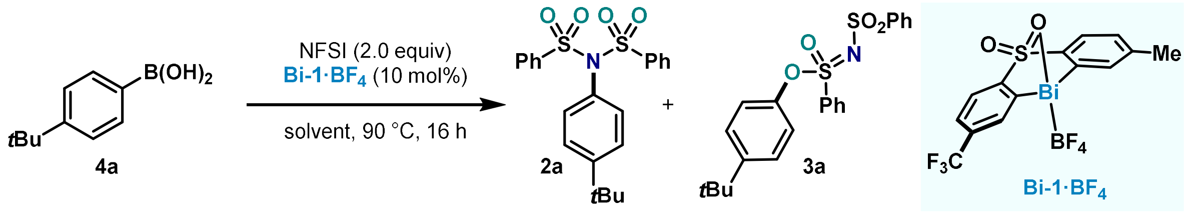

Entry	Solvent	Combined Yield	2a:3a
**1**	**$CDCl_3$**	**76%**	**~ 2:1**
2	PhCl	58%	~ 2:1
3	*o*-Cl_2Ph	47%	~ 2:1
4	DCE	39%	~ 2:1
5	*o*-F_2Ph	30%	~ 2:1
6	Toluene	50%	~ 2:1
7	THF[a]	Trace	nd
8	MTBE[a]	Trace	nd
9	Dioxane	25%	~ 2:1
10	DME	8%	~ 2:1
11	DMAc	Trace	nd
12	DMF	Trace	nd
13	DMSO	Trace	nd
**14**	**MeCN**	**35%**	**~ 1:2**
15	EtOAc[a]	Trace	nd
16	MeOH[a]	Trace	nd
17	EtOH	Trace	nd
18	*n*-BuOH	Trace	nd
19	HFIP	Trace	nd
20	$CHCl_3$	77%	~ 2:1

[a] Reaction performed at 70 °C

Table S2 Optimization toward selective C(sp²)–N coupling. Yields were determined by quantitative ^{1}H NMR using CH_2Br_2 as an internal standard. Selectivities were roughly estimated using quantitative ^{1}H NMR analysis and MestReNova line fitting feature or by HPLC using the protocol described in §3.

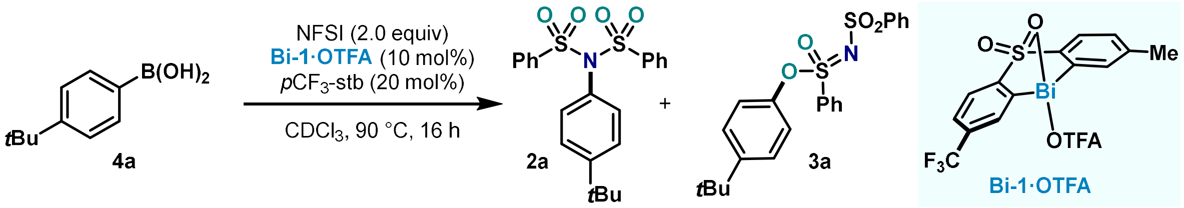

Entry	Deviations from above	Combined Yield	2a:3a
**1**	**None**	**79%**	**2.11:1**
2	Without Bi-1·OTFA	no product	-
3	NSIH instead of NFSI	no product	-
4	With H_2O (10 µL)	Trace	nd
5	At 25 °C	Trace	nd
6	At 60 °C	28%	~ 2:1
7	5 mol% of Bi-1·OTFA	14%	nd
8	without *p*CF_3-stb	63%	1.98:1
9	PhBpin or $PhBF_3K$ instead of **4a**	Trace	nd
10	With KF (1.0 equiv.)	25%	~ 2:1
11	With Na_2CO_3 (1.0 equiv.)	41%	~ 2:1
12	With KH_2PO_4 (1.0 equiv.)	63%	2.27:1
13	With PPh_3 (1.0 equiv.)	18%	~ 2:1
14	With DMAP (1.0 equiv.)	20%	~ 2:1
15	With NaNSI (1.0 equiv.)	59%	2.11:1
16	With TMSCl (20 mol%)	Trace	nd

Table S3 Catalyst optimization toward selective C(sp²)–N coupling. Yields were determined by quantitative ^{1}H NMR using CH_2Br_2 as an internal standard. Selectivities were determined by HPLC using the protocol described in §3.

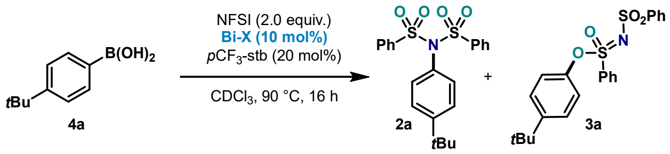

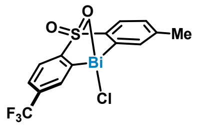

Bi-1·Cl
67% yield
3.00 **2a:3a**

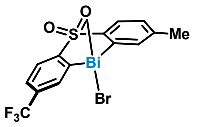

Bi-1·Br
81% yield
2.22 **2a:3a**

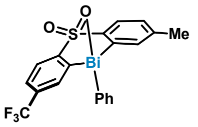

Bi-1·Ph
75% yield
2.48 **2a:3a**

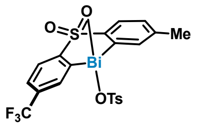

Bi-1·OTs
77% yield
1.97 **2a:3a**

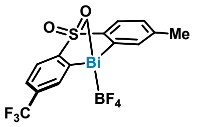

Bi-1·BF_4
90% yield
1.93 **2a:3a**

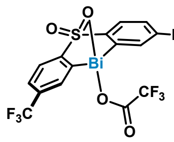

Bi-1·OTFA
79% yield
2.11 **2a:3a**

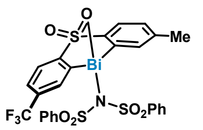

Bi-1·NSI
67% yield
2.35 **2a:3a**

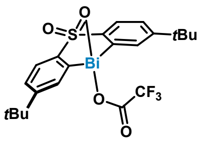

Bi-2·OTFA
62% yield
2.82 **2a:3a**

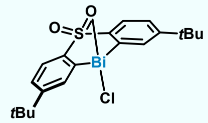

Bi-2·Cl
31% yield
3.65 **2a:3a**
@ 100°C, 84% yield, 3.42:1

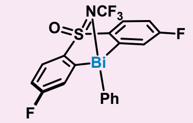

Bi-3·Ph
53% yield
0.76 **2a:3a**

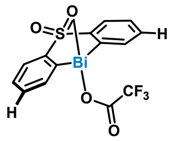

Bi-4·OTFA
77% yield
2.13 **2a:3a**

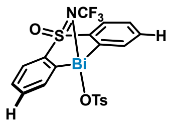

Bi-5·OTs
40% yield
1.03 **2a:3a**

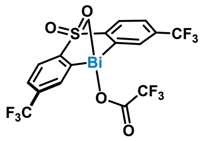

Bi-6·OTFA
69% yield
1.58 **2a:3a**

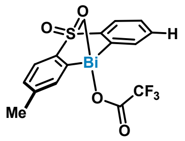

Bi-7·OTFA
49% yield
2.74 **2a:3a**

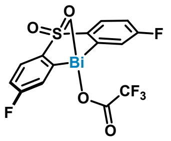

Bi-8·OTFA
67% yield
1.84 **2a:3a**

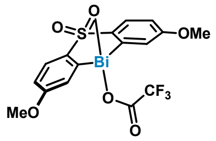

Bi-9·OTFA
61% yield
1.91 **2a:3a**

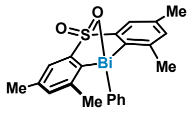

Bi-10·Ph
traces

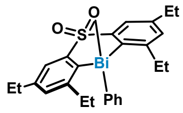

Bi-11·Ph
traces

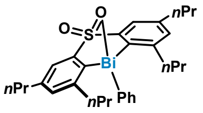

Bi-12·Ph
traces

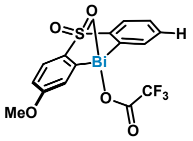

Bi-13·OTFA
59% yield
2.11 **2a:3a**

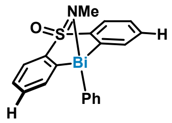

Bi-14·Ph
traces

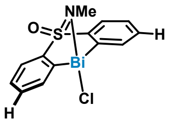

Bi-14·Cl
traces

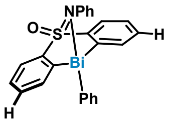

Bi-15·Ph
traces

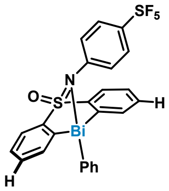

Bi-16·Ph
traces

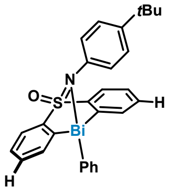

Bi-17·Ph
traces

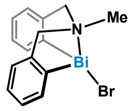

Bi-18
traces

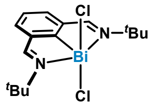

Bi-19
traces

4.2. C–O selectivity, optimization

Table S4 Solvent optimization toward selective C(sp²)–O coupling. Yields were determined by quantitative ^{1}H NMR using CH_2Br_2 as an internal standard. Selectivities were determined by HPLC using the protocol described in §3.

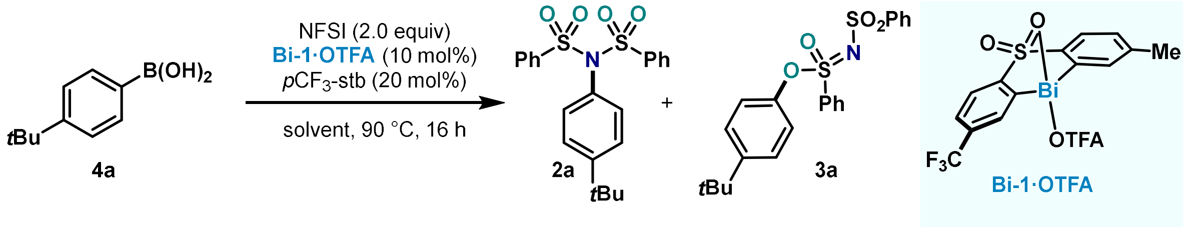

Entry	Solvent	Combined Yield	3a:2a
1	$CDCl_3$	75%	0.49
**2**	**MeCN**	**35%**	**2.23**
3	$CDCl_3$:MeCN 1:1	25%	0.47
4	$CDCl_3$:MeCN 7:1	22%	0.38
5	$CDCl_3$:MeCN 1:7	36%	2.28
6	$MeNO_2$	12%	0.70
7	*t*BuCN	13%	0.43
8	PhCN	trace	nd
9	MeCN-*d*$_3$	36%	2.24

Table S5 Catalyst optimization toward selective C(sp²)–O coupling. Yields were determined by quantitative ^{1}H NMR using CH_2Br_2 as an internal standard. Selectivities were determined by HPLC using the protocol described in §3.

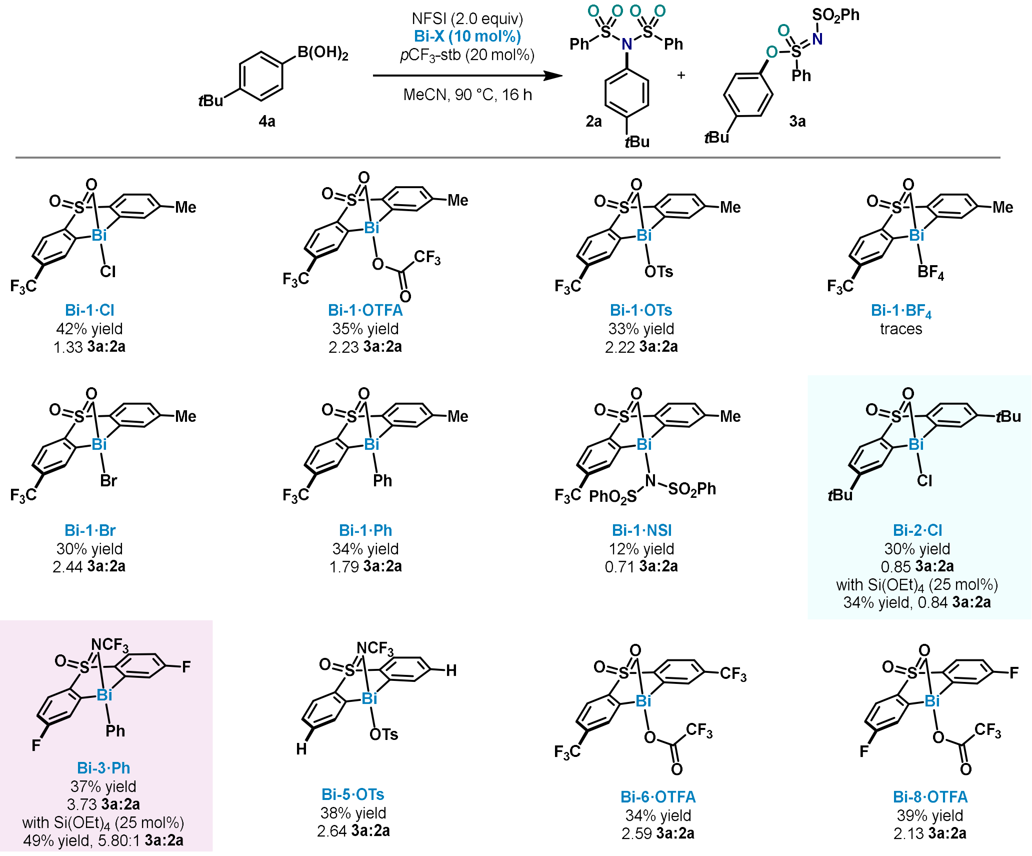

5. Reaction scope

General procedure for Condition A (C(sp²)–N selective)

A flame dried culture tube equipped with a Teflon-coated stir bar was charged with 2,8-di-tert-butyl-10-chloro-10H-dibenzo[b,e][1,4]thiabismine 5,5-dioxide **Bi-2·Cl** (4.3 mg, 0.0075 mmol, 10 mol%), *N*-fluorosulfonimide derivative **1** (0.15 mmol, 2.0 equiv.), arylboronic acid **4** (0.075 mmol, 1.0 equiv.) and (*E*)-1,2-bis(4-(trifluoromethyl)phenyl)ethene (4.7 mg, 0.015 mmol, 20 mol%). The culture tube was closed with a Teflon screw-cap, connected to an argon/vacuum Schlenk line and evacuated and refilled with argon (3 cycles). Anhydrous and degassed $CDCl_3$ (0.75 mL, 0.1 M) was added through the septa. The reaction vessel was then sealed using two pieces (*ca.* 2 cm) of electrical tape, the first one covering needle punctures and the second one around the cap thread. The reaction was stirred (600 rpm) for 24 h at 100 °C in a pre-heated oil bath. After the reaction time, the crude solution was allowed to cool down to room temperature, concentrated in the culture tube at reduced pressure and dissolved in $CDCl_3$ with a known amount of CH_2Br_2 as an internal standard. Quantitative ^{1}H NMR was then recorded to determine the yield of the transformation, as well as HPLC following the aforementioned procedure to determine the selectivity of the reaction. Preparative TLC was then used to obtain the pure C(sp²)–N product **2**, using typically a pentane/EtOAc (85:15) or pentane/CH_2Cl_2 (70:30) eluent.

General procedure for Condition B (C(sp²)–O selective)

A flame dried culture tube equipped with a Teflon-coated stir bar was charged with 2,8-difluoro-10-phenyl-5-((trifluoromethyl)imino)-5,10-dihydro-5l4-dibenzo[b,e][1,4]thiabismine 5-oxide **Bi-3·Ph** (4.5 mg, 0.0075 mmol, 10 mol%), *N*-fluorosulfonimide derivative **1** (0.15 mmol, 2.0 equiv.), arylboronic acid **4** (0.075 mmol, 1.0 equiv.) and (*E*)-1,2-bis(4-(trifluoromethyl)phenyl)ethene (4.7 mg, 0.015 mmol, 20 mol%). The culture tube was closed with a Teflon screw-cap, connected to an argon/vacuum Schlenk line and evacuated and refilled with argon (3 cycles). Anhydrous and degassed MeCN (0.75 mL, 0.1 M) was added through the septa, followed by tetraethylorthosilicate using a microliter syringe (4.2 μL, 0.019 mmol, 25 mol%). The reaction vessel was then sealed using two pieces (*ca.* 2 cm) of electrical tape, the first one covering needle punctures and the second one around the cap thread. The reaction was stirred (600 rpm) for 24 h at 90 °C in a pre-heated oil bath. After the reaction time, the crude solution was allowed to cool down to room temperature, concentrated in the culture tube at reduced pressure and dissolved in $CDCl_3$ with a known amount of CH_2Br_2 as an internal standard. Quantitative ^{1}H NMR was then recorded to determine the yield of the transformation, as well as HPLC following the aforementioned procedure to determine the selectivity of the reaction. Preparative TLC was then used to obtain the pure C(sp²)–O product **3**, using typically a pentane/EtOAc (75:25) or pentane/CH_2Cl_2 (50:50) eluent.

5.1. Product characterization

N-(4-(tert-butyl)phenyl)-N-(phenylsulfonyl)benzenesulfonamide (2a)

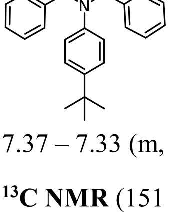

Following General procedure for condition A, starting from 13.4 mg of 4-tertbutylphenylboronic acid (0.075 mmol), compound **2a** (20.2 mg, 63% yield) was isolated as a white solid.

^{1}H NMR (600 MHz, $CDCl_3$) δ 7.97 – 7.94 (m, 4H), 7.69 – 7.65 (m, 2H), 7.57 – 7.53 (m, 4H), 7.37 – 7.33 (m, 2H), 6.95 – 6.92 (m, 2H), 1.32 (s, 9H).

^{13}C NMR (151 MHz, $CDCl_3$) δ 153.7, 139.8, 134.0, 131.5, 131.0, 129.1, 128.7, 126.4, 35.0, 31.4.

HRMS (EI-Orbitrap) calc'd for $C_{22}H_{23}NO_4S_2$ $[M]^{•+}$: 429.10630, found: 429.10643.

HPLC Zorbax Eclipse Plus, C18 1.8 μm, 4.6 x 50 mm; gradient 70% to 90% (5 min) MeOH/H_2O, 1.0 mL/min, 220 nm; t_R = 3.54 min.

R_f (pentane:EtOAc = 85:15) 0.58

4-(tert-butyl)phenyl N-(phenylsulfonyl)benzenesulfonimidate (3a)

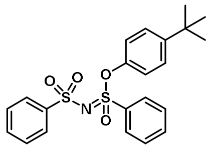

Following General procedure for condition B, starting from 13.4 mg of (4-tertbutylphenyl)boronic acid (0.075 mmol), compound **3a** (13.0 mg, 40% yield) was isolated as a white solid.

^{1}H NMR (600 MHz, $CDCl_3$) δ 8.00 – 7.97 (m, 2H), 7.95 – 7.93 (m, 2H), 7.72 – 7.67 (m, 1H), 7.57 – 7.51 (m, 3H), 7.47 – 7.43 (m, 2H), 7.29 – 7.25 (m, 2H), 6.94 – 6.89 (m, 2H), 1.27 (s, 9H).

^{13}C NMR (151 MHz, $CDCl_3$) δ 151.1, 146.8, 143.0, 135.7, 135.0, 132.6, 129.4, 128.8, 128.4, 127.0, 126.8, 34.8, 31.4.

HRMS (EI-Orbitrap) calc'd for $C_{22}H_{23}NO_4S_2$ $[M]^{\bullet+}$: 429.10630, found: 429.10623.

HPLC Zorbax Eclipse Plus, C18 1.8 µm, 4.6 x 50 mm; gradient 70% to 90% (5 min) MeOH/H_2O, 1.0 mL/min, 220 nm; t_R = 3.21 min.

R_f (pentane:EtOAc = 85:15) 0.21

N-phenyl-N-(phenylsulfonyl)benzenesulfonamide (2b)

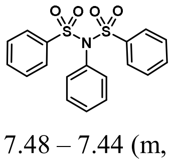

Following General Procedure for condition A, starting from 9.1 mg of phenylboronic acid (0.075 mmol), compound **2b** (16.1 mg, 58% yield) was isolated as a white solid.

^{1}H NMR (600 MHz, $CDCl_3$) δ 7.97 – 7.93 (m, 4H), 7.71 – 7.66 (m, 2H), 7.58 – 7.53 (m, 4H), 7.48 – 7.44 (m, 1H), 7.38 – 7.34 (m, 2H), 7.05 – 7.01 (m, 2H).

^{13}C NMR (151 MHz, $CDCl_3$) δ 139.6, 134.4, 134.1, 131.7, 130.5, 129.4, 129.1, 128.7.

HRMS (EI-Orbitrap) calc'd for $C_{18}H_{15}NO_4S_2$ $[M]^{\bullet+}$: 373.04370, found: 373.04398.

HPLC Zorbax Eclipse Plus, C18 1.8 µm, 4.6 x 50 mm; gradient 70% to 90% (5 min) MeOH/H_2O, 1.0 mL/min, 220 nm; t_R = 1.34 min.

R_f (pentane:EtOAc = 85:15) 0.38

phenyl N-(phenylsulfonyl)benzenesulfonimidate (3b)

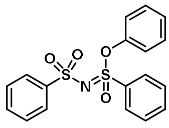

Following General procedure for condition B, starting from 9.1 mg of phenylboronic acid (0.075 mmol), compound **3b** (9.9 mg, 35% yield) was isolated as a light yellow solid.

^{1}H NMR (600 MHz, $CDCl_3$) δ 8.01 – 7.98 (m, 2H), 7.95 – 7.92 (m, 2H), 7.72 – 7.68 (m, 1H), 7.57 – 7.52 (m, 3H), 7.48 – 7.44 (m, 2H), 7.31 – 7.25 (m, 3H), 7.04 – 6.99 (m, 2H).

^{13}C NMR (151 MHz, $CDCl_3$) δ 149.1, 143.0, 135.5, 135.1, 132.6, 130.0, 129.5, 128.9, 128.4, 128.0, 127.0, 122.9.

HRMS (EI-Orbitrap) calc'd for $C_{18}H_{15}NO_4S_2$ $[M]^{\bullet+}$: 373.04370, found: 373.04394.

HPLC Zorbax Eclipse Plus, C18 1.8 µm, 4.6 x 50 mm; gradient 70% to 90% (5 min) MeOH/H_2O, 1.0 mL/min, 220 nm; t_R = 1.66 min.

R_f (pentane:EtOAc = 85:15) 0.12

N-(3,5-dimethylphenyl)-N-(phenylsulfonyl)benzenesulfonamide (2c)

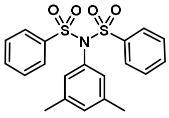

Following General procedure for condition A, starting from 11.2 mg of (3,5-dimethylphenyl)boronic acid (0.075 mmol), compound **2c** (17.9 mg, 59% yield) was isolated as a white solid.

^{1}H NMR (600 MHz, $CDCl_3$) δ 8.00 – 7.91 (m, 4H), 7.70 – 7.65 (m, 2H), 7.59 – 7.52 (m, 4H), 7.07 (dq, J = 1.6, 0.8 Hz, 1H), 6.61 (dt, J = 1.5, 0.7 Hz, 2H), 2.25 (q, J = 0.7 Hz, 6H).

^{13}C NMR (151 MHz, $CDCl_3$) δ 139.7, 139.1, 134.01, 133.97, 132.3, 129.2, 129.0, 128.8, 21.2.

HRMS (ESI-Orbitrap) calc'd for $C_{20}H_{19}NO_4S_2$ $[M+NA]^+$: 424.06477, found: 424.06516

HPLC Zorbax Eclipse Plus, C18 1.8 μm, 4.6 x 50 mm; gradient 70% to 90% (5 min) MeOH/H_2O, 1.0 mL/min, 220 nm; t_R = 2.38 min.

R_f (pentane:EtOAc = 85:15) 0.38

3,5-dimethylphenyl N-(phenylsulfonyl)benzenesulfonimidate (3c)

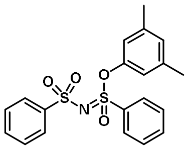

Following General procedure for condition B, starting from 11.2 mg of (3,5-dimethylphenyl)boronic acid (0.075 mmol), compound **3c** (9.8 mg, 33% yield) was isolated as a white solid.

^{1}H NMR (600 MHz, $CDCl_3$) δ 7.96 (ddt, J = 15.2, 7.7, 1.2 Hz, 4H), 7.73 – 7.67 (m, 1H), 7.59 – 7.50 (m, 3H), 7.48 – 7.41 (m, 2H), 6.87 (tt, J = 1.5, 0.8 Hz, 1H), 6.60 (dt, J = 1.6, 0.7 Hz, 2H), 2.21 (q, J = 0.7 Hz, 6H).

^{13}C NMR (151 MHz, $CDCl_3$) δ 148.9, 143.1, 139.9, 135.8, 135.0, 132.5, 129.6, 129.4, 128.8, 128.4, 127.0, 120.2, 21.3.

HRMS (ESI-Orbitrap) calc'd for $C_{20}H_{19}NO_4S_2$ $[M+NA]^+$: 424.06477, found: 424.06499

HPLC Zorbax Eclipse Plus, C18 1.8 μm, 4.6 x 50 mm; gradient 70% to 90% (5 min) MeOH/H_2O, 1.0 mL/min, 220 nm; t_R = 2.15 min.

R_f (pentane:EtOAc = 85:15) 0.15

N-(3-methoxyphenyl)-N-(phenylsulfonyl)benzenesulfonamide (2d)

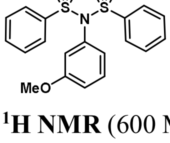

Following General procedure for condition A, starting from 11.4 mg of (3-methoxyphenyl)boronic acid (0.075 mmol), compound **2d** (17.4 mg, 57% yield) was isolated as a white solid.

^{1}H NMR (600 MHz, $CDCl_3$) δ 8.00 – 7.94 (m, 4H), 7.71 – 7.65 (m, 2H), 7.59 – 7.52 (m, 4H), 7.28 – 7.23 (m, 1H), 6.99 (ddd, J = 8.4, 2.5, 0.9 Hz, 1H), 6.62 (ddd, J = 7.8, 2.0, 0.9 Hz, 1H), 6.52 – 6.50 (m, 1H), 3.69 (s, 3H).

^{13}C NMR (151 MHz, $CDCl_3$) δ 160.1, 139.6, 135.2, 134.1, 129.8, 129.1, 128.8, 123.9, 117.0, 116.6, 55.5.

HRMS (EI-Orbitrap) calc'd for $C_{19}H_{17}NO_5S_2$ $[M]^{\bullet+}$: 403.05427, found: 403.05420.

HPLC Zorbax Eclipse Plus, C18 1.8 μm, 4.6 x 50 mm; gradient 70% to 90% (5 min) MeOH/H_2O, 1.0 mL/min, 220 nm; t_R = 1.76 min.

R_f (pentane:EtOAc = 85:15) 0.33

3-methoxyphenyl N-(phenylsulfonyl)benzenesulfonimidate (3d)

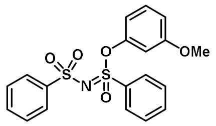

Following General procedure for condition B, starting from 11.4 mg of (3-methoxyphenyl)boronic acid (0.075 mmol), compound **3d** (10.9 mg, 36% yield) was isolated as a light yellow oil.

^{1}H NMR (600 MHz, $CDCl_3$) δ 8.03 – 7.98 (m, 2H), 7.95 – 7.92 (m, 2H), 7.73 – 7.67 (m, 1H), 7.58 – 7.51 (m, 3H), 7.48 – 7.44 (m, 2H), 7.14 (t, J = 8.26 Hz, 1H), 6.80 (ddd, J = 8.38, 2.46, 0.88 Hz, 1H), 6.63 (t, J = 2.34 Hz, 1H), 6.52 (ddd, J = 8.15, 2.26, 0.88 Hz, 1H), 3.72 (s, 3H).

^{13}C NMR (151 MHz, $CDCl_3$) δ 160.7, 149.9, 143.0, 135.4, 135.1, 132.6, 130.1, 129.5, 128.9, 128.4, 127.0, 114.6, 114.3, 108.5, 55.7.

HRMS (ESI-Orbitrap) calc'd for $C_{19}H_{17}NNaO_5S_2$ $[M+Na]^+$: 426.04404, found: 426.04399.

HPLC Zorbax Eclipse Plus, C18 1.8 μm, 4.6 x 50 mm; gradient 70% to 90% (5 min) MeOH/H_2O, 1.0 mL/min, 220 nm; t_R = 1.52 min.

R_f (pentane:EtOAc = 85:15) 0.13

N-(4-methoxyphenyl)-N-(phenylsulfonyl)benzenesulfonamide (2e)

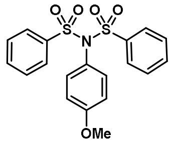

Following General procedure for condition A, starting from 11.4 mg of (4-methoxyphenyl)boronic acid (0.075 mmol), compound **2e** (8.2 mg, 27% yield) was isolated as a white solid.

^{1}H NMR (600 MHz, $CDCl_3$) δ 7.96 – 7.91 (m, 4H), 7.70 – 7.65 (m, 2H), 7.58 – 7.53 (m, 4H), 6.96 – 6.90 (m, 2H), 6.85 – 6.81 (m, 2H), 3.82 (s, 3H).

^{13}C NMR (151 MHz, $CDCl_3$) δ 161.1, 139.7, 134.0, 132.9, 129.1, 128.7, 126.6, 114.6, 55.6.

HRMS (EI-Orbitrap) calc'd for $C_{19}H_{17}NO_5S_2$ $[M]^{\bullet+}$: 403.05427, found: 403.05456.

HPLC Zorbax Eclipse Plus, C18 1.8 μm, 4.6 x 50 mm; gradient 70% to 90% (5 min) MeOH/H_2O, 1.0 mL/min, 220 nm; t_R = 1.80 min.

R_f (pentane:EtOAc = 85:15) 0.11

4-methoxyphenyl N-(phenylsulfonyl)benzenesulfonimidate (3e)

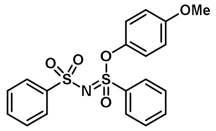

Following General procedure for condition B, starting from 11.4 mg of (4-methoxyphenyl)boronic acid (0.075 mmol), compound **3e** (4.2 mg, 14% yield) was isolated as an orange oil.

^{1}H NMR (600 MHz, $CDCl_3$) δ 8.01 – 7.97 (m, 2H), 7.93 – 7.90 (m, 2H), 7.72 – 7.68 (m, 1H), 7.55 – 7.51 (m, 3H), 7.49 – 7.43 (m, 2H), 6.96 – 6.89 (m, 2H), 6.78 – 6.73 (m, 2H), 3.77 (s, 3H).

^{13}C NMR (151 MHz, $CDCl_3$) δ 158.9, 143.0, 142.4, 135.3, 135.0, 132.6, 129.4, 128.8, 128.5, 127.0, 123.9, 114.8, 55.7.

HRMS (ESI-Orbitrap) calc'd for $C_{19}H_{17}NNaO_5S_2$ $[M+Na]^+$: 426.04404, found: 426.04407.

HPLC Zorbax Eclipse Plus, C18 1.8 μm, 4.6 x 50 mm; gradient 70% to 90% (5 min) MeOH/H_2O, 1.0 mL/min, 220 nm; t_R = 1.49 min.

R_f (pentane:EtOAc = 85:15) 0.30

N-(4-fluorophenyl)-N-(phenylsulfonyl)benzenesulfonamide (2f)

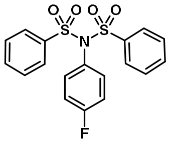

Following General procedure for condition A, starting from 10.5 mg of (4-fluorophenyl)boronic acid (0.075 mmol), compound **2f** (19.8 mg, 67% yield) was isolated as a white solid.

^{1}H NMR (600 MHz, $CDCl_3$) δ 8.00 – 7.89 (m, 4H), 7.72 – 7.66 (m, 2H), 7.60 – 7.53 (m, 4H), 7.06 – 7.02 (m, 2H), 7.01 – 6.98 (m, 2H).

^{19}F NMR (282 MHz, $CDCl_3$) δ -109.38.

^{13}C NMR (151 MHz, $CDCl_3$) δ 163.7 (d, J = 251.8 Hz), 139.4, 134.2, 133.6 (d, J = 9.2 Hz), 130.2 (d, J = 3.3 Hz), 129.2, 128.7, 116.5 (d, J = 23.1 Hz).

HRMS (EI-Orbitrap) calc'd for $C_{18}H_{14}NO_4S_2F$ $[M]^{\bullet+}$: 391.03485, found: 391.03428.

HPLC Zorbax Eclipse Plus, C18 1.8 μm, 4.6 x 50 mm; gradient 70% to 90% (5 min) MeOH/H_2O, 1.0 mL/min, 220 nm; t_R = 1.87 min.

R$_f$ (pentane:EtOAc = 85:15) 0.48

4-fluorophenyl N-(phenylsulfonyl)benzenesulfonimidate (3f)

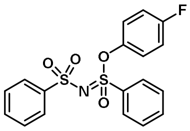

Following General procedure for condition A, starting from 10.5 mg of (4-fluorophenyl)boronic acid (0.075 mmol), compound **3f** (2.7 mg, 9% yield) was isolated as a white solid.

^{1}H NMR (600 MHz, $CDCl_3$) δ 8.01 – 7.97 (m, 2H), 7.95 – 7.91 (m, 2H), 7.74 – 7.70 (m, 1H), 7.60 – 7.52 (m, 3H), 7.49 – 7.45 (m, 2H), 7.02 – 6.99 (m, 2H), 6.98 – 6.94 (m, 2H).

^{19}F NMR (282 MHz, $CDCl_3$) δ -113.26.

^{13}C NMR (151 MHz, $CDCl_3$) δ 161.6 (d, J = 247.9 Hz), 144.8 (d, J = 2.9 Hz), 142.8, 135.3, 135.1, 132.8, 129.6, 128.9, 128.4, 127.0, 124.6 (d, J = 8.8 Hz), 116.7 (d, J = 23.8 Hz).

HRMS (EI-Orbitrap) calc'd for $C_{18}H_{14}NO_4S_2F$ $[M]^{\bullet+}$: 391.03428, found: 391.03451.

HPLC Zorbax Eclipse Plus, C18 1.8 μm, 4.6 x 50 mm; gradient 70% to 90% (5 min) MeOH/H_2O, 1.0 mL/min,

220 nm; t_R = 1.44 min.

R$_f$ (pentane:EtOAc = 85:15) 0.18

N-(phenylsulfonyl)-N-(4-(trifluoromethoxy)phenyl)benzenesulfonamide (2g)

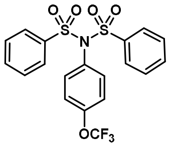

Following General procedure for condition A, starting from 15.4 mg of (4-(trifluoromethoxy)phenyl)boronic acid (0.075 mmol), compound **2g** (12.6 mg, 37% yield) was isolated as a white solid.

^{1}H NMR (600 MHz, $CDCl_3$) δ 7.96 – 7.91 (m, 4H), 7.73 – 7.67 (m, 2H), 7.60 – 7.55 (m, 4H), 7.20 – 7.16 (m, 2H), 7.07 – 7.03 (m, 2H).

^{19}F NMR (282 MHz, $CDCl_3$) δ -57.78.

^{13}C NMR (151 MHz, $CDCl_3$) δ 150.5 (q, J = 2.2 Hz), 139.3, 134.3, 133.3, 132.6, 129.3, 128.7, 121.4, 120.4 (q, J = 258.7 Hz).

HRMS (EI-Orbitrap) calc'd for $C_{19}H_{14}NO_5S_2F_3$ $[M]^{\bullet+}$: 457.02640, found: 457.02600.

HPLC Zorbax Eclipse Plus, C18 1.8 μm, 4.6 x 50 mm; gradient 70% to 90% (5 min) MeOH/H_2O, 1.0 mL/min, 220 nm; t_R = 2.80 min.

R_f (pentane:EtOAc = 85:15) 0.60

4-(trifluoromethoxy)phenyl N-(phenylsulfonyl)benzenesulfonimidate (3g)

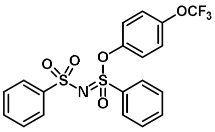

Following General procedure for condition A, starting from 15.4 mg of (4-(trifluoromethoxy)phenyl)boronic acid (0.075 mmol), compound **3g** (2.4 mg, 7% yield) was isolated as a light yellow oil.

^{1}H NMR (600 MHz, $CDCl_3$) δ 8.00 – 7.97 (m, 2H), 7.96 – 7.93 (m, 2H), 7.73 (ddt, *J* = 8.7, 7.3, 1.2 Hz, 1H), 7.60 – 7.56 (m, 2H), 7.55 – 7.52 (m, 1H), 7.50 – 7.44 (m, 2H), 7.16 – 7.12 (m, 2H), 7.09 – 7.05 (m, 2H).

^{19}F NMR (282 MHz, $CDCl_3$) δ -58.11.

^{13}C NMR (151 MHz, $CDCl_3$) δ 148.2 (q, *J* = 1.8 Hz), 147.0, 142.7, 135.4, 135.1, 132.8, 129.6, 128.9, 128.4, 127.0, 124.4, 122.4, 120.4 (q, *J* = 258.3 Hz).

HRMS (EI-Orbitrap) calc'd for $C_{19}H_{14}NO_5S_2F_3$ $[M]^{\bullet+}$: 457.02640, found: 457.02601.

HPLC Zorbax Eclipse Plus, C18 1.8 μm, 4.6 x 50 mm; gradient 70% to 90% (5 min) MeOH/H_2O, 1.0 mL/min, 220 nm; t_R = 2.23 min.

R_f (pentane:EtOAc = 85:15) 0.21

N-(phenylsulfonyl)-N-(4-(trifluoromethyl)phenyl)benzenesulfonamide (2h)

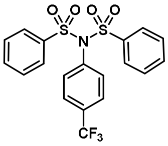

Following General procedure for condition A, starting from 14.2 mg of (4-(trifluoromethyl)phenyl)boronic acid (0.075 mmol), compound **2h** (15.7 mg, 47% yield) was isolated as a white solid.

^{1}H NMR (600 MHz, $CDCl_3$) δ 7.97 – 7.91 (m, 4H), 7.77 – 7.67 (m, 2H), 7.65 – 7.62 (m, 2H), 7.61 – 7.55 (m, 4H), 7.19 – 7.13 (m, 2H).

^{19}F NMR (282 MHz, $CDCl_3$) δ -62.85.

^{13}C NMR (151 MHz, $CDCl_3$) δ 139.3, 137.6 (q, *J* = 1.5 Hz), 134.4, 132.4 (q, *J* = 32.2 Hz), 132.2, 129.3, 128.7, 126.5 (q, *J* = 3.7 Hz), 123.6 (q, *J* = 272.7 Hz).

HRMS (EI-Orbitrap) calc'd for $C_{19}H_{14}NO_4S_2F_3$ $[M]^{\bullet+}$: 441.03147, found: 441.03109.

R_f (pentane:EtOAc = 85:15) 0.55

N-(4-(tert-butyl)phenyl)-4-(trifluoromethyl)-N-((4-(trifluoromethyl)phenyl)sulfonyl)benzenesulfonamide (2ab)

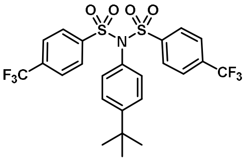

Following General procedure for condition A, starting from 13.4 mg of 4-tertbutylphenylboronic acid (0.075 mmol) and 67.7 mg of N-fluoro-4-(trifluoromethyl)-N-((4-(trifluoromethyl)phenyl)sulfonyl)benzenesulfonamide (0.150 mmol), compound **2ab** (19.4 mg, 46% yield) was isolated as a white solid.

^{1}H NMR (600 MHz, $CDCl_3$) δ 8.17 – 8.04 (m, 1H), 7.87 – 7.79 (m, 4H), 7.44 – 7.38 (m, 2H), 6.96 – 6.91 (m, 2H), 1.34 (s, 9H).

^{19}F NMR (565 MHz, $CDCl_3$) δ -63.23.

^{13}**C NMR** (151 MHz, $CDCl_3$) δ 154.5, 142.9, 135.8 (q, *J* = 33.2 Hz), 130.9, 130.8, 129.3, 126.8, 126.4 (q, *J* = 3.7 Hz), 123.2 (q, *J* = 273.3 Hz), 35.1, 31.4.

HRMS (ESI-Orbitrap) calc'd for $C_{24}H_{21}NO_4S_2F_6Na$ $[M+Na]^+$: 588.0708, found: 588.0711.

HPLC Zorbax Eclipse Plus, C18 1.8 µm, 4.6 x 50 mm; gradient 70% to 90% (5 min) MeOH/H_2O, 1.0 mL/min, 220 nm; t_R = 5.52 min.

R_f (pentane:EtOAc = 90:10) 0.73

4-(tert-butyl)phenyl 4-(trifluoromethyl)-N-((4-(trifluoromethyl)phenyl)sulfonyl)benzenesulfonimidate (3ab)

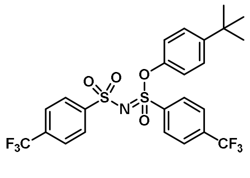

Following General procedure for condition B, starting from 13.4 mg of 4-tertbutylphenylboronic acid (0.075 mmol) and 67.7 mg of N-fluoro-4-(trifluoromethyl)-N-((4-(trifluoromethyl)phenyl)sulfonyl)benzenesulfonamide (0.150 mmol), compound **3ab** (10.9 mg, 26% yield) was isolated as a light yellow oil.

^{1}**H NMR** (600 MHz, $CDCl_3$) δ 8.13 – 8.09 (m, 2H), 8.09 – 8.05 (m, 2H), 7.87 – 7.82 (m, 2H), 7.73 – 7.67 (m, 2H), 7.34 – 7.31 (m, 2H), 6.97 – 6.93 (m, 2H), 1.29 (s, 9H).

^{13}**C NMR** (151 MHz, $CDCl_3$) δ 151.8, 146.3, 146.0, 139.2, 136.8 (q, *J* = 33.5 Hz), 134.5 (q, *J* = 33.0 Hz), 129.0, 127.6, 127.2, 126.7 (q, *J* = 3.7 Hz), 126.1 (q, *J* = 3.7 Hz), 124.3 (q, *J* = 272.2 Hz), 122.9 (q, *J* = 273.3 Hz), 121.9, 34.8, 31.4.

^{19}**F NMR** (565 MHz, $CDCl_3$) δ -63.1, -63.4.

HRMS (ESI-Orbitrap) calc'd for $C_{24}H_{21}NO_4S_2F_6Na$ $[M+Na]^+$: 588.0708, found: 588.0709.

HPLC Zorbax Eclipse Plus, C18 1.8 µm, 4.6 x 50 mm; gradient 70% to 90% (5 min) MeOH/H_2O, 1.0 mL/min, 220 nm; t_R = 4.97 min.

R_f (pentane:EtOAc = 85:15) 0.77

dimethyl 4,4'-(((4-(tert-butyl)phenyl)(hydrosulfonyl)amino)sulfonyl)dibenzoate (2ac)

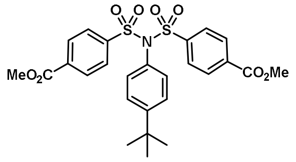

Following General procedure for condition A, starting from 13.4 mg of 4-tertbutylphenylboronic acid (0.075 mmol) and 64.7 mg of dimethyl 4,4'-((fluoro(hydrosulfonyl)amino)sulfonyl)dibenzoate (0.150 mmol), compound **2ac** (16.2 mg, 40% yield) was isolated as a white solid.

^{1}**H NMR** (600 MHz, $CDCl_3$) δ 8.24 – 8.20 (m, 4H), 8.05 – 8.01 (m, 4H), 7.39 – 7.36 (m, 2H), 6.93 – 6.90 (m, 2H), 3.99 (s, 6H), 1.33 (s, 9H).

^{13}**C NMR** (151 MHz, $CDCl_3$) δ 165.6, 154.2, 143.2, 135.1, 131.0, 130.9, 130.3, 128.8, 126.7, 52.9, 35.1, 31.4.

HRMS (ESI-Orbitrap) calc'd for $C_{26}H_{27}NO_8S_2Na$ $[M+Na]^+$: 568.1070, found: 568.1064.

HPLC Zorbax Eclipse Plus, C18 1.8 µm, 4.6 x 50 mm; gradient 70% to 90% (5 min) MeOH/H_2O, 1.0 mL/min, 220 nm; t_R = 4.56 min.

R_f (pentane:EtOAc = 85:15) 0.28

methyl 4-(N-((4-(tert-butyl)phenoxy)(4-(methoxycarbonyl)phenyl)(oxo)-l6-sulfaneylidene)sulfamoyl)benzoate (3ac)

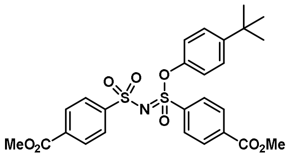

Following General procedure for condition B, starting from 13.4 mg of 4-tertbutylphenylboronic acid (0.075 mmol) and 64.7 mg of dimethyl 4,4'-((fluoro(hydrosulfonyl)amino)sulfonyl)dibenzoate (0.150 mmol), compound **3ac** (15.8 mg, 39% yield) was isolated as a white solid.

^{1}H NMR (600 MHz, $CDCl_3$) δ 8.21 – 8.19 (m, 2H), 8.12 – 8.09 (m, 2H), 8.03 – 8.00 (m, 4H), 7.30 – 7.27 (m, 2H), 6.94 – 6.90 (m, 2H), 3.98 (s, 3H), 3.94 (s, 3H), 1.28 (s, 9H).

^{13}C NMR (151 MHz, $CDCl_3$) δ 165.9, 165.2, 151.6, 146.5, 146.4, 139.3, 136.1, 133.8, 130.6 (d, J = 3.5 Hz), 130.2, 128.5, 128.2, 127.1, 122.0, 53.1, 52.7, 35.2, 34.8, 31.4.

HRMS (ESI-Orbitrap) calc'd for $C_{26}H_{27}NO_8S_2Na$ $[M+Na]^+$: 568.1070, found: 568.1066.

HPLC Zorbax Eclipse Plus, C18 1.8 μm, 4.6 x 50 mm; gradient 70% to 90% (5 min) MeOH/H_2O, 1.0 mL/min, 220 nm; t_R = 3.90 min.

R_f (pentane:EtOAc = 85:15) 0.14

5.2. Unsuccessful substrates

Some arylboronic acids derivatives failed to produce the desired coupling products during the evaluation of the scope of the reaction (**Figure S3**). Particularly, electron-rich boronic acids proved to be reactive with NFSI,[7] leading to unwanted side reactivity. Moreover, ortho-substituted boronic acids were also not tolerated in the transformation.

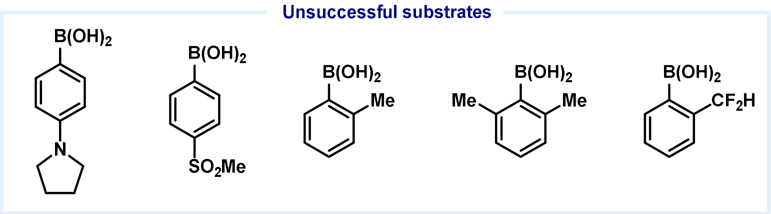

Figure S3 Unsuccessful substrates. Reactions performed using the general procedure for condition A.

6. Reproducibility issues

While evaluating the reaction scope, reproducibility issues were observed. After careful analysis, the origin of the boronic acid derivative could lead to dramatic yield change. **Tables S6** and **S7** present some observed irregularities. Surprisingly, even after careful NMR and HRMS analysis; the non-productive starting materials appear to be identical to the working ones. Moreover, when engaged in a typical Suzuki-Miyaura coupling, similar yields were observed regardless of the supplier. Purification by recrystallization as well as use of typical additive failed to restore reactivity in our reaction. *When synthesizing ourselves the boronic acid derivative (either by lithiation borylation from the corresponding aryl bromide or by Miyaura borylation followed by oxidation), satisfactory and reproducible yields were observed. Consequently, the entire scope evaluation was performed with home-made arylboronic acids.*

Table S6 Reproducibility issues with 4-tertbutylphenylboronic acid. Yields were determined by quantitative ^{1}H NMR using CH_2Br_2 as an internal standard.

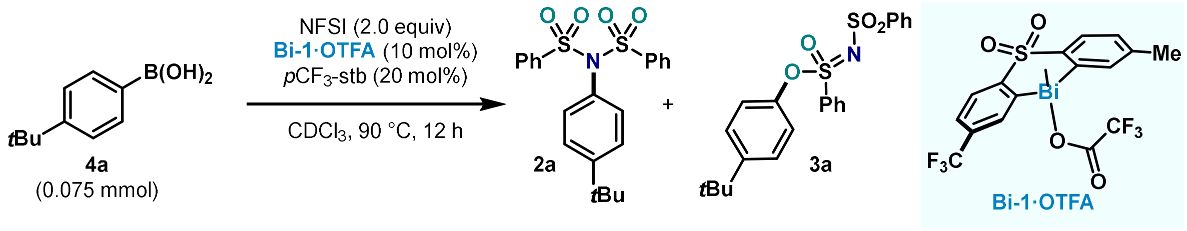

Entry	Supplier	Purification	Overall yield
1	Supplier A (Lot 1)	-	67%
2	Supplier B	-	7%
3	Supplier B	Recristallized in H_2O	10%
4	Supplier B	Recristallized in H_2O + dry on Drierite and high vacuum	22%
5	Supplier B	Entry 4 + 4 Å MS or KH_2PO_4 or $Si(OEt)_4$	18-27%
6	Supplier C	-	73% (18 h)
7	Supplier A (Lot 2)	-	85% (18 h)
8	Homemade by lithiation/borylation	Column chromatography	66%
9	Homemade by Miyaura borylation	Column chromatography	80% (18 h)

Table S7 Reproducibility issues with phenylboronic acid. Yields were determined by quantitative ^{1}H NMR using CH_2Br_2 as an internal standard.

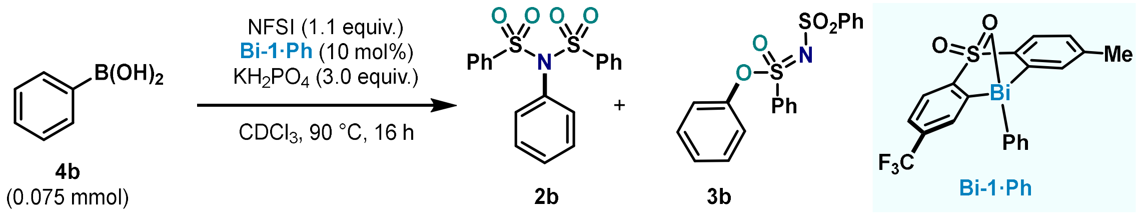

Entry	Supplier	Overall yield
1	Supplier A 98%+	68%
2	Supplier B 95%	17%
3	Supplier C 99.95%	37%
4	Supplier D 98%	60%
5	Supplier E	54%

7. Additives effects

7.1. Stilbene

Procedure for catalyst stability evaluation

A flame dried culture tube equipped with a Teflon-coated stir bar was charged with the suitable bismuth complex (0.075 mmol, 1.0 equiv.), and (*E*)-1,2-bis(4-(trifluoromethyl)phenyl)ethene (47.4 mg, 0.15 mmol, 1.5 equiv.). The culture tube was closed with a Teflon screw-cap, connected to an argon/vacuum Schlenk line and evacuated and refilled with argon (3 cycles). Anhydrous and degassed $CDCl_3$ (0.75 mL, 0.1 M) was added through the septa. The reaction vessel was then sealed using two pieces (*ca.* 2 cm) of electrical tape, the first one covering needle punctures and the second one around the cap thread. The reaction was stirred (600 rpm) for 16 h at 100 °C in a pre-heated oil bath. After the reaction time, the crude solution was allowed to cool down to room temperature and a known amount of CH_2Br_2 was added as an internal standard. Quantitative ^{1}H NMR was then recorded to determine the yield of the transformation.

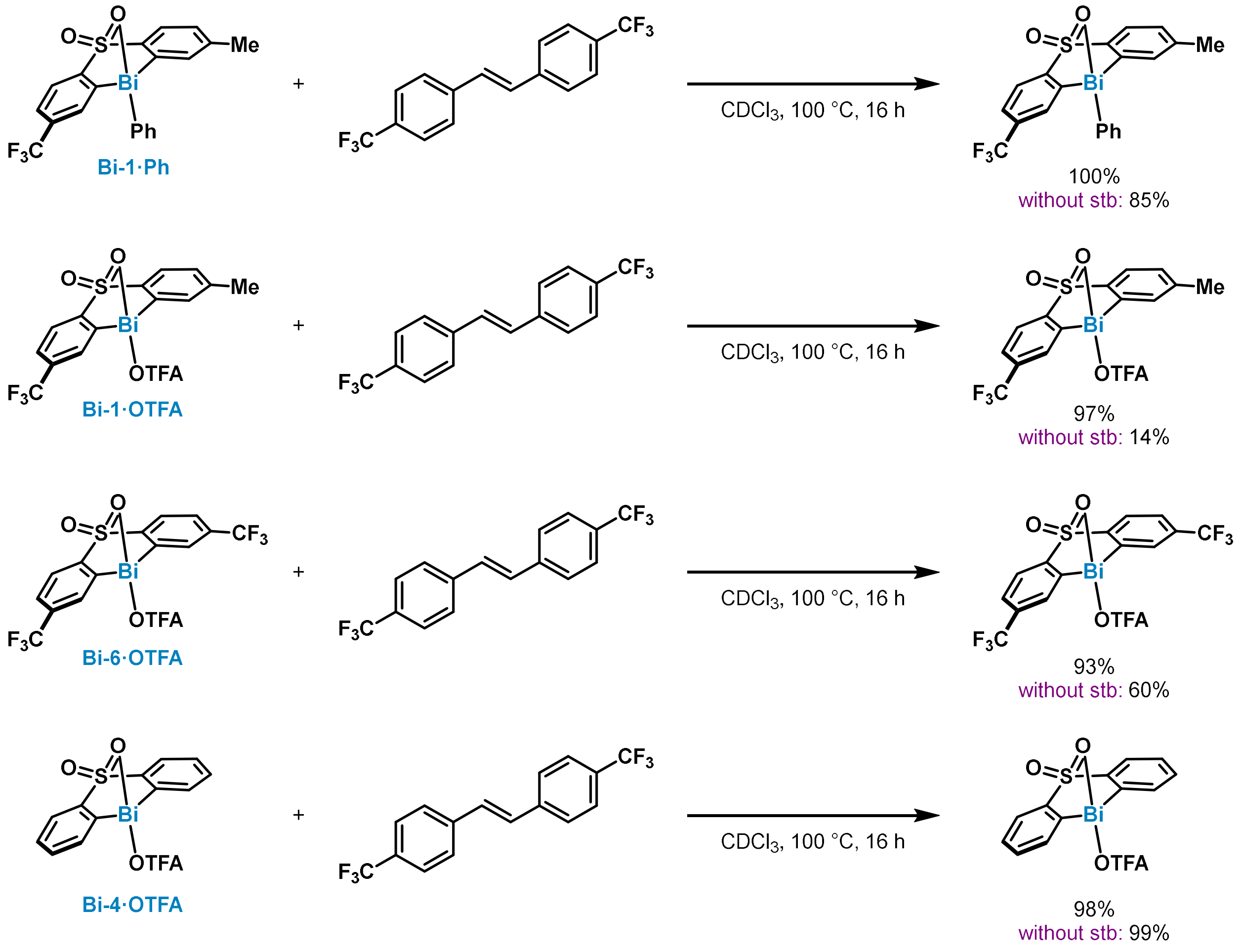

Figure S4 Evaluation of catalyst stability under prolonged heating in presence or absence of stilbene additive. Yields determined by ^{1}H NMR using CH_2Br_2 as an internal standard.

Stilbene stability in oxidative condition was also confirmed as exposure of *p*-CF_3-stb to NFSI under prolonged heating did not lead to any reaction.

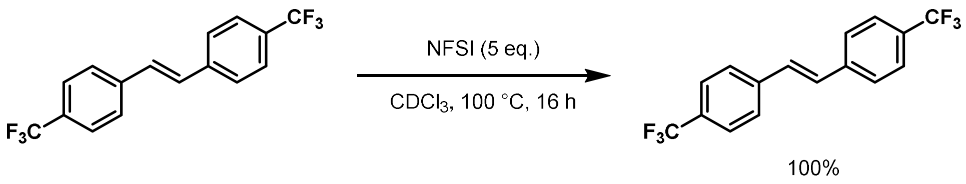

Figure S5 Stability of selected stilbene under oxidative conditions. Yield determined by ^{1}H NMR using CH_2Br_2 as an internal standard.

7.2. $Si(OEt)_4$

Table S8 Further optimization toward selective C(sp²)–O coupling by screening reported fluoride anion scavenger. Yields were determined by quantitative ^{1}H NMR using CH_2Br_2 as an internal standard. Selectivities were determined by HPLC using the protocol described in §3.

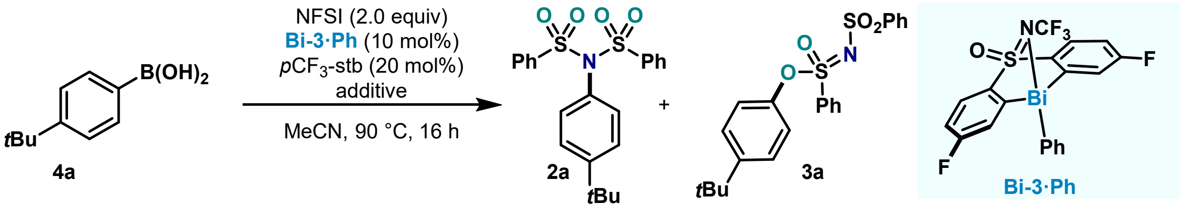

Entry	Additive	Combined Yield	3a:2a
1	-	37%	3.73:1
2	$Si(OEt)_4$ (1.0 equiv.)	43%	5.60:1
**3**	**$Si(OEt)_4$ (25 mol%)**	49%	5.80:1
4	$Si(OEt)_4$ (10 mol%)	42%	nd
5	trimethyl((1-phenylvinyl)oxy)silane (1.0 equiv.)	traces	-
6	$Me_3Si\text{-}SiMe_3$ (1.0 equiv.)	21%	1.69:1
7	$TMSOCH_2\text{-}CH_2OTMS$ (1.0 equiv.)	no product	-
8	B_2pin_2 (1.0 equiv.)	traces	-

8. Monitoring

Procedure for NMR monitoring

A flame dried culture tube equipped with a Teflon-coated stir bar was charged with 2-methyl-5,5-dioxido-8-(trifluoromethyl)-10H-dibenzo[b,e][1,4]thiabismin-10-yl 2,2,2-trifluoroacetate **Bi-1·OTFA** (0.0075 mmol, 10 mol%), NFSI (47.3 mg, 0.15 mmol, 2.0 equiv.), (4-tertbutyl)phenyl boronic acid **4a** (13.4 mg, 0.075 mmol, 1.0 equiv.) and (*E*)-1,2-bis(4-(trifluoromethyl)phenyl)ethene (4.7 mg, 0.015 mmol, 20 mol%). The culture tube was closed with a Teflon screw-cap, connected to an argon/vacuum Schlenk line and evacuated and refilled with argon (3 cycles). Anhydrous and degassed $CDCl_3$ (0.75 mL, 0.1 M) was added as well as a known amount of 1,3,5-trifluorobenzene as an internal standard. The reaction was stirred at room temperature for 5 min until obtaining a clear homogeneous mixture. Then, the reaction was moved to an argon-filled glovebox and transferred to an oven-dried J Young NMR tube and sealed with a Teflon-threaded cap. The sample was then inserted in a preheated (363 K) 300 MHz NMR, the spectrometer was tuned, matched, locked and shimmed. ^{1}H and $^{19}F\{^{1}H\}$ spectra were then acquired every 6 min for 24 h.

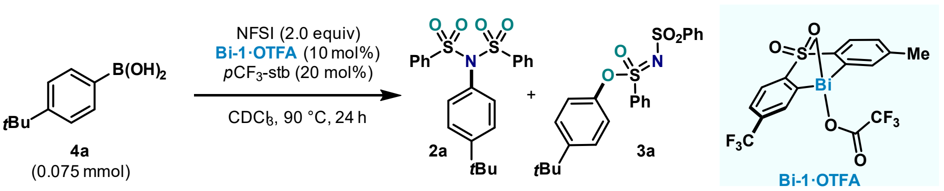

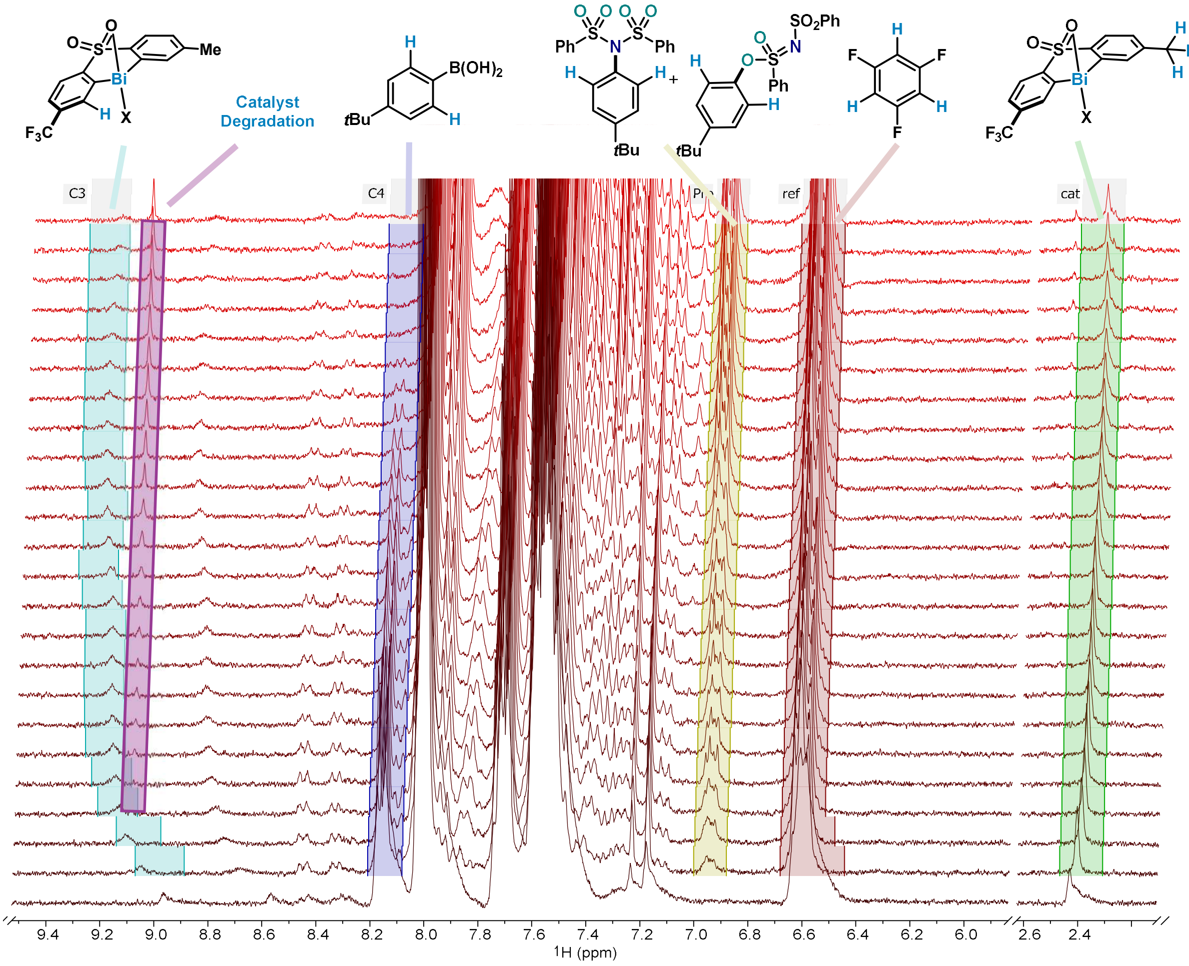

Figure S6 Stacked selected spectra for continuous ^{1}H NMR monitoring of the reaction of NFSI and 4-tertbutylphenylboronic acid catalyzed by **Bi-1·OTFA**. The highlighted peaks were used to construct plots of concentration versus time for the various reaction components, using 1,3,5-

trifluorobenzene (6.6 ppm, red band) as a true internal standard. In order of decreasing chemical shift value: Bi(III)–X catalyst (9.2 ppm, cyan band), catalyst decomposition (9.1 ppm, purple band), boronic acid (8.2 ppm, blue band), mixture of products (6.9 ppm, yellow band), Bi(III)–X catalyst –Me signal (2.4 ppm, green band)

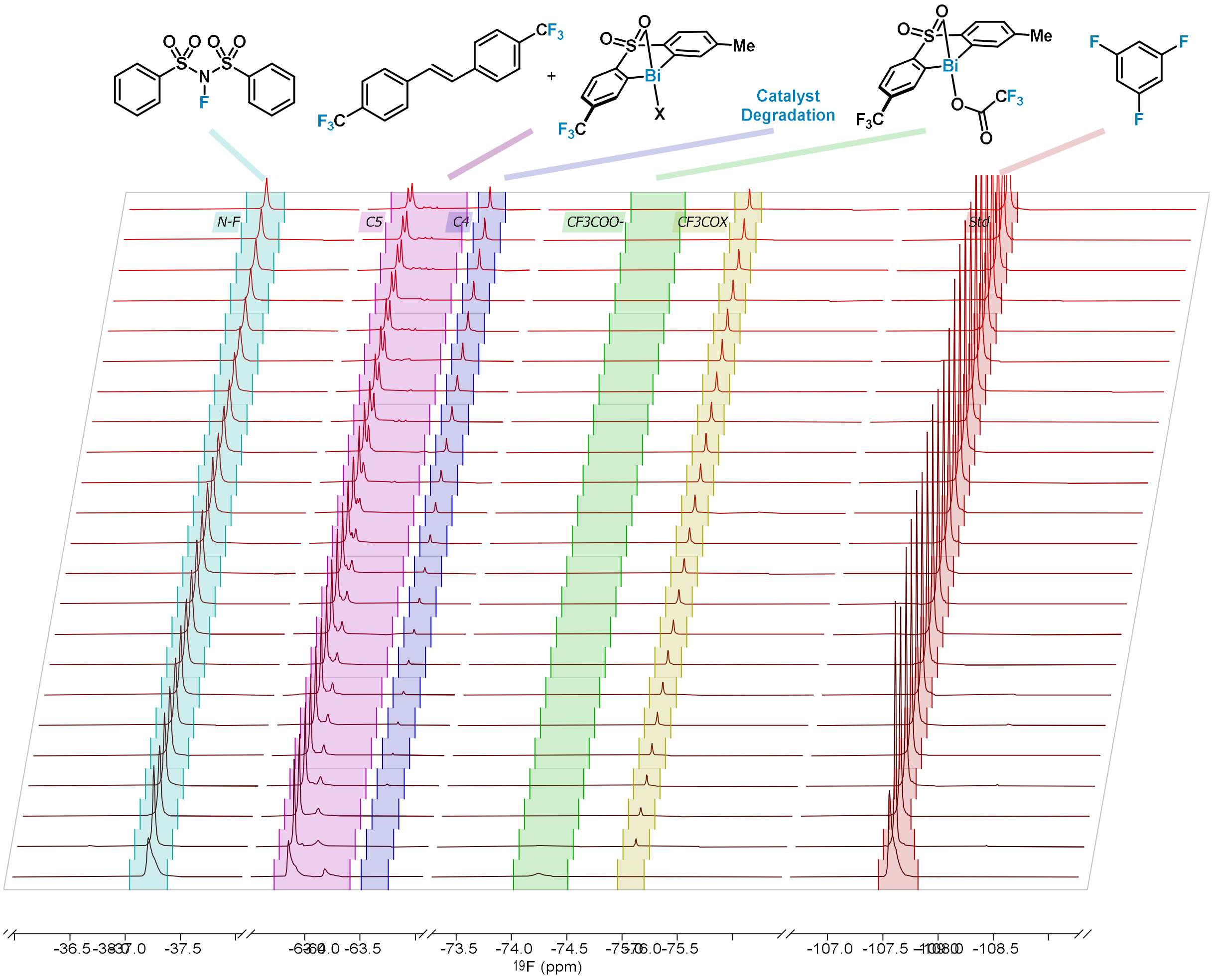

Figure S7 Stacked selected spectra for continuous $^{19}F\{^1H\}$ NMR monitoring of the reaction of NFSI and 4-tertbutylphenylboronic acid catalyzed by **Bi-1·OTFA**. The highlighted peaks were used to construct plots of concentration versus time for the various reaction components, using 1,3,5-trifluorobenzene (-107.5 ppm, red band) as a true internal standard. In order of decreasing chemical shift value: NFSI (-37.2 ppm, cyan band), Bi(III)–X catalyst C(sp²)–CF_3 signal overlapped with stilbene signal (-63.0 ppm, purple band), catalyst decomposition (-63.6 ppm, blue band), Bi(III)–OCOCF_3 catalyst (-74.3 ppm, green band), CF_3COX signal (-75.2 ppm, yellow band).

The formation of the mixture of products and the consumption of starting material (NFSI) was then plotted against time (Figure S8). NFSI was chosen for monitoring instead of the arylboronic acid derivative as the latter is transformed into the boroxine along the reaction course, which does not allow quantitative interpretation.

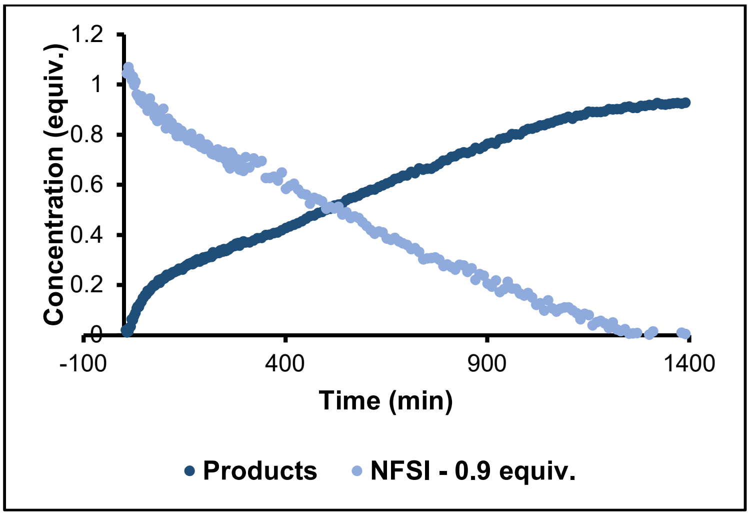

Figure S8 Dark blue: concentration of the mixture of products **2a** and **3a** against time extracted from ^{1}H NMR. Light blue: concentration of NFSI -0.9 equiv. against time extracted from ^{19}F{^{1}H} NMR.

^{1}H NMR monitoring allows us to identify the catalyst resting state as $Ar_2Bi(III)$–X where –X is evolving from the initial anion (–OTFA in that case) to –NSI. This was validated by overlapping ^{1}H NMR spectra at different time points by monitoring against isolated bismuth complexes (**Figure S9**).

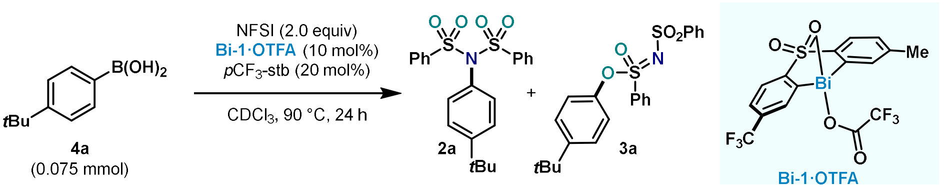

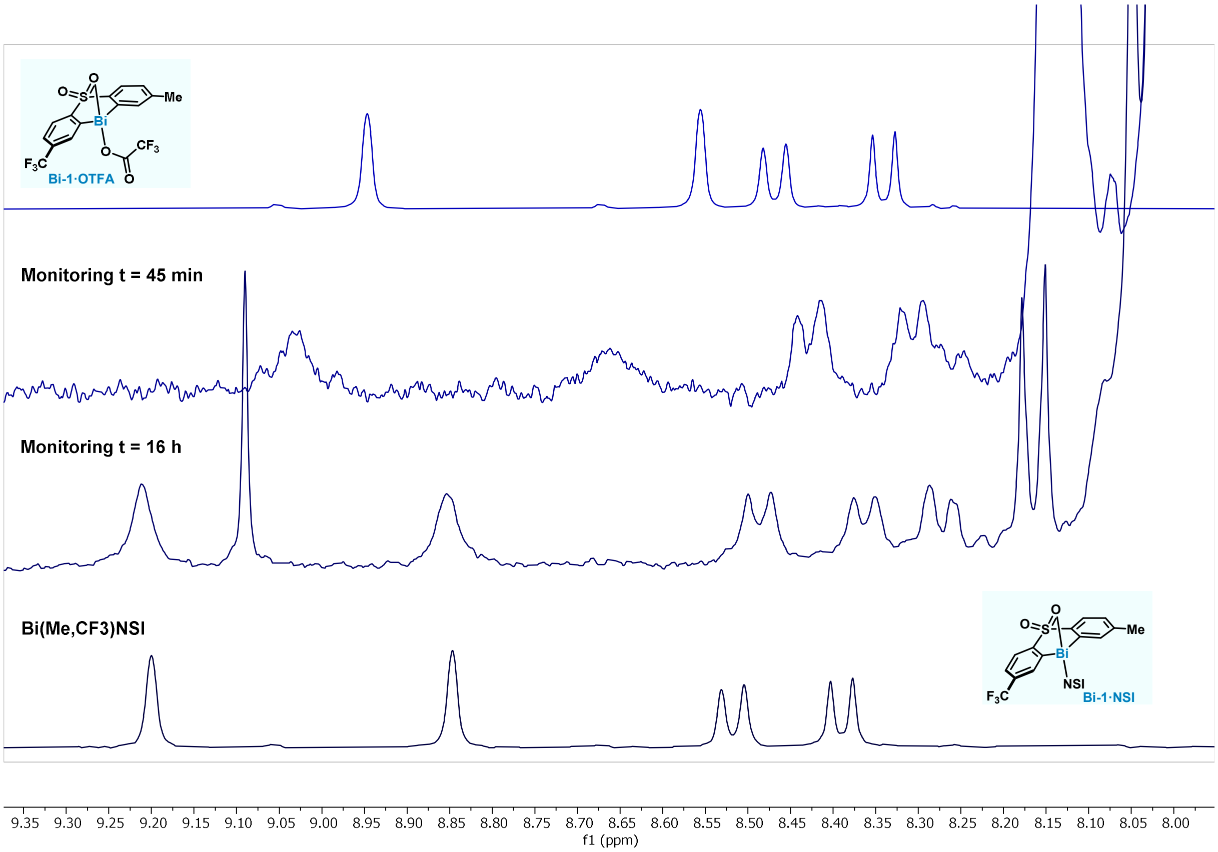

Figure S9 ^{1}H NMRs Bi(III)–X fingerprints from top to bottom: pure **Bi-1·OTFA** catalyst; crude reaction mixture after 45 min of heating; crude reaction mixture after 16 h of heating; pure **Bi-1·NSI** catalyst.

During the reaction, catalyst decomposition was also observed by both ^{1}H (s, 9.08 ppm) and ^{19}F (s, -63.6 ppm) NMRs. By correction of mass balance, the distinct signal in ^{1}H NMR was identified to account for 1 proton. Kinetics of the decomposition were then compared using either **Bi-1·OTFA** in $CDCl_3$ or MeCN, or **Bi-1·Cl** in $CDCl_3$ using the following procedure. *Note: as the crude reaction mixture is heterogeneous in MeCN, in situ NMR monitoring is not possible.*

Procedure for manual monitoring in $CDCl_3$

A flame dried Schlenk flask equipped with a Teflon-coated stir bar was charged with the desired bismuth catalyst (0.075 mmol, 10 mol%), NFSI (473 mg, 1.5 mmol, 2.0 equiv.), (4-tertbutyl)phenyl boronic acid (134 mg, 0.75 mmol, 1.0 equiv.) **4a** and (*E*)-1,2-bis(4-(trifluoromethyl)phenyl)ethene (47 mg, 0.15 mmol, 20 mol%). The Schlenk flask was closed, connected to an argon/vacuum Schlenk line and evacuated and refilled with argon (3 cycles). Anhydrous and degassed $CDCl_3$ (7.5 mL, 0.1 M) was added. The reaction was stirred at room temperature for 5 min until obtaining a clear homogeneous mixture. Then, the reaction was equally divided with a syringe into ten flame dried culture tube equipped with a Teflon-coated stir bar. The reaction vessels were then sealed using two pieces (*ca.* 2 cm) of electrical tape, the first one covering needle punctures and the second one around the cap thread. The reactions were stirred (600 rpm) at 90 °C in a pre-heated oil bath for the appropriate time. After the desired reaction time, the culture tube was immerged in a 0 °C ice cold bath in order to quench the

reaction. A known amount of a 0.1 M CH_2Br_2 solution in $CDCl_3$ was added. Quantitative ^{1}H NMR was then recorded in order to evaluate the amount of catalyst decomposition.

Procedure for manual monitoring in MeCN

A flame dried culture tube equipped with a Teflon-coated stir bar was charged with 2-methyl-5,5-dioxido-8-(trifluoromethyl)-10H-dibenzo[b,e][1,4]thiabismin-10-yl 2,2,2-trifluoroacetate **Bi-1·OTFA** (0.0075 mmol, 10 mol%), NFSI (47.3 mg, 0.15 mmol, 2.0 equiv.), (4-tertbutyl)phenyl boronic acid (13.4 mg, 0.075 mmol, 1.0 equiv.) and (*E*)-1,2-bis(4-(trifluoromethyl)phenyl)ethene (4.7 mg, 0.015 mmol, 20 mol%). The culture tube was closed with a Teflon screw-cap, connected to an argon/vacuum Schlenk line and evacuated and refilled with argon (3 cycles). The procedure was repeated nine times in order to prepare ten reaction mixtures. Anhydrous and degassed MeCN (0.75 mL, 0.1 M) was then added to each culture tube. The reaction vessels were then sealed using two pieces (*ca.* 2 cm) of electrical tape, the first one covering needle punctures and the second one around the cap thread. The reactions were stirred (600 rpm) at 90 °C in a pre-heated oil bath for the appropriate time. After the desired reaction time, the culture tube was immerged in a 0 °C ice cold bath in order to quench the reaction. The solvent was removed under reduced pressure and a known amount of a 0.1 M CH_2Br_2 solution in $CDCl_3$ was added. Quantitative ^{1}H NMR was then recorded in order to evaluate the amount of catalyst decomposition.

Figure S10 is depicting the catalyst decomposition in the evaluated conditions. Decomposition kinetic appears to be faster in acetonitrile rather than in $CDCl_3$, in line with the lower yields observed in this solvent.

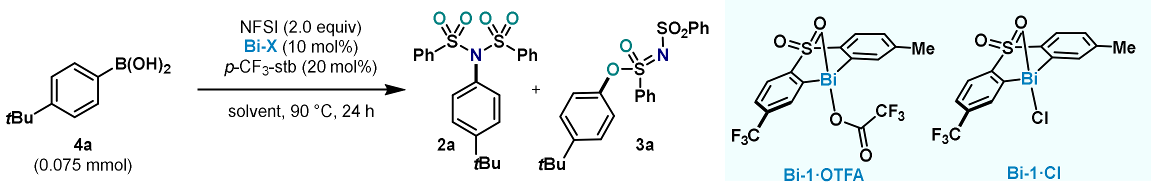

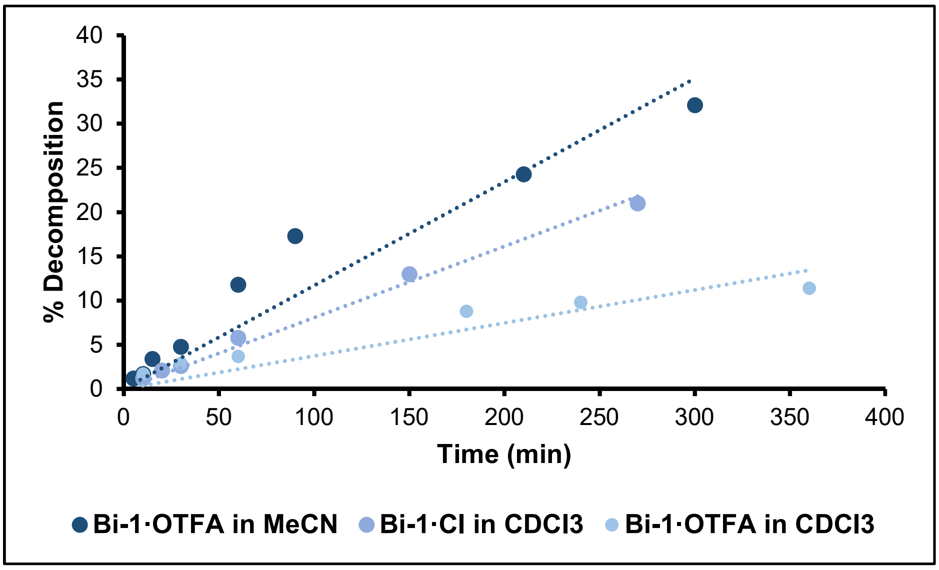

Figure S10 Catalyst decomposition (in percent of the catalyst amount; 100% corresponds to a total decomposition) in function of reaction time determined by quantitative ^{1}H NMR in various condition. Dark blue: **Bi-1·OTFA** catalyst in MeCN, blue: **Bi-1·Cl** catalyst in $CDCl_3$, light blue: **Bi-1·OTFA** catalyst in $CDCl_3$.

9. Stoichiometric experiments

9.1. Stoichiometric reactivity of Bi(III)–Ar with NFSI

Procedure for stoichiometric coupling

A flame dried culture tube equipped with a Teflon-coated stir bar was charged with 10-(4-(tert-butyl)phenyl)-2-methyl-8-(trifluoromethyl)-10H-dibenzo[b,e][1,4]thiabismine 5,5-dioxide **Bi-1·4-*t*BuPh** (48.0 mg, 0.075 mmol, 1.0 equiv.) and NFSI (47.3 mg, 0.15 mmol, 2.0 equiv.). The culture tube was closed with a Teflon screw-cap, connected to an argon/vacuum Schlenk line and evacuated and refilled with argon (3 cycles). Anhydrous and deoxygenated appropriate solvent (0.75 mL, 0.1 M) was added through the septa. The reaction vessel was then sealed using two pieces (*ca.* 2 cm) of electrical tape, the first one covering needle punctures and the second one around the cap thread. The reaction was stirred (600 rpm) for 16 h at 90 °C in a pre-heated oil bath. After the reaction time, the crude solution was allowed to cool down to room temperature, concentrated in the culture tube at reduced pressure and dissolved in $CDCl_3$ with a known amount of CH_2Br_2 as an internal standard. Quantitative ^{1}H NMR was then recorded to determine the yield of the transformation, as well as an estimation of the reaction selectivity using line fitting.

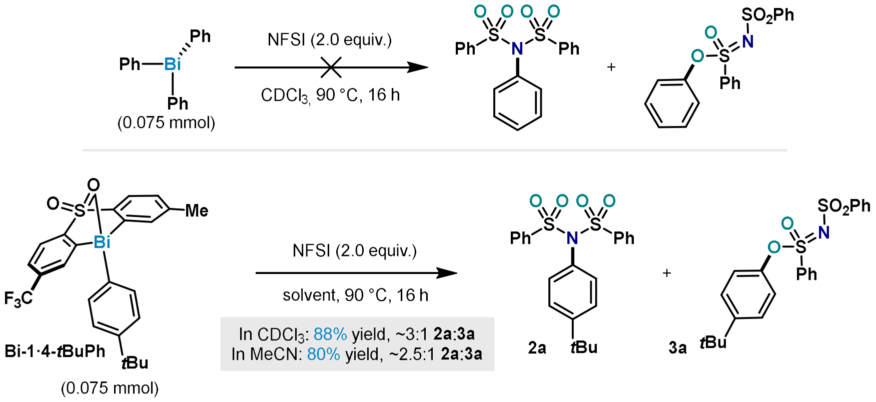

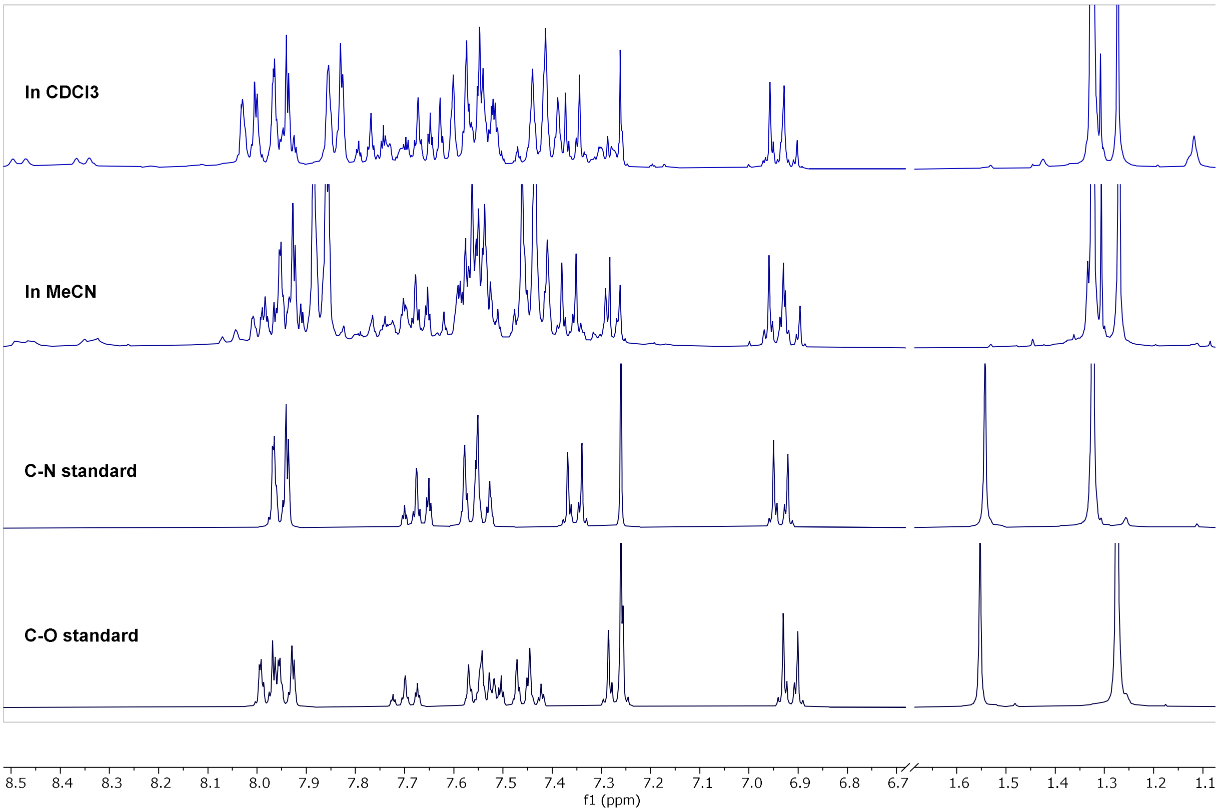

Figure S11 Stoichiometric reaction following the procedure for stoichiometric coupling. Yields were determined by quantitative ^{1}H NMR using CH_2Br_2 as an internal standard. Selectivities were roughly estimated using quantitative ^{1}H NMR analysis and MestReNova line fitting feature. Corresponding ^{1}H NMR ($CDCl_3$) spectra of the crude reaction mixture in stacked with isolated products. From top to bottom: reaction in $CDCl_3$; reaction in MeCN; pure C(sp^2)–N product **2a**; pure C(sp^2)–O product **3a**.

9.2. Products stability

Procedure for product stability

A flame dried culture tube equipped with a Teflon-coated stir bar was charged either with N-(4-(tert-butyl)phenyl)-N-(phenylsulfonyl)benzenesulfonamide **2a** (32.2 mg, 0.075 mmol, 1.0 equiv.) or 4-(tert-butyl)phenyl N-(phenylsulfonyl)benzenesulfonimidate **3a** (32.2 mg, 0.075 mmol, 1.0 equiv.). If needed the solid additive was weighted. The culture tube was closed with a Teflon screw-cap, connected to an argon/vacuum Schlenk line and evacuated and refilled with argon (3 cycles). Anhydrous and degassed $CDCl_3$ (0.75 mL, 0.1 M) was added through the septa. The reaction vessel was then sealed using two pieces (*ca.* 2 cm) of electrical tape, the first one covering needle punctures and the second one around the cap thread. The reaction was stirred (600 rpm) for 16 h at 100 °C in a pre-heated oil bath. After the reaction time, the crude solution was allowed to cool down to room temperature and a known amount of mesitylene was added as an internal standard. Quantitative ^{1}H NMR was then recorded to determine the yield of the transformation.

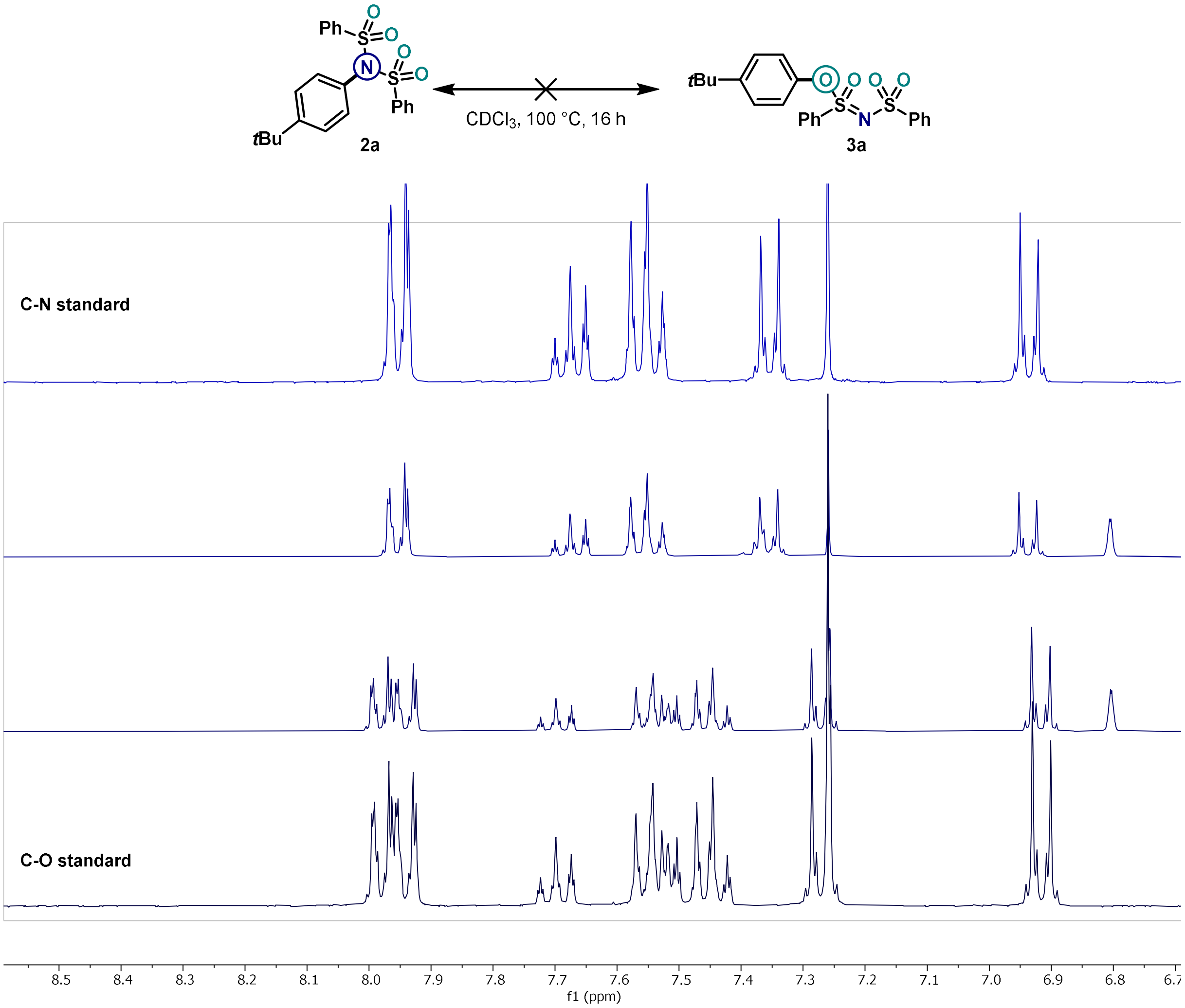

Figure S12 Stacked ^{1}H NMR ($CDCl_3$) spectra of isomerization control without additive following the procedure for product stability. From top to bottom: pure C(sp²)–N product **2a**; crude reaction mixture using **2a** as starting material; crude reaction mixture using **3a** as starting material; pure C(sp²)–O product **3a**.

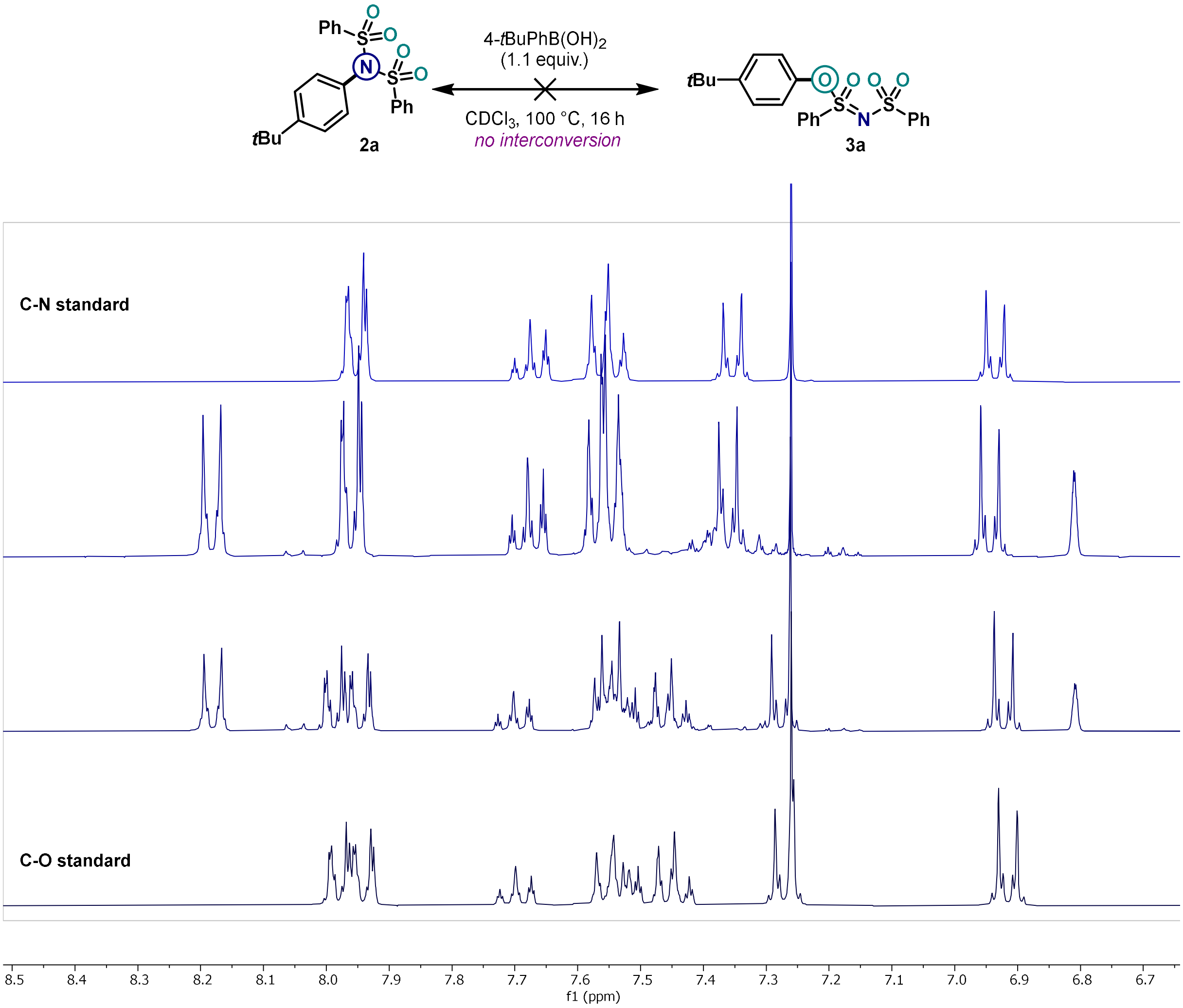

Figure S13 Stacked ^{1}H NMR ($CDCl_3$) spectra of isomerization control with arylboronic acid additive following the procedure for product stability. From top to bottom: pure C(sp^2)–N product **2a**; crude reaction mixture using **2a** as starting material; crude reaction mixture using 3**a** as starting material; pure C(sp^2)–O product **3a**.

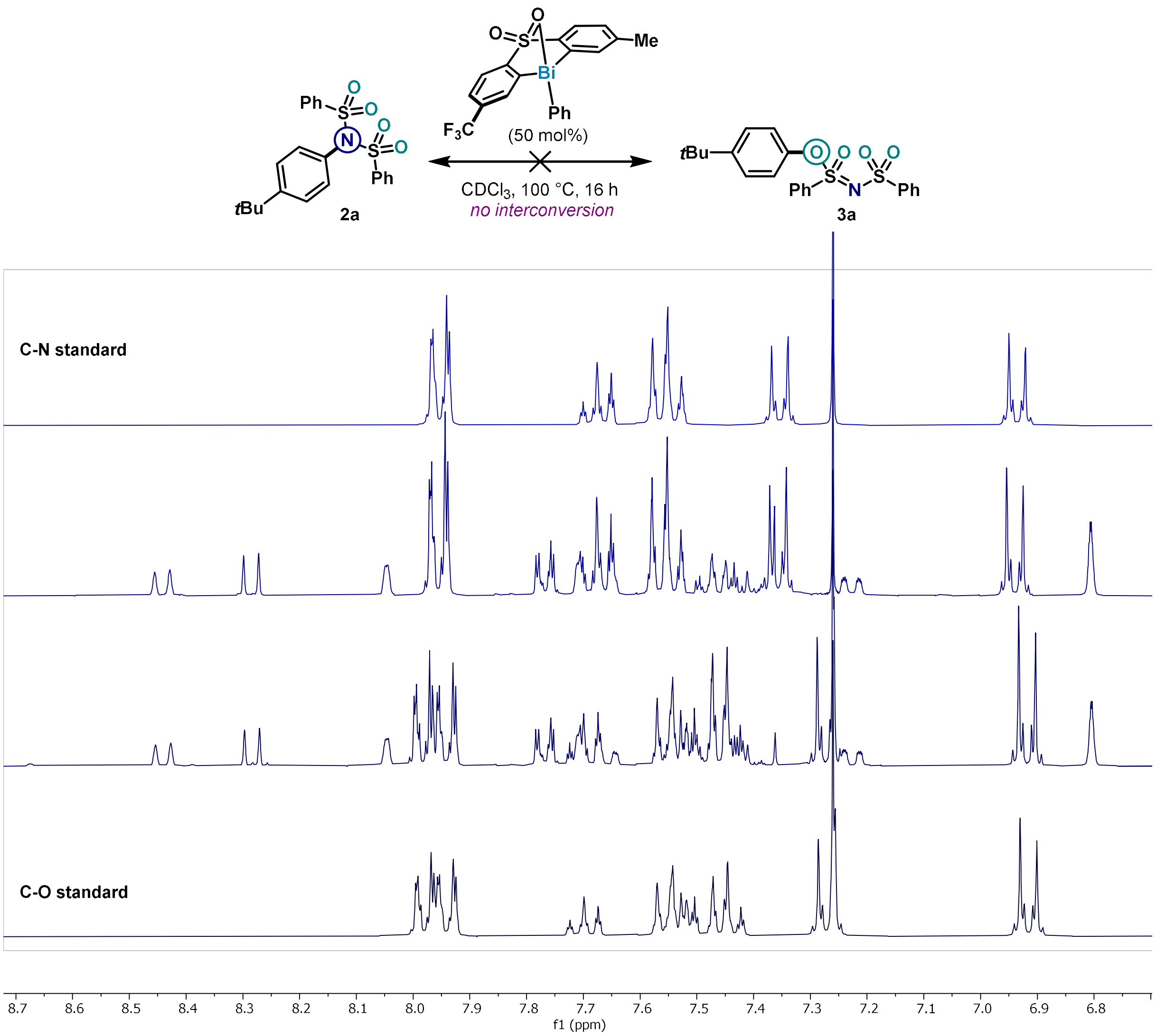

Figure S14 Stacked ^{1}H NMR ($CDCl_3$) spectra of isomerization control with bismuth complex additive **Bi-1·Ph** following the procedure for product stability. From top to bottom: pure C(sp^2)–N product **2a**; crude reaction mixture using **2a** as starting material; crude reaction mixture using 3**a** as starting material; pure C(sp^2)–O product **3a**.

9.3. Transmetalation studies

Absence of transmetalation of $Ar_2Bi(III)$–X with 4-tertbutylphenylboronic acid **4a** was controlled using the following procedure:

Procedure for transmetalation

A flame dried culture tube equipped with a Teflon-coated stir bar was charged with the appropriate $Ar_2Bi(III)$–X complex (0.075 mmol, 1.0 equiv.), 4-tertbutylphenylboronic acid **4a** (16.1 mg, 0.090 mmol, 1.2 equiv.) and sodium bis(phenylsulfonyl)amide **5** (23.9 mg, 0.075 mmol, 1.0 equiv.). The culture tube was closed with a Teflon screw-cap, connected to an argon/vacuum Schlenk line and evacuated and refilled with argon (3 cycles). Anhydrous and deoxygenated appropriate solvent (0.75 mL, 0.1 M) was added through the septa. The reaction vessel was then sealed using two pieces (*ca.* 2 cm) of electrical tape, the first one covering needle punctures and the second one around the cap thread. The reaction was stirred (600 rpm) for 16 h at 90 °C in a pre-heated oil bath. After the reaction time, the crude solution was allowed to cool down to room temperature and a known amount of CH_2Br_2 was

added as an internal standard. Quantitative ^{1}H NMR was then recorded to determine the yield of the transformation.

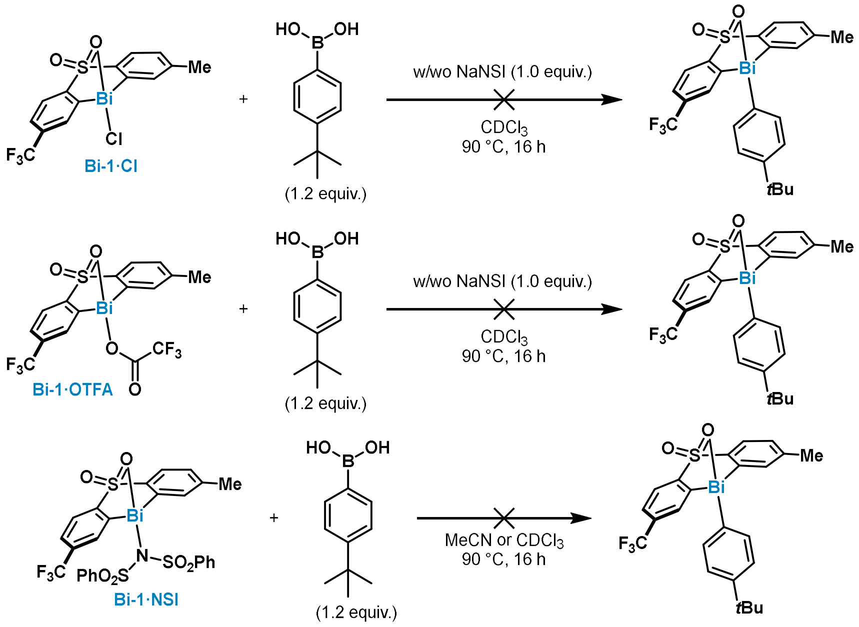

Figure S15 Absence of transmetalation with arylboronic acid of various Bi(III) complexes in the catalysis conditions validated by stoichiometric reactions.

9.4. Oxidative addition

Attempts to identify oxidation product resulting from NFSI reaction with $Ar_2Bi(III)$–X failed. In line with such observation, to the best of our knowledge, no Bi(V) derivative possessing only two Bi–C bonds are reported in the literature. Nonetheless, loss of mass balance was observed when exposing several $Ar_2Bi(III)$–X to NFSI as depicted in the **Figure S16**.

Procedure for $Ar_2Bi(III)$–X oxidation

A flame dried culture tube equipped with a Teflon-coated stir bar was charged with the appropriate $Ar_2Bi(III)$–X complex (0.075 mmol, 1.0 equiv.), and NFSI (47.3 mg, 0.15 mmol, 2.0 equiv.). The culture tube was closed with a Teflon screw-cap, connected to an argon/vacuum Schlenk line and evacuated and refilled with argon (3 cycles). Anhydrous and degassed appropriate solvent (0.75 mL, 0.1 M) was added through the septa. The reaction vessel was then sealed using two pieces (*ca.* 2 cm) of electrical tape, the first one covering needle punctures and the second one around the cap thread. The reaction was stirred (600 rpm) for 16 h at 90 °C in a pre-heated oil bath. After the reaction time, the crude solution was allowed to cool down to room temperature and a known amount of CH_2Br_2 was added as an internal standard. Quantitative ^{1}H NMR was then recorded to determine the yield of the transformation.

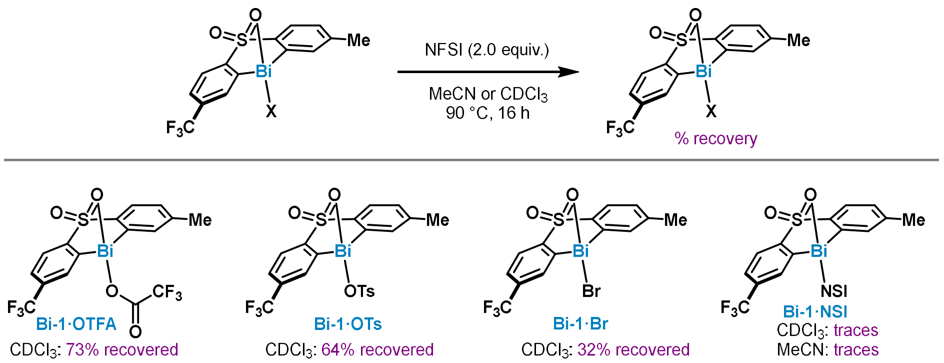

Figure S16 Decomposition of $Ar_2Bi(III)–X$ when exposed to NFSI. Recovery percentage determined by quantitative ^1H NMR using CH_2Br_2 as an internal standard.

9.5. Reaction with an external oxidant

In order to improve the catalytic transformation, we try to use a more potent oxidant to facilitate the oxidation step. To do so we chose to use two distinct reactants instead of NFSI: one for oxidation one as nucleophile. XeF_2 however, is a super strong oxidant whose high reactivity leads to numerous side reactions. In the past, we did attempt slow addition of XeF_2; yet, its low solubility and reactivity in the solvent used precluded this method. [8] Hence, we tested 2,6-dichloro-1-fluoropyridinium tetrafluoroborate, as it was successful in our group in the past to access Bi(V).[1,3] NaNSI was then used as nucleophile. Unfortunately, no desired product was observed in such conditions.

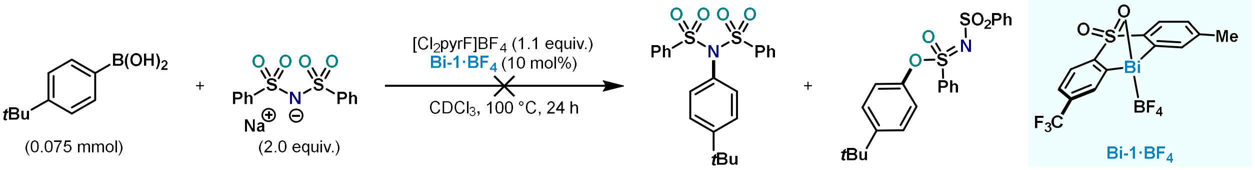

A flame dried culture tube equipped with a Teflon-coated stir bar was charged with **Bi-1·BF$_4$** (4.5 mg, 0.0075 mmol, 10 mol%), 4-tertbutylphenylboronic acid **4a** (13.4 mg, 0.075 mmol, 1.0 equiv.) and sodium bis(phenylsulfonyl)amide **5** (47.8 mg, 0.15 mmol, 2.0 equiv.). The culture tube was transferred to an argon-filled glovebox and 2,6-dichloro-1-fluoropyridinium tetrafluoroborate (20.9 mg, 0.083 mmol, 1.1 equiv.) was added. The culture tube was closed with a Teflon screw-cap and connected to an argon/vacuum Schlenk line. Anhydrous and deoxygenated $CDCl_3$ (0.75 mL, 0.1 M) was added through the septa. The reaction vessel was then sealed using two pieces (*ca.* 2 cm) of electrical tape, the first one covering needle punctures and the second one around the cap thread. The reaction was stirred (600 rpm) for 24 h at 100 °C in a pre-heated oil bath. After the reaction time, the crude solution was allowed to cool down to room temperature and a known amount of CH_2Br_2 was added as an internal standard. Quantitative ^1H NMR was then recorded to determine the yield of the transformation.

9.6. Stoichiometric reductive elimination

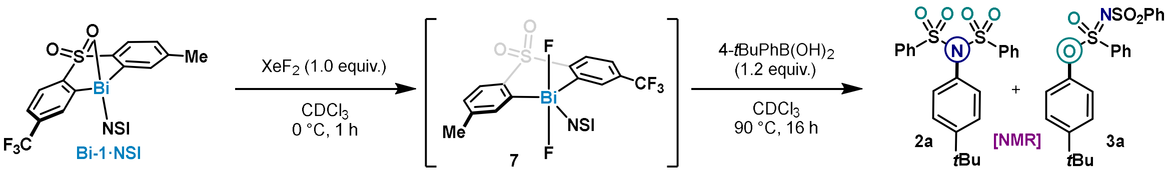

A flame dried culture tube equipped with a Teflon-coated stir bar was charged with 10-((iodo-l3-sulfaneylidene)amino)-2-methyl-8-(trifluoromethyl)-10H-dibenzo[b,e][1,4]thiabismine 5,5-dioxide **Bi-1·NSI** (60.3 mg, 0.075 mmol, 1.0 equiv.). The culture tube was closed with a Teflon screw-cap,

connected to an argon/vacuum Schlenk line and evacuated and refilled with argon (3 cycles). Anhydrous and degassed $CDCl_3$ (0.75 mL) was added through the septa. The reaction was stirred (600 rpm) for 5 min at 0 °C in an ice bath. Then, under a stream of argon, xenon difluoride (14.0 mg, 0.075 mmol, 1.0 equiv.) was quickly added to the reaction mixture. *Note: xenon difluoride was stored at -40 °C in an argon-filled glovebox and weighted in a dry HPLC vial in the glovebox.* The reaction mixture was stirred for 1 h at 0 °C until completion. *Note: attempts to isolate the Bi(V) species were unsuccessful.* 4-tertbutylphenylboronic acid (16.0 mg, 0.090 mmol, 1.2 equiv.) was then added to the reaction mixture under a stream of argon. The reaction vessel was then sealed using two pieces (*ca*. 2 cm) of electrical tape, the first one covering needle punctures and the second one around the cap thread. The reaction was stirred (600 rpm) for 24 h at 90 °C in a pre-heated oil bath. After the reaction time, the crude solution was allowed to cool down to room temperature and analyzed by ^{1}H NMR, where characteristics product signals were observed.

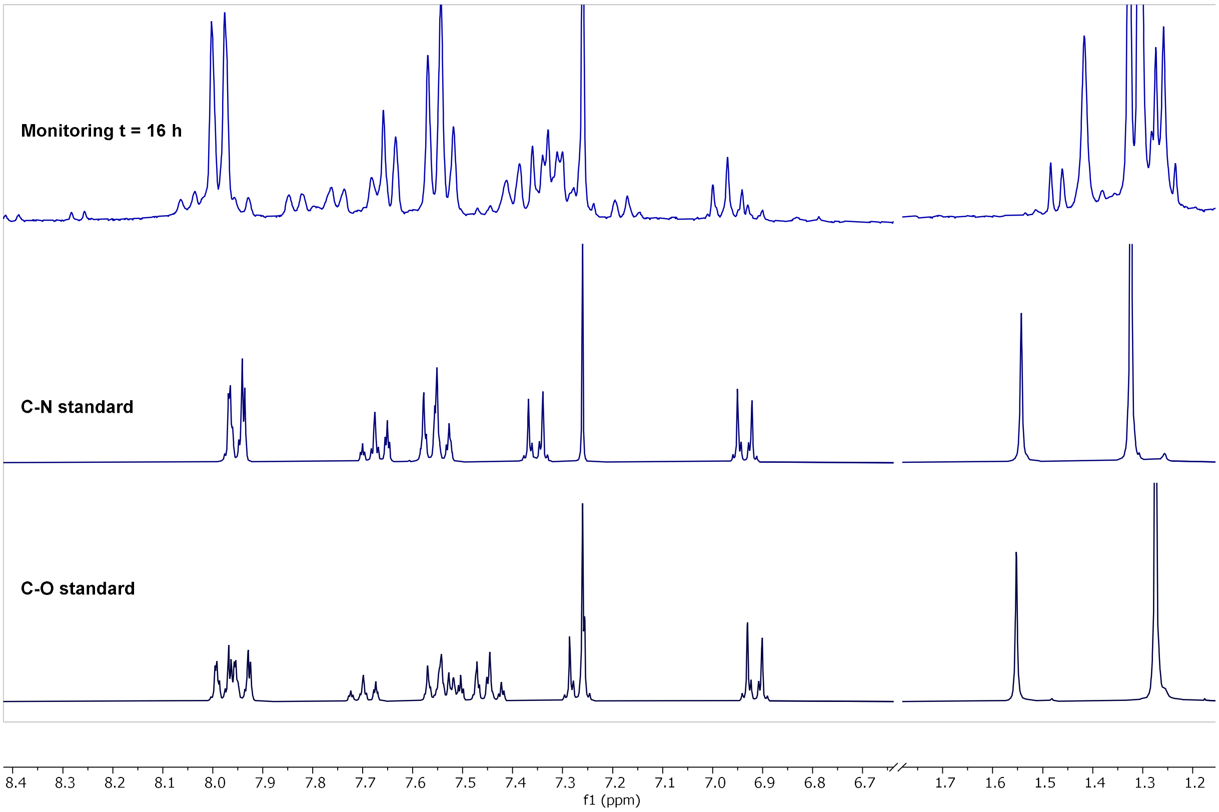

Figure S17 Stacked ^{1}H NMR ($CDCl_3$) spectra of stoichiometric reductive elimination from **7**. From top to bottom: crude reaction mixture after 16 h of heating; pure C(sp^2)–N product **2a**; pure C(sp^2)–O product **3a**.

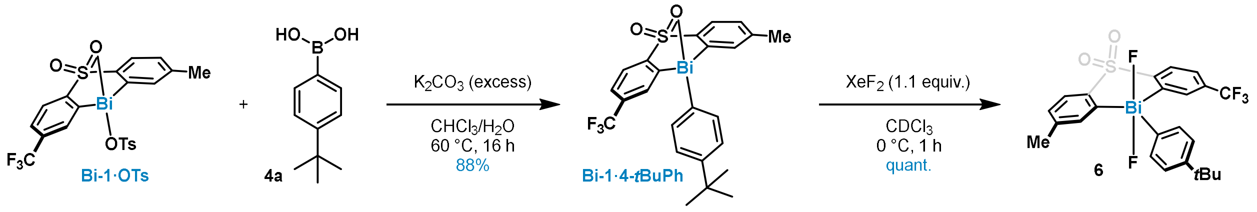

Preparation of 10-(4-(tert-butyl)phenyl)-2-methyl-8-(trifluoromethyl)-10H-dibenzo[b,e][1,4]thiabismine 5,5-dioxide (Bi-1·4-*t*BuPh)

A flame dried culture tube equipped with a Teflon-coated stir bar was charged with 2-methyl-5,5-dioxido-8-(trifluoromethyl)-10H-dibenzo[b,e][1,4]thiabismin-10-yl 4-methylbenzenesulfonate (250 mg, 0.369 mmol, 1.0 equiv.) and 4-tertbutylphenylboronic acid (79 mg, 0.443 mmol, 1.2 equiv.). The culture tube was sealed with a Teflon screw-cap, connected to an argon/vacuum Schlenk line and evacuated and refilled with argon (3 cycles). $CHCl_3$ (2.5 mL) was added through the septa followed by a K_2CO_3 saturated aqueous solution (0.25 mL). The reaction was vigorously stirred (1200 rpm) for 16 h at 60 °C in a pre-heated oil bath. Then, the reaction was cooled down to room temperature, diluted with CH_2Cl_2 (10 mL) and water (10 mL) and transferred to a separatory funnel and the organic layer was collected. The aqueous layer was extracted with CH_2Cl_2 (2 × 10 mL) and the combined organic layers were dried on $MgSO_4$, filtered and concentrated under reduced pressure to afford a light-yellow oil. The crude reaction mixture was purified by column chromatography (silica gel, CH_2Cl_2: pentane 6:4 to 10:0) to afford **Bi-1·4-*t*BuPh** (208 mg, 88% yield) as a white solid.

^{1}H NMR (600 MHz, $CDCl_3$) δ 8.43 (dt, *J* = 8.0, 0.7 Hz, 1H), 8.28 (d, *J* = 7.9 Hz, 1H), 8.01 (dt, *J* = 1.9, 0.7 Hz, 1H), 7.74 (dt, *J* = 1.7, 0.6 Hz, 1H), 7.69 – 7.67 (m, 2H), 7.66 – 7.62 (m, 1H), 7.51 – 7.46 (m, 2H), 7.22 (ddt, *J* = 7.9, 1.2, 0.6 Hz, 1H), 2.29 (s, 3H), 1.32 (s, 9H).

^{19}F NMR (282 MHz, $CDCl_3$) δ -62.97.

^{13}C NMR (151 MHz, $CDCl_3$) δ 162.9, 159.2, 158.8, 152.3, 145.7 (q, *J* = 1.6 Hz), 144.7, 138.4, 138.4, 138.1, 134.6 (q, *J* = 32.4 Hz), 134.5 (q, *J* = 3.6 Hz), 129.2, 128.3, 127.7, 126.8, 125.3 (q, *J* = 3.7 Hz), 123.4 (q, *J* = 273.6 Hz), 35.1, 31.3, 21.6.

HRMS (ESI-Orbitrap) calc'd for $C_{24}H_{23}BiO_2SF_3$ $[M+H]^+$: 641.1169, found: 641.1166.

10-(4-(tert-butyl)phenyl)-10,10-difluoro-2-methyl-8-(trifluoromethyl)-10H-10l5-dibenzo[b,e][1,4]thiabismine 5,5-dioxide (6)

A flame dried culture tube equipped with a Teflon-coated stir bar was charged with 10-(4-(tert-butyl)phenyl)-2-methyl-8-(trifluoromethyl)-10H-dibenzo[b,e][1,4]thiabismine 5,5-dioxide **Bi-1·4-*t*BuPh** (48.0 mg, 0.075 mmol, 1.0 equiv.). The culture tube was closed with a Teflon screw-cap, connected to an argon/vacuum Schlenk line and evacuated and refilled with argon (3 cycles). Anhydrous and degassed $CDCl_3$ (0.75 mL) was added through the septa. The reaction was stirred (600 rpm) for 5 min at 0 °C in an ice bath. Then, under a stream of argon, xenon difluoride (15.4 mg, 0.0825 mmol 1.1 equiv.) was quickly added to the reaction mixture. *Note: xenon difluoride was stored at -40 °C in an argon-filled glovebox and weighted in a dry HPLC vial in the glovebox.* The reaction mixture was stirred for 1 h at 0 °C until completion. The crude reaction mixture was concentrated and dried under high vacuum for one hour (pressure reading on vacuum gauge stayed at maximum 1 × 10^{-2} mbar) to afford **6** as a white solid (50.9 mg, quantitative yield) that was further use without purification.

^{1}H NMR (300 MHz, $CDCl_3$) δ 8.61 (s, 1H), 8.40 (d, *J* = 7.4 Hz, 2H), 8.22 (d, *J* = 7.9 Hz, 1H), 8.09 (d, *J* = 8.5 Hz, 2H), 7.81 (d, *J* = 8.0 Hz, 1H), 7.73 (d, *J* = 8.5 Hz, 2H), 7.41 (d, *J* = 7.9 Hz, 1H), 2.49 (s, 3H), 1.36 (s, 9H).

^{19}F NMR (282 MHz, $CDCl_3$) δ -62.9, -118.3.

Procedure for stoichiometric reductive elimination from 6

A flame dried culture tube equipped with a Teflon-coated stir bar was charged with 10-(4-(tert-butyl)phenyl)-10,10-difluoro-2-methyl-8-(trifluoromethyl)-10H-10l5-dibenzo[b,e][1,4]thiabismine 5,5-dioxide **6** (50.9 mg, 0.075 mmol, 1.0 equiv.) and sodium bis(phenylsulfonyl)amide (28.7 mg, 0.090 mmol, 1.2 equiv.). The culture tube was closed with a Teflon screw-cap, connected to an argon/vacuum Schlenk line and evacuated and refilled with argon (3 cycles). Anhydrous and degassed appropriate solvent (0.75 mL) was added through the septa followed eventually by 1,1-diphenylethylene (132 µL, 0.75 mmol, 10 equiv.). The reaction vessel was then sealed using two pieces (*ca.* 2 cm) of electrical tape, the first one covering needle punctures and the second one around the cap thread. The reaction was stirred (600 rpm) for 24 h at 90 °C in a pre-heated oil bath. After the reaction time, the crude solution was allowed to cool down to room temperature, concentrated in the culture tube at reduced pressure and dissolved in $CDCl_3$ with a known amount of CH_2Br_2 as an internal standard. Quantitative ^{1}H NMR was then recorded to determine the yield of the transformation as well as the selectivity.

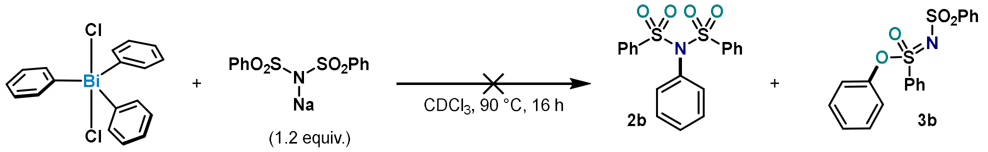

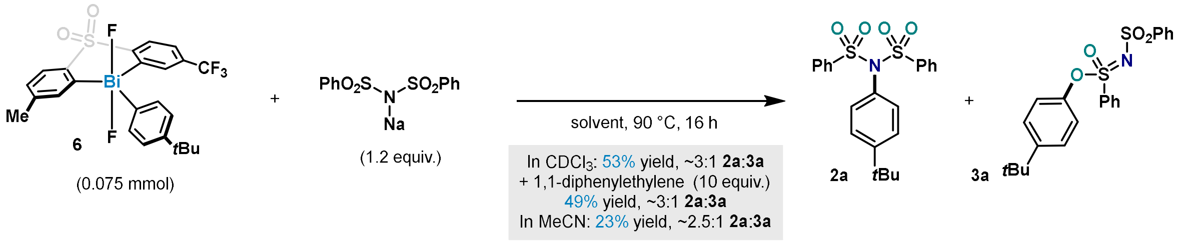

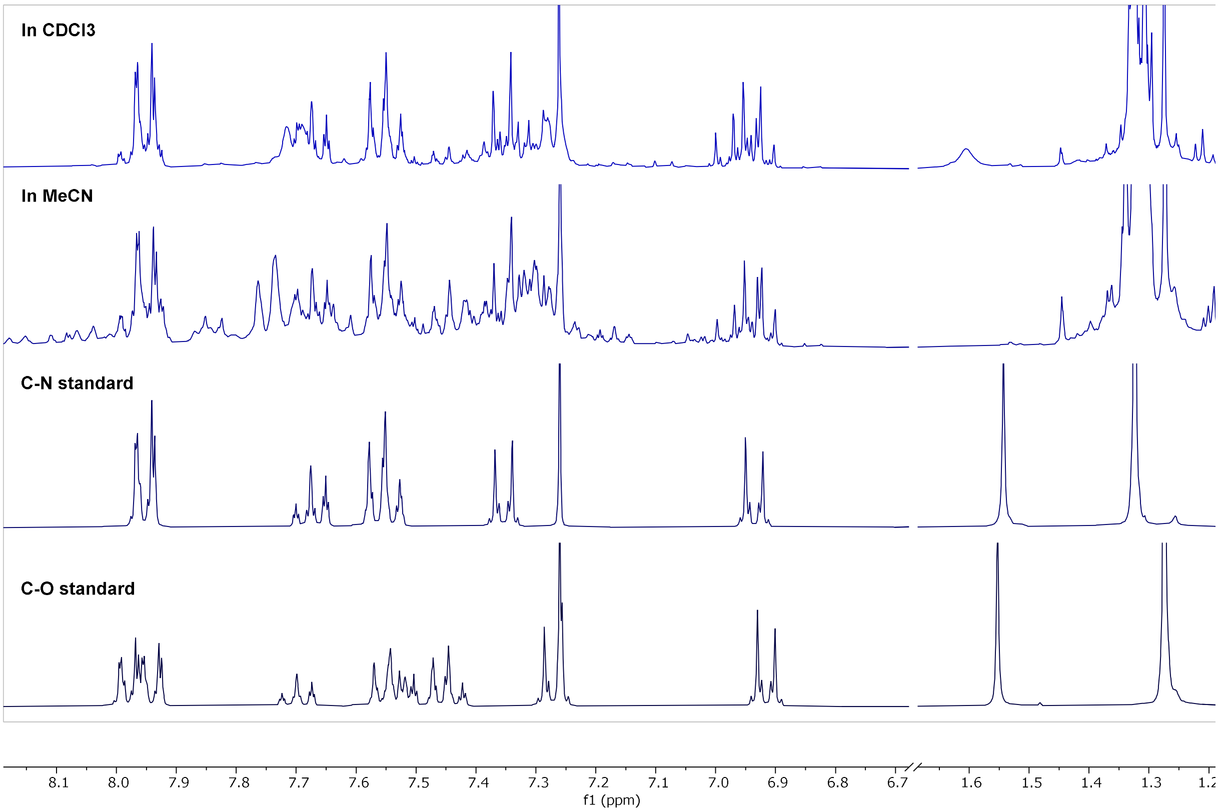

Figure S18 Stacked ^1H NMR ($CDCl_3$) spectra of stoichiometric reductive elimination from **6**. From top to bottom: crude reaction mixture after 16 h of heating in $CDCl_3$; crude reaction mixture after 16 h of heating in MeCN; pure C(sp²)–N product **2a**; pure C(sp²)–O product **3a**.

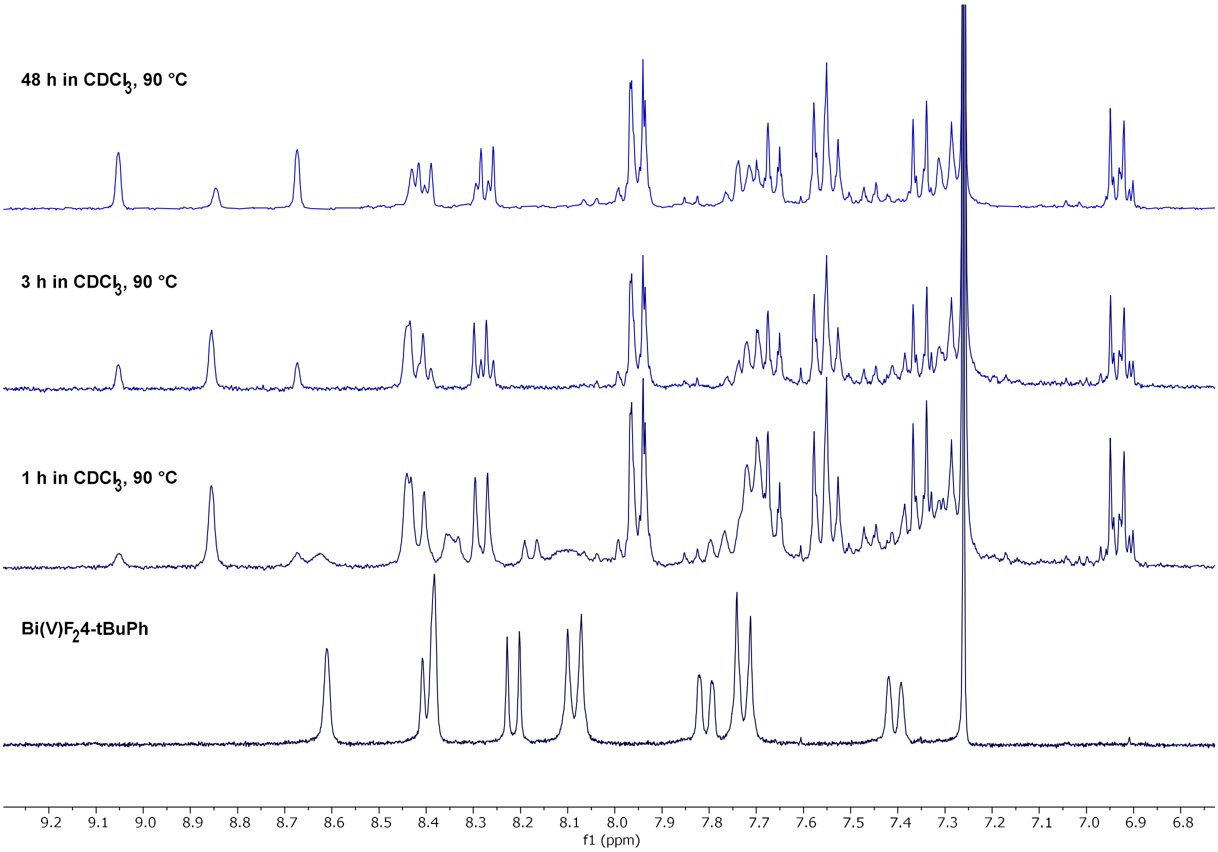

Figure S19 Stacked ^1H NMR ($CDCl_3$) spectra of stoichiometric reductive elimination from **6** after different time of reaction. Consumption of **6** and formation of product and Bi(III)-X by product. From top to bottom: crude reaction mixture after 48 h of heating; crude reaction mixture after 3 h of heating; crude reaction mixture after 1 h of heating; pure **6**.

9.7. Other reaction pathways

Generation of Aryl radical by oxidation of Bi(III) to Bi (IV)[9]

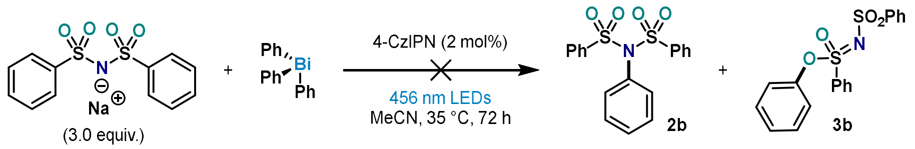

A flame dried culture tube equipped with a Teflon-coated stir bar was charged with triphenyl bismuth (220 mg, 0.50 mmol, 1.0 equiv.), sodium bis(phenylsulfonyl)amide (479 mg, 1.50 mmol, 3.0 equiv.) and 4-CzIPN (7.9 mg, 0.01 mmol, 2 mol%). The culture tube was closed with a Teflon screw-cap, connected to an argon/vacuum Schlenk line and evacuated and refilled with argon (3 cycles). Anhydrous and degassed acetonitrile (5 mL) was added through the septa. The reaction vessel was then sealed using two pieces (*ca.* 2 cm) of electrical tape, the first one covering needle punctures and the second one around the cap thread. The reaction was stirred (600 rpm) for 72 h under blue LEDs irradiation (Kessil 456 nm) and cooled with fans at 35 °C. *Note: apparition of large amount of black solid was observed.* After the reaction time, the crude solution was allowed to cool down to room temperature, concentrated

in the culture tube at reduced pressure and dissolved in $CDCl_3$ with a known amount of CH_2Br_2 as an internal standard. Quantitative ^{1}H NMR was then recorded to determine the yield of the transformation.

Generation of N-centered radical by oxidation with TEMPO[10] followed by ipso substitution[11]

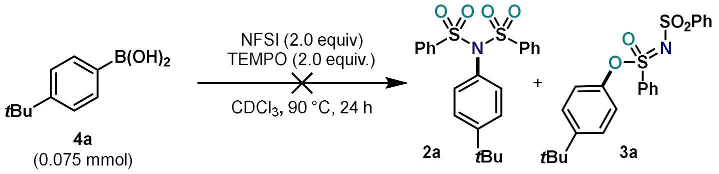

A flame dried culture tube equipped with a Teflon-coated stir bar was charged with 4-tertbutylphenylboronic acid **4a** (13.4 mg, 0.075 mmol, 1.0 equiv.), N-fluorosulfonimide (47.3 mg, 0.15 mmol, 2.0 equiv.) and TEMPO (23.4 mg, 0.15 mmol, 2.0 equiv.). The culture tube was closed with a Teflon screw-cap, connected to an argon/vacuum Schlenk line and evacuated and refilled with argon (3 cycles). Anhydrous and degassed $CDCl_3$ (0.75 mL) was added through the septa. The reaction vessel was then sealed using two pieces (*ca.* 2 cm) of electrical tape, the first one covering needle punctures and the second one around the cap thread. The reaction was stirred (600 rpm) for 16 h at 90 °C in a pre-heated oil bath. After the reaction time, the crude solution was allowed to cool down to room temperature, concentrated in the culture tube at reduced pressure and dissolved in $CDCl_3$ with a known amount of CH_2Br_2 as an internal standard. Quantitative ^{1}H NMR was then recorded to determine the yield of the transformation.

Disproportionation of Bismuth species[12]

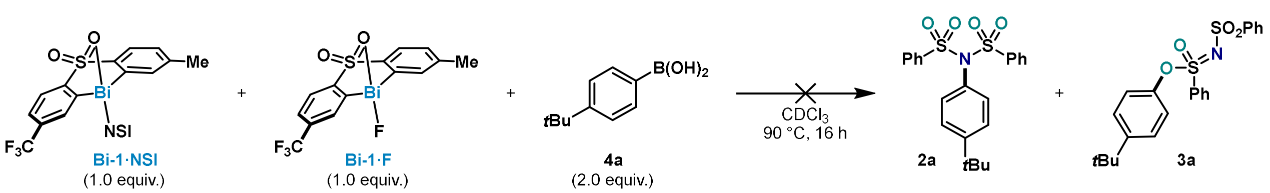

A flame dried culture tube equipped with a Teflon-coated stir bar was charged with 4-tertbutylphenylboronic acid **4a** (6.6 mg, 0.037 mmol, 2.0 equiv.), N-(2-methyl-5,5-dioxido-8-(trifluoromethyl)-10H-dibenzo[b,e][1,4]thiabismin-10-yl)-N-(phenylsulfonyl)benzenesulfonamide **Bi-1·NSI** (15.0 mg, 0.019 mmol, 1.0 equiv.) and 10-fluoro-2-methyl-8-(trifluoromethyl)-10H-dibenzo[b,e][1,4]thiabismine 5,5-dioxide **Bi-1·F** (9.8 mg, 0.019 mmol, 1.0 equiv.). The culture tube was closed with a Teflon screw-cap, connected to an argon/vacuum Schlenk line and evacuated and refilled with argon (3 cycles). Anhydrous and degassed $CDCl_3$ (0.30 mL) was added through the septa. The reaction vessel was then sealed using two pieces (*ca.* 2 cm) of electrical tape, the first one covering needle punctures and the second one around the cap thread. The reaction was stirred (600 rpm) for 16 h at 90 °C in a pre-heated oil bath. After the reaction time, the crude solution was allowed to cool down to room temperature, concentrated in the culture tube at reduced pressure and dissolved in $CDCl_3$ with a known amount of CH_2Br_2 as an internal standard. Quantitative ^{1}H NMR was then recorded to determine the yield of the transformation.

10. Electrochemical data

Cyclic voltammograms were collected using a 3-electrode cell consisting of a 1.6 mm Ø glassy carbon working electrode, a platinum wire counter electrode, and a bare silver wire as a pseudoreference electrode at ambient temperature in an argon-filled glovebox equipped with electrochemical outlets. Sublimed ferrocene was added as the internal reference.

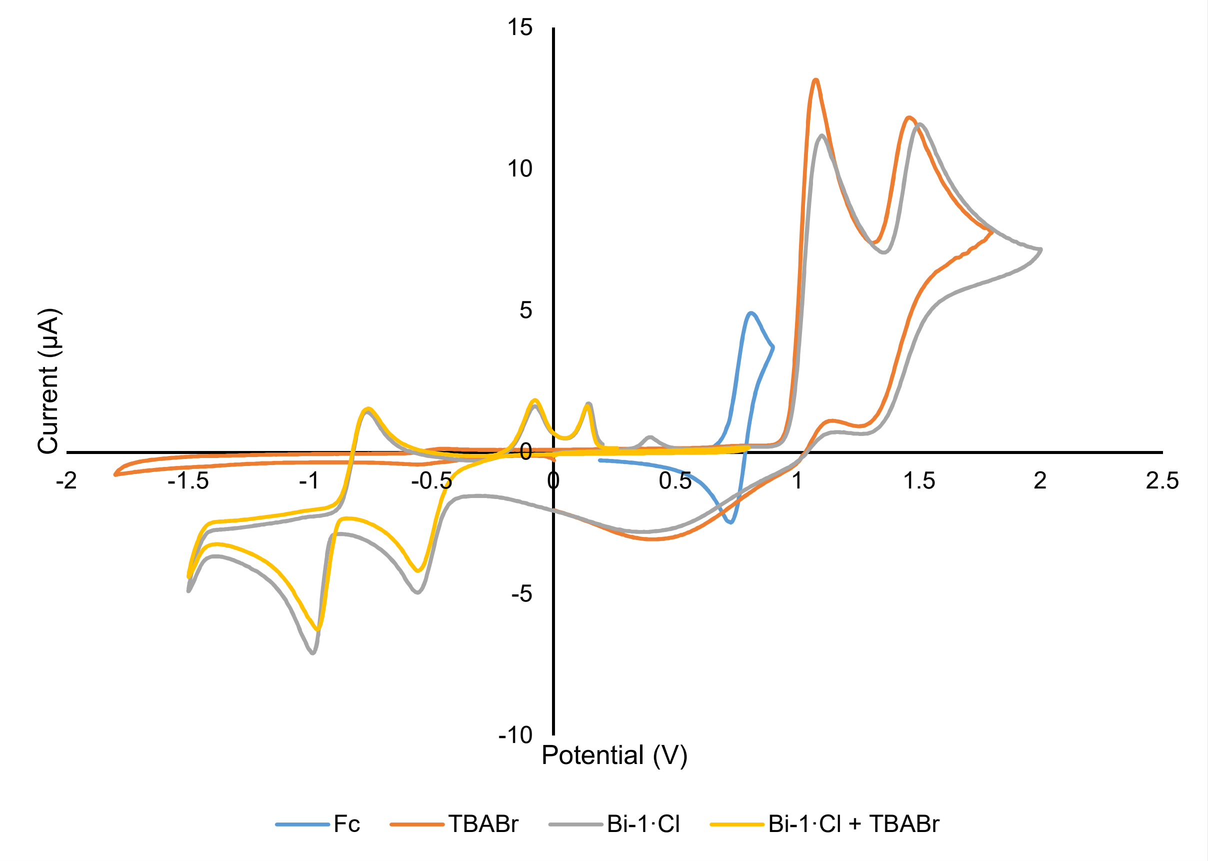

Figure S20 Cyclic voltammogram of a 1 mM solution of **Bi-1·Cl** with 1 equiv. of TBABr in CH_3CN using 0.1 M [^{n}Bu_4N][PF_6] as the supporting electrolyte at ambient temperature; scan rate: 100 mV/s.

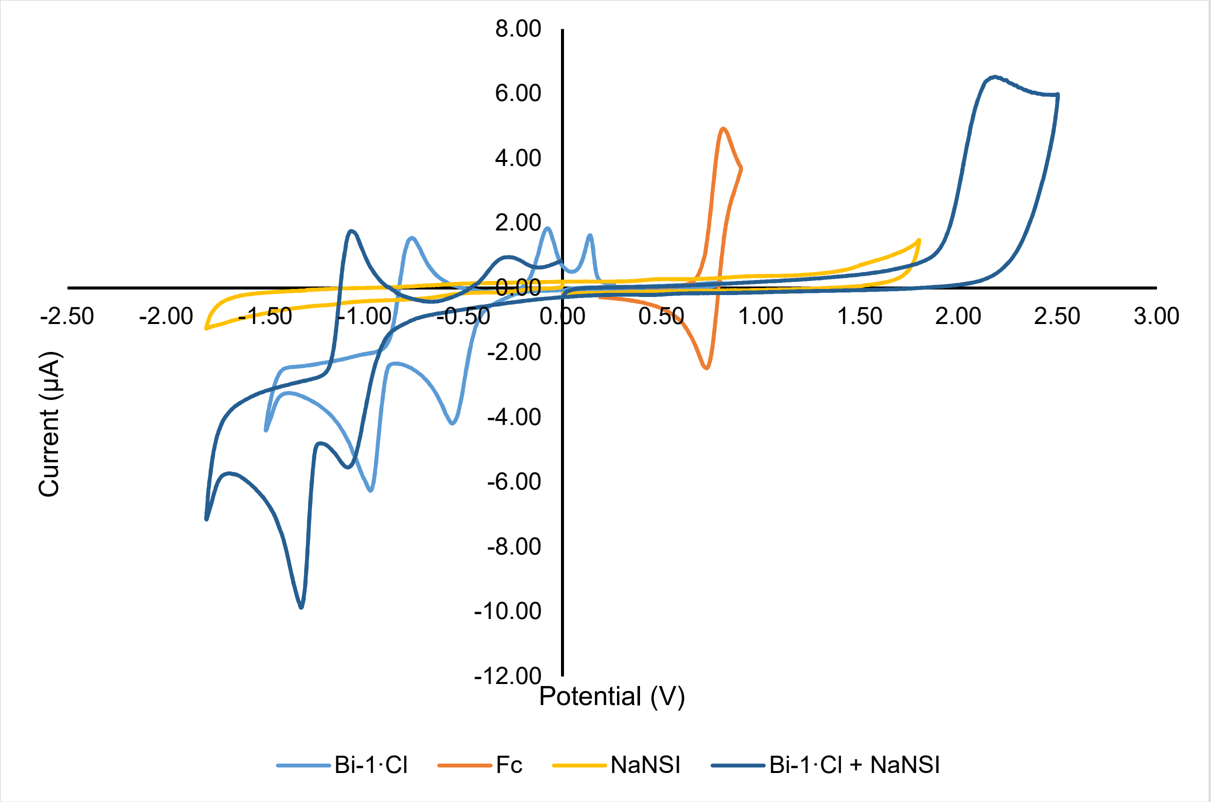

Figure S21 Cyclic voltammogram of a 1 mM solution of **Bi-1·Cl** with 1 equiv. of NaNSI in CH_3CN using 0.1 M [^{n}Bu$_4$N][PF$_6$] as the supporting electrolyte at ambient temperature; scan rate: 100 mV/s.

11. Computational Chemistry

11.1. Computational Details

Geometry optimizations and Hessian calculations were carried out with the TURBOMOLE[13] program (version 7.5.2) using the GGA BP86[14] in combination with the def2-TZVP[15] basis set and the D4 dispersion correction.[16] For all calculations, the standard effective core potential (ECP) was used for bismuth atoms.[17] These calculations were performed with the COSMO solvation model (default parameters) with an infinite dielectric constant.[18] At the same level of theory, stationary points were verified via analysis of the vibrational frequencies. Final electronic single point energies were obtained using the B3LYP[19] functional along with the def2-TZVP[15] basis set and also the D4 dispersion correction.[16] The resolution-of-identity (RI) approximation[20] and corresponding auxiliary basis sets were applied for all calculations.

For the computation of Gibbs free energies, zero-point vibrational energies and thermodynamic corrections were obtained at the level of geometry optimization (at T = 363.15 K and p = 1 bar). The solvation contribution was calculated using the COSMO-RS[21] model in the COSMOtherm[22] program (Version 18.0.0; Revision 4360) for infinite dilution in chloroform employing the FINE parametrization with a reference state of 1 mol/l at the reaction temperature of 363.15 K. Connections between transition states and minima were verified by displacing the transition state geometry along the transition mode followed by geometry optimization or IRC calculations. IRC calculations were done using the IRC code in the Gaussian16[23] software package, however utilizing energies, gradients and force constants from TURBOMOLE as external program. For barrierless reactions, a potential energy scan was performed. Pictures of molecular structures were generated with Cylview[24] and Chemcraft[25].

For the exploration of reaction paths, the MGSM[26] code in combination with a precomplex builder was used.[27] Excessive conformational screening for all transition states and intermediates was performed using the CREST[28] (version 2.11.2) program package developed by Grimme et. al followed by DFT optimization and single point calculation of selected structures for a ranking of the free energies on single point level. For simplification, only the lowest conformers were considered.

11.2. Feature Calculation

For the calculation of properties, ORCA[29] (version 5.0.4) singlepoints were performed with the B3LYP[19] functional and the def2-TZVP[15] basis set on the lowest energy conformer. Implicit solvation treatment was done with CPCM[30] using chloroform as solvent. From these singlepoint calculations, NMR-shifts and HOMO-LUMO parameters were collected. MORFEUS[31] was used to collect buried volume and Sterimol values. Wiberg bond indices and NPA charges were collected at the level of geometry optimization from the TURBOMOLE[13] calculation from the Natural Population Analysis.[32] Hammett parameters were taken from the literature for the arene substituents.[33] Coordination isomers were manually screened for selected examples, afterwards a conformational screening on the same coordination isomer was performed for all complexes in an automated fashion. On the initial conformer ensemble obtained from CREST, DFT geometry optimizations followed by singlepoint calculations were performed on 10-20 conformers, for a ranking in free energy. The calculations of descriptors were only performed for the lowest conformer. It is noted that since this automated workflow was also performed for the species shown in the mechanistical analysis (Bi-4-OTFA, Bi-5-OTs, Bi-2-Cl), this resulted in slightly different – but within the error range identical − conformers for the statistical modeling compared to the mechanism calculations. For properties from the arene ligand, an average for the atoms of both sides (C^1-C^6 and $C^{1'}$-$C^{6'}$), as well as the maximum and minimum value were considered for the dataset (av-, min-, max-). Molecular descriptors were collected using an in-house developed Python script.[34] A general atom labeling was applied for the nomenclature of the descriptors. Based on RDKIT[35] substructure search, atom mapping was performed. The final mapping of atom numbers to atom names is given in Figures S22 and in the xlsx file.

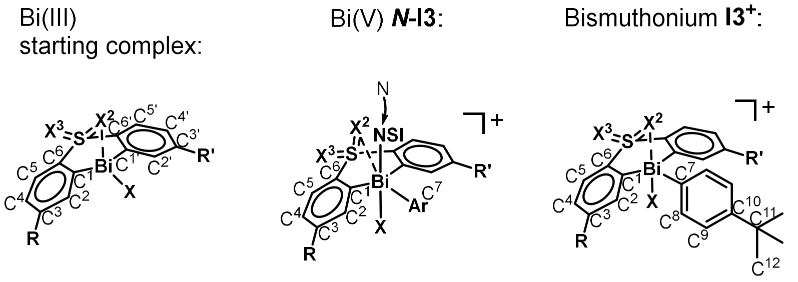

Figure S22 General atom labeling in the bismuthonium cation. The positions C^1-C^6, C^8, C^9, C^{12} were treated with an average, minimum and maximum value of the descriptors at of both (or in the case of C12 all three) atoms.

11.3. Statistical Modeling

The experimental selectivity (C−N:C−O) was regressed. However, to ensure linear dependency on a difference in free activation energy the form $\Delta\Delta G^{\ddagger} = -RTln(Selectivity)$ was used (T=363 K). Since different solvents were used, the modeling process was only performed for those data collecting using chloroform as the solvent. Two data points were considered as structural outliers as they fall outside of the distribution of the other data points.

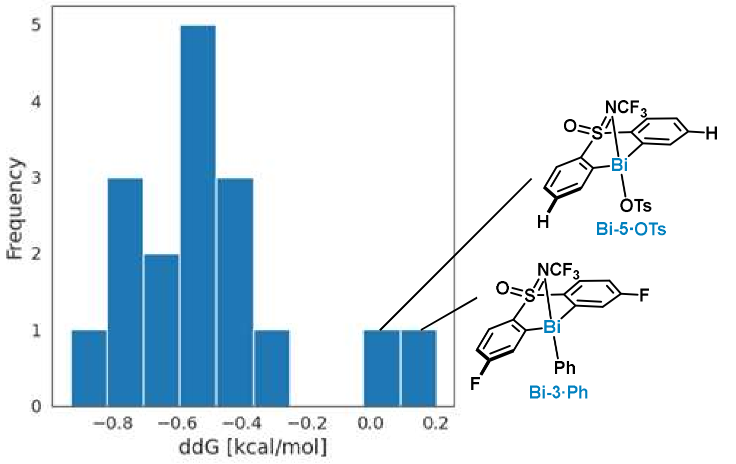

Figure S23 Data distribution; 2 data points are defined as outliers, these are the two sulfoximine species Bi-3·Ph and Bi-5·OTs.

Multivariate linear regression (MLR) modeling was performed using an in-house python script,[36] which uses scikit-learn.[37] The collected features for all complexes (see Computational Details for insights into the feature calculation) are provided as an .xlsx file. A collinearity filter for the features was applied with a cutoff at R^2=0.5 to remove correlated features from the same model. Furthermore, the features were normalized for direct interpretability of the model's coefficients. A brute force feature selection was used in the model search, building and comparing all possible models with one or two features.

To evaluate the model's performance, the data was divided into a training and a validation set. An external test set was not applied because of the too small data set size. The splitting was performed applying a 12:3 train:validation split. The validation points were chosen to be evenly (equidistant) distributed throughout the output variable, but not extrapolating. The cross-validation technique leave-one-out (LOO) was used to evaluate the robustness of the models and to test for overfitting. For both

data sets and the cross-validation, a R^2 (namely Q^2 in the case of LOO) and MAE were calculated. A good linear correlation is defined with a R^2 value near 1 and a low MAE in all cases.

After building all possible models, models were filtered and selected based on the statistical measures (R^2 values, MAEs, Q^2) and descriptor interpretability. Primarily, the product of the training R^2 and Q^2 was utilized for model selection. It is noted that the goal of the linear models in this project is not predictivity, but interpretability and reproducing the experimental data points to investigate what effects influence chemoselectivity.

Table S9 Experimental Selectivities Sel(C−N:C−O), natural log ln(Sel), and ΔΔG(ΔG‡N−ΔG‡O) obtained via $\Delta\Delta G = -R \cdot T \cdot \ln(\mathrm{Sel})$; as well as the information whether the point is in the training or validation set or considered as outlier; *= for Bi-1·OTFA a slightly different N:O selectivity is used than in the experimental section, due to the mean-value being based on less experiments, both values are in the error range similar (2.05 vs 2.11).

	Sel (C−N:C−O)	ln(Sel)	$\Delta\Delta G^{exp}$ kcal/mol	Train./Valid. or Outlier
Bi-1·BF_4	1.93	0.66	-0.47	Train.
Bi-3·Ph	0.76	-0.27	0.20	Outlier
Bi-5·OTs	1.03	0.03	-0.02	Outlier
Bi-2·OTFA	2.82	1.04	-0.75	Train.
Bi-4·OTFA	2.13	0.76	-0.55	Train.
Bi-13·OTFA	2.11	0.75	-0.54	Train.
Bi-9·OTFA	1.91	0.65	-0.47	Train.
Bi-6·OTFA	1.58	0.46	-0.33	Train.
Bi-1·OTFA	2.05*	0.72	-0.52	Train.
Bi-7·OTFA	2.74	1.01	-0.73	Train.
Bi-8·OTFA	1.84	0.61	-0.44	Valid.
Bi-2·Cl	3.65	1.29	-0.93	Train.
Bi-1·Cl	3.00	1.10	-0.79	Valid.
Bi-1·Br	2.22	0.80	-0.58	Train.
Bi-1·Ph	2.48	0.91	-0.66	Train.
Bi-1·OTs	1.97	0.68	-0.49	Train.
Bi-1·NSI	2.35	0.85	-0.62	Valid.

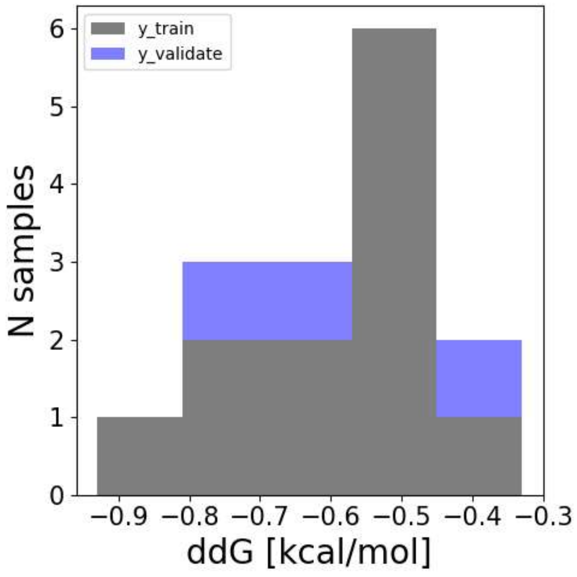

Figure S24 0.2 Train/Validation Splitting.

Different MLR models and selection of the best model

Initially, the Bi(III) resting states of the catalyst were used for statistical modeling. However, when evaluating the various statistical measures (see chapter 11.3 for more information about model selection), no convincing model was found. The most promising model is shown in Figure S25. Two steric parameters are used in this model. No other model with decent statistical measures was found for the Bi(III) resting state. The statistical measures are worse than for the models for the Bi(V) and the bismuthonium complexes.

The model for the Bi(V) intermediate shows better statistical measures (Figure S27). However, both features are only classifiers for the ligand substitution (Hammett σ_meta value) and the counter ion (Sterimol parameter), thus effectively one-hot encoding the catalysts. The linear model based on the bismuthonium (Figure S31) shows comparable statistical measures. However, it has only one clearly classifying feature (the WBI) and was thus the preferred model. Furthermore, the tendency of competitive and changing pathways is more readily interpretable with the bismuthonium model, because the NPA charge at the oxygen directly correlates to a possible stabilization of a bismuthonium intermediate or a 3-membered transition state structurally close to a bismuthonium. It is noted that while the shown models are the best models which were found, these are only representative.

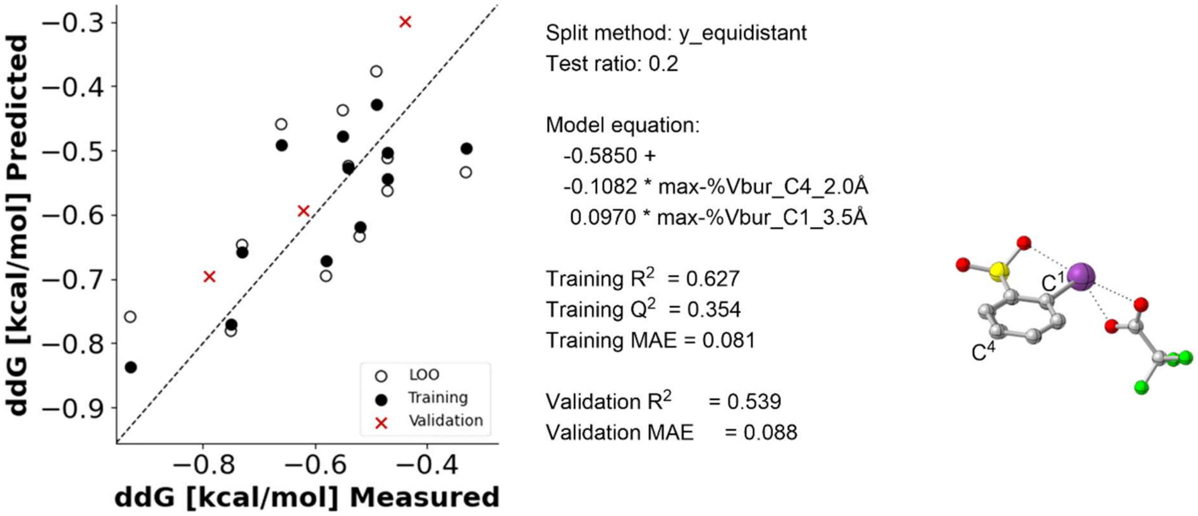

Figure S25 Best multivariate linear regression model for the Bi(III) starting complexes.

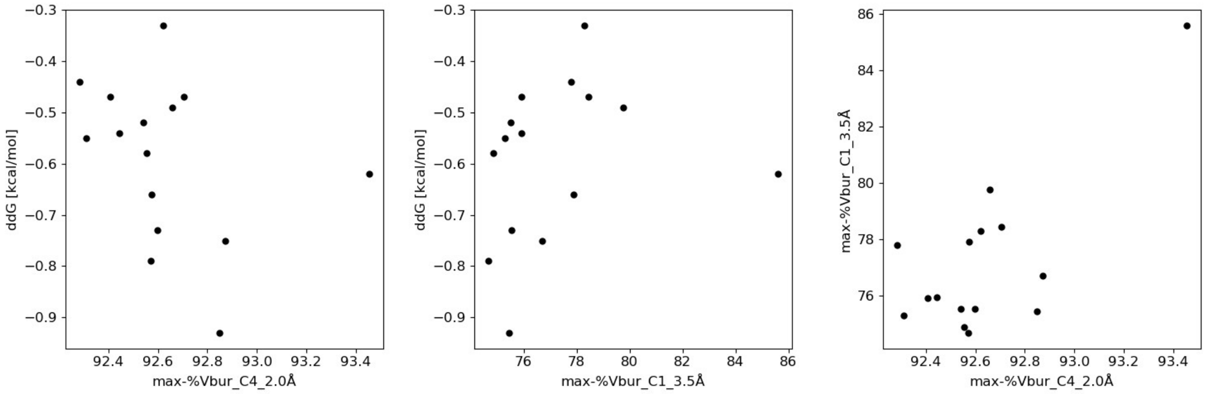

Figure S26 ΔΔG versus feature as well as feature versus feature plots for the best multivariate linear regression model for the Bi(III) starting complexes; for this plot unscaled features were used.

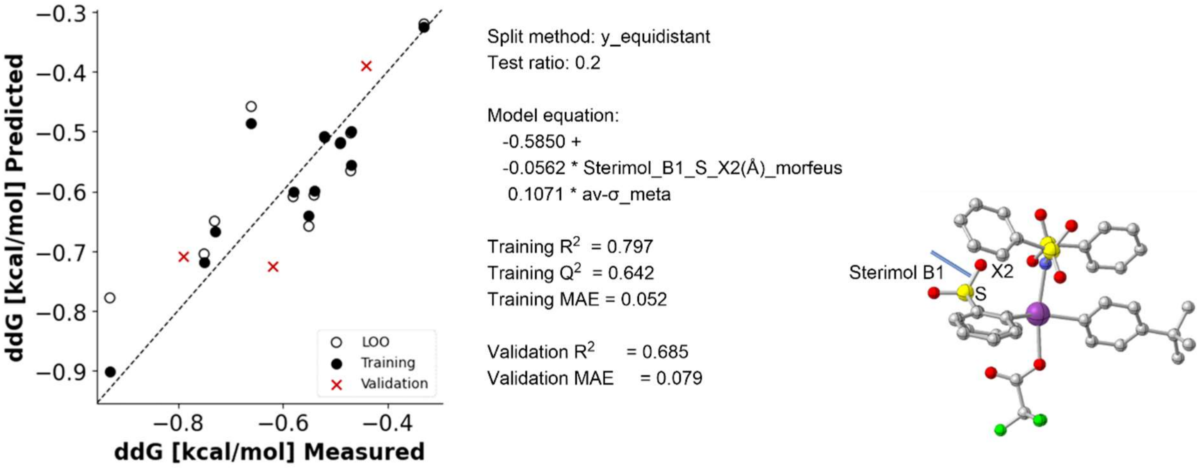

Figure S27 Best multivariate linear regression model for the Bi(V) intermediate ***N*-I3**.

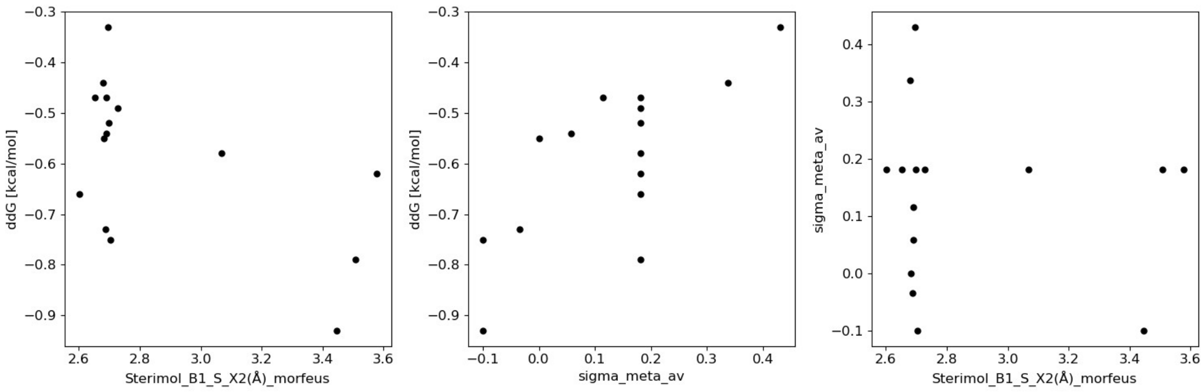

Figure S28 ΔΔG versus feature as well as feature versus feature plots for the best multivariate linear regression model for the Bi(V) intermediate ***N*-I3**; for this plot unscaled features were used.

The Sterimol parameter is difficult to interpret. To investigate what the parameter describes, it was regressed against all other descriptors in the library. A good correlation was found for two buried volume terms at the sulfur and the C^1 atom, which is coordinated to the bismuth center. With this, the Sterimol parameter mainly classifies the counter ion:

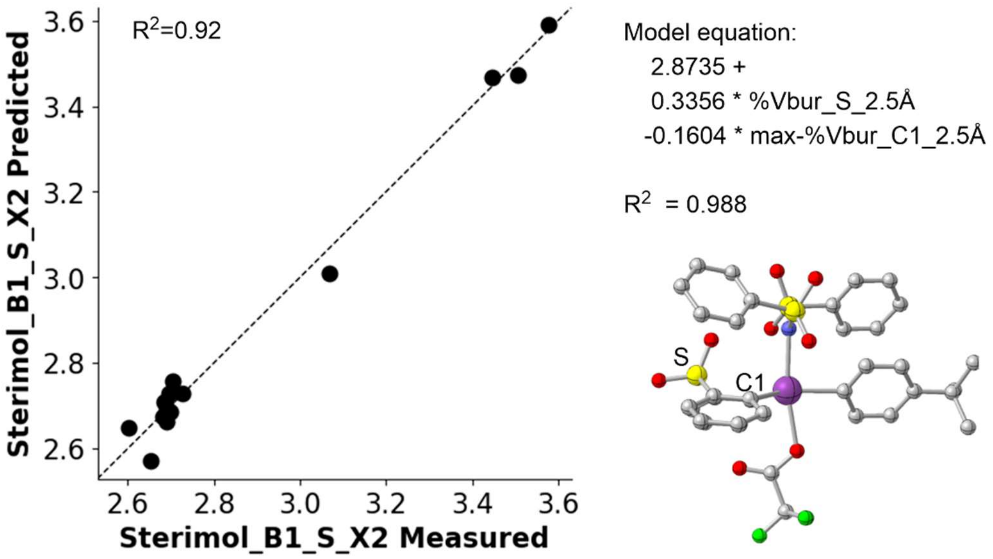

Figure S29 MLR model to regress the Sterimol parameter used in the model based on the Bi(V) complexes ***N*-I3**.

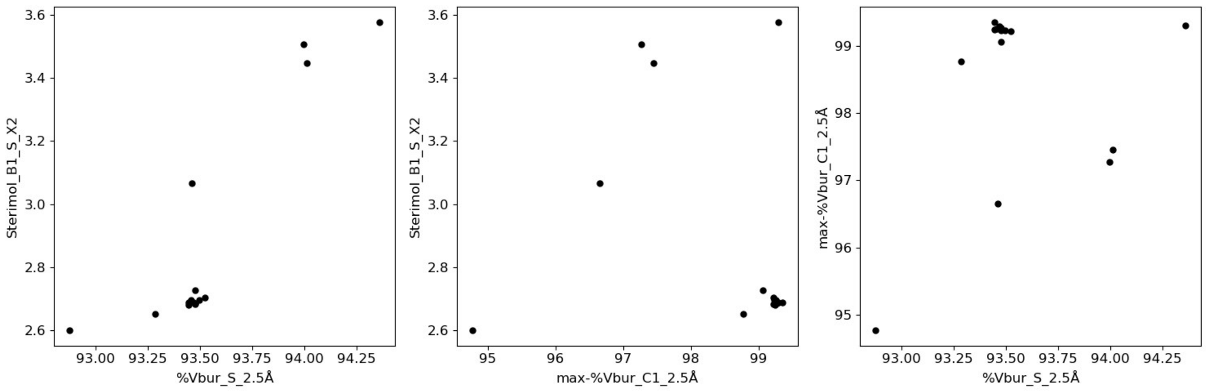

Figure S30 ΔΔG versus feature as well as feature versus feature plots for the best multivariate linear regression model for the regression of the Sterimol_B1_S_X2 parameter; for this plot unscaled features were used.

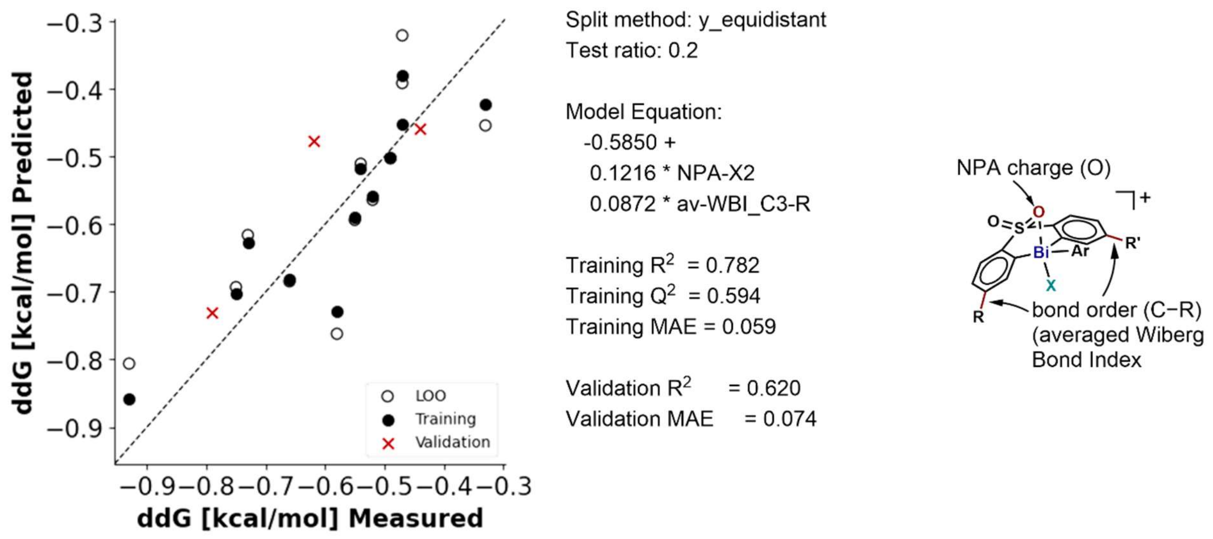

Figure S31 Best multivariate linear regression model for the bismuthonium complexes **I3**$^+$.

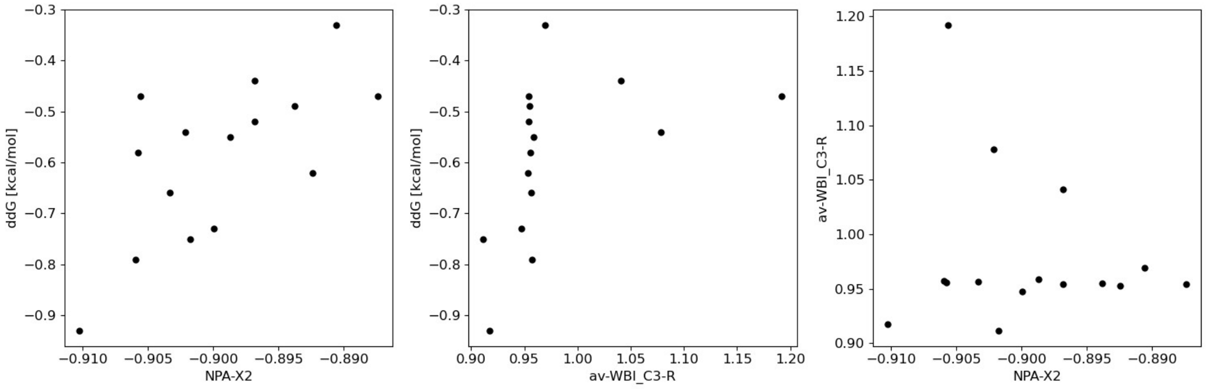

Figure S32 ΔΔG versus feature as well as feature versus feature plots for the best multivariate linear regression model for the bismuthonium complexes **I3**$^+$; for this plot unscaled features were used.

To further understand what the Wiberg bond index describes, it was also regressed against other molecular descriptors. This regression revealed a correlation with two Sterimol parameters, the width (Sterimol L for C^2–C^3) and length (Sterimol L for R–C^3) of the substituent at the *meta*-position, suggesting the sterics about the bismuth atom is read out.

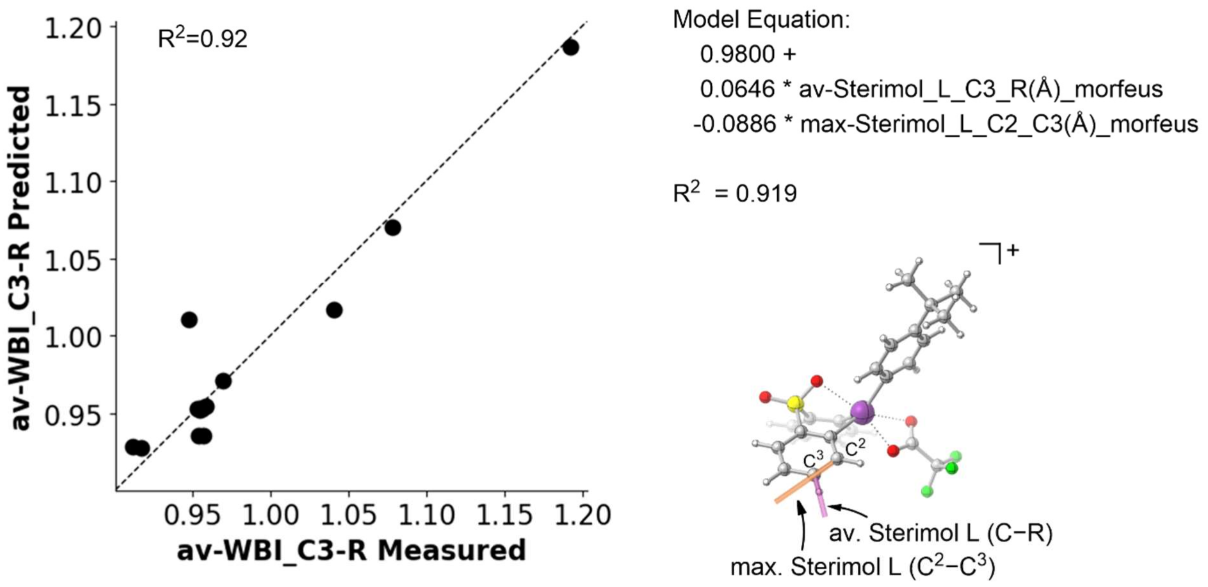

Figure S33 MLR model to regress the Wiberg bond index used in the model based on the bismuthonium complexes **I3**$^+$.

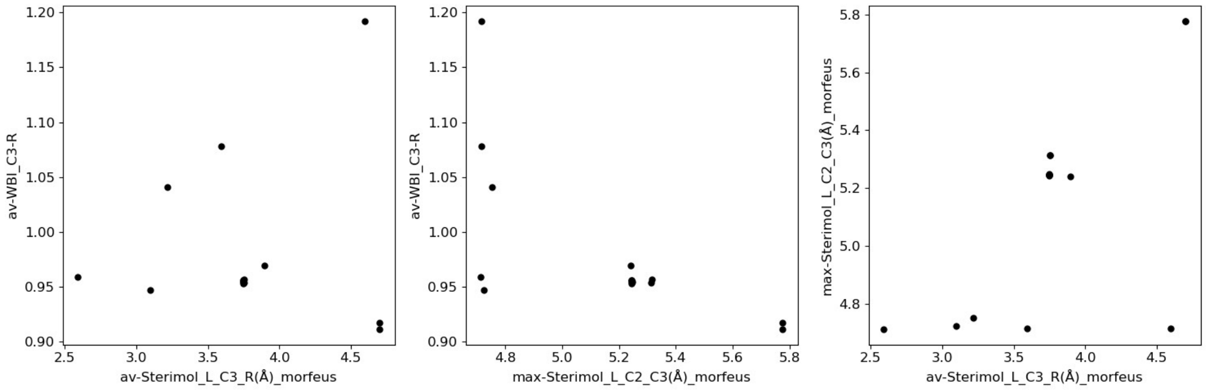

Figure S34 ΔΔG versus feature as well as feature versus feature plots for the regression of the Wiberg bond index; for this plot unscaled features were used.

The sulfoximine-containing catalysts were not used for the statistical modeling, since they are outliers in the histogram plot (Figure S23). Using these two catalysts as test points in the final MLR model trained on the sulfone-containing bismuthonium complexes failed (Figure S35):

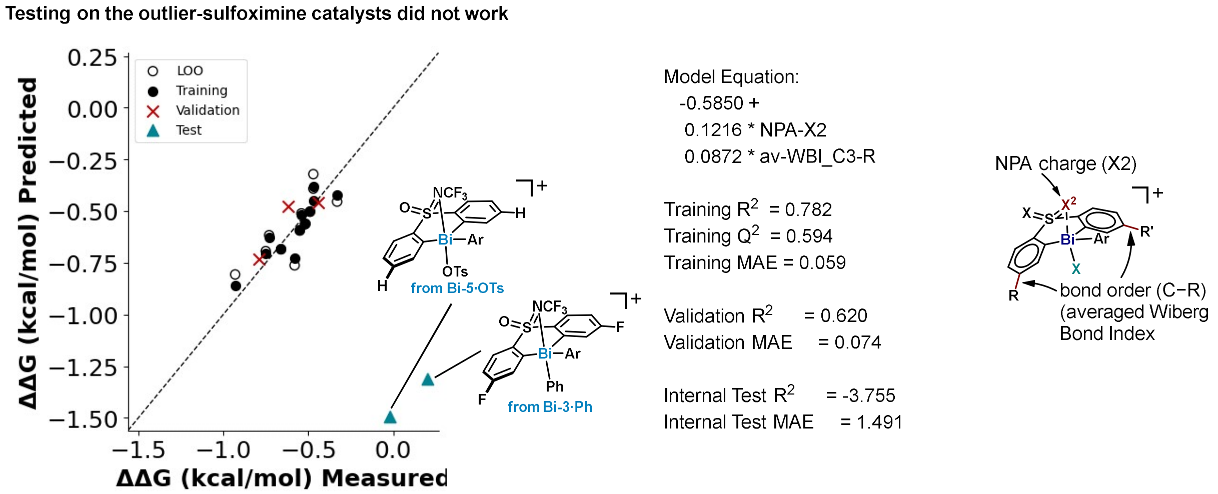

Figure S35 Using the sulfoximine-containing catalysts as test points in the MLR model trained on the sulfone-containing bismuthonium complexes (Figure S31) failed.

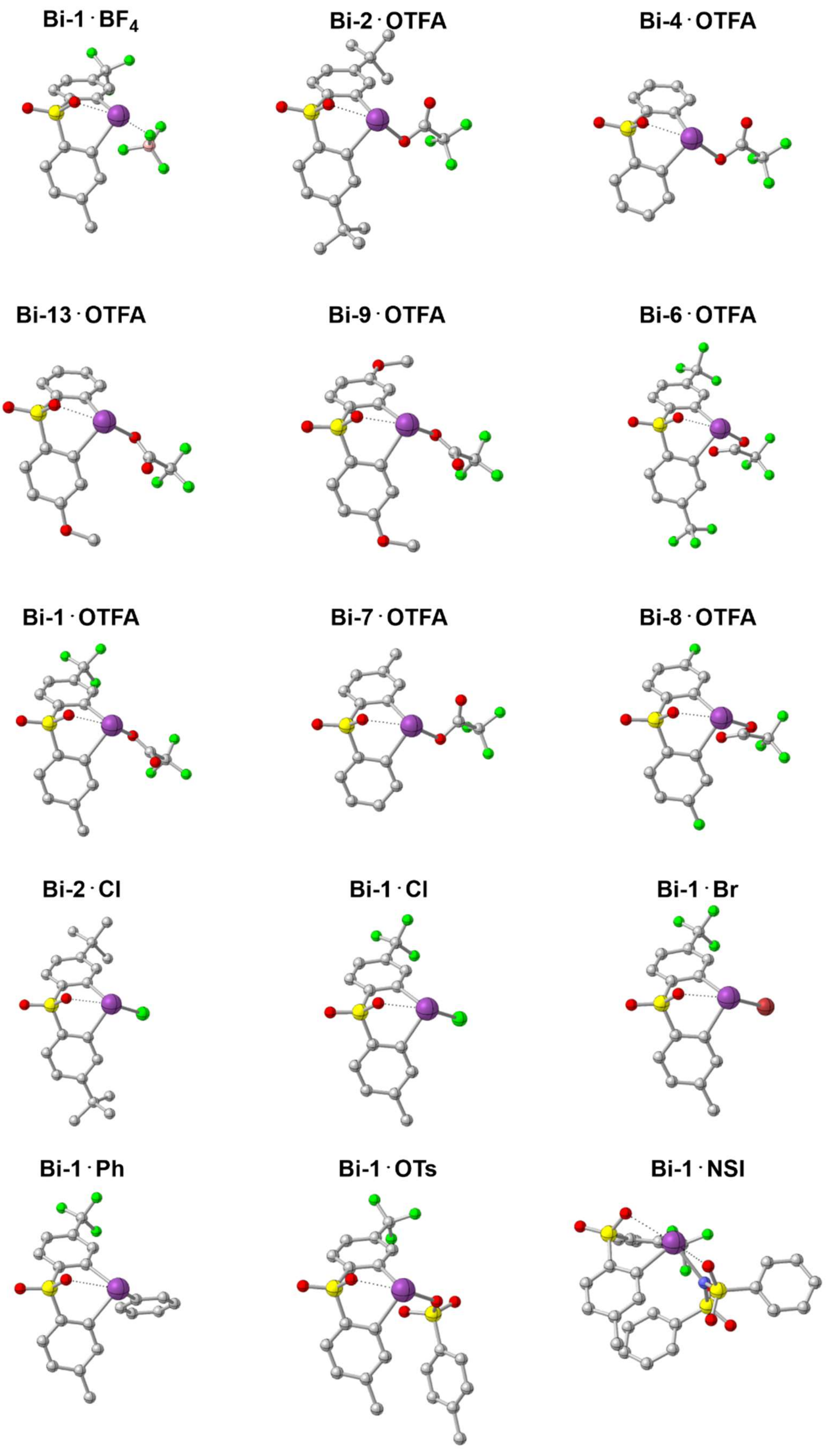

Figure S36 Optimized Structures of all Bi(III) starting complexes; cosmo(∞)-BP86-D4/def2-TZVP.

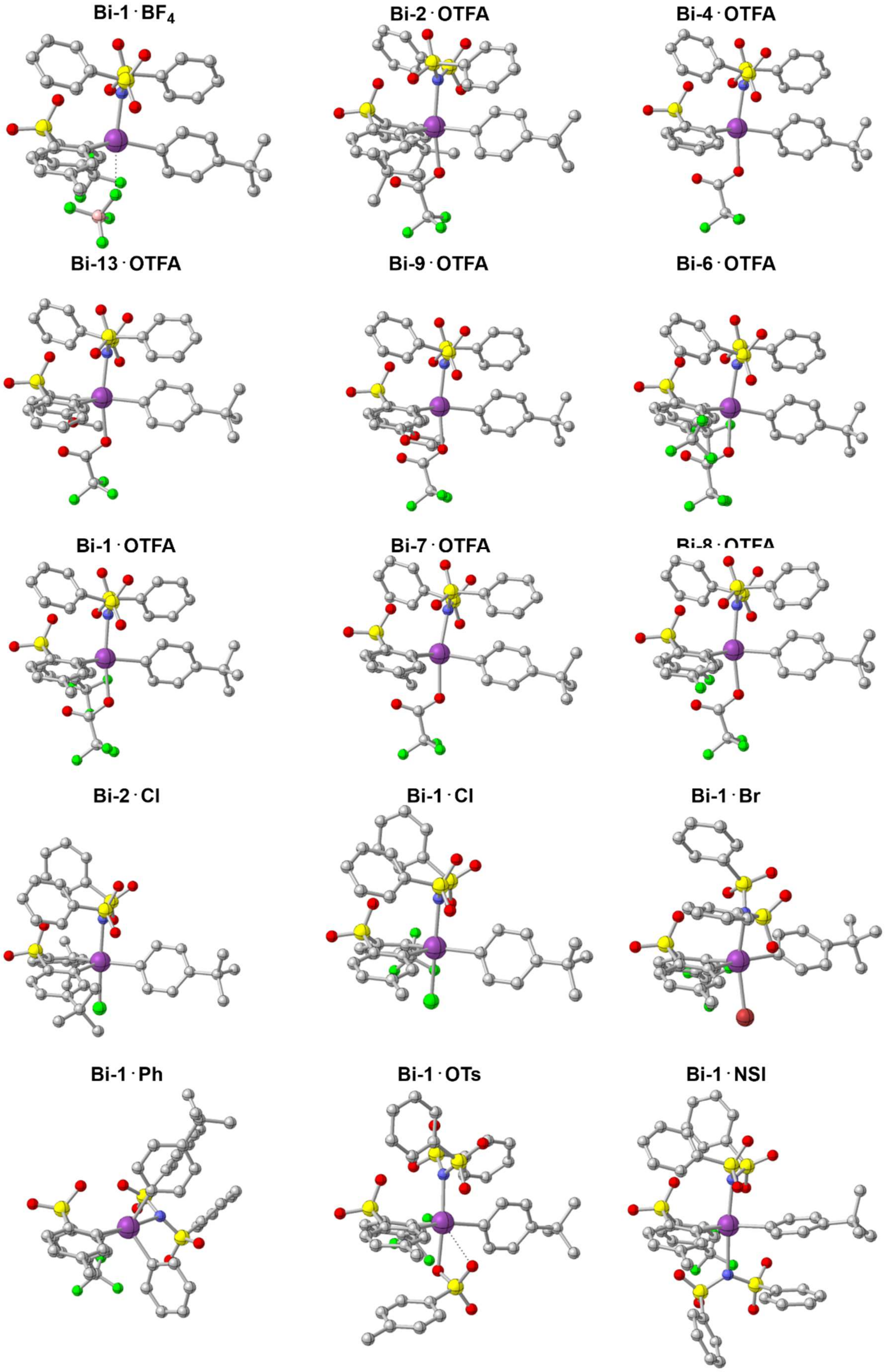

Figure S37 Optimized Structures of all *N*-bound Bi(V) intermediates; cosmo(∞)-BP86-D4/def2-TZVP.

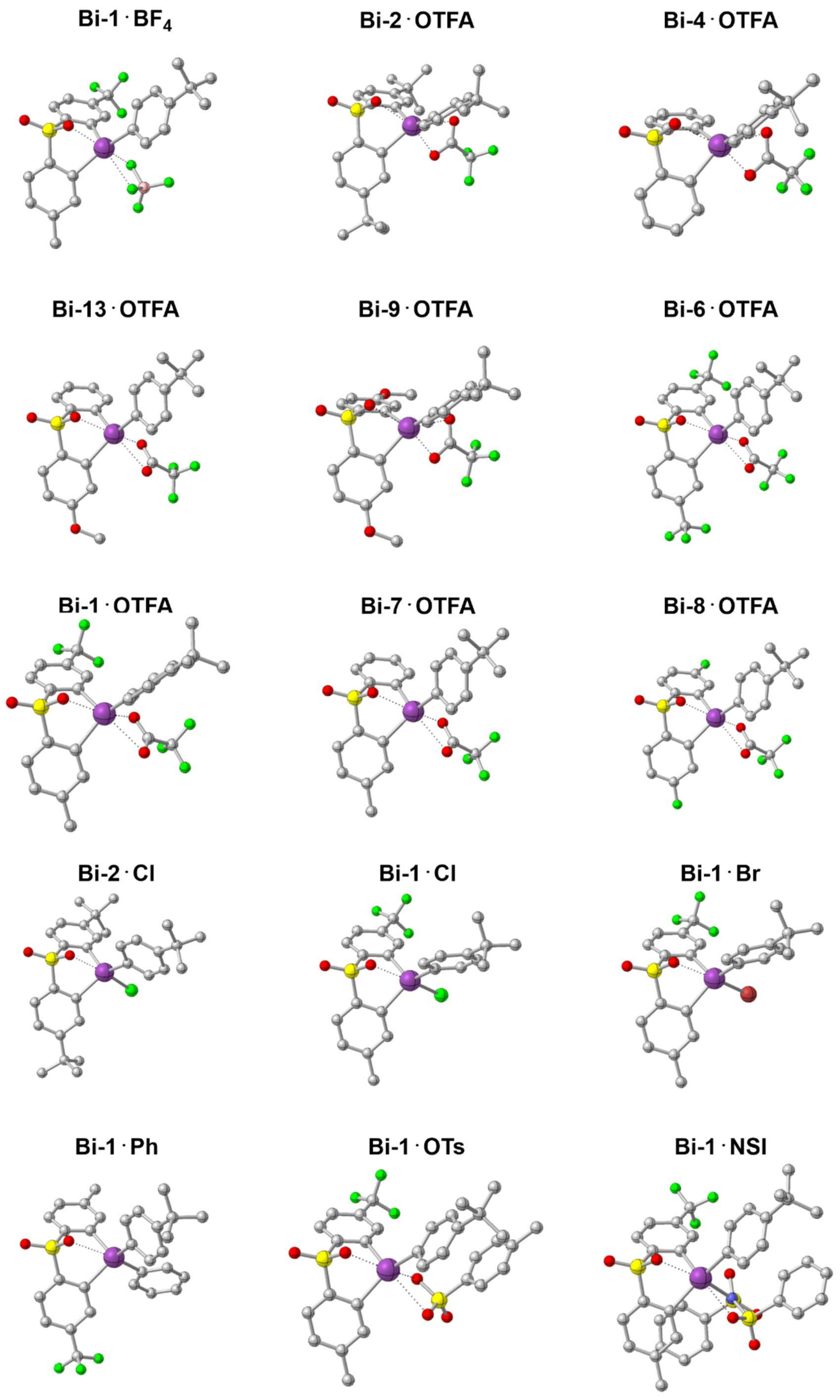

Figure S38 Optimized Structures of all bismuthonium complexes; cosmo(∞)-BP86-D4/def2-TZVP.

11.4. Oxidative Addition

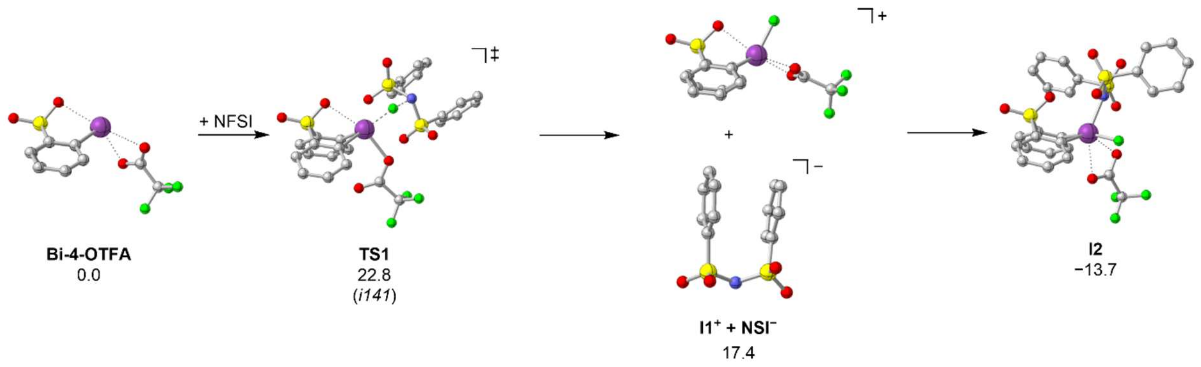

Figure S39 Stepwise oxidative addition via **TS1**; ΔG^{363} in kcal·mol^{-1}; B3LYP-D4/def2-TZVP//cosmo(∞)-BP86-D4/def2-TZVP; COSMO-RS (chloroform).

11.5. Transmetallation

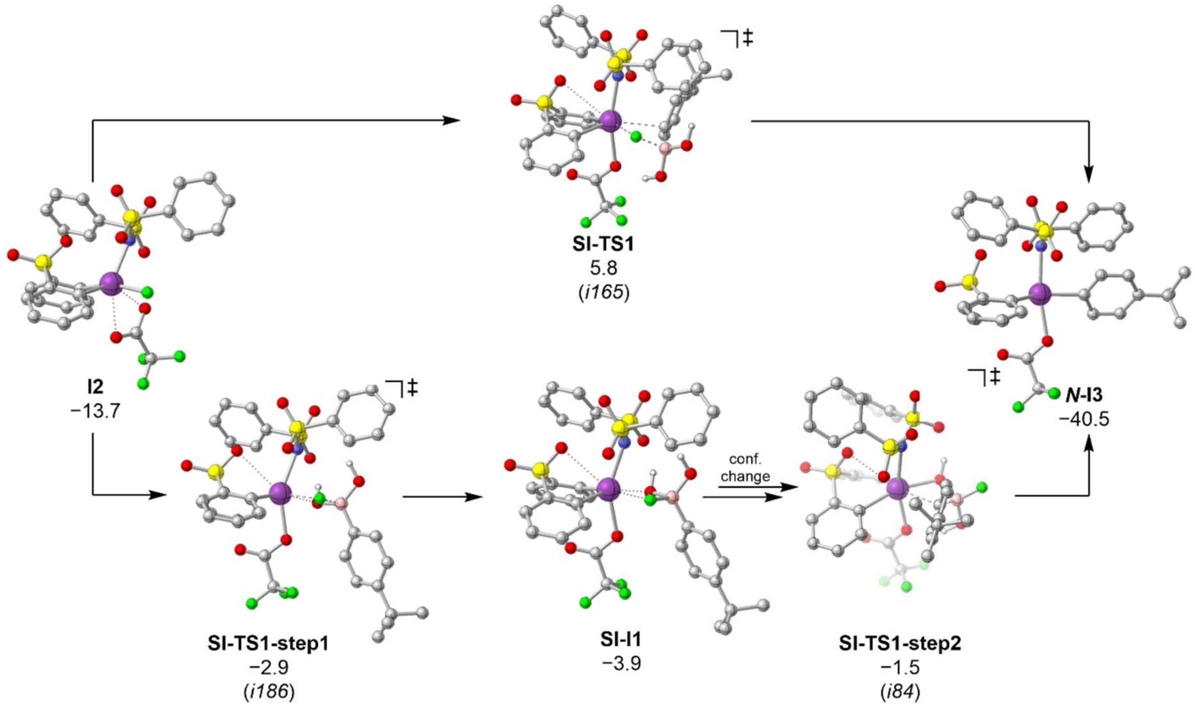

Figure S40 A stepwise transmetalation is preferred compared to a concerted transmetalation using boronic acid via **SI-TS1**; ΔG^{363} in kcal·mol^{-1}; B3LYP-D4/def2-TZVP//cosmo(∞)-BP86-D4/def2-TZVP; COSMO-RS (chloroform).

11.6. Bi(V) Intermediate **I3**: Coordination Isomers

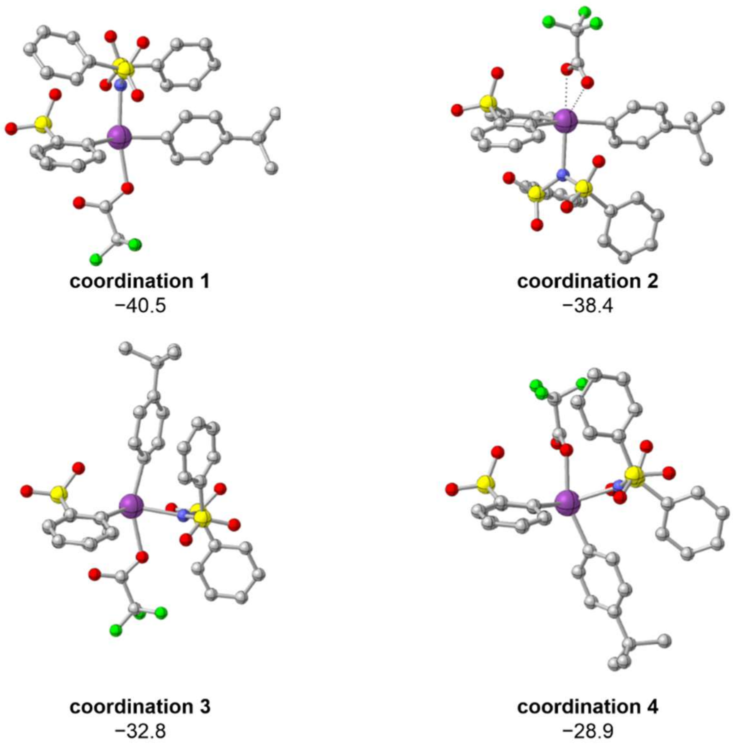

Figure S41 Coordination isomers for the *N*-bound Bi(V) intermediate ***N*-I3**; for each coordination a conformational search was conducted; ΔG^{363} in kcal·mol^{-1}; B3LYP-D4/def2-TZVP//cosmo(∞)-BP86-D4/def2-TZVP; COSMO-RS (chloroform).

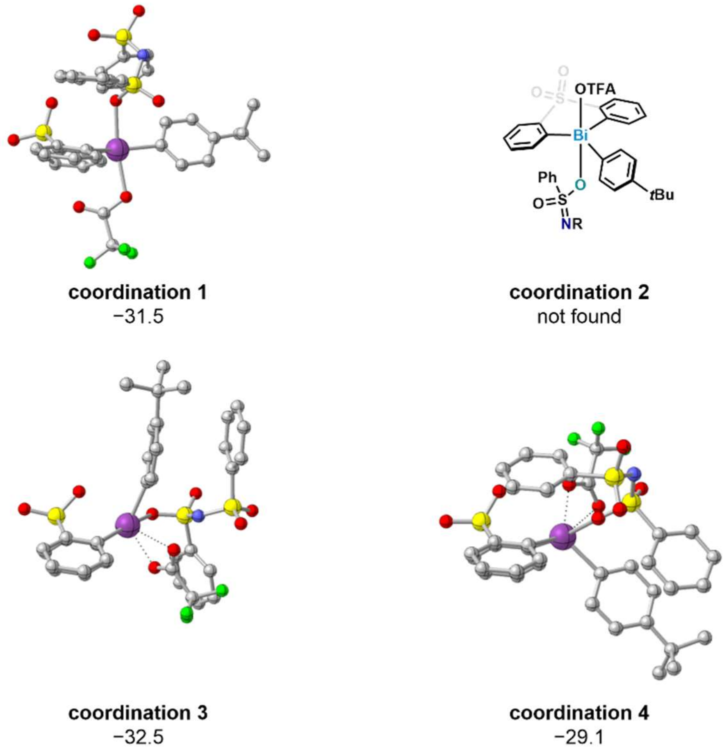

Figure S42 Coordination isomers for the *O*-bound Bi(V) intermediate ***O*-I3**; for each coordination a conformational search was conducted; ΔG^{363} in kcal·mol^{-1}; B3LYP-D4/def2-TZVP//cosmo(∞)-BP86-D4/def2-TZVP; COSMO-RS (chloroform).

11.7. Reductive Elimination: Pathway B

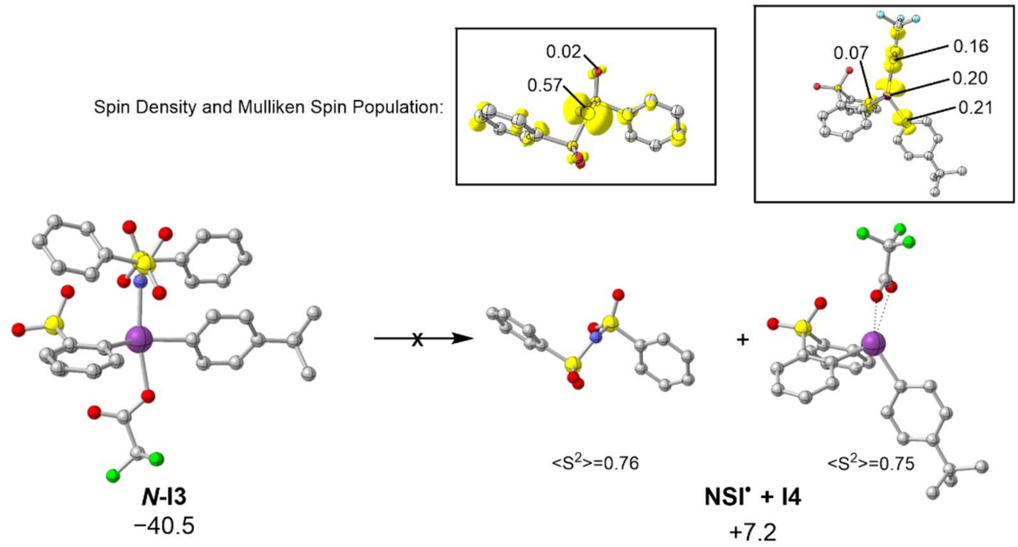

Figure S43 A formally Bi(IV) species is not feasible after once having reached a Bi(V) intermediate; ΔG^{363} in kcal·mol^{-1}; B3LYP-D4/def2-TZVP//cosmo(∞)-BP86-D4/def2-TZVP; COSMO-RS (chloroform).

11.8. Reductive Elimination: Pathway A and C

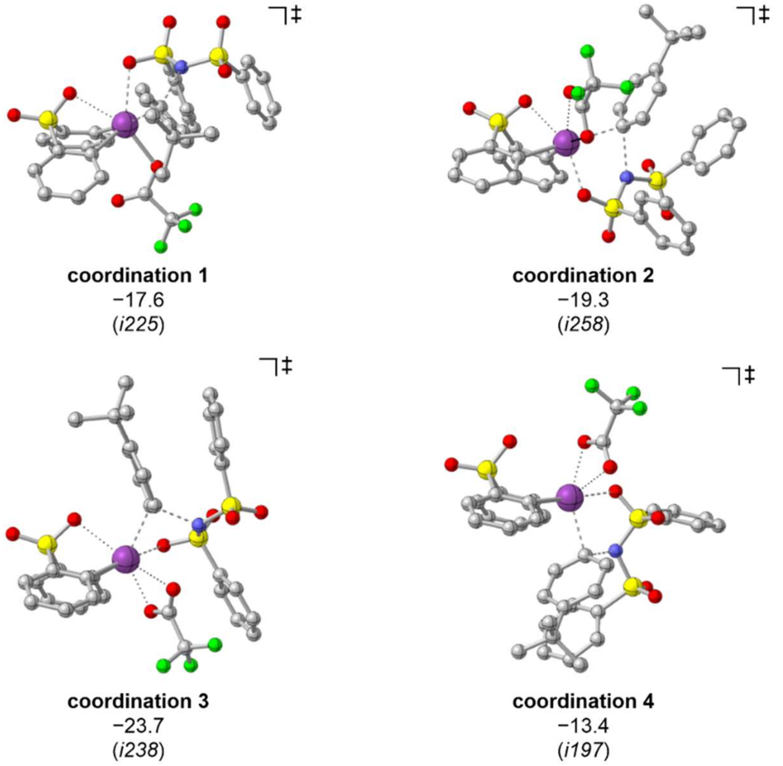

Figure S44 Coordination isomers for the five-membered transition states of the reductive elimination ***N*-TS2**; for each coordination a conformational search was conducted; ΔG^{363} in kcal·mol^{-1}; B3LYP-D4/def2-TZVP//cosmo(∞)-BP86-D4/def2-TZVP; COSMO-RS (chloroform).

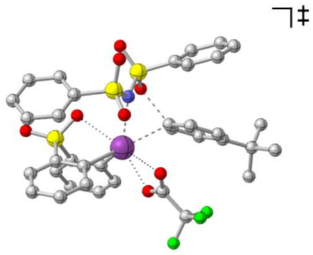

coordination 1
−23.0
(*i279*)

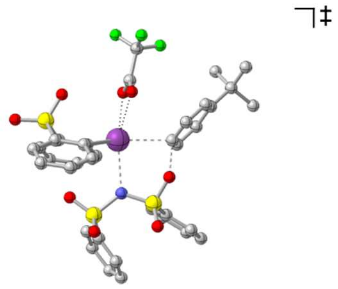

coordination 2
−17.7
(*i278*)

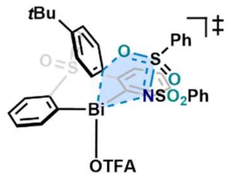

coordination 3
not found
always yielded **coordination 1**,
due to direct isomerization to
the more stable ***N*-I3-c1**

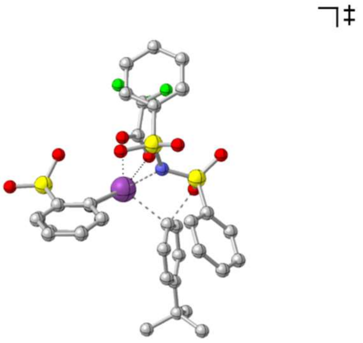

coordination 4
−11.7
(*i305*)

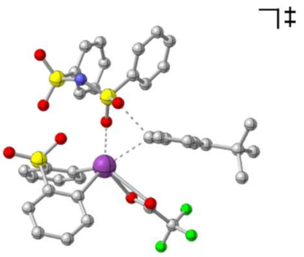

coordination 5
−15.8
(*i251*)

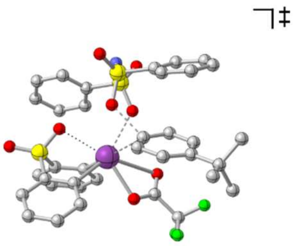

7-membered TS
−22.6
(*i260*)

Figure S45 Coordination isomers for the five-membered transition states of the reductive elimination ***O*-TS2**; the nomenclature refers to the according coordination isomers of ***N*-TS2**; for each coordination a conformational search was conducted; ΔG^{363} in kcal·mol^{-1}; B3LYP-D4/def2-TZVP//cosmo(∞)-BP86-D4/def2-TZVP; COSMO-RS (chloroform).

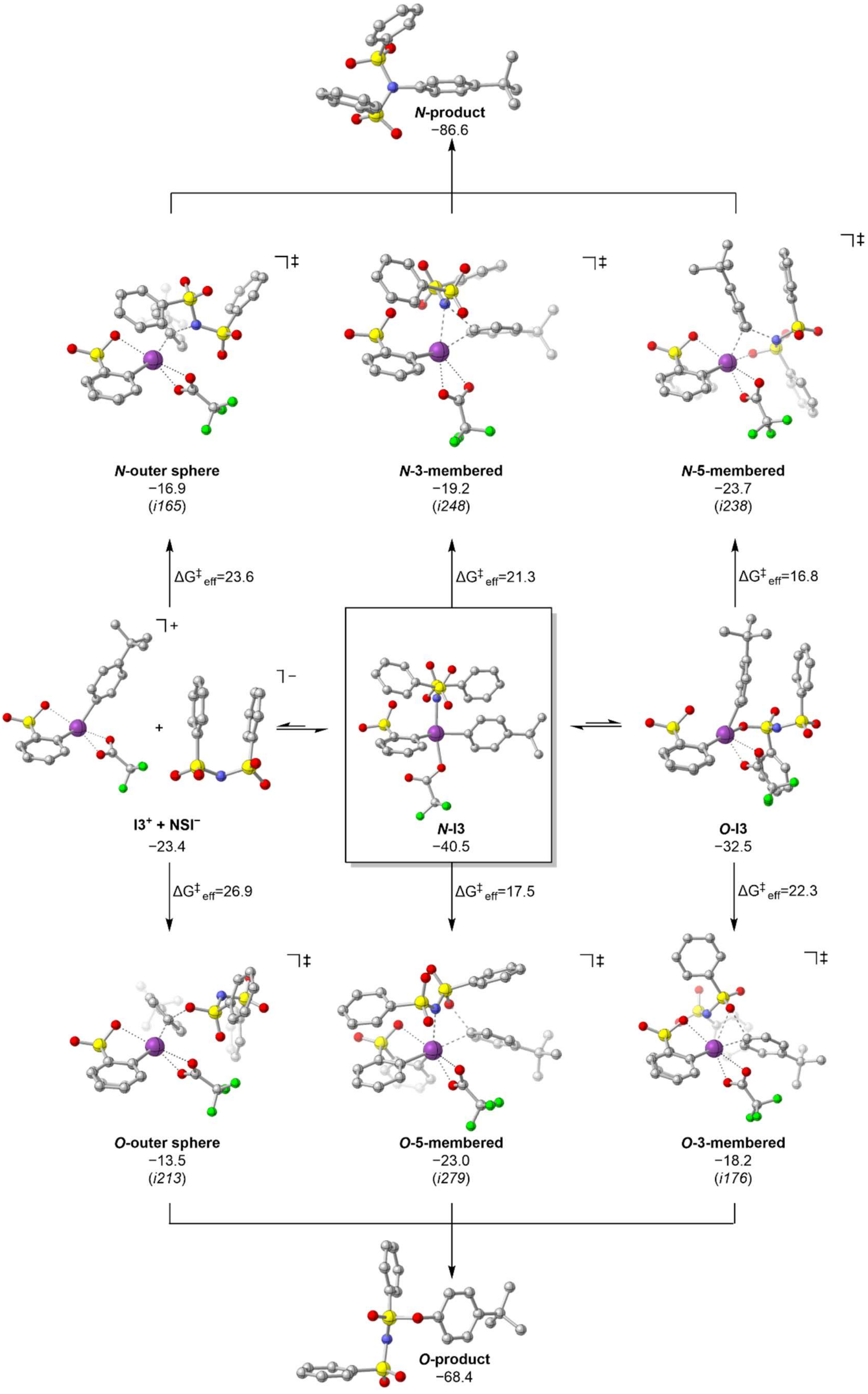

Figure S46 Different mechanisms for the reductive elimination yielding the *N*- or *O*-coupled product via a 3-membered TS, a 5-membered TS or an outer sphere attack; the outer sphere attack is considered as an extreme case of the 3-membered TS; **Bi-4·OTFA;** ΔG^{363} in kcal·mol^{-1}; B3LYP-D4/def2-TZVP//cosmo(∞)-BP86-D4/def2-TZVP; COSMO-RS (chloroform).

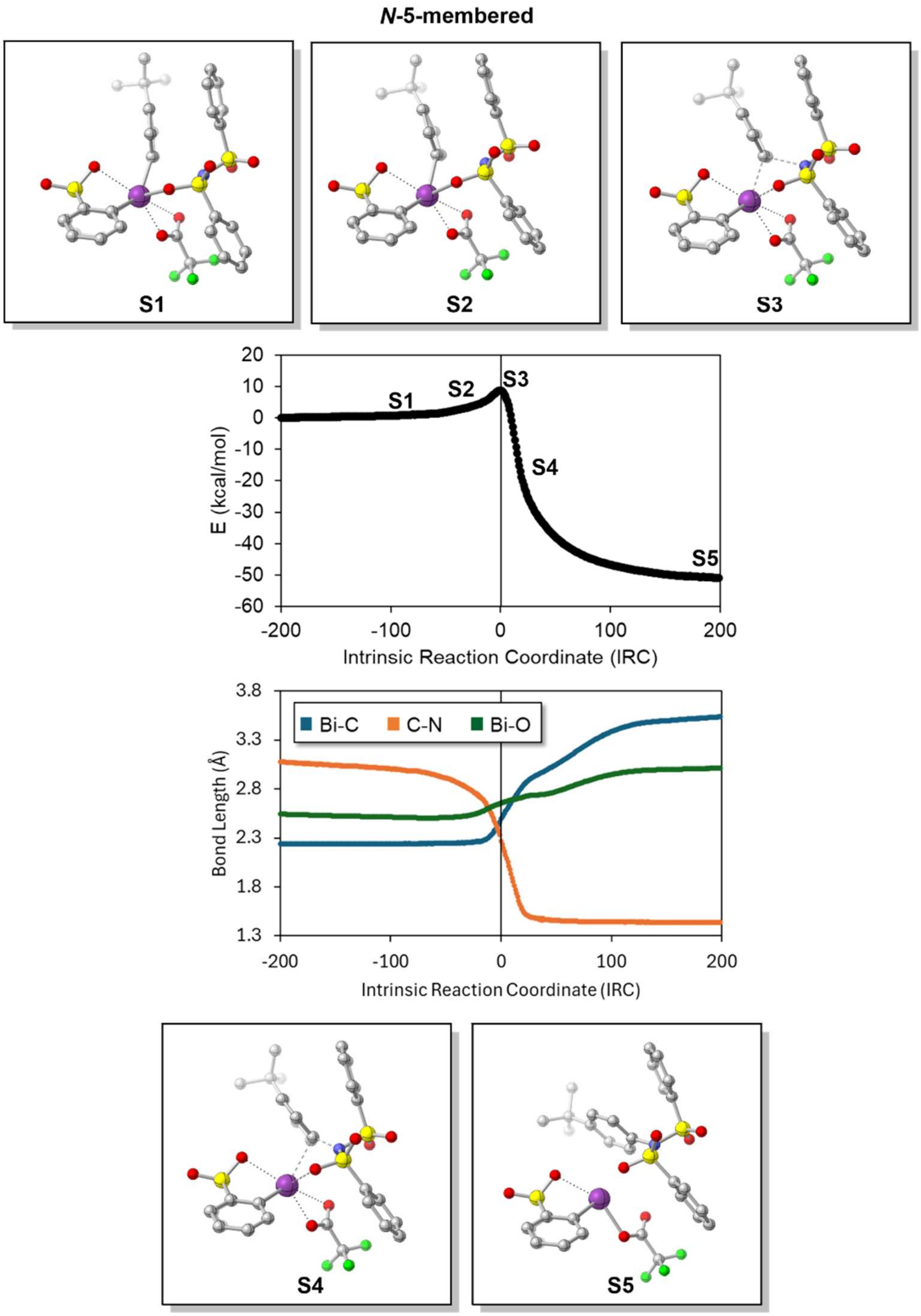

Figure S47 IRC calculation with selected structures for the reductive elimination to the *N*-product via the 5-membered TS; ΔE in kcal·mol^{-1}; cosmo(∞)-BP86-D4/def2-TZVP; COSMO-RS (chloroform).

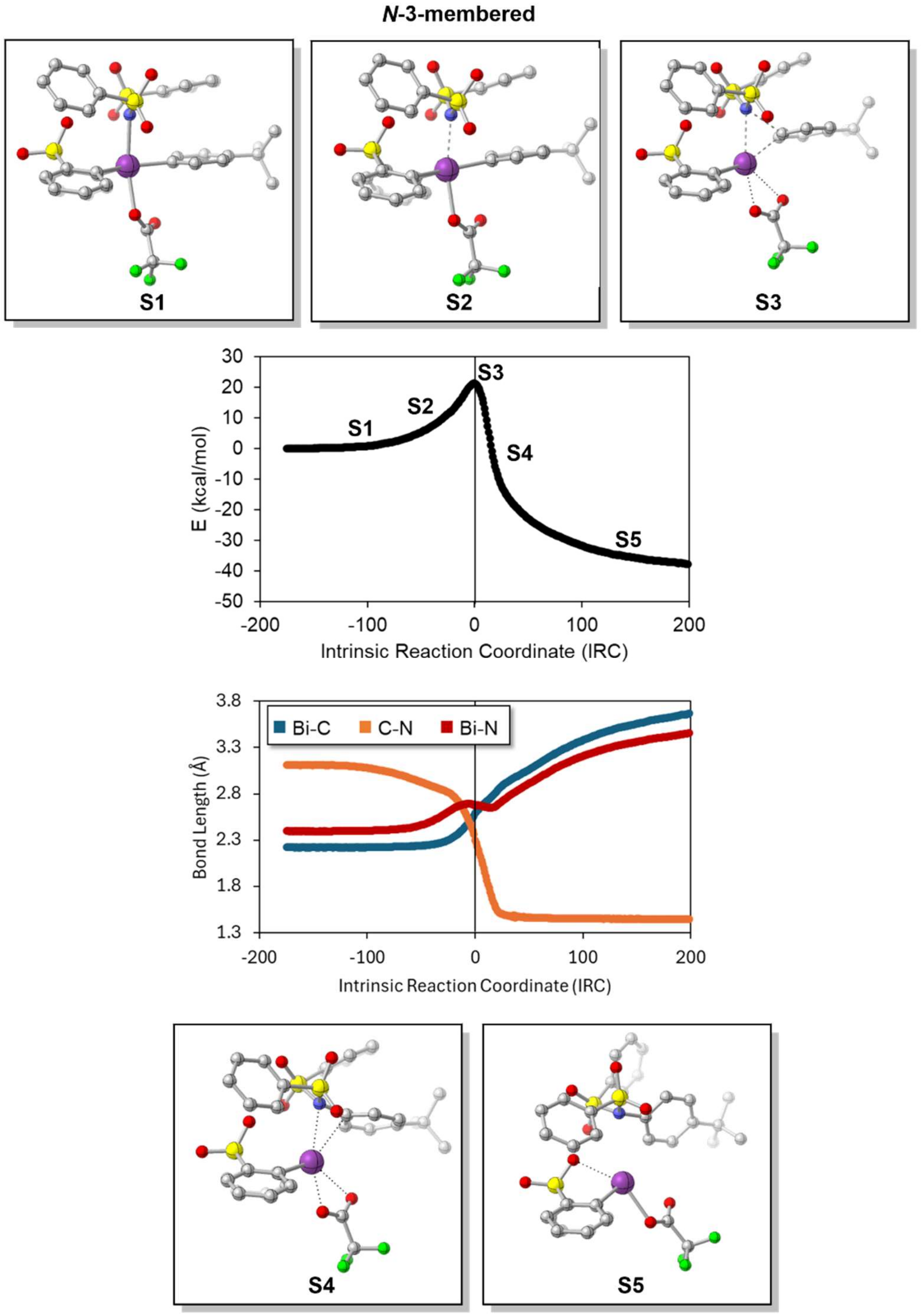

Figure S48 IRC calculation with selected structures for the reductive elimination to the *N*-product via the 3-membered TS; ΔE in kcal·mol^{-1}; cosmo(∞)-BP86-D4/def2-TZVP; COSMO-RS (chloroform).

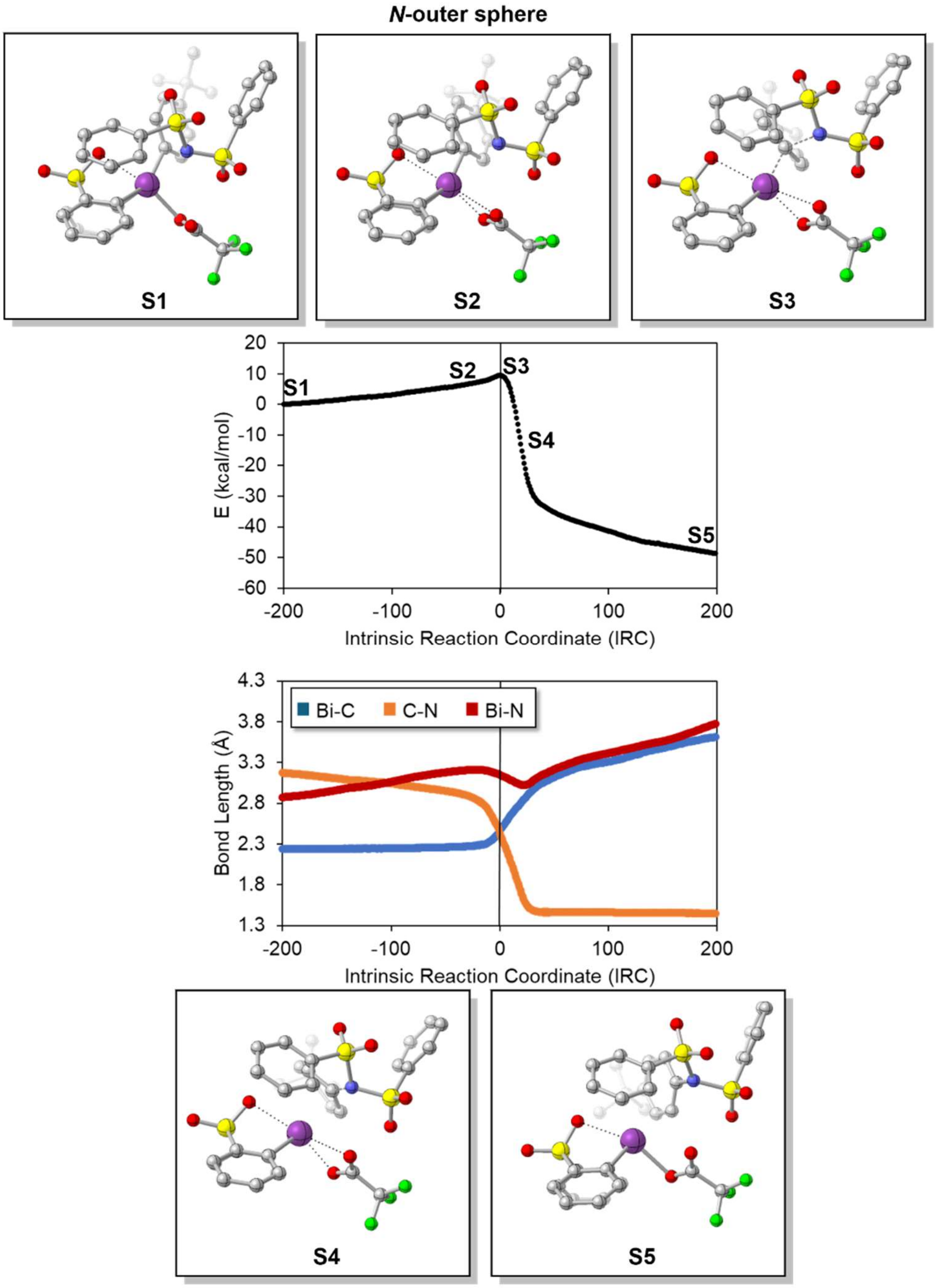

Figure S49 IRC calculation with selected structures for the reductive elimination to the *N*-product via the outer sphere TS; ΔE in kcal·mol^{-1}; cosmo(∞)-BP86-D4/def2-TZVP; COSMO-RS (chloroform).

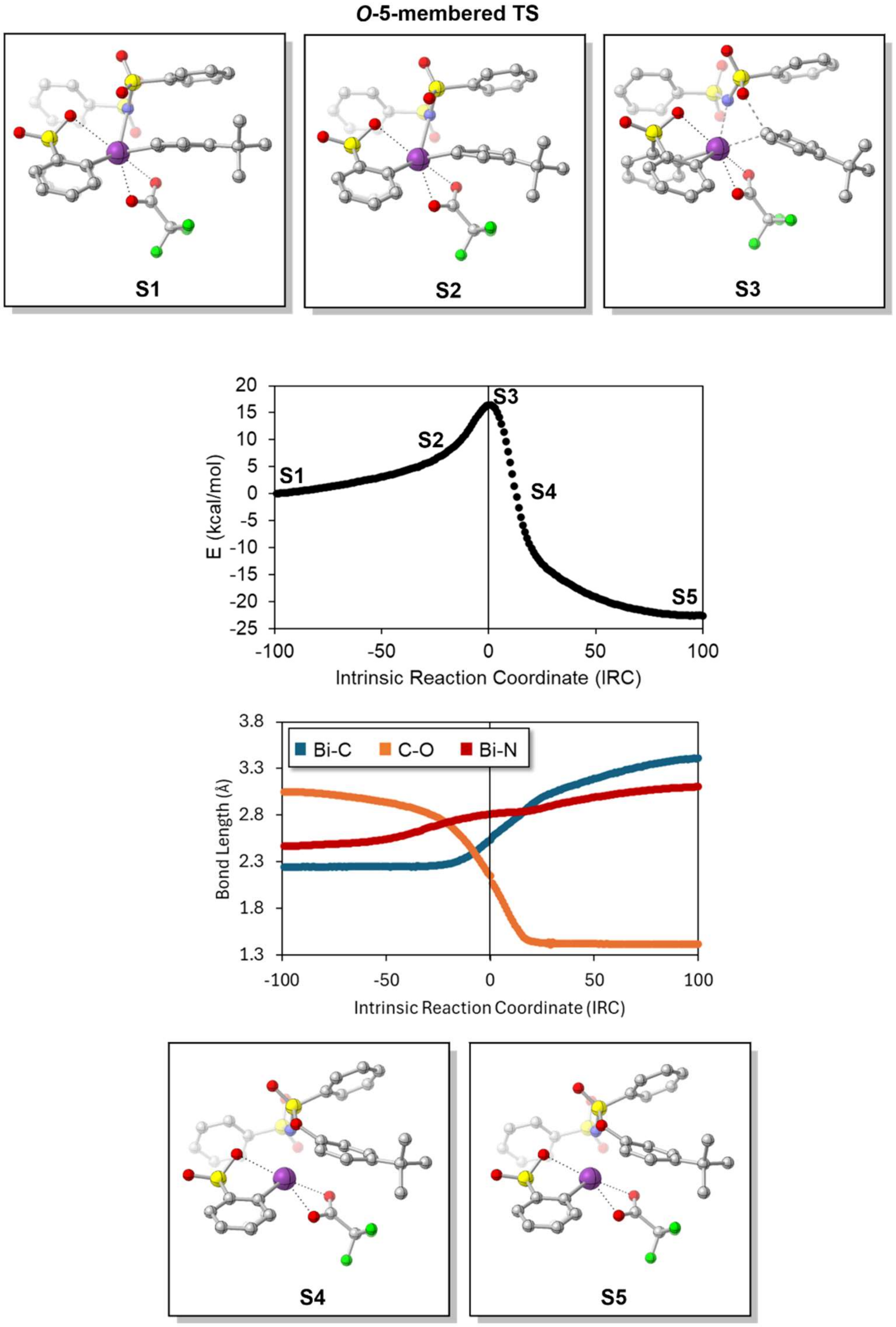

Figure S50 IRC calculation with selected structures for the reductive elimination to the *O*-product via the 5-membered TS; ΔE in kcal·mol^{-1}; cosmo(∞)-BP86-D4/def2-TZVP; COSMO-RS (chloroform).

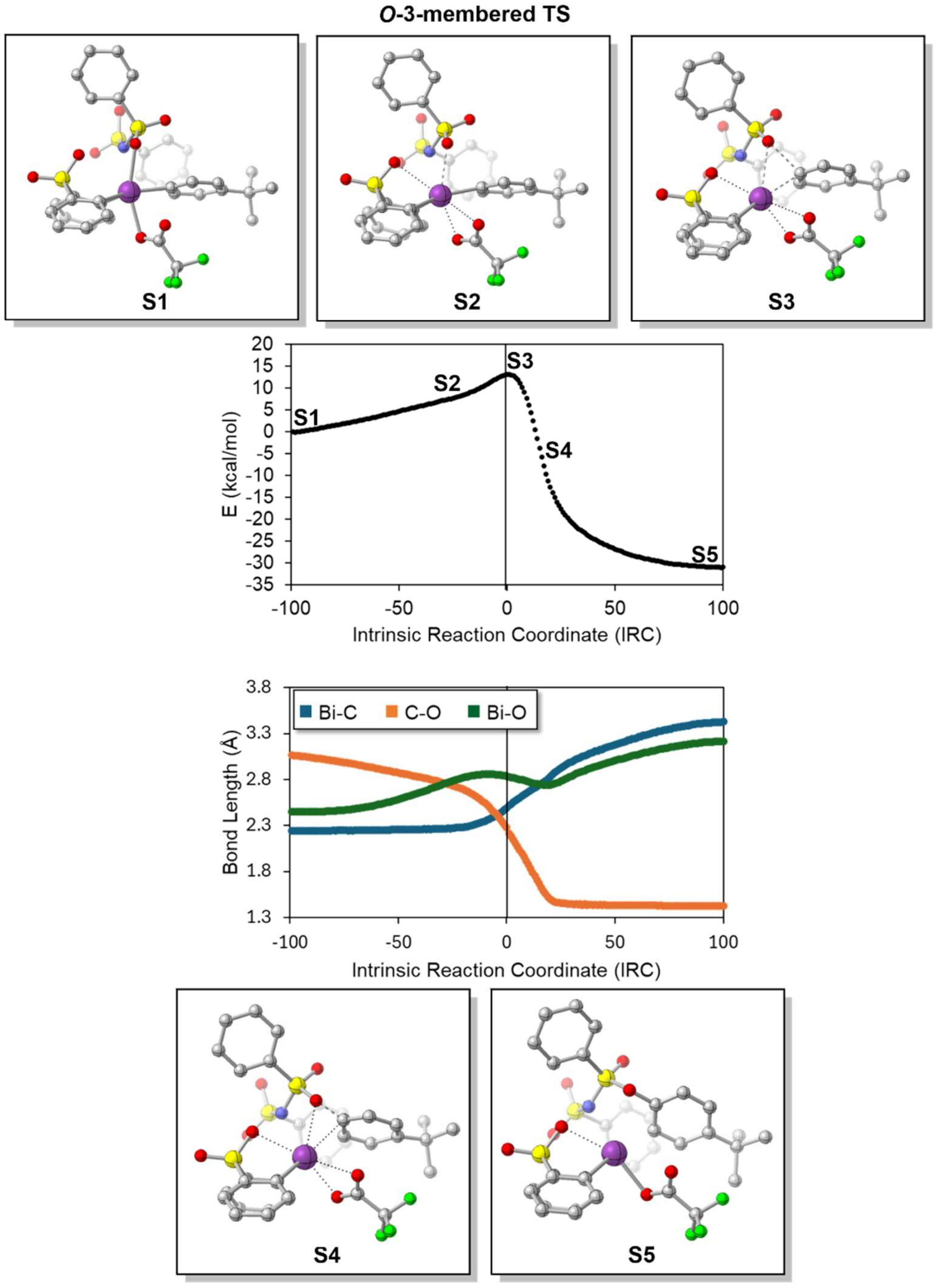

Figure S51 IRC calculation with selected structures for the reductive elimination to the *O*-product via the 3-membered TS; ΔE in kcal·mol^{-1}; cosmo(∞)-BP86-D4/def2-TZVP; COSMO-RS (chloroform).

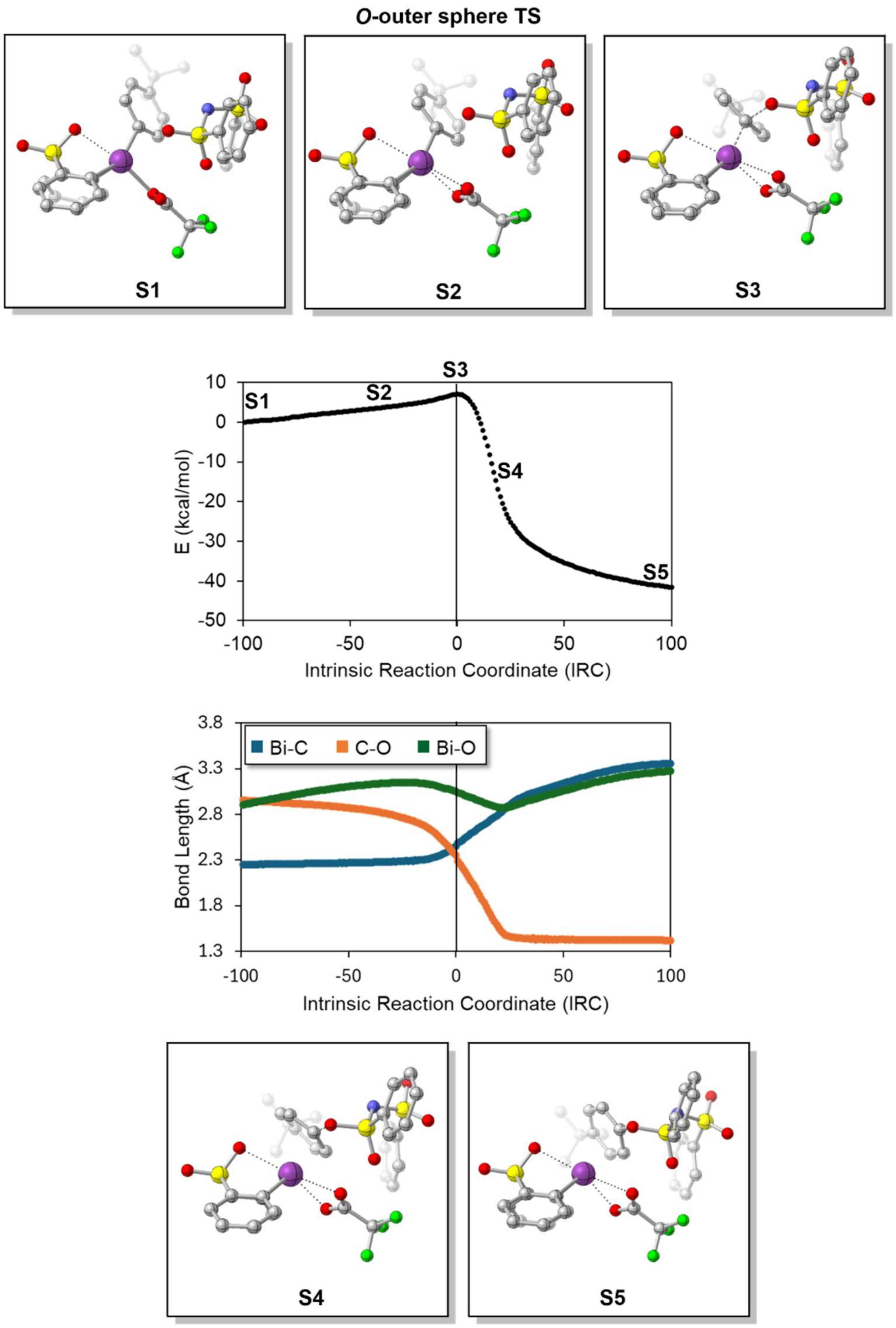

Figure S52 IRC calculation with selected structures for the reductive elimination to the *O*-product via the outer sphere TS; ΔE in kcal·mol^{-1}; cosmo(∞)-BP86-D4/def2-TZVP; COSMO-RS (chloroform).

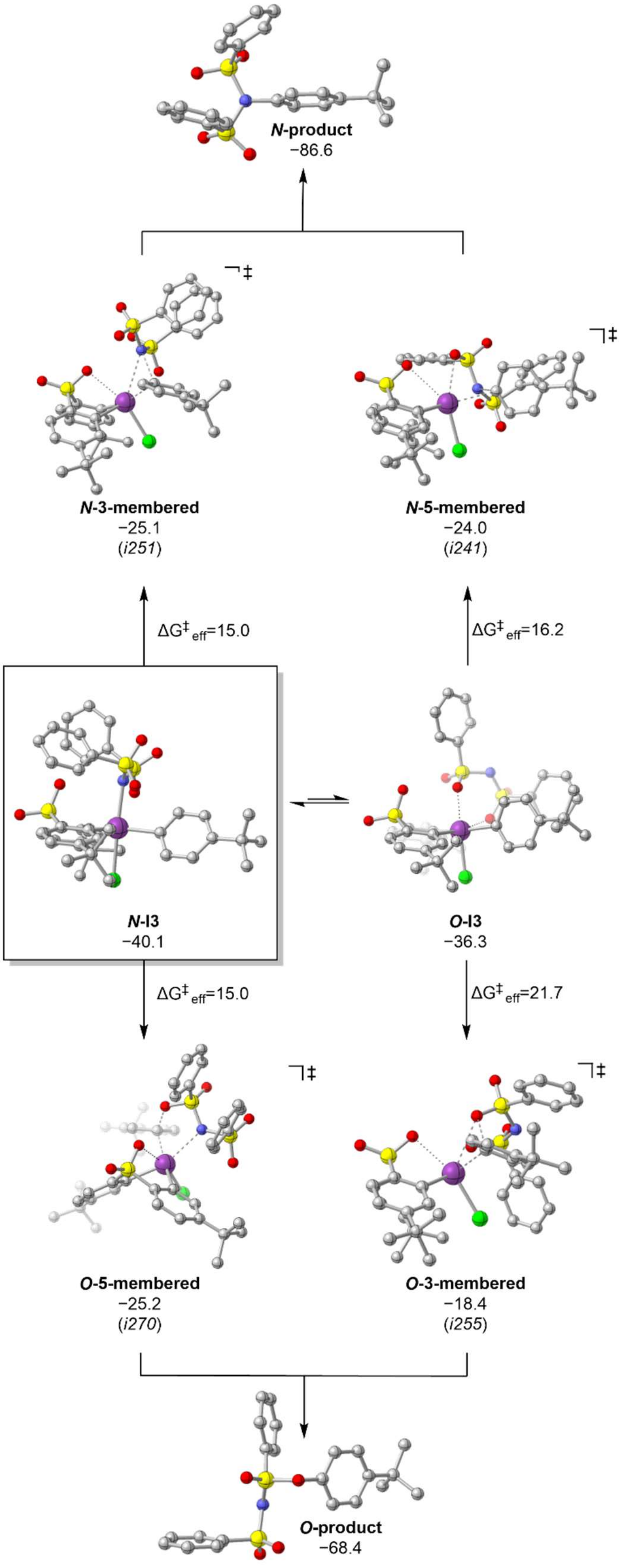

Figure S53 Different mechanisms for the reductive elimination for catalyst **Bi-2·Cl** yielding the *N*- or *O*-coupled product via a 3-membered TS or a 5-membered TS; ΔG^{363} in kcal·mol^{-1}; B3LYP-D4/def2-TZVP//cosmo(∞)-BP86-D4/def2-TZVP; COSMO-RS (chloroform).

The key step of the reductive elimination was calculated for a catalyst with sulfoximine backbone. For this, **Bi-5·OTs** was chosen, since this catalyst features the same backbone substitution as the sulfone model catalyst. In accordance with former work, for this analysis only the Bi−NCF$_3$-coordinated complexes were calculated.[1,3]

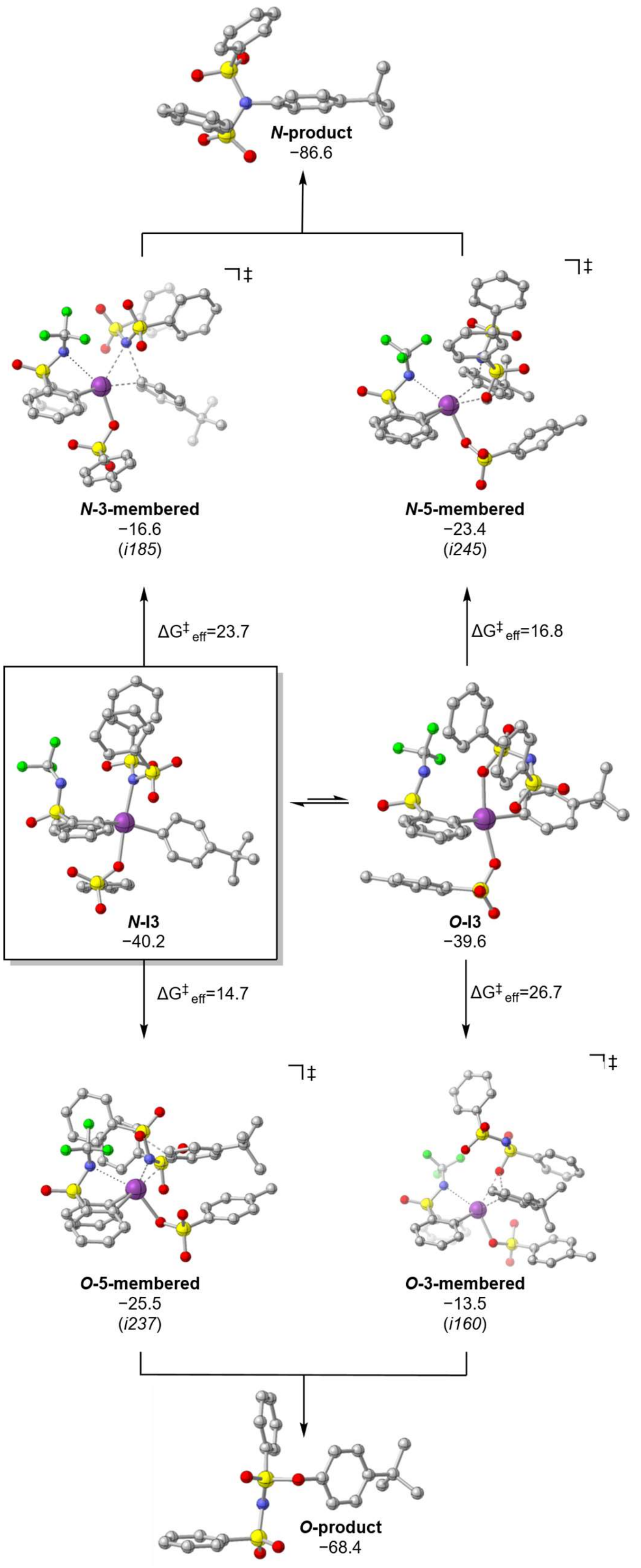

Figure S54 Different mechanisms for the reductive elimination for catalyst **Bi-5·OTs** yielding the *N*- or *O*-coupled product via a 3-membered TS or a 5-membered TS; ΔG^{363} in kcal·mol^{-1}; B3LYP-D4/def2-TZVP//cosmo(∞)-BP86-D4/def2-TZVP; COSMO-RS (chloroform).

12. Crystallographic Data

Suitable single crystal of **3a** was obtained by slow diffusion of pentane into a solution of **3a** in CH_2Cl_2 at 25 °C.

Single Crystal structure of **3a**

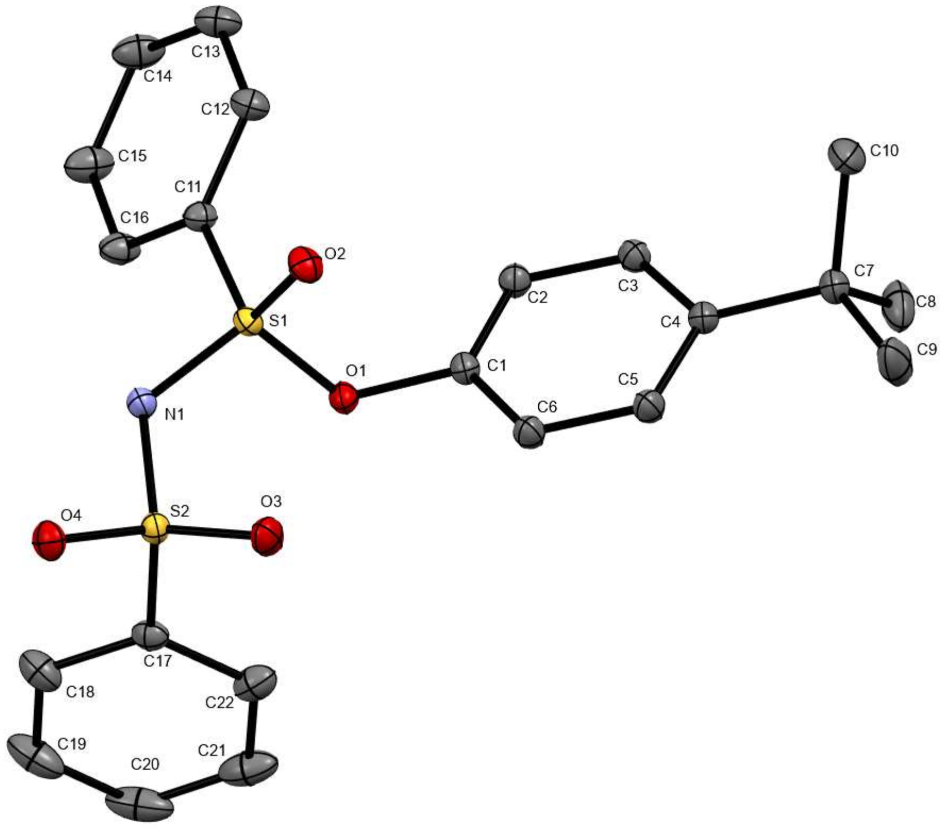

Figure S55 The molecular structure of compound **3a**. H atoms have been removed for clarity.

X-ray Crystal Structure Analysis of 3a:

$C_{22}H_{23}NO_4S_2$, M_r = 429.53 g mol^{-1}, yellow plate, crystal size 0.302 x 0.216 x 0.146 mm^3, Monoclinic, space group $P2_1/n$ [14], a = 8.6977(4) Å, b = 24.8844(11) Å, c = 9.8330(4) Å, β= 98.661(2)°, V = 2103.96(16) Å^3, T = 100(2) K, Z = 4, D_{calc} = 1.356 g·cm^3, λ = 0.71073 Å, $\mu(Mo\text{-}K_\alpha)$ = 0.282 mm^{-1}, Gaussian absorption correction (T_{min} = 0.9304, T_{max} = 1.0000), Bruker-AXS D8 Venture with Photon III detector and IµS Diamond microfocus Mo-anode X-ray source, 2.249 < θ < 33.164°, 396915 measured reflections, 8041 independent reflections, 7628 reflections with $I > 2\sigma(I)$, R_{int} = 0.0389. The structure was solved by *SHELXT* and refined by full-matrix least-squares (*SHELXL*) against F^2 to R_1 = 0.0269 [$I > 2\sigma(I)$], wR_2 = 0.0838 [all data], 354 parameters and 0 restraints.

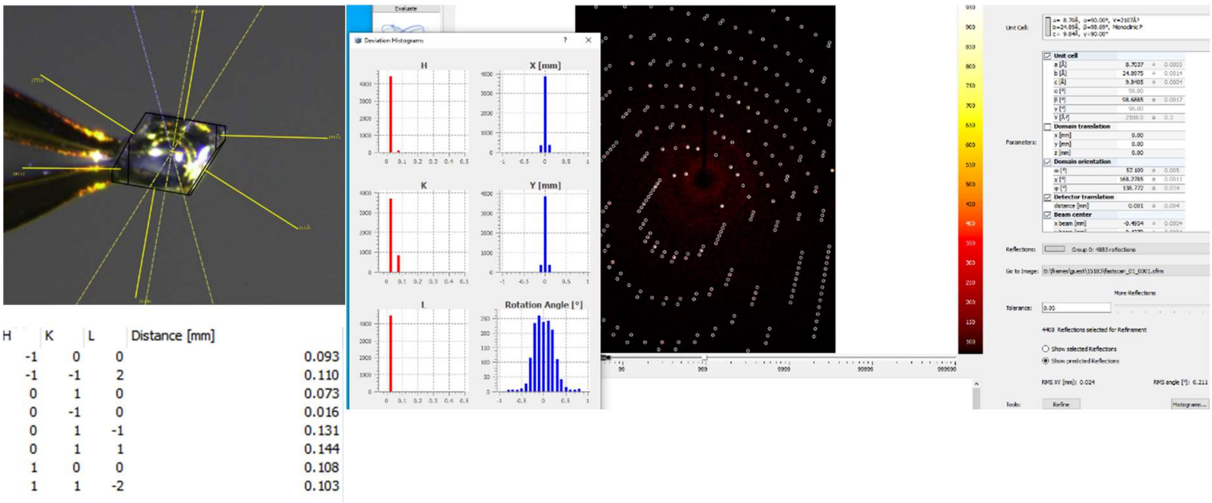

Figure S56 Crystal faces and unit cell determination/refinement of compound **3a**.

INTENSITY STATISTICS FOR DATASET

Resolution	#Data	#Theory	%Complete	Redundancy	Mean I	Mean I/s	Rmerge	Rsigma
Inf - 2.70	126	128	98.4	38.55	101.09	106.64	0.0279	0.0174
2.70 - 1.78	285	287	99.3	67.44	34.98	152.00	0.0288	0.0122
1.78 - 1.41	410	411	99.8	83.63	19.19	167.78	0.0278	0.0061
1.41 - 1.22	436	436	100.0	66.81	19.74	151.55	0.0277	0.0053
1.22 - 1.11	412	412	100.0	60.33	17.99	139.05	0.0299	0.0058
1.11 - 1.03	425	425	100.0	56.89	12.82	119.93	0.0325	0.0057
1.03 - 0.97	394	394	100.0	54.35	8.24	96.16	0.0373	0.0065
0.97 - 0.92	429	429	100.0	51.95	7.30	88.88	0.0416	0.0072
0.92 - 0.88	402	402	100.0	50.22	6.44	84.93	0.0456	0.0077
0.88 - 0.85	375	375	100.0	49.17	5.83	76.90	0.0488	0.0083
0.85 - 0.81	563	563	100.0	45.68	5.32	70.96	0.0528	0.0093
0.81 - 0.79	329	329	100.0	42.84	5.58	69.45	0.0492	0.0096
0.79 - 0.77	365	365	100.0	42.74	4.87	62.10	0.0541	0.0105
0.77 - 0.75	397	397	100.0	42.71	4.38	60.90	0.0585	0.0114
0.75 - 0.73	446	446	100.0	42.10	4.10	55.78	0.0598	0.0119
0.73 - 0.71	490	490	100.0	41.31	3.55	49.41	0.0647	0.0134
0.71 - 0.70	285	285	100.0	40.65	3.34	47.58	0.0682	0.0143
0.70 - 0.68	591	591	100.0	39.00	3.59	45.65	0.0687	0.0144
0.68 - 0.67	308	308	100.0	37.18	2.79	39.40	0.0846	0.0176
0.67 - 0.66	358	358	100.0	34.11	2.92	35.52	0.0904	0.0186
0.66 - 0.65	389	390	99.7	33.59	2.60	33.15	0.0936	0.0207
0.75 - 0.65	2867	2868	100.0	38.50	3.33	44.43	0.0718	0.0151
Inf - 0.65	8215	8221	99.9	48.89	9.87	82.28	0.0387	0.0101

All hydrogens were found in the residual density map and are freely refined.

Complete .cif-data of the compound are available under the CCDC number **CCDC-2298341**.

Crystal data and structure refinement of **3a**.

Identification code	15183	
Empirical formula	$C_{22} H_{23} N O_4 S_2$	
Color	yellow	
Formula weight	429.53 g · mol^{-1}	
Temperature	100(2) K	
Wavelength	0.71073 Å	
Crystal system	Monoclinic	
Space group	*P*2$_1$/*n*, (no. 14)	
Unit cell dimensions	a = 8.6977(4) Å	α= 90°.
	b = 24.8844(11) Å	β= 98.661(2)°.
	c = 9.8330(4) Å	γ = 90°.
Volume	2103.96(16) Å^3	
Z	4	
Density (calculated)	1.356 Mg · m^{-3}	
Absorption coefficient	0.282 mm^{-1}	
F(000)	904 e	
Crystal size	0.302 x 0.216 x 0.146 mm^3	
θ range for data collection	2.249 to 33.164°.	
Index ranges	-13 ≤ h ≤ 13, -38≤ k ≤ 38, -14 ≤ l ≤ 15	
Reflections collected	396915	
Independent reflections	8041 [R_{int} = 0.0389]	
Reflections with I>2σ(I)	7628	
Completeness to θ = 25.242°	99.9 %	
Absorption correction	Semi-empirical from equivalents	
Max. and min. transmission	1.00 and 0.93	
Refinement method	Full-matrix least-squares on F^2	
Data / restraints / parameters	8041 / 0 / 354	
Goodness-of-fit on F^2	1.068	
Final R indices [I>2σ(I)]	R_1 = 0.0269	wR^2 = 0.0822
R indices (all data)	R_1 = 0.0284	wR^2 = 0.0838
Largest diff. peak and hole	0.5 and -0.3 e · Å^{-3}	

Table S10. Bond lengths [Å] and angles [°] of **3a.**

S(1)-O(1)	1.6028(5)	S(1)-O(2)	1.4323(5)
S(1)-N(1)	1.5331(6)	S(1)-C(11)	1.7476(7)
S(2)-O(3)	1.4383(6)	S(2)-O(4)	1.4360(6)
S(2)-N(1)	1.6359(7)	S(2)-C(17)	1.7639(8)
O(1)-C(1)	1.4217(8)	C(1)-C(2)	1.3842(10)
C(1)-C(6)	1.3794(10)	C(2)-H(2)	0.995(13)
C(2)-C(3)	1.3913(10)	C(3)-H(3)	0.952(14)
C(3)-C(4)	1.4025(10)	C(4)-C(5)	1.3960(10)
C(4)-C(7)	1.5320(10)	C(5)-H(5)	0.939(14)
C(5)-C(6)	1.3947(10)	C(6)-H(6)	0.930(13)
C(7)-C(8)	1.5382(13)	C(7)-C(9)	1.5314(12)
C(7)-C(10)	1.5337(11)	C(8)-H(8A)	0.950(16)
C(8)-H(8B)	0.988(17)	C(8)-H(8C)	0.983(17)
C(9)-H(9A)	0.956(16)	C(9)-H(9B)	0.996(17)
C(9)-H(9C)	0.953(16)	C(10)-H(10A)	0.982(14)
C(10)-H(10B)	1.017(15)	C(10)-H(10C)	0.968(16)
C(11)-C(12)	1.3918(10)	C(11)-C(16)	1.3949(10)
C(12)-H(12)	0.943(14)	C(12)-C(13)	1.3924(11)
C(13)-H(13)	0.929(15)	C(13)-C(14)	1.3877(12)
C(14)-H(14)	0.974(16)	C(14)-C(15)	1.3941(12)
C(15)-H(15)	0.996(15)	C(15)-C(16)	1.3889(11)
C(16)-H(16)	0.955(14)	C(17)-C(18)	1.3932(11)
C(17)-C(22)	1.3942(11)	C(18)-H(18)	0.964(14)
C(18)-C(19)	1.3926(12)	C(19)-H(19)	0.976(17)
C(19)-C(20)	1.3886(17)	C(20)-H(20)	0.970(17)
C(20)-C(21)	1.3875(17)	C(21)-H(21)	0.923(17)
C(21)-C(22)	1.3956(13)	C(22)-H(22)	0.949(15)
O(1)-S(1)-C(11)	104.17(3)	O(2)-S(1)-O(1)	108.87(3)
O(2)-S(1)-N(1)	121.51(4)	O(2)-S(1)-C(11)	110.38(3)
N(1)-S(1)-O(1)	105.81(3)	N(1)-S(1)-C(11)	104.71(3)
O(3)-S(2)-N(1)	111.60(3)	O(3)-S(2)-C(17)	108.70(4)
O(4)-S(2)-O(3)	118.63(4)	O(4)-S(2)-N(1)	105.92(3)
O(4)-S(2)-C(17)	107.29(3)	N(1)-S(2)-C(17)	103.60(3)
C(1)-O(1)-S(1)	117.72(4)	S(1)-N(1)-S(2)	121.05(4)
C(2)-C(1)-O(1)	119.75(6)	C(6)-C(1)-O(1)	117.82(6)
C(6)-C(1)-C(2)	122.40(6)	C(1)-C(2)-H(2)	121.3(7)

C(1)-C(2)-C(3)	118.10(7)	C(3)-C(2)-H(2)	120.5(7)
C(2)-C(3)-H(3)	117.2(8)	C(2)-C(3)-C(4)	121.77(7)
C(4)-C(3)-H(3)	120.9(8)	C(3)-C(4)-C(7)	119.64(6)
C(5)-C(4)-C(3)	117.76(6)	C(5)-C(4)-C(7)	122.60(6)
C(4)-C(5)-H(5)	120.7(9)	C(6)-C(5)-C(4)	121.54(7)
C(6)-C(5)-H(5)	117.7(9)	C(1)-C(6)-C(5)	118.43(7)
C(1)-C(6)-H(6)	119.7(8)	C(5)-C(6)-H(6)	121.9(8)
C(4)-C(7)-C(8)	109.28(7)	C(4)-C(7)-C(10)	109.68(6)
C(9)-C(7)-C(4)	112.15(7)	C(9)-C(7)-C(8)	108.62(8)
C(9)-C(7)-C(10)	108.23(8)	C(10)-C(7)-C(8)	108.82(7)
C(7)-C(8)-H(8A)	109.5(10)	C(7)-C(8)-H(8B)	112.4(10)
C(7)-C(8)-H(8C)	109.4(9)	H(8A)-C(8)-H(8B)	108.7(13)
H(8A)-C(8)-H(8C)	108.9(13)	H(8B)-C(8)-H(8C)	107.8(13)
C(7)-C(9)-H(9A)	114.3(9)	C(7)-C(9)-H(9B)	110.0(10)
C(7)-C(9)-H(9C)	109.8(9)	H(9A)-C(9)-H(9B)	108.6(14)
H(9A)-C(9)-H(9C)	106.1(13)	H(9B)-C(9)-H(9C)	107.9(13)
C(7)-C(10)-H(10A)	108.7(8)	C(7)-C(10)-H(10B)	109.8(9)
C(7)-C(10)-H(10C)	110.0(9)	H(10A)-C(10)-H(10B)	110.9(12)
H(10A)-C(10)-H(10C)	108.4(13)	H(10B)-C(10)-H(10C)	109.0(13)
C(12)-C(11)-S(1)	117.49(6)	C(12)-C(11)-C(16)	122.42(7)
C(16)-C(11)-S(1)	119.98(5)	C(11)-C(12)-H(12)	121.8(9)
C(11)-C(12)-C(13)	118.37(7)	C(13)-C(12)-H(12)	119.8(9)
C(12)-C(13)-H(13)	118.8(9)	C(14)-C(13)-C(12)	120.09(7)
C(14)-C(13)-H(13)	121.1(9)	C(13)-C(14)-H(14)	121.2(9)
C(13)-C(14)-C(15)	120.72(7)	C(15)-C(14)-H(14)	118.1(9)
C(14)-C(15)-H(15)	119.6(9)	C(16)-C(15)-C(14)	120.20(8)
C(16)-C(15)-H(15)	120.2(9)	C(11)-C(16)-H(16)	121.2(8)
C(15)-C(16)-C(11)	118.20(7)	C(15)-C(16)-H(16)	120.5(8)
C(18)-C(17)-S(2)	118.46(6)	C(18)-C(17)-C(22)	121.81(7)
C(22)-C(17)-S(2)	119.71(6)	C(17)-C(18)-H(18)	120.8(9)
C(19)-C(18)-C(17)	118.74(9)	C(19)-C(18)-H(18)	120.4(9)
C(18)-C(19)-H(19)	117.9(10)	C(20)-C(19)-C(18)	120.17(9)
C(20)-C(19)-H(19)	121.9(10)	C(19)-C(20)-H(20)	120.7(10)
C(21)-C(20)-C(19)	120.49(8)	C(21)-C(20)-H(20)	118.8(10)
C(20)-C(21)-H(21)	120.8(11)	C(20)-C(21)-C(22)	120.38(9)
C(22)-C(21)-H(21)	118.8(10)	C(17)-C(22)-C(21)	118.39(9)
C(17)-C(22)-H(22)	121.5(9)	C(21)-C(22)-H(22)	120.1(9)

13. NMR Spectra

2,8-difluoro-10-phenyl-10H-dibenzo[b,e][1,4]thiabismine 5,5-dioxide (Bi-8·Ph)

1H NMR (600 MHz, CDCl3)

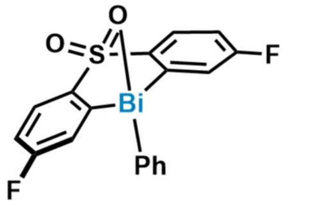

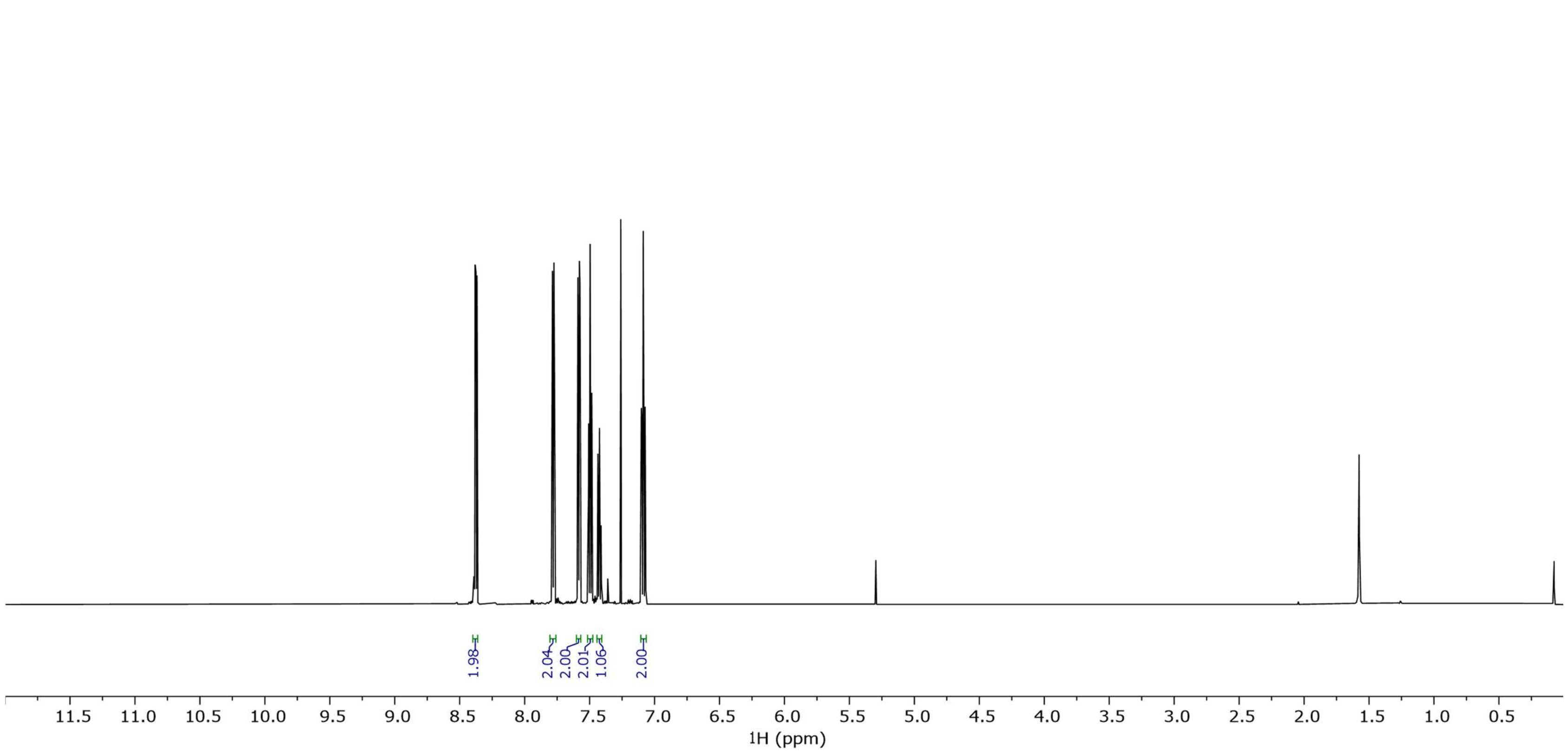

13C NMR (150 MHz, CDCl3)

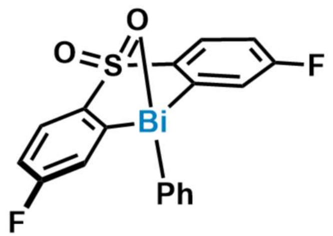

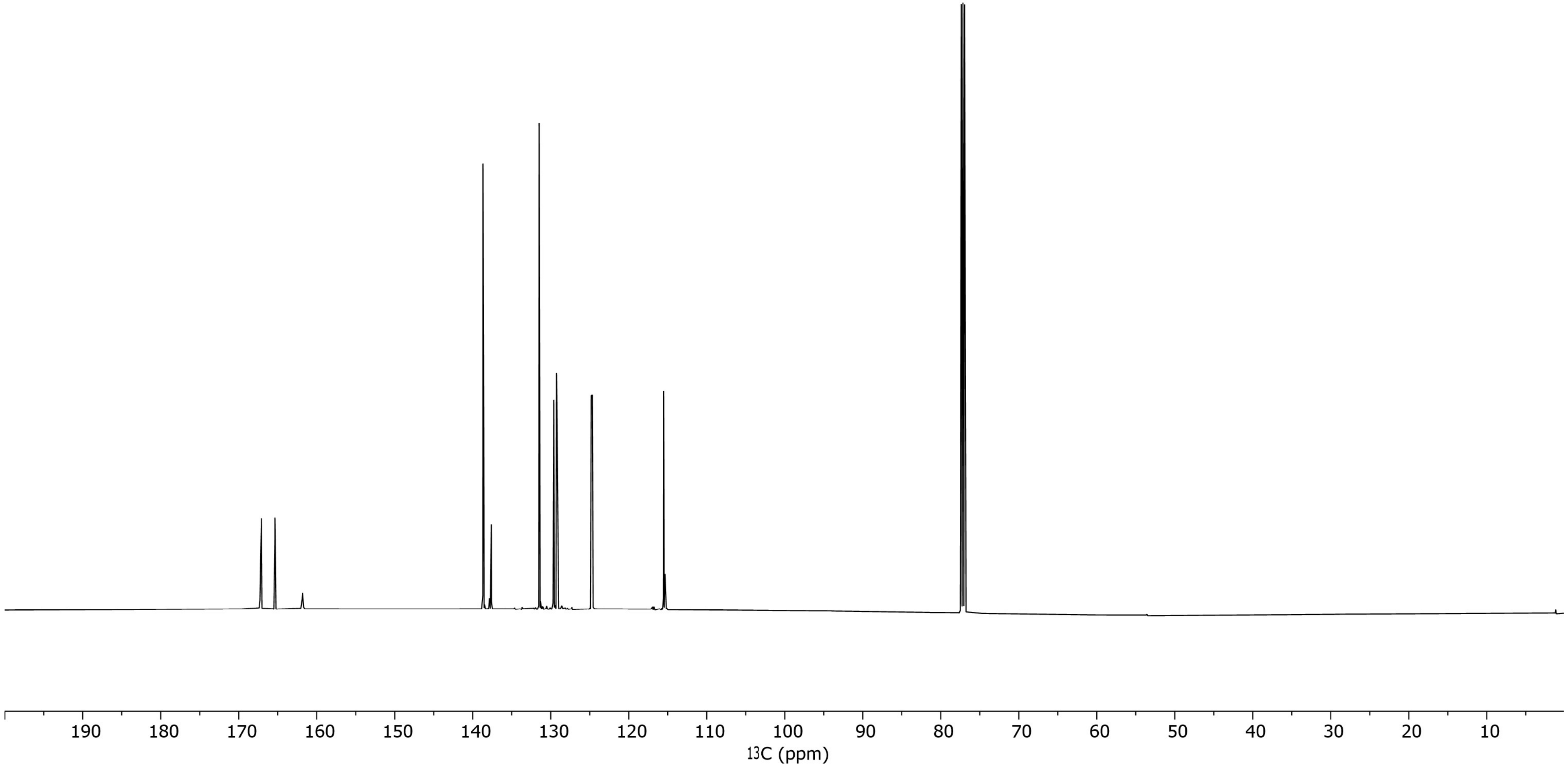

19F NMR (564 MHz, CDCl3)

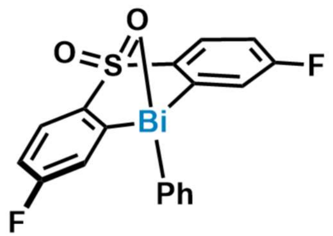

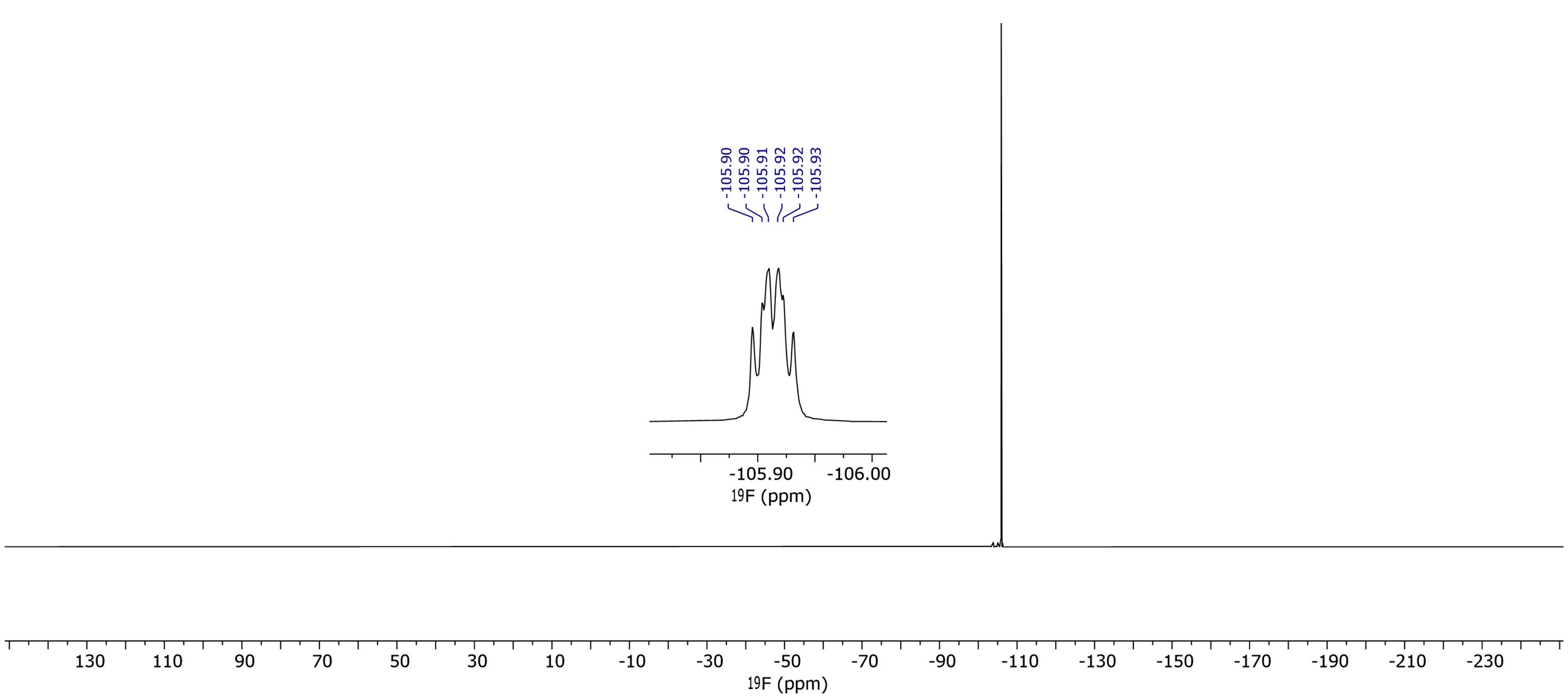

1,3,7,9-tetraethyl-10-phenyl-10H-dibenzo[b,e][1,4]thiabismine 5,5-dioxide (Bi-11·Ph)
1H NMR (600 MHz, CDCl3)

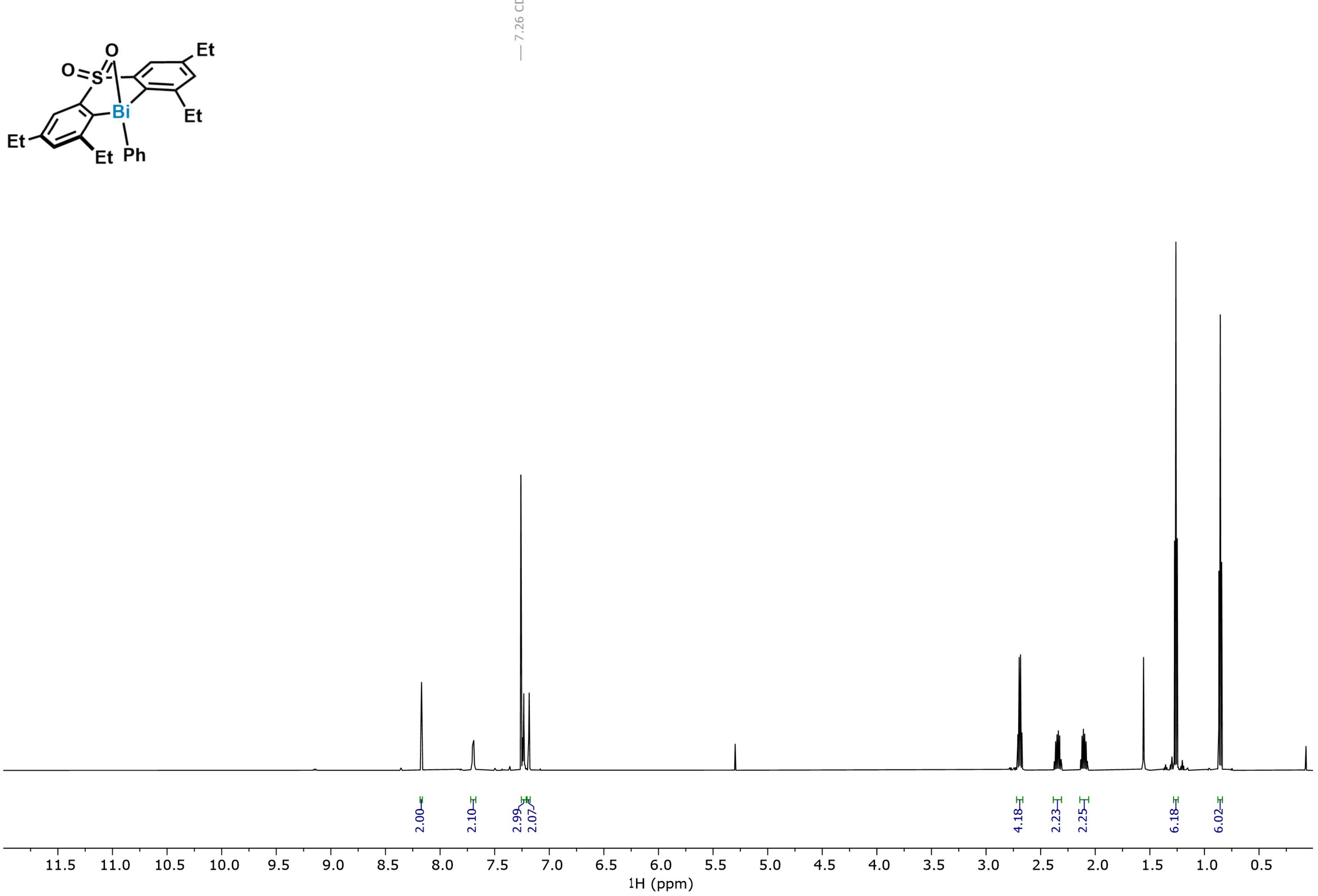

13C NMR (150 MHz, CDCl3)

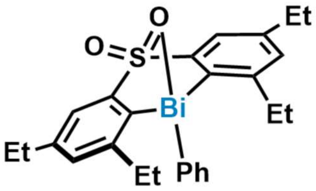

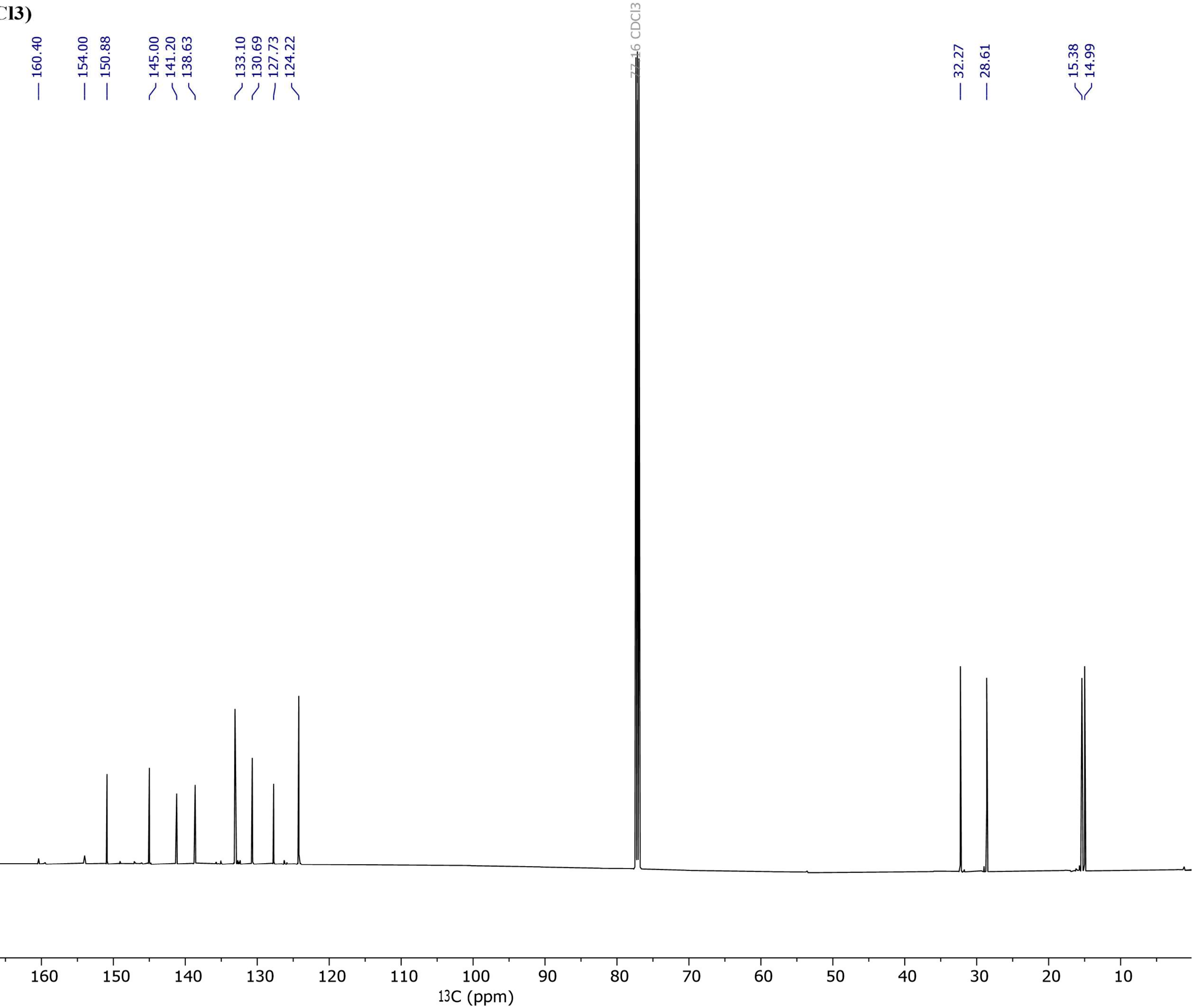

1,3,7,9-tetrapropyl-10-phenyl-10H-dibenzo[b,e][1,4]thiabismine 5,5-dioxide (Bi-12·Ph)

1H-NMR (600 MHz, CDCl3)

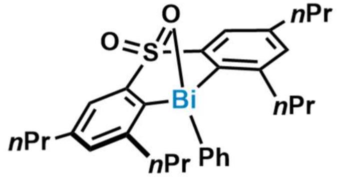

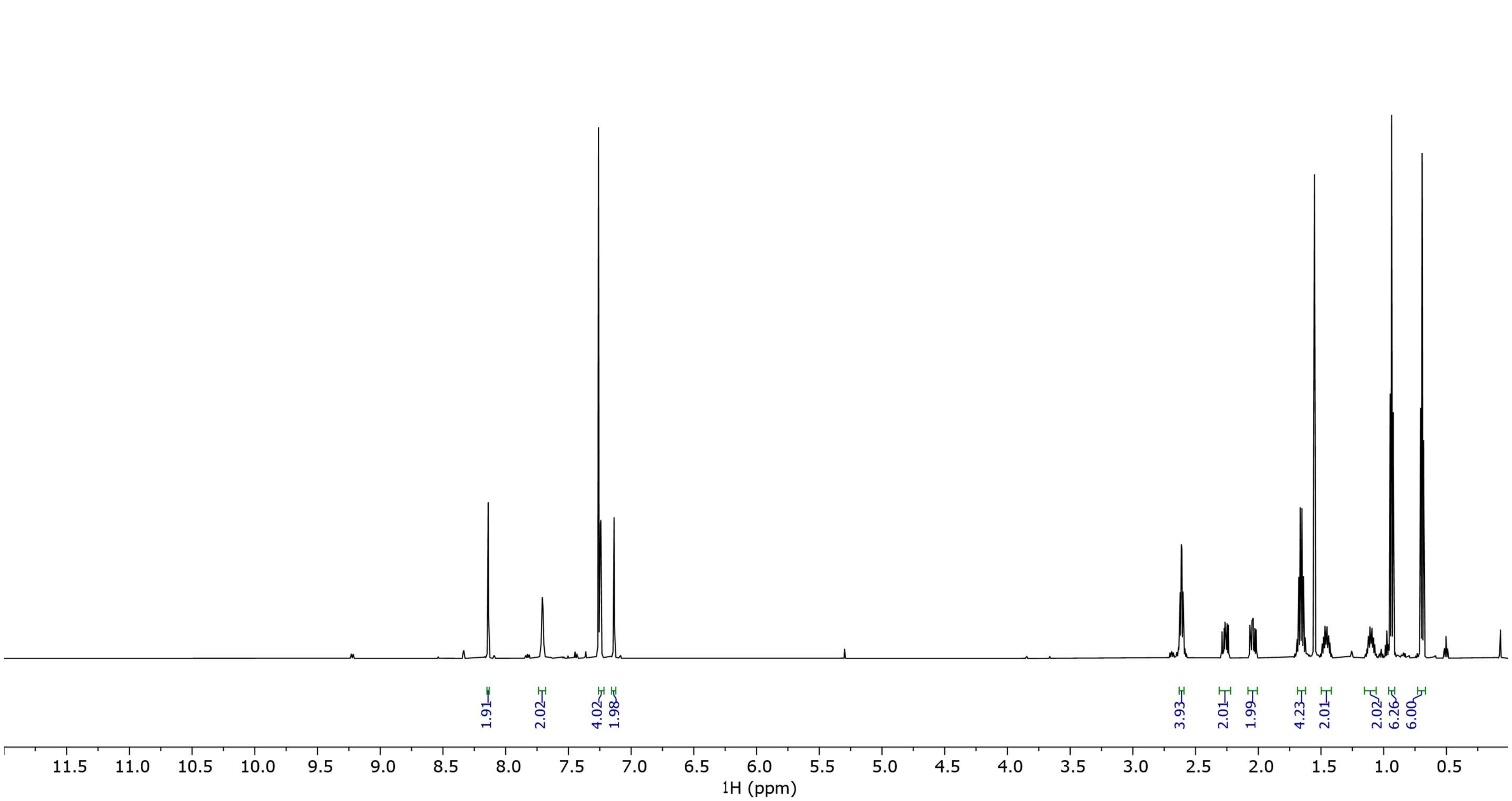

13C NMR (150 MHz, CDCl3)

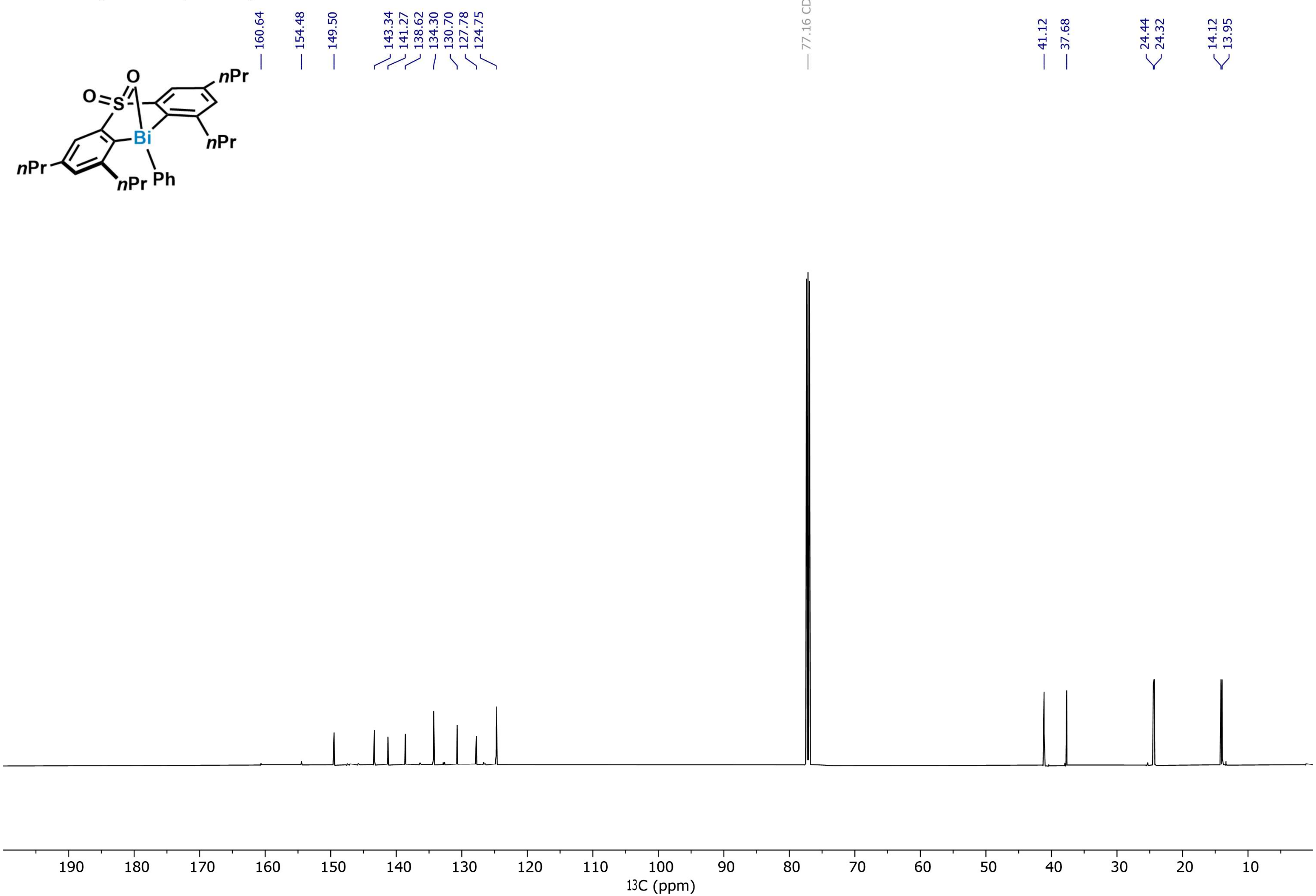

2,8-di-tert-butyl-5,5-dioxido-10H-dibenzo[b,e][1,4]thiabismin-10-yl 2,2,2-trifluoroacetate (Bi-2·OTFA)

1H NMR (400 MHz, CDCl3)

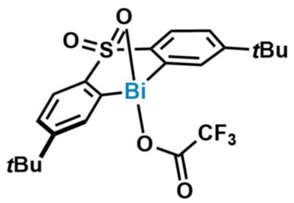

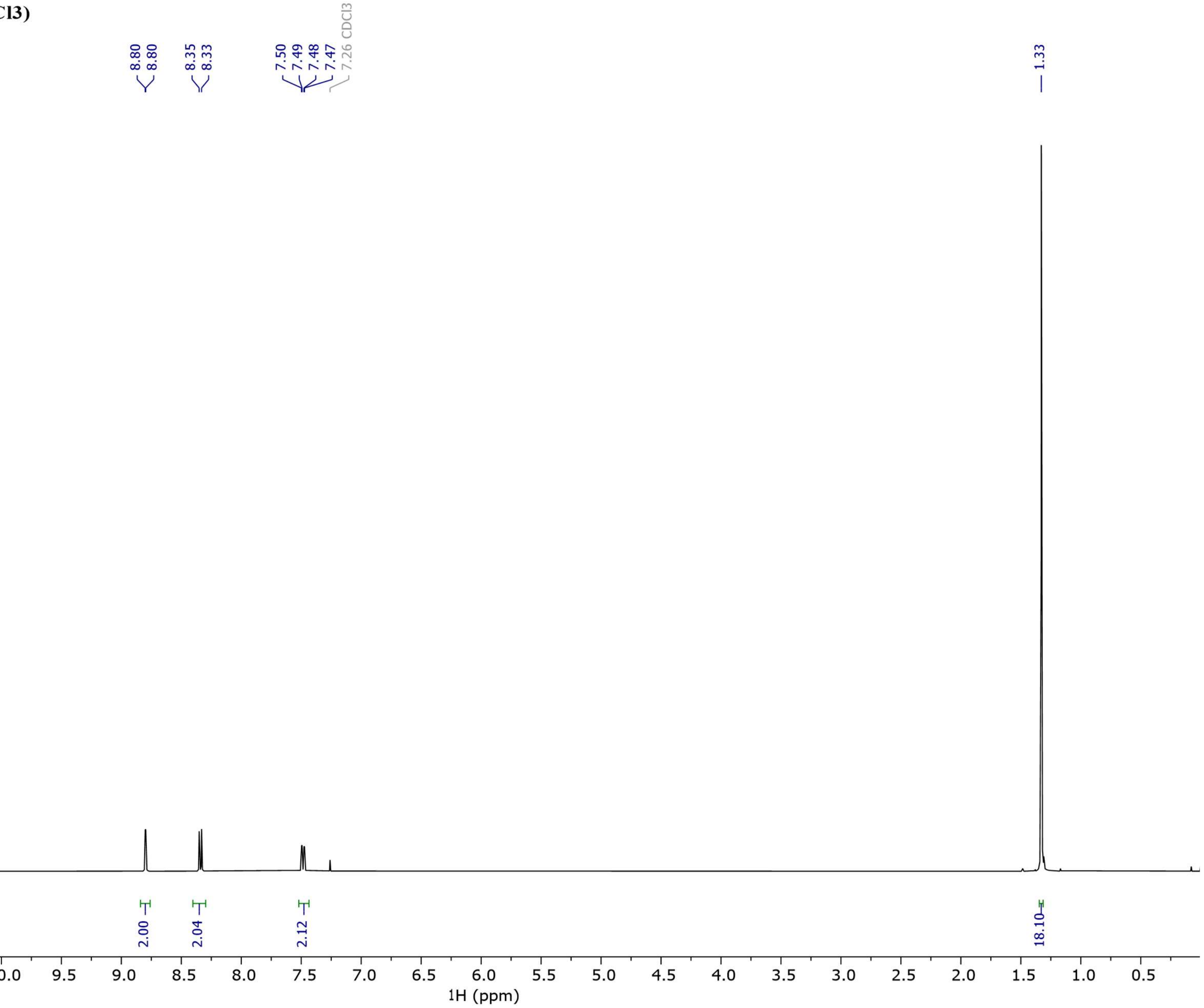

13C NMR (100 MHz, CDCl3)

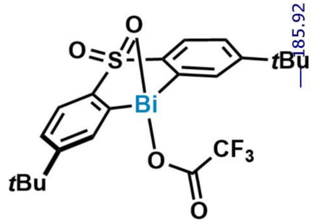

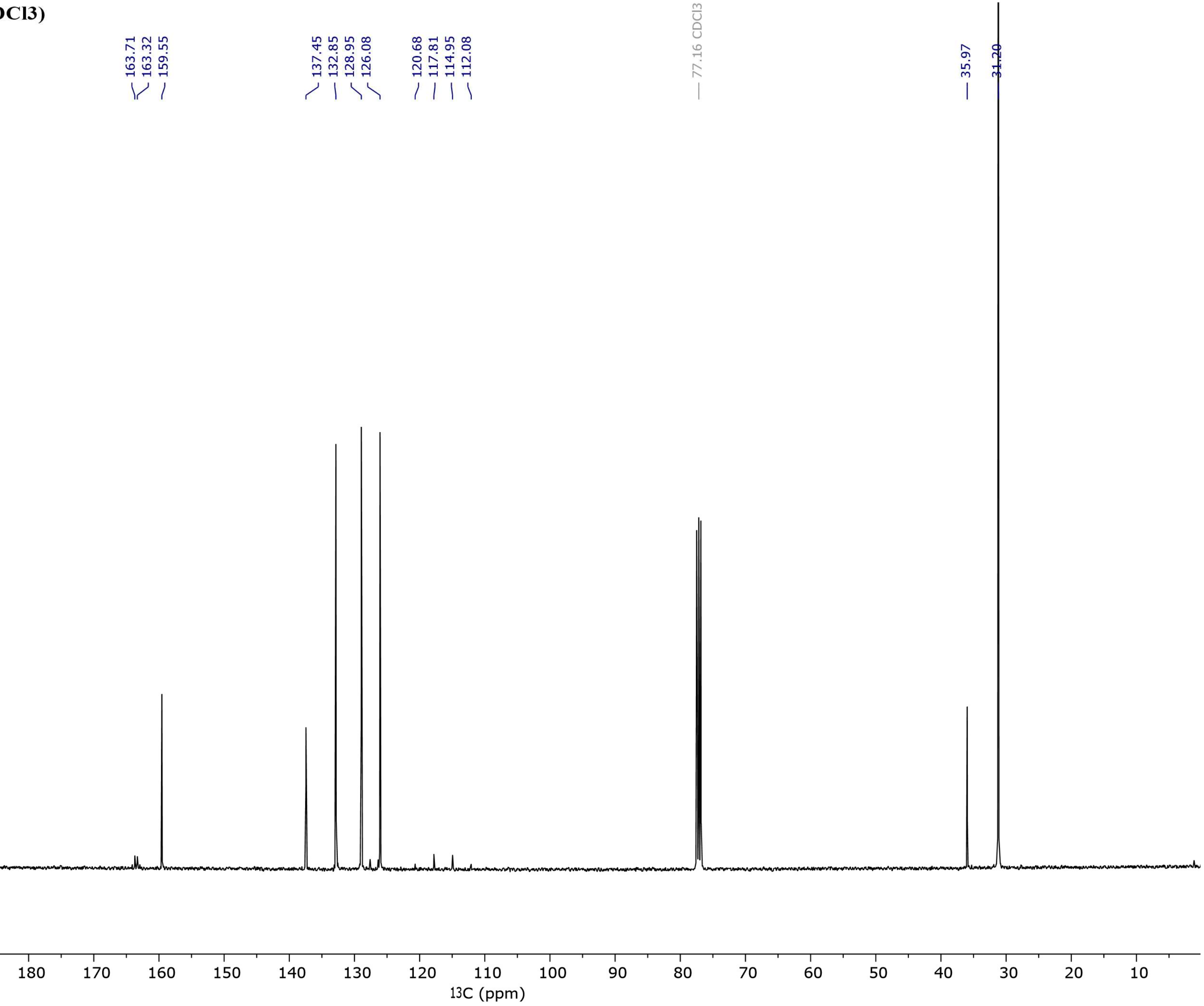

19F NMR (282 MHz, CDCl3)

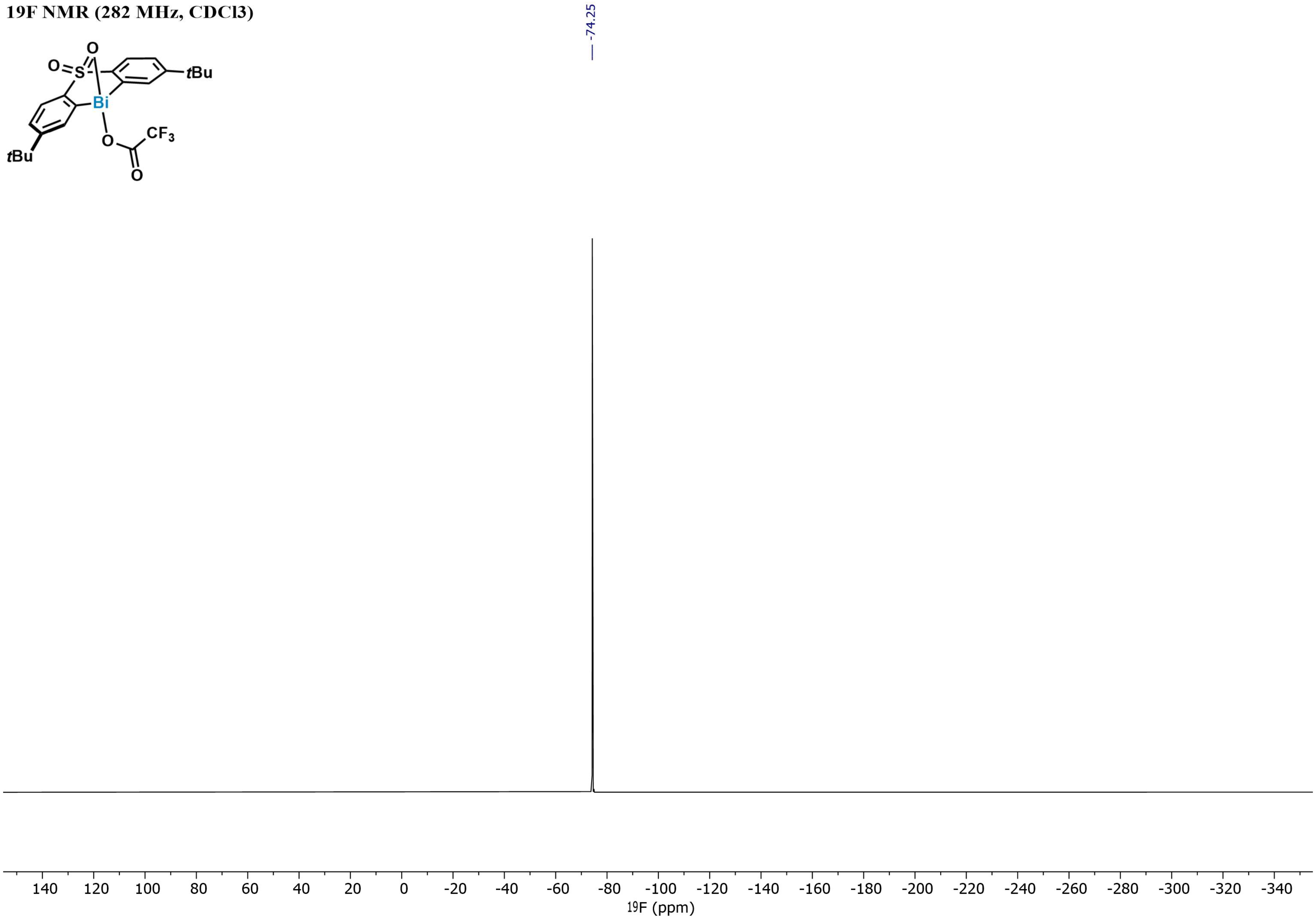

5,5-dioxido-10H-dibenzo[b,e][1,4]thiabismin-10-yl 2,2,2-trifluoroacetate (Bi-4·OTFA)

1H NMR (600 MHz, CDCl3)

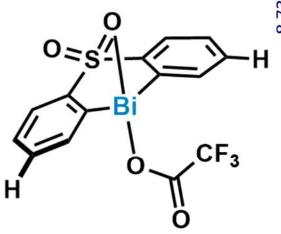

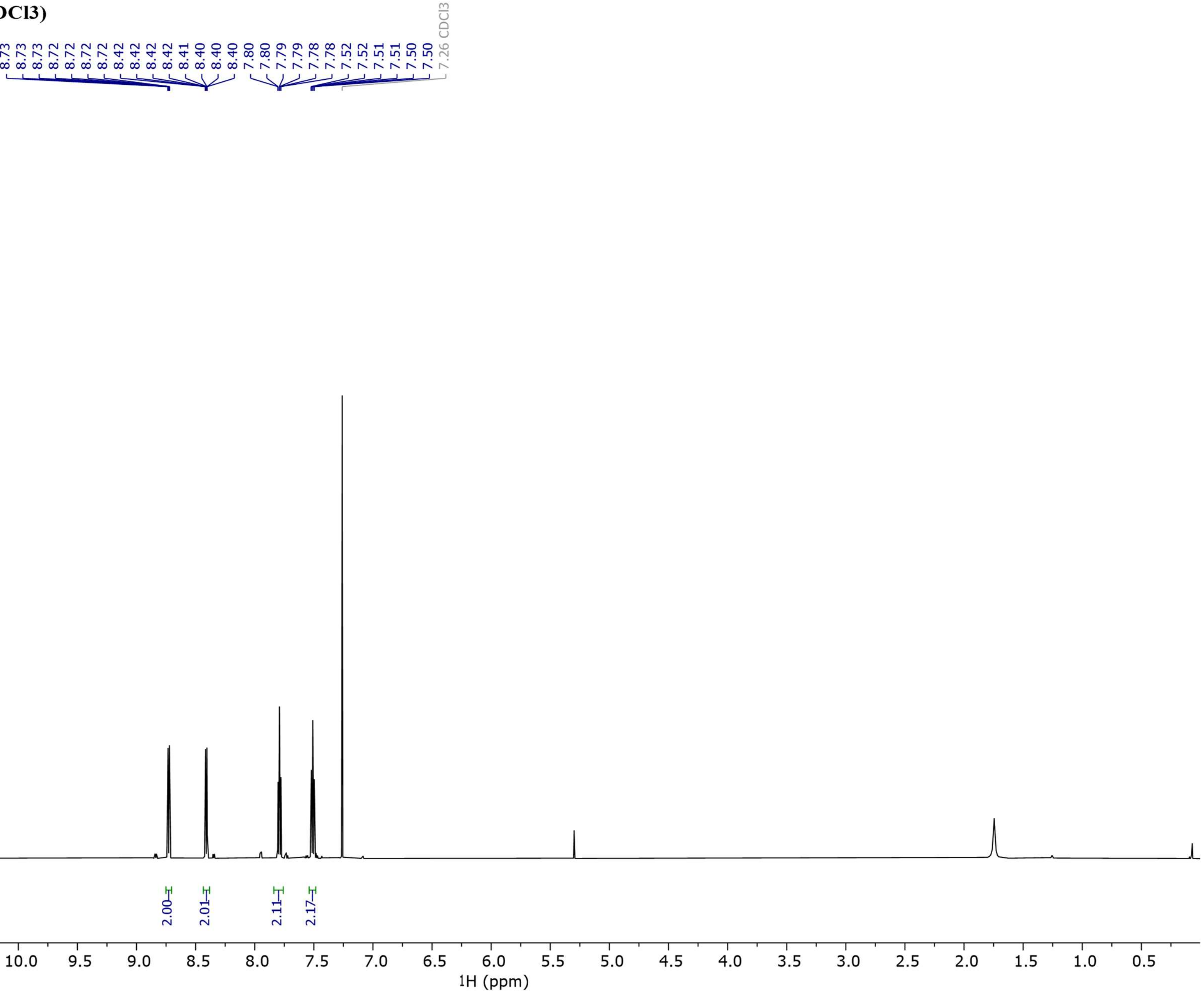

13C NMR (150 MHz, CDCl3)

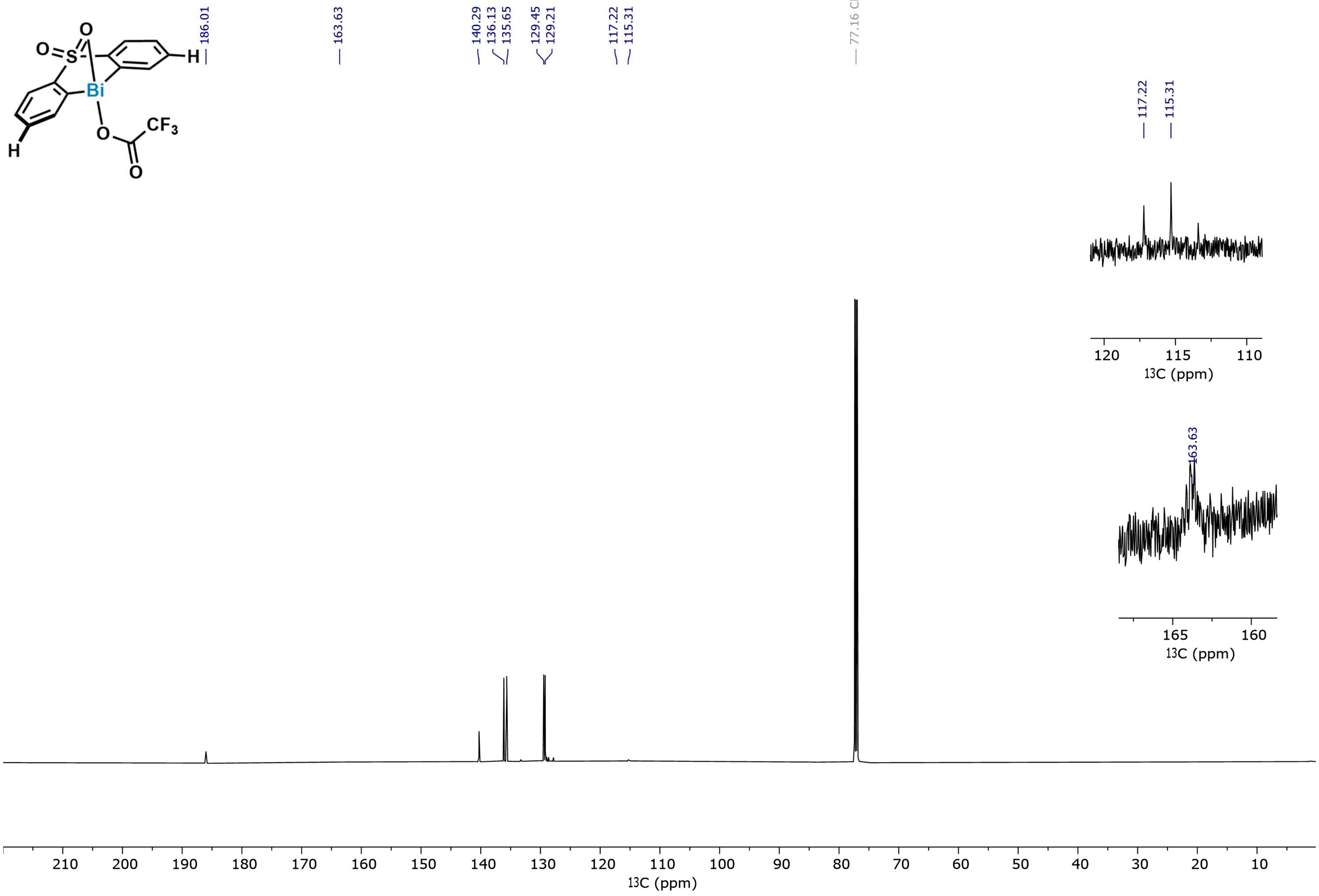

19F NMR (564 MHz, CDCl3)

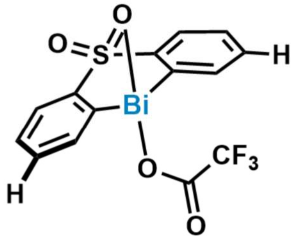

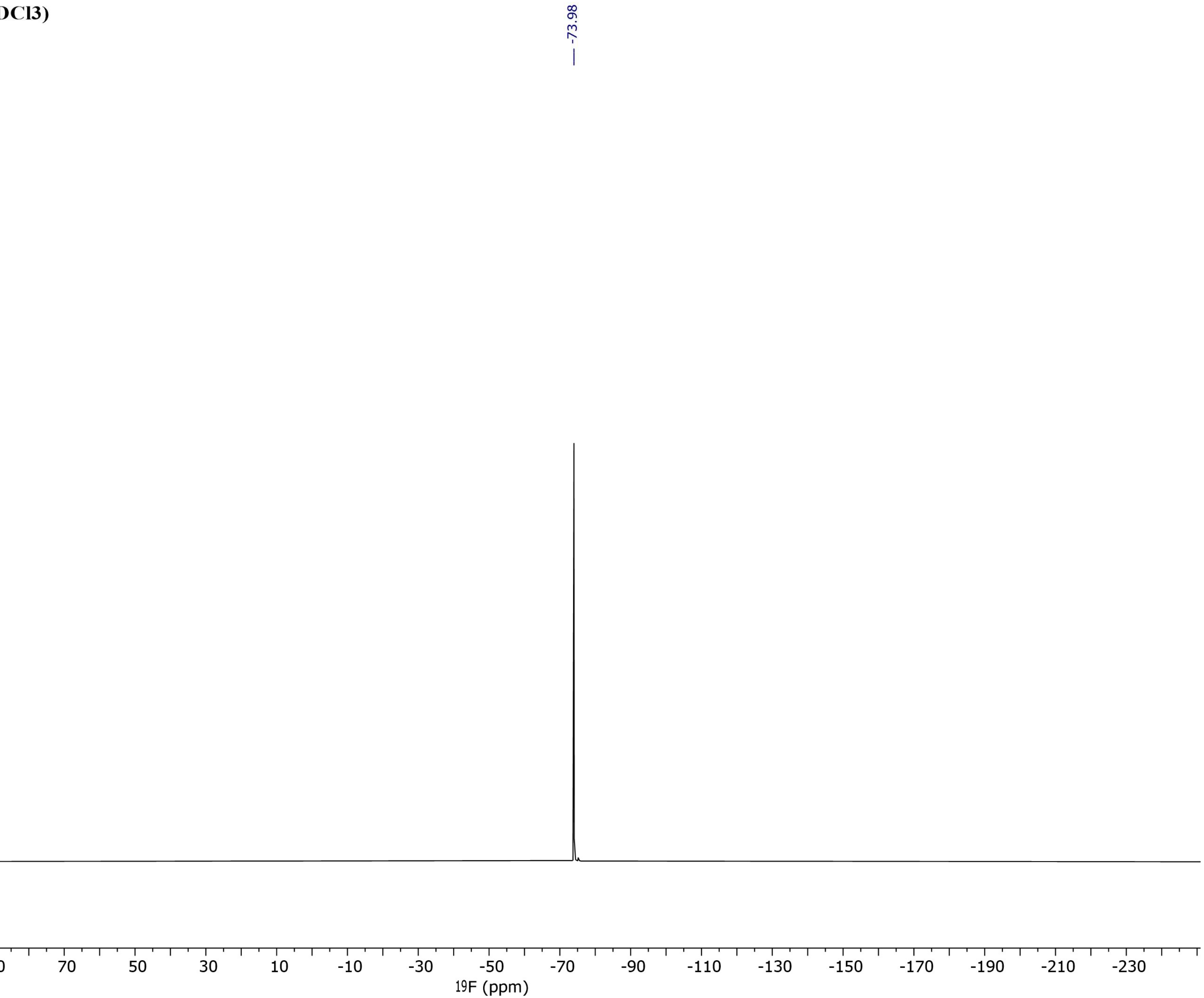

2-methyl-5,5-dioxido-10H-dibenzo[b,e][1,4]thiabismin-10-yl 2,2,2-trifluoroacetate (Bi-7·OTFA)

1H NMR (300 MHz, CDCl3)

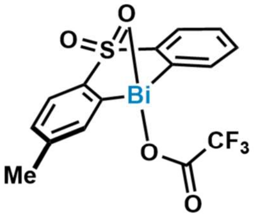

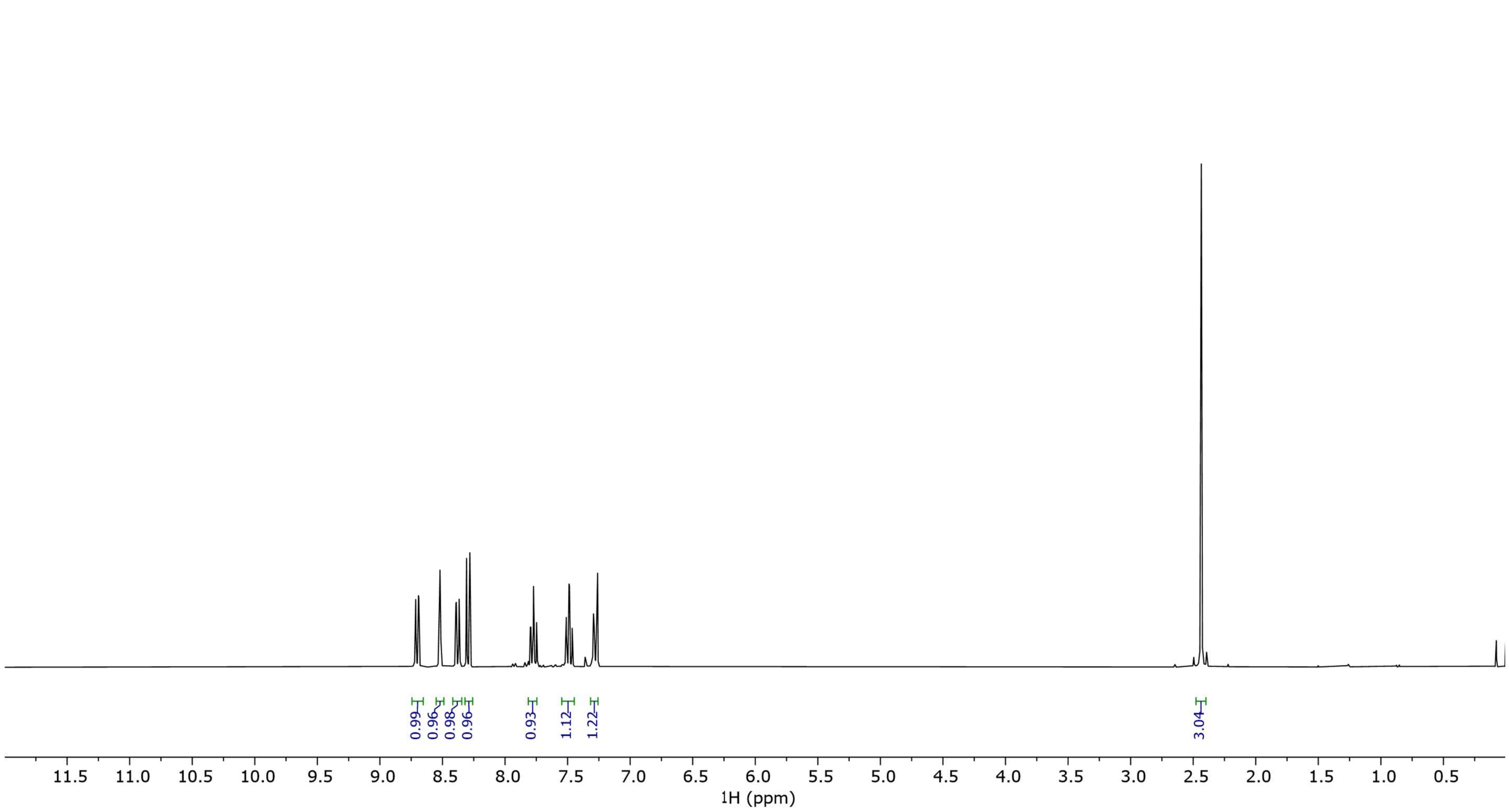

13C NMR (75. MHz, CDCl3)

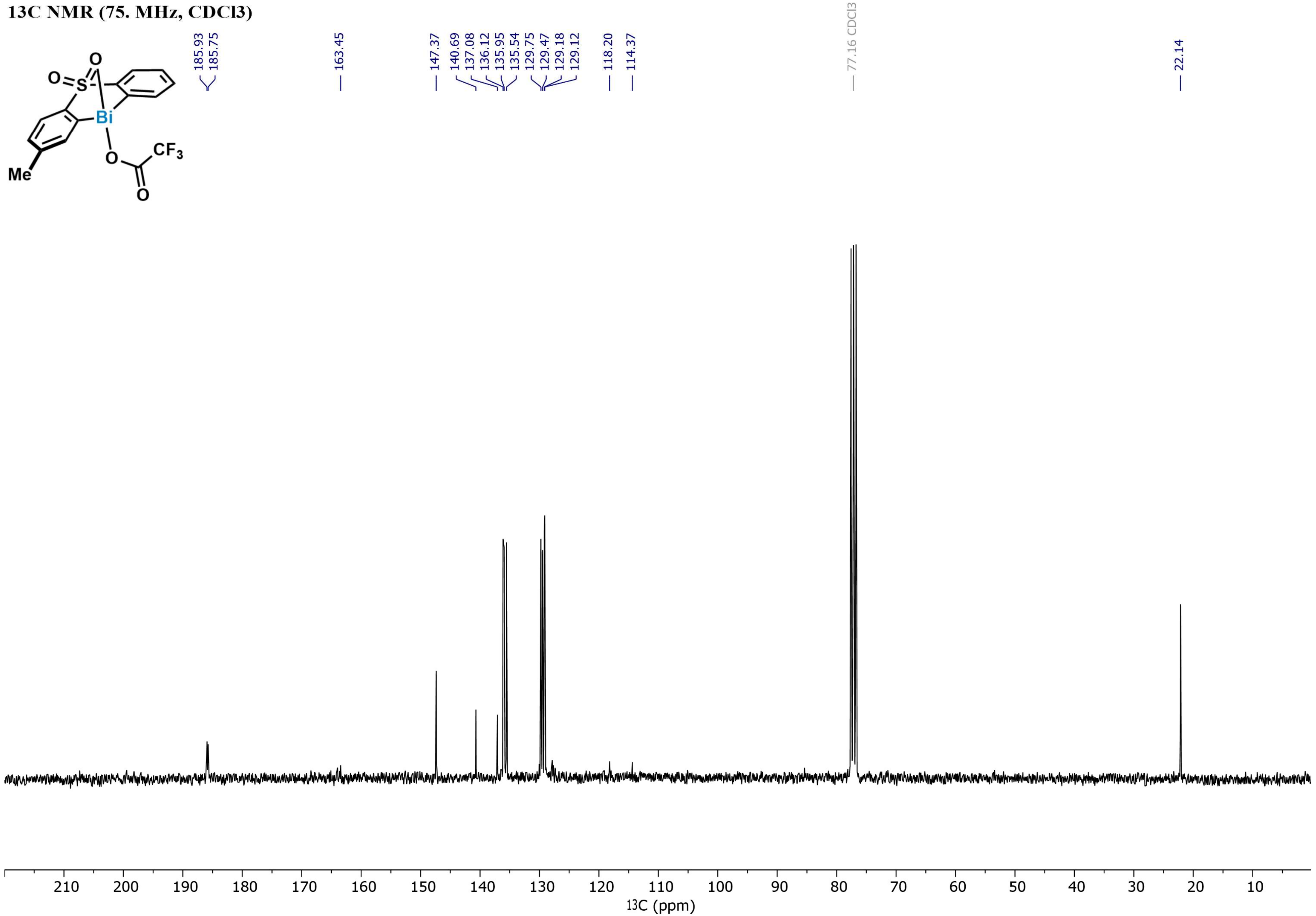

19F NMR (282 MHz, CDCl3)

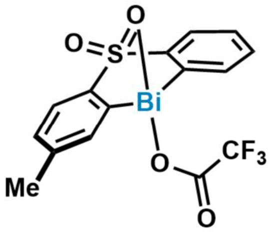

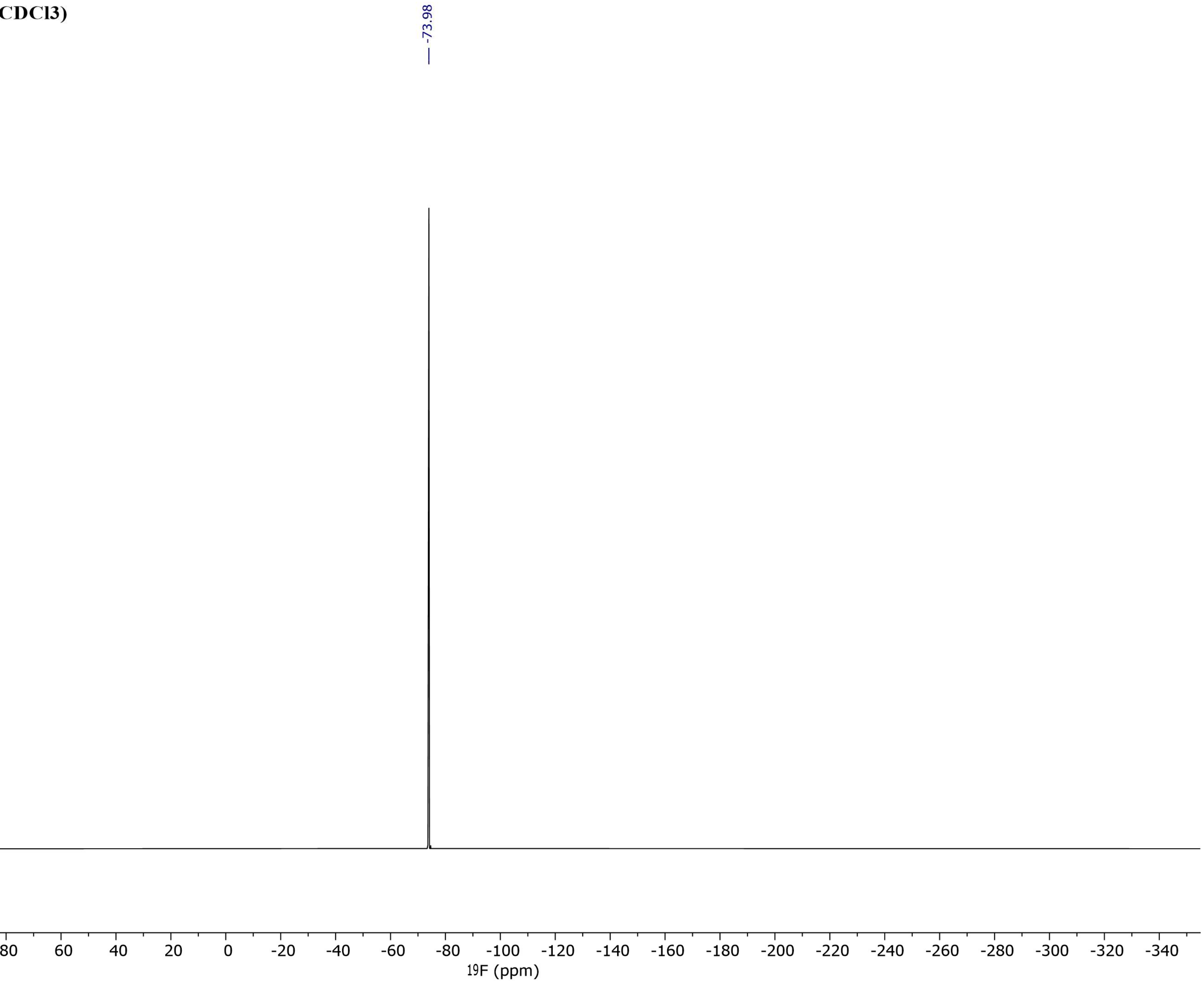

2,8-difluoro-5,5-dioxido-10H-dibenzo[b,e][1,4]thiabismin-10-yl 2,2,2-trifluoroacetate (Bi-8·OTFA)

1H NMR (400 MHz, CDCl3)

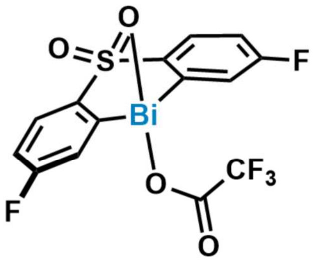

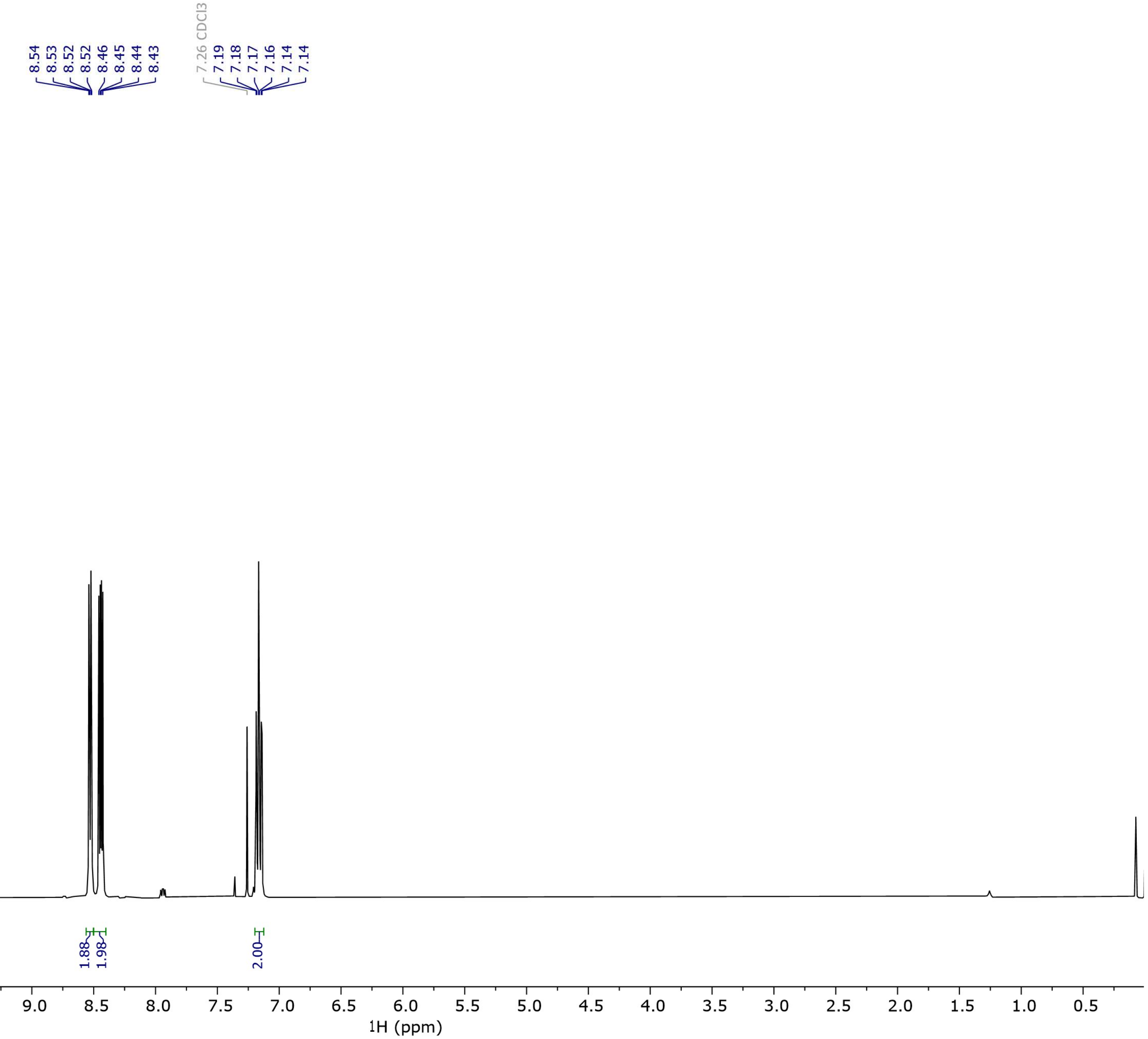

13C NMR (100 MHz, CDCl3)

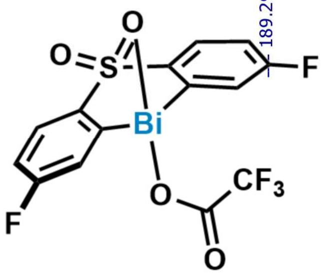

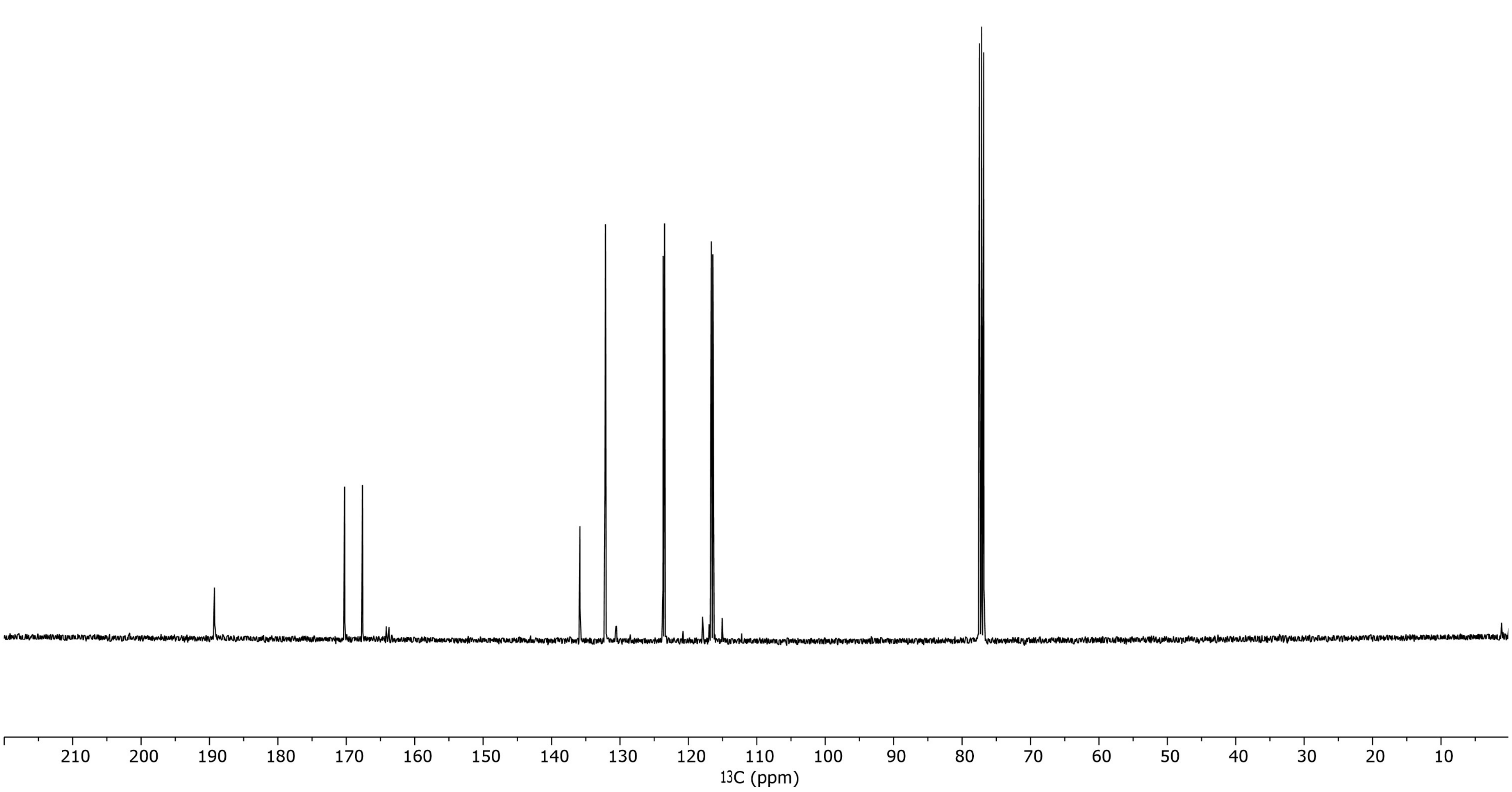

19F NMR (282 MHz, CDCl3)

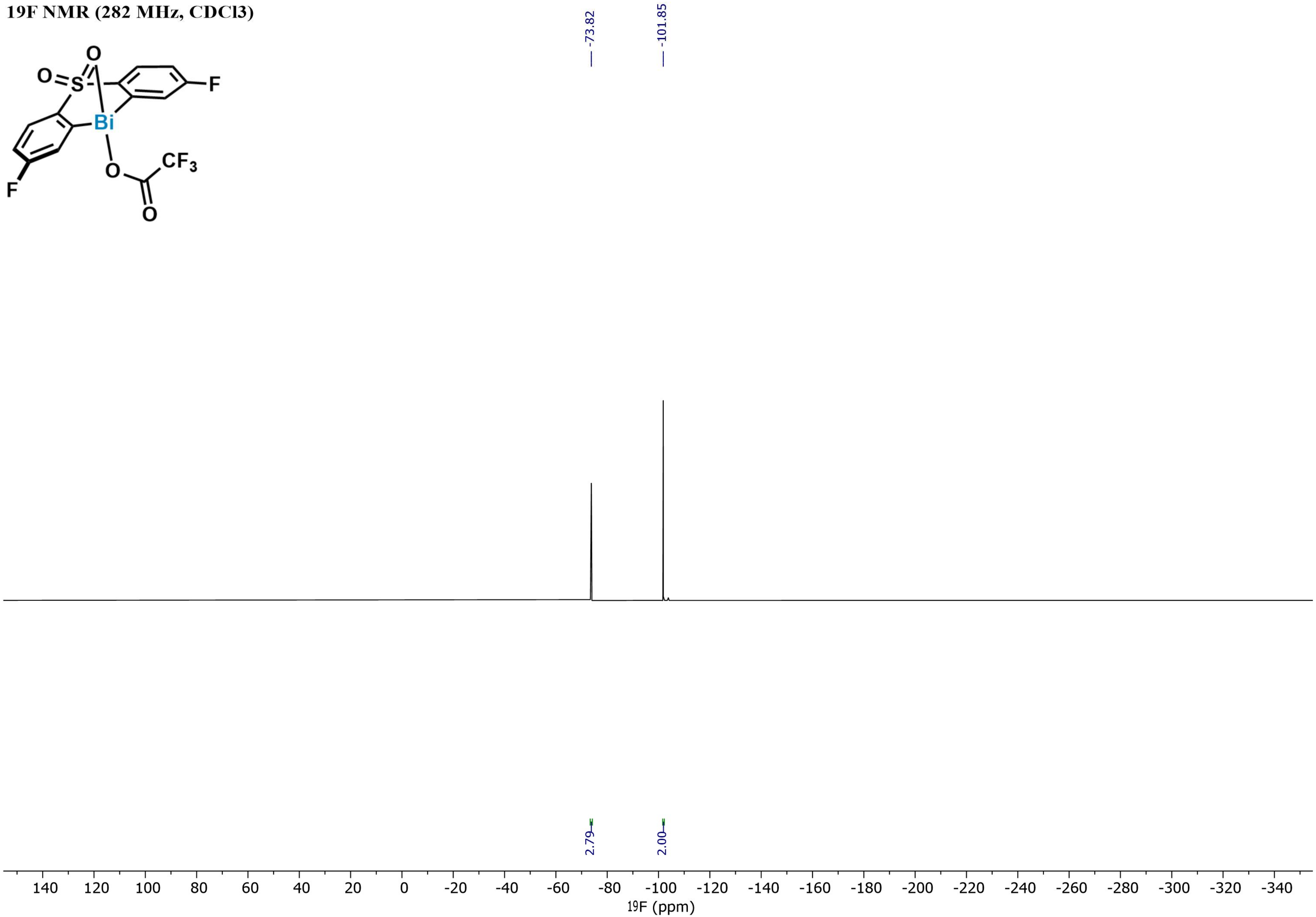

2-methoxy-5,5-dioxido-10H-dibenzo[b,e][1,4]thiabismin-10-yl 2,2,2-trifluoroacetate (Bi-13·OTFA)

1H NMR (300 MHz, CDCl3)

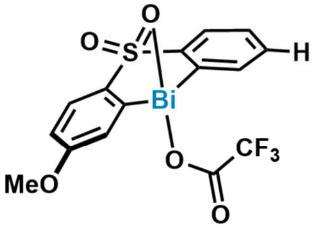

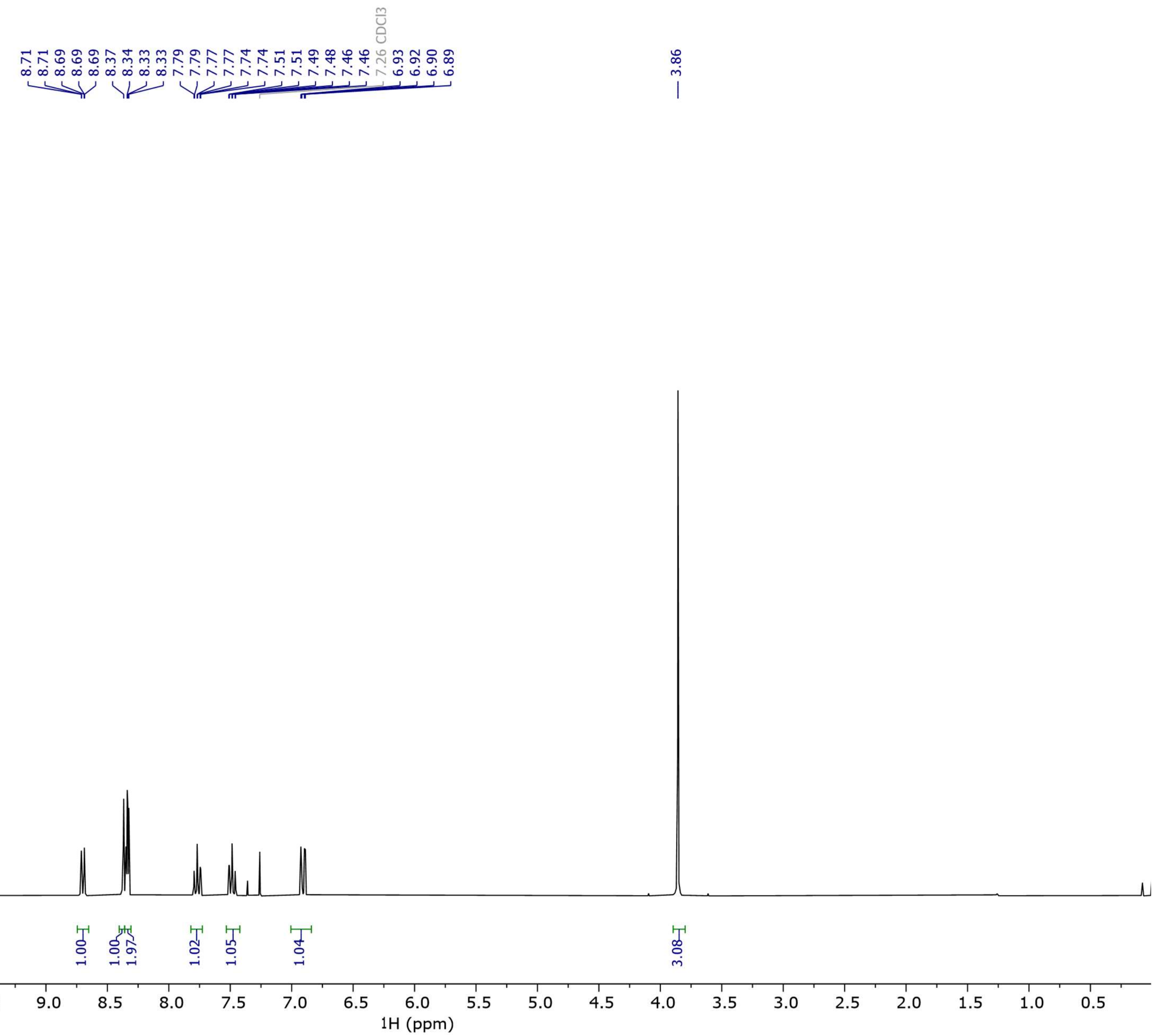

13C NMR (75. MHz, CDCl3)

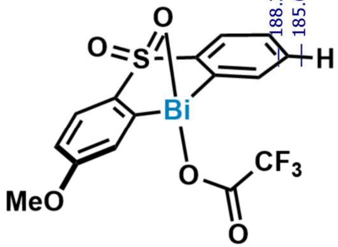

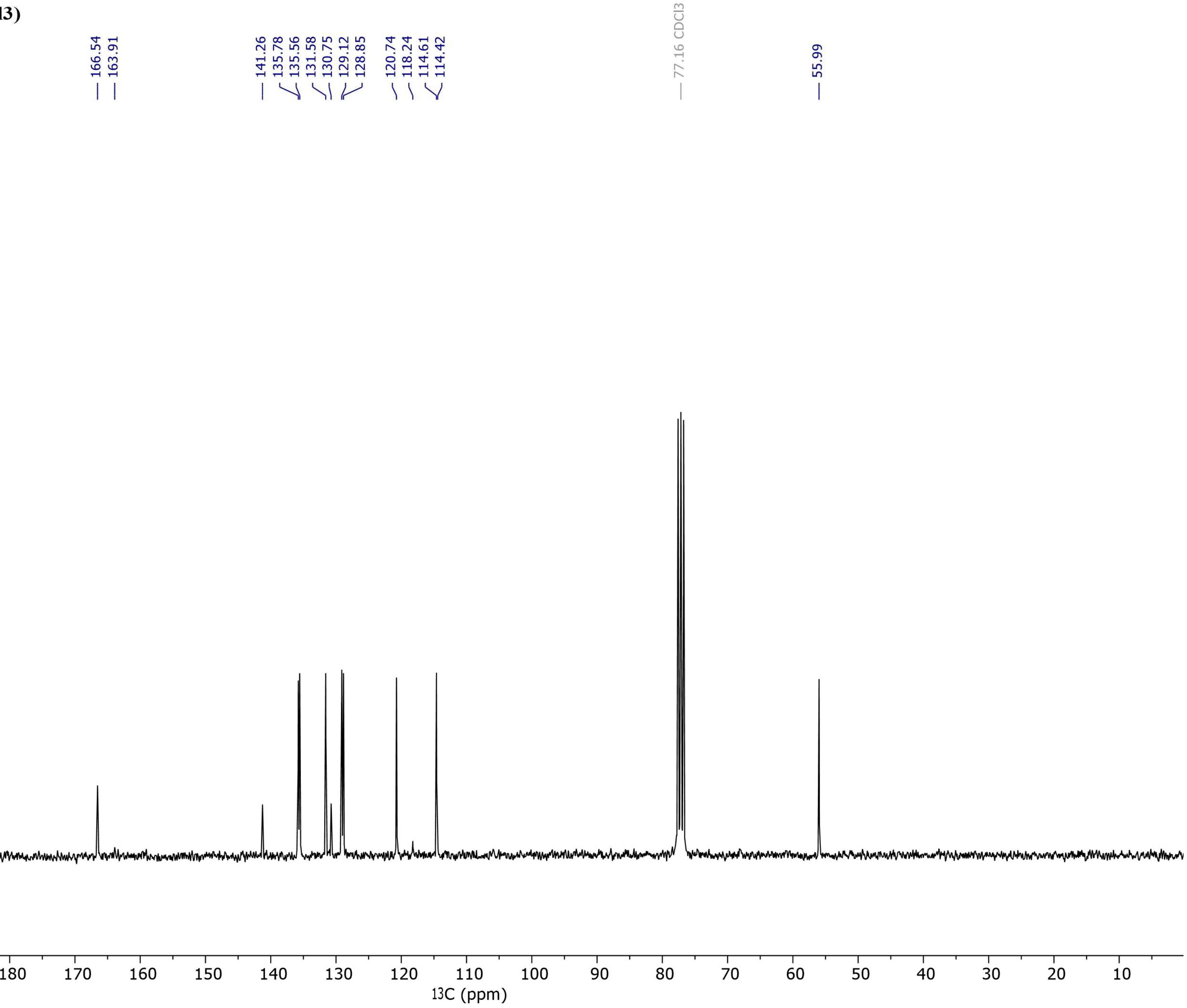

19F NMR (282 MHz, CDCl3)

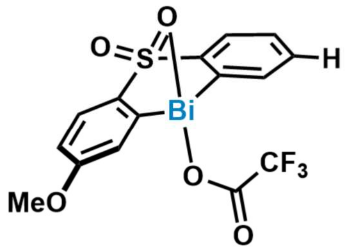

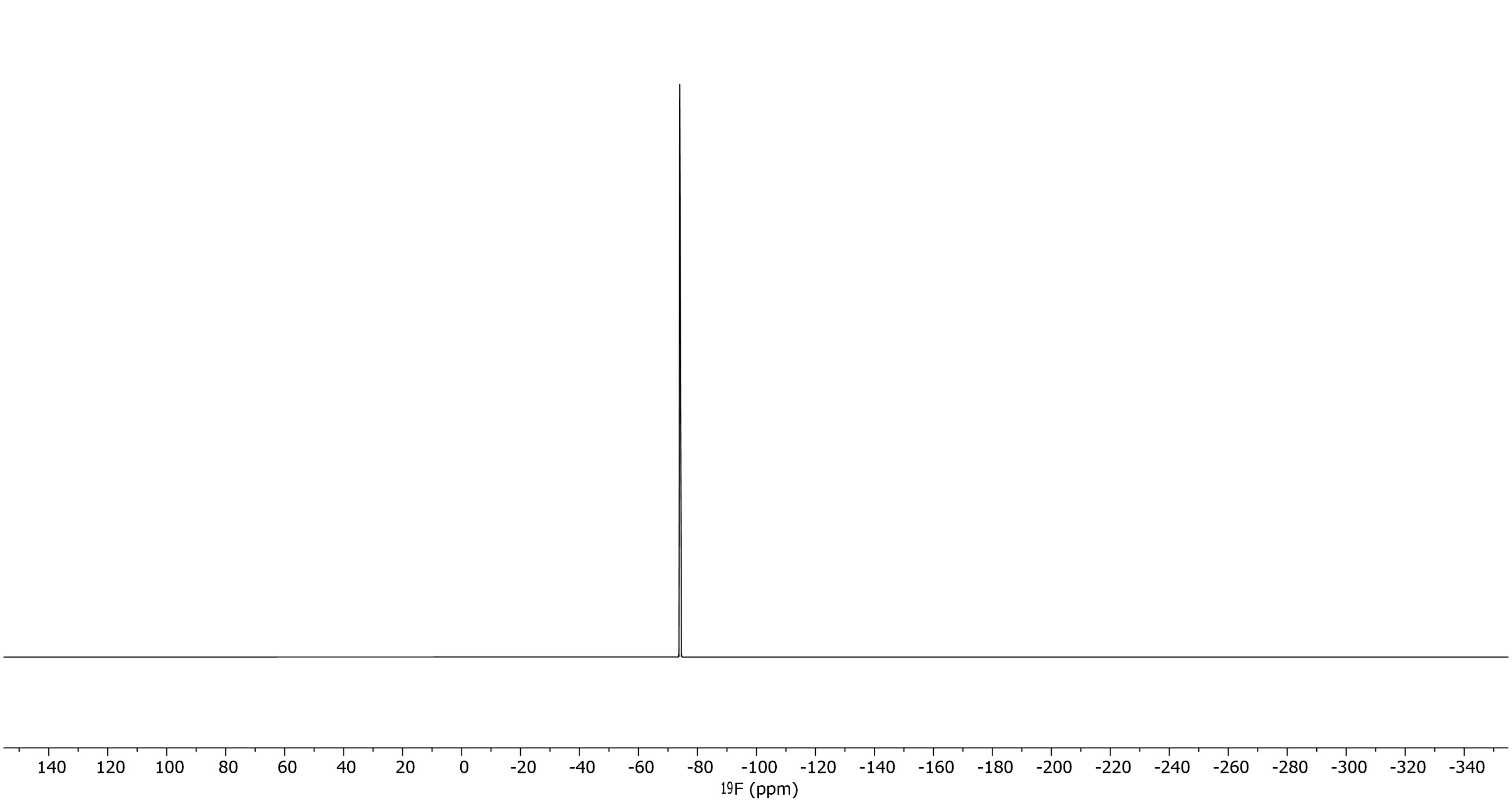

2,8-di-tert-butyl-10-chloro-10H-dibenzo[b,e][1,4]thiabismine 5,5-dioxide (Bi-2·Cl)
1H NMR (600 MHz, CDCl3)

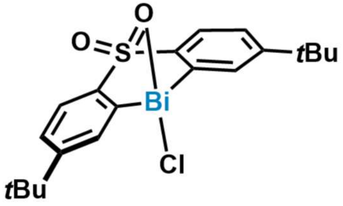

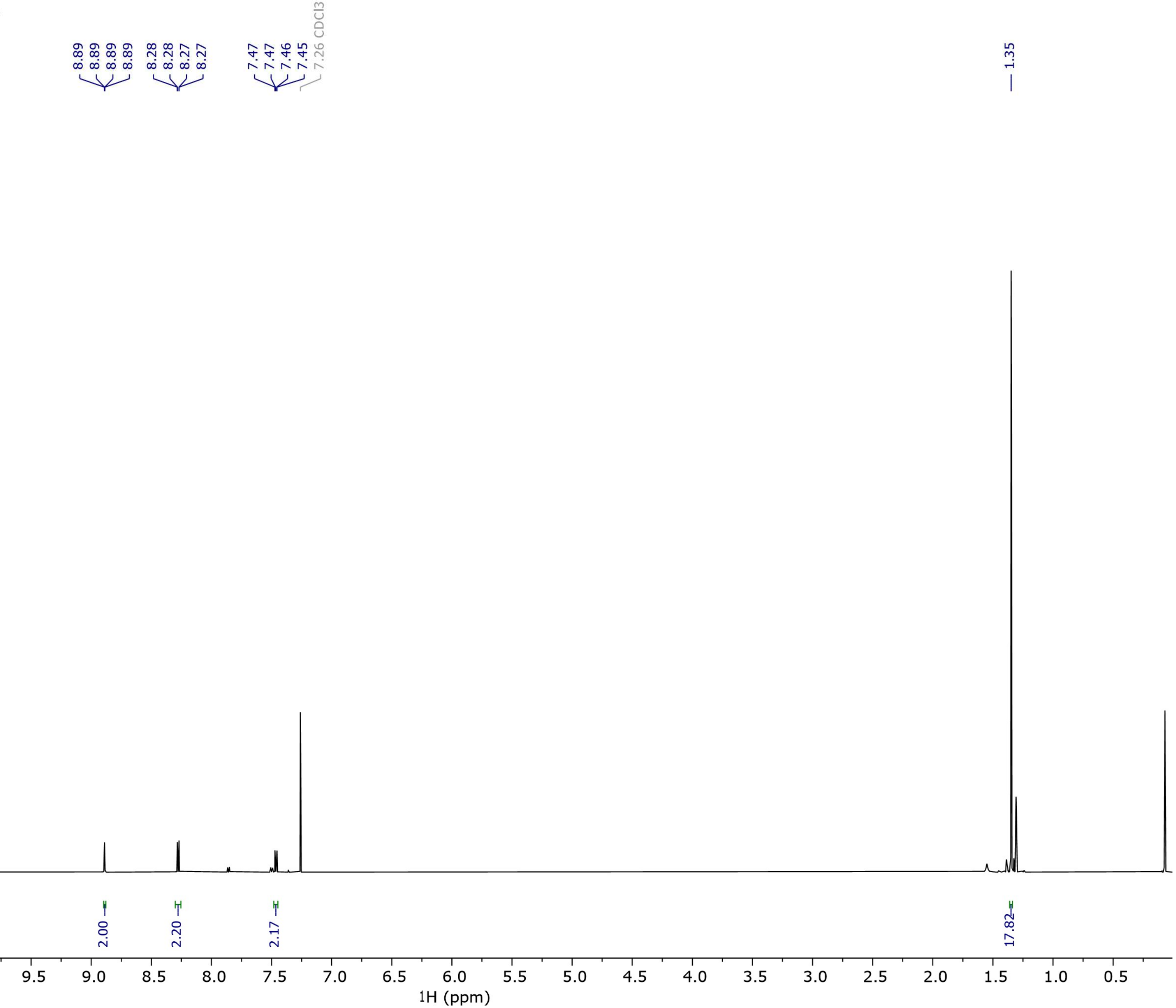

13C NMR (150 MHz, CDCl3)

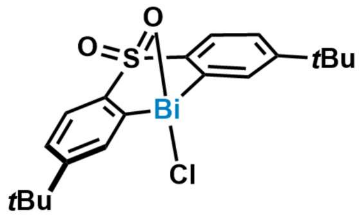

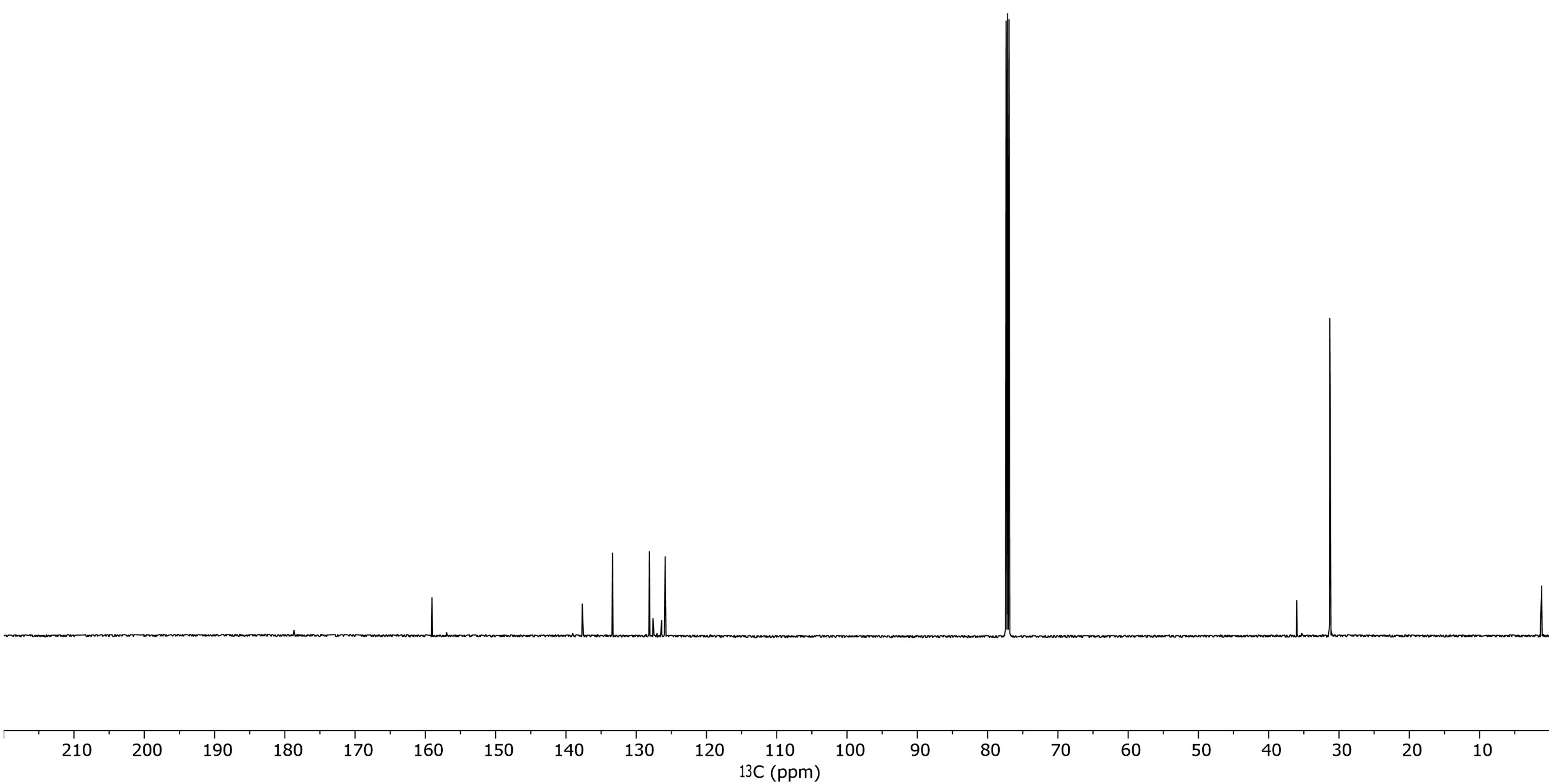

10-chloro-5-(methylimino)-5,10-dihydro-5l4-dibenzo[b,e][1,4]thiabismine 5-oxide (Bi-14·Cl)

1H NMR (600 MHz, DMSO)

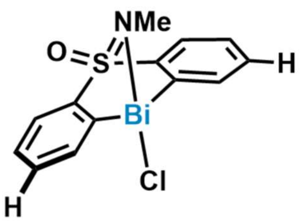

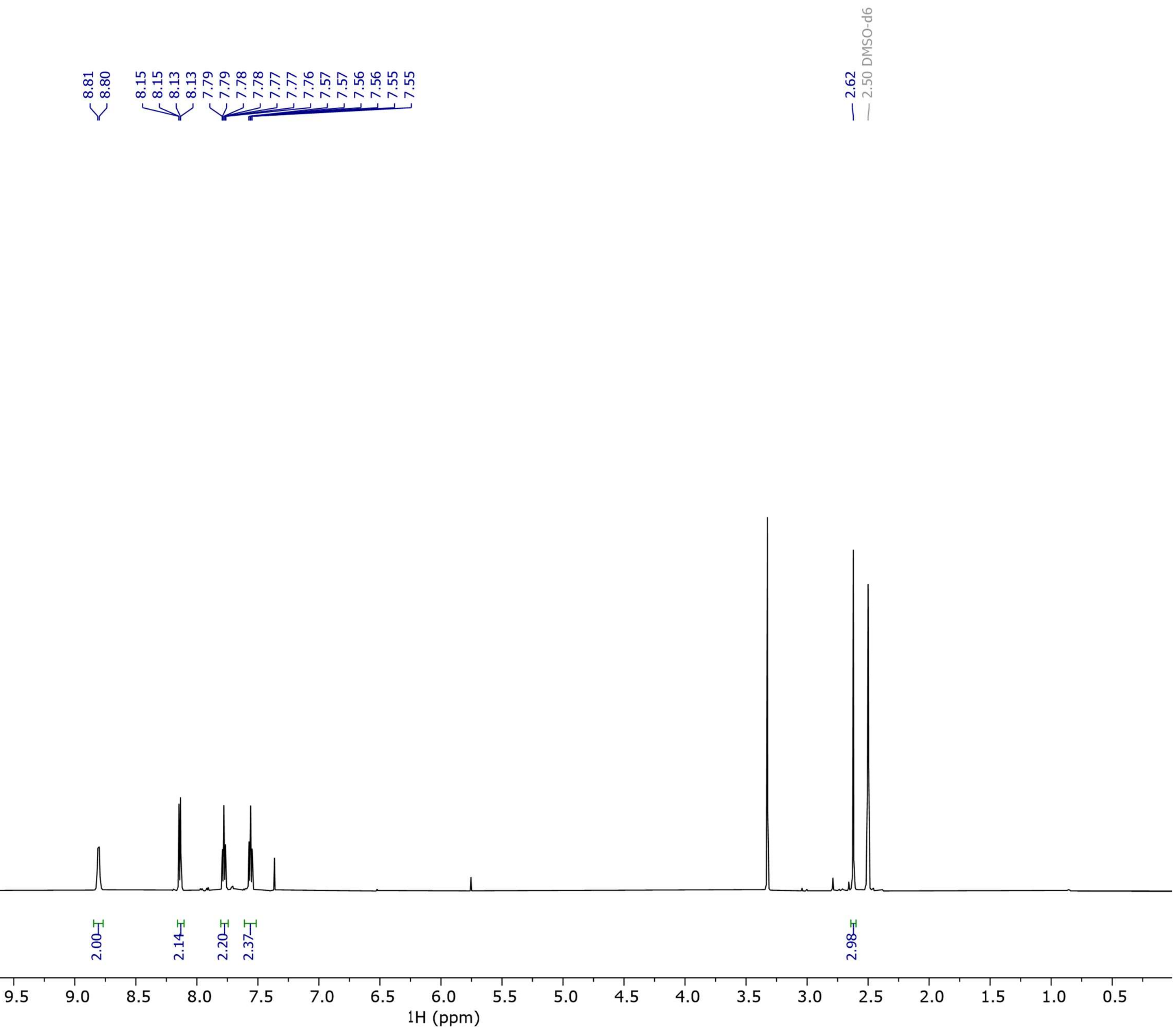

13C NMR (150 MHz, DMSO)

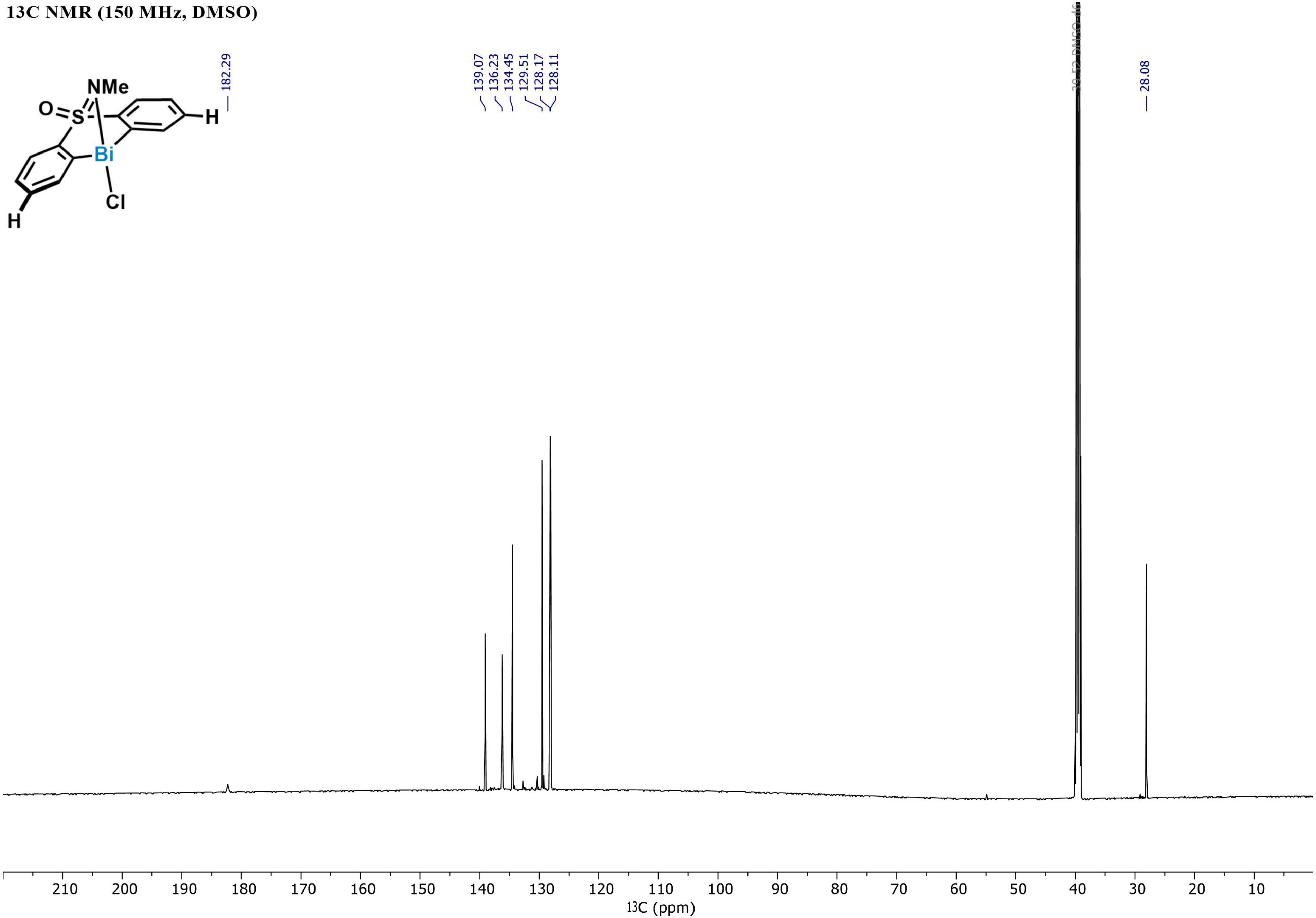

10-bromo-2-methyl-8-(trifluoromethyl)-10H-dibenzo[b,e][1,4]thiabismine 5,5-dioxide (Bi-1·Br)

1H NMR (600 MHz, CDCl3)

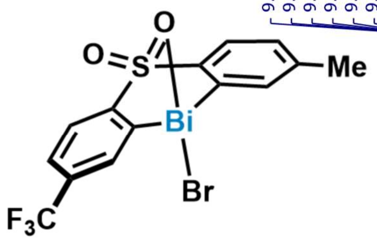

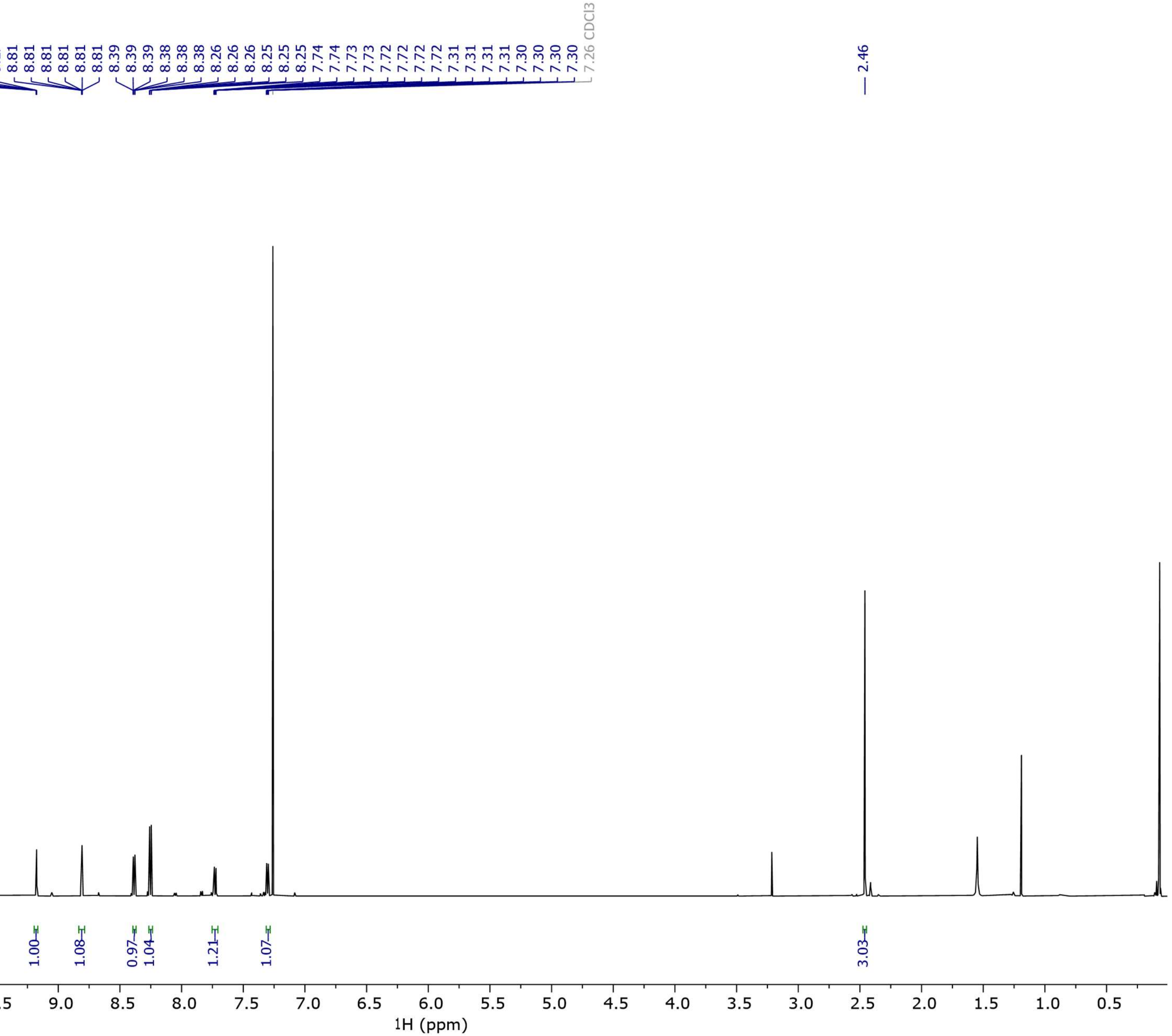

13C NMR (150 MHz, CDCl3)

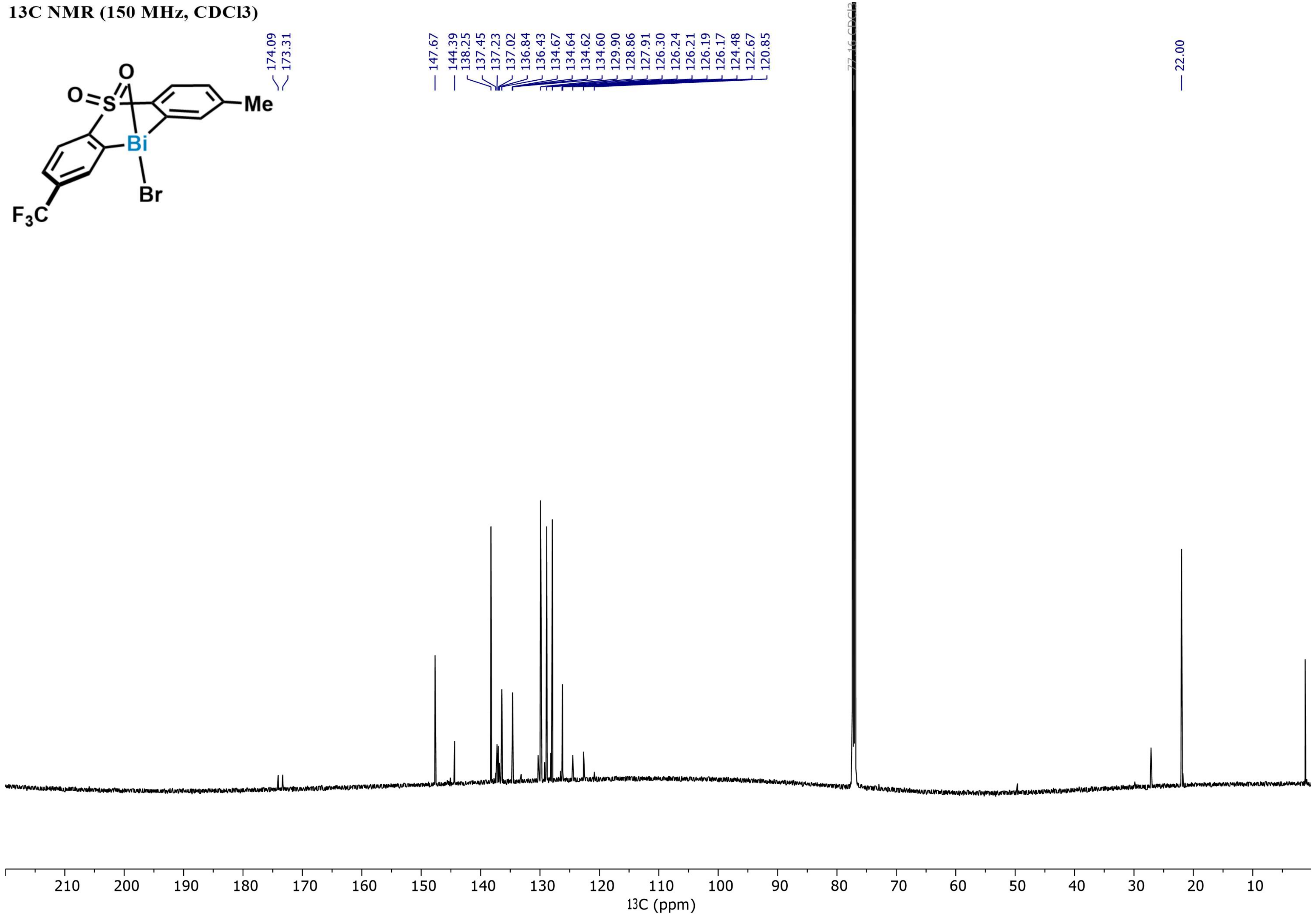

19F NMR (564 MHz, CDCl3)

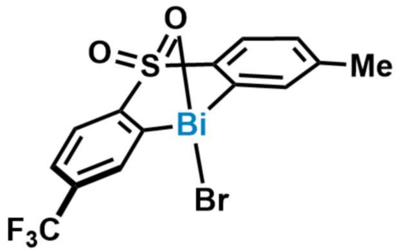

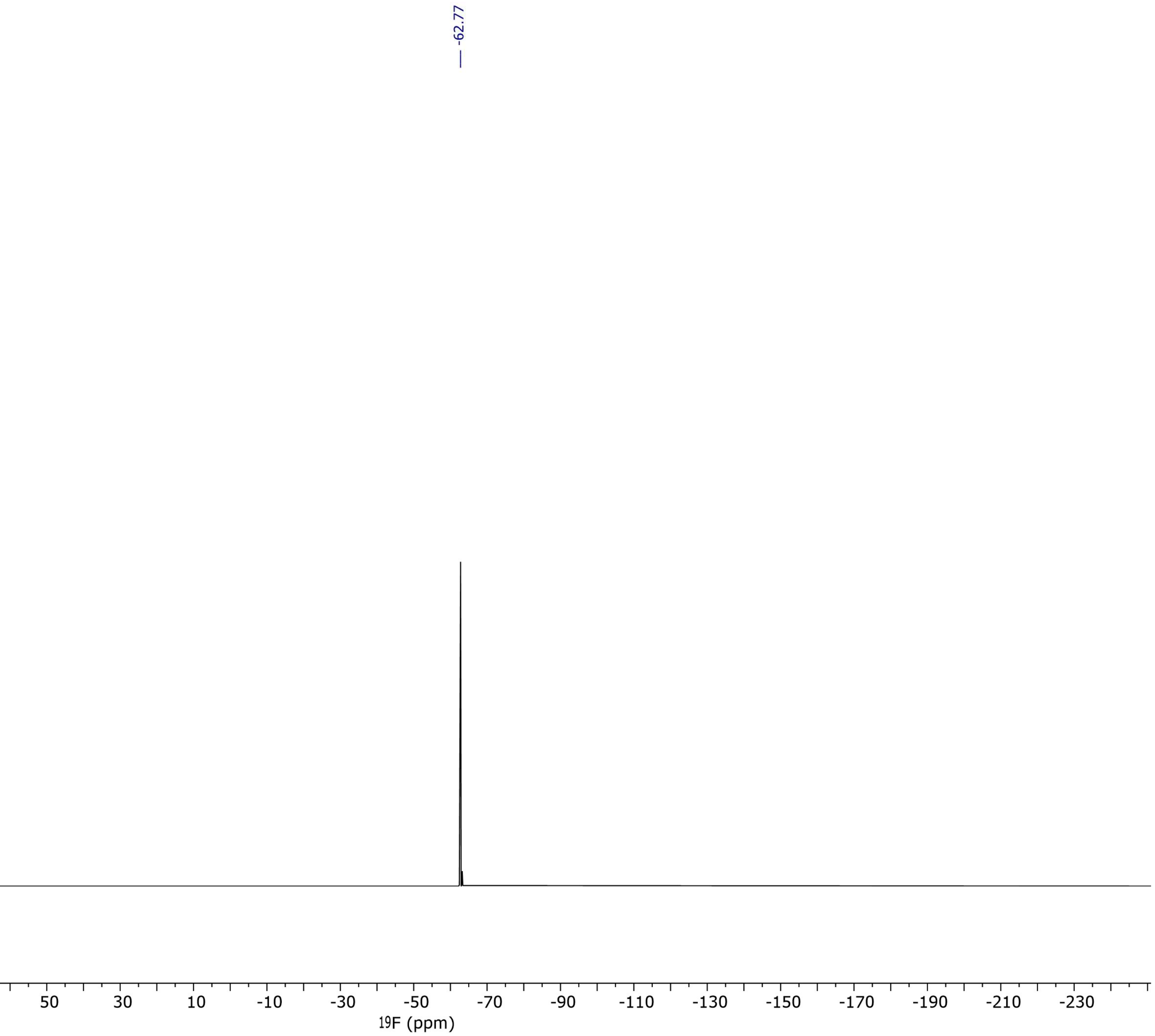

N-(2-methyl-5,5-dioxido-8-(trifluoromethyl)-10H-dibenzo[b,e][1,4]thiabismin-10-yl)-N-(phenylsulfonyl)benzenesulfonamide Bi-1·NSI

1H NMR (300 MHz, CDCl3)

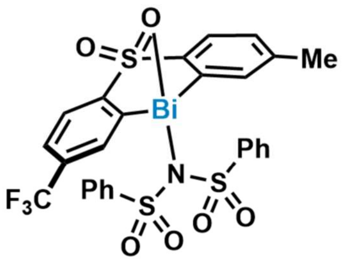

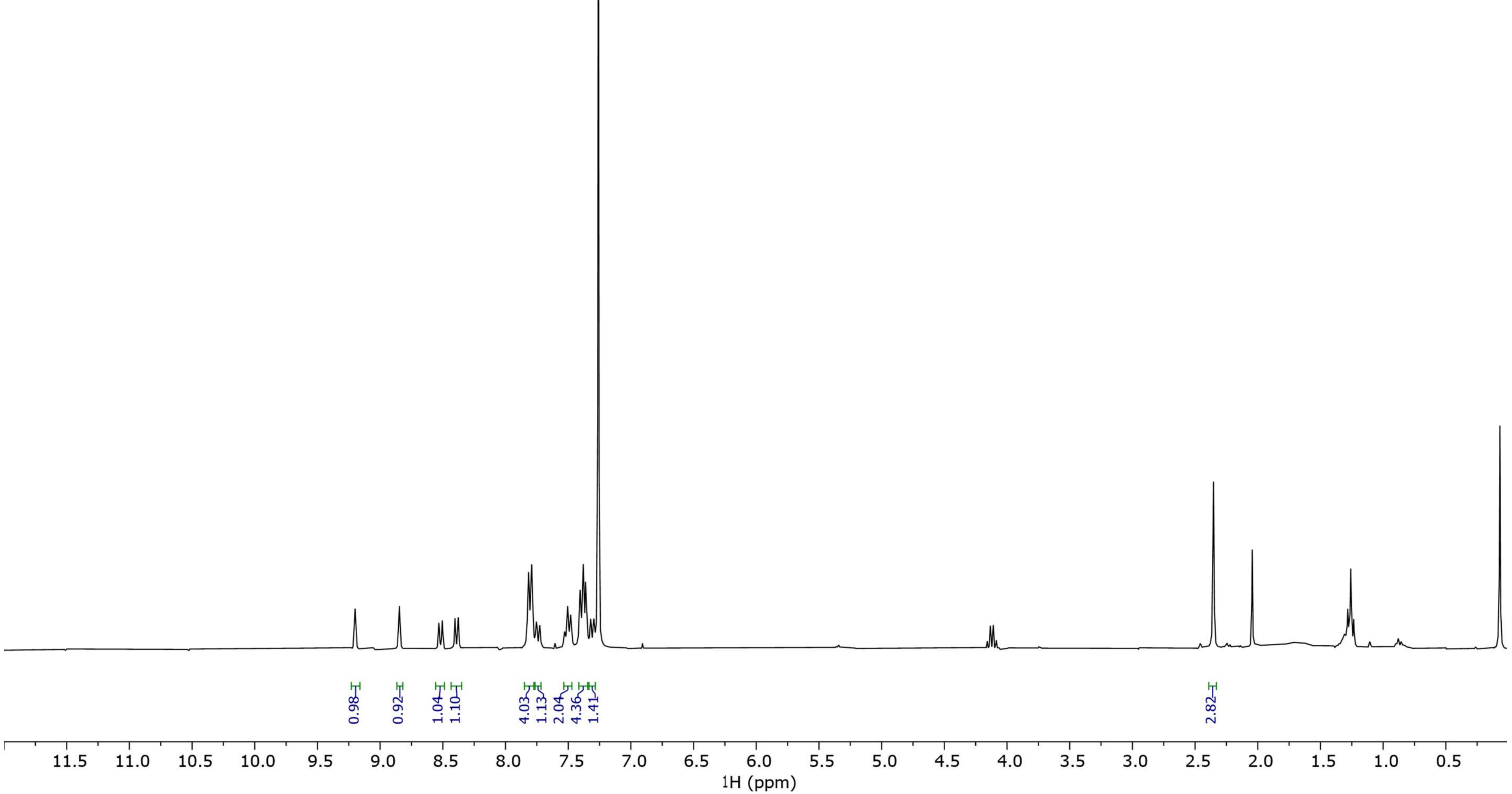

13C NMR (100 MHz, CDCl3)

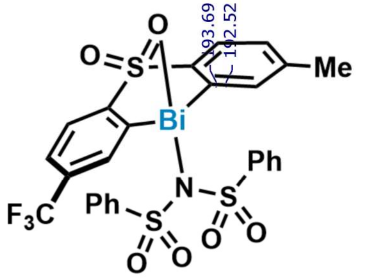

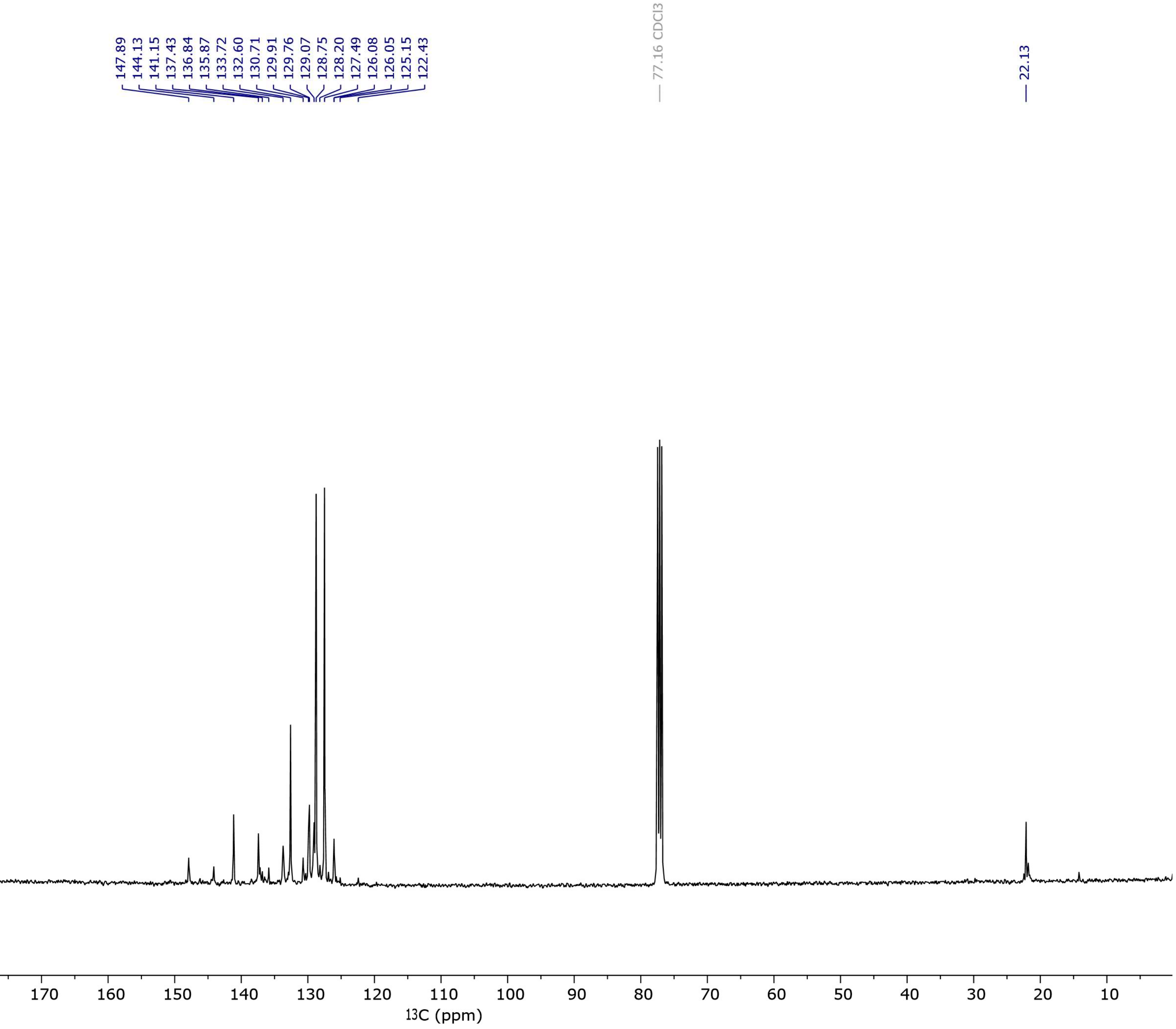

19F NMR (282 MHz, CDCl3)

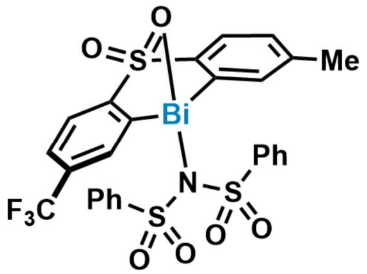

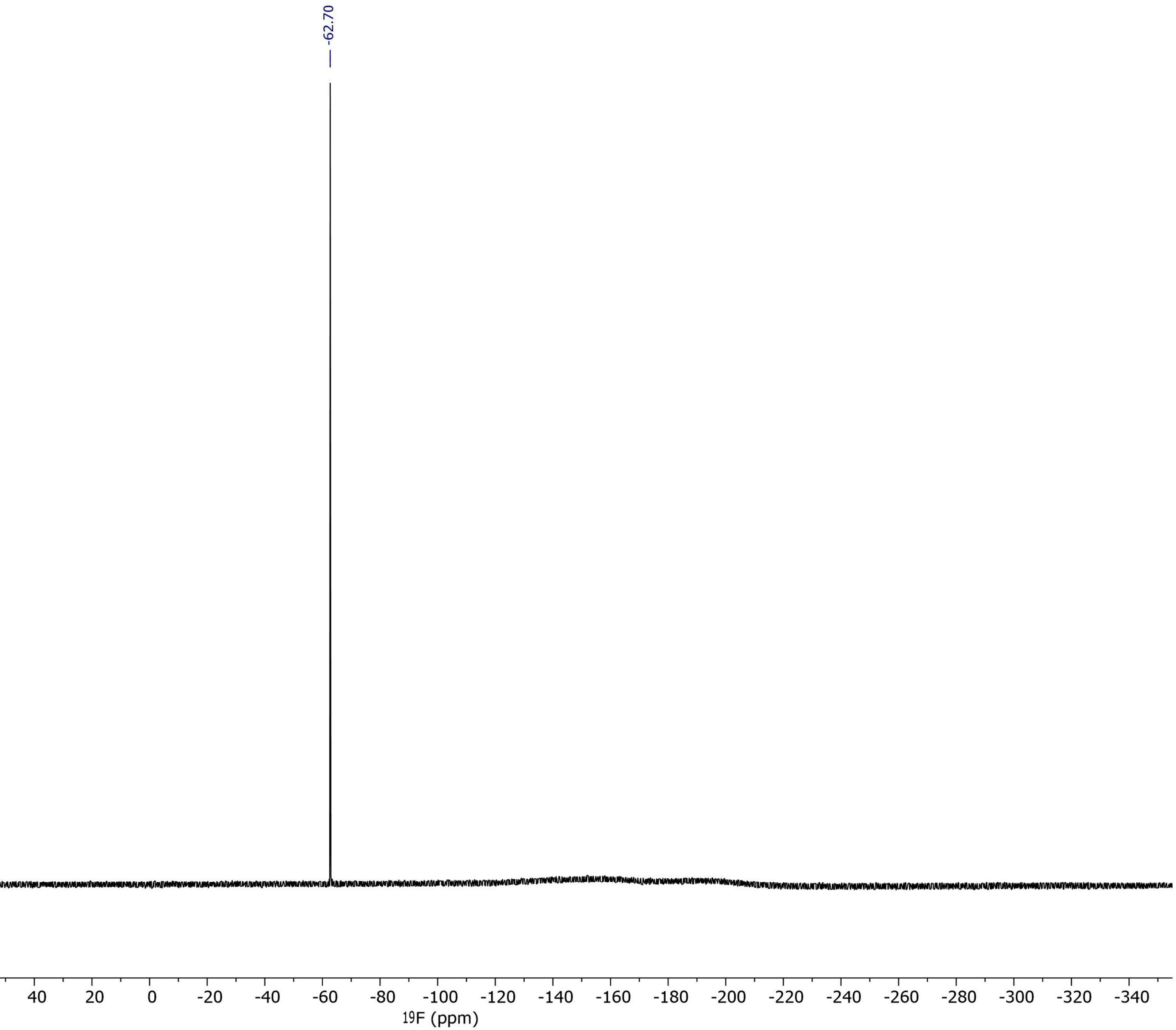

N-(4-(tert-butyl)phenyl)-N-(phenylsulfonyl)benzenesulfonamide (2a)

1H NMR (600 MHz, CDCl3)

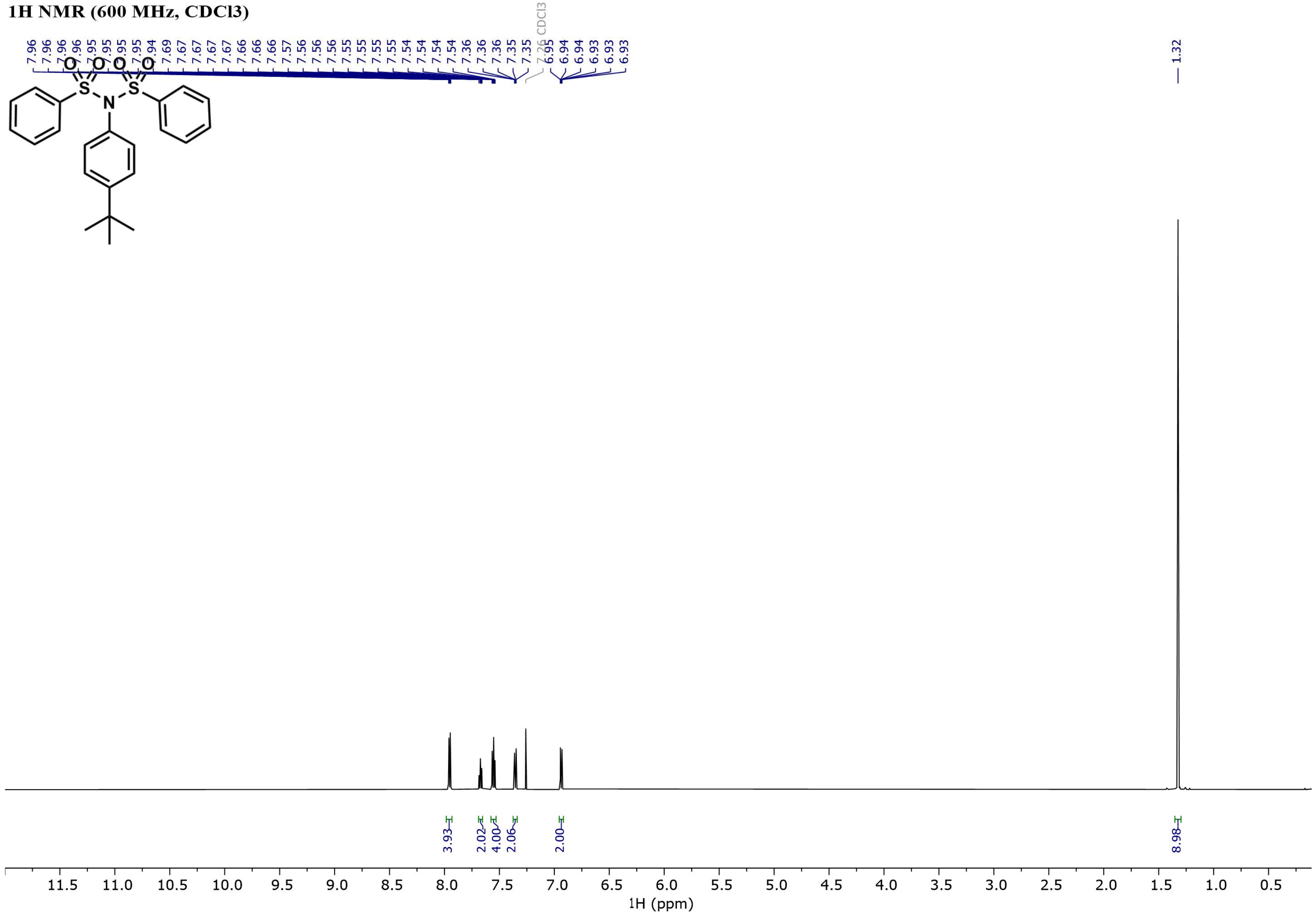

13C NMR (150 MHz, CDCl3)

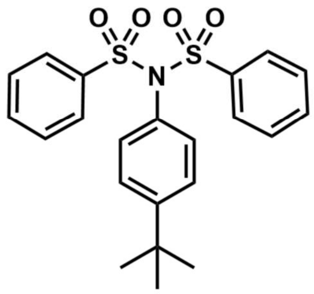

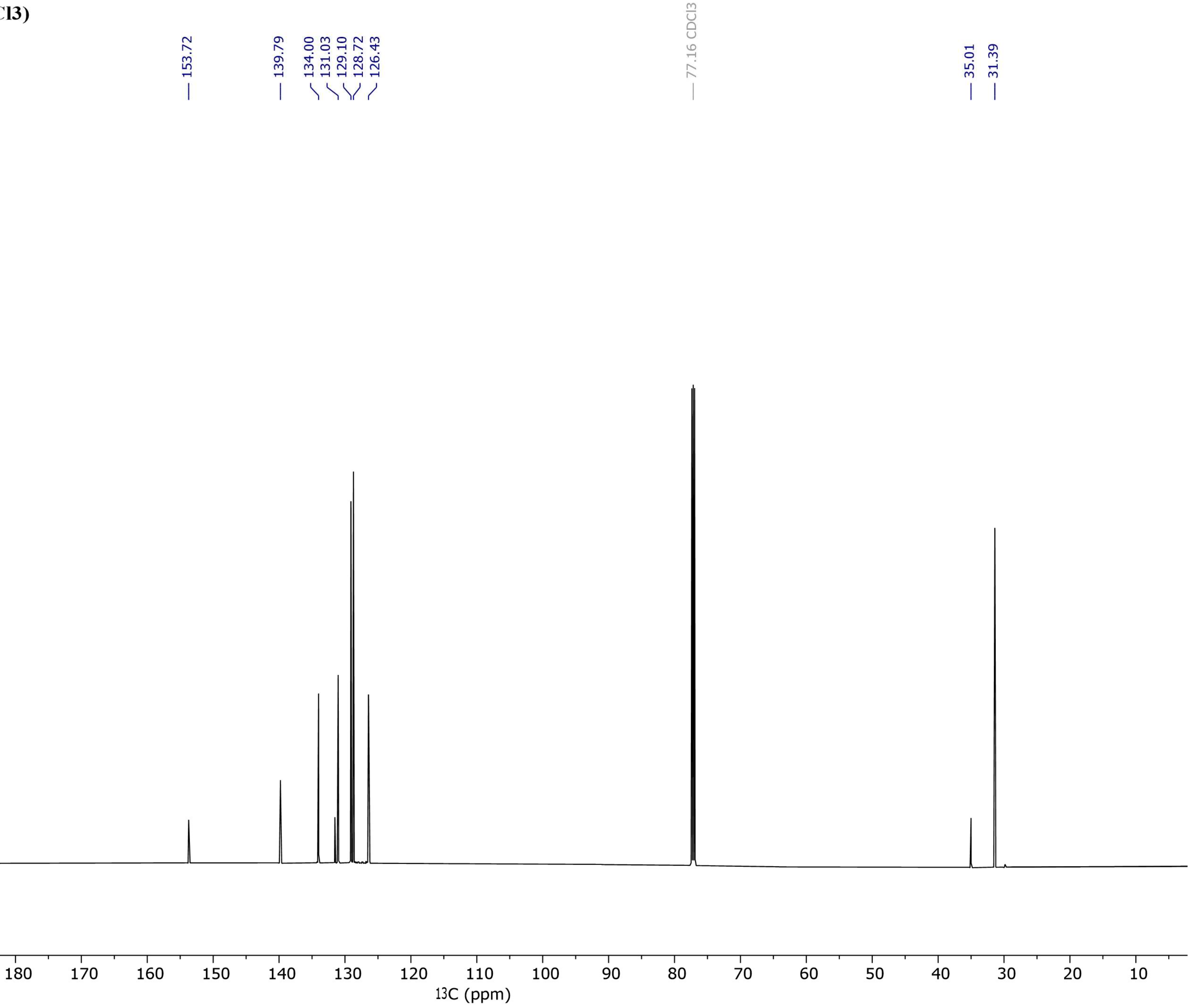

4-(tert-butyl)phenyl N-(phenylsulfonyl)benzenesulfonimidate (3a)

1H NMR (600 MHz, CDCl3)

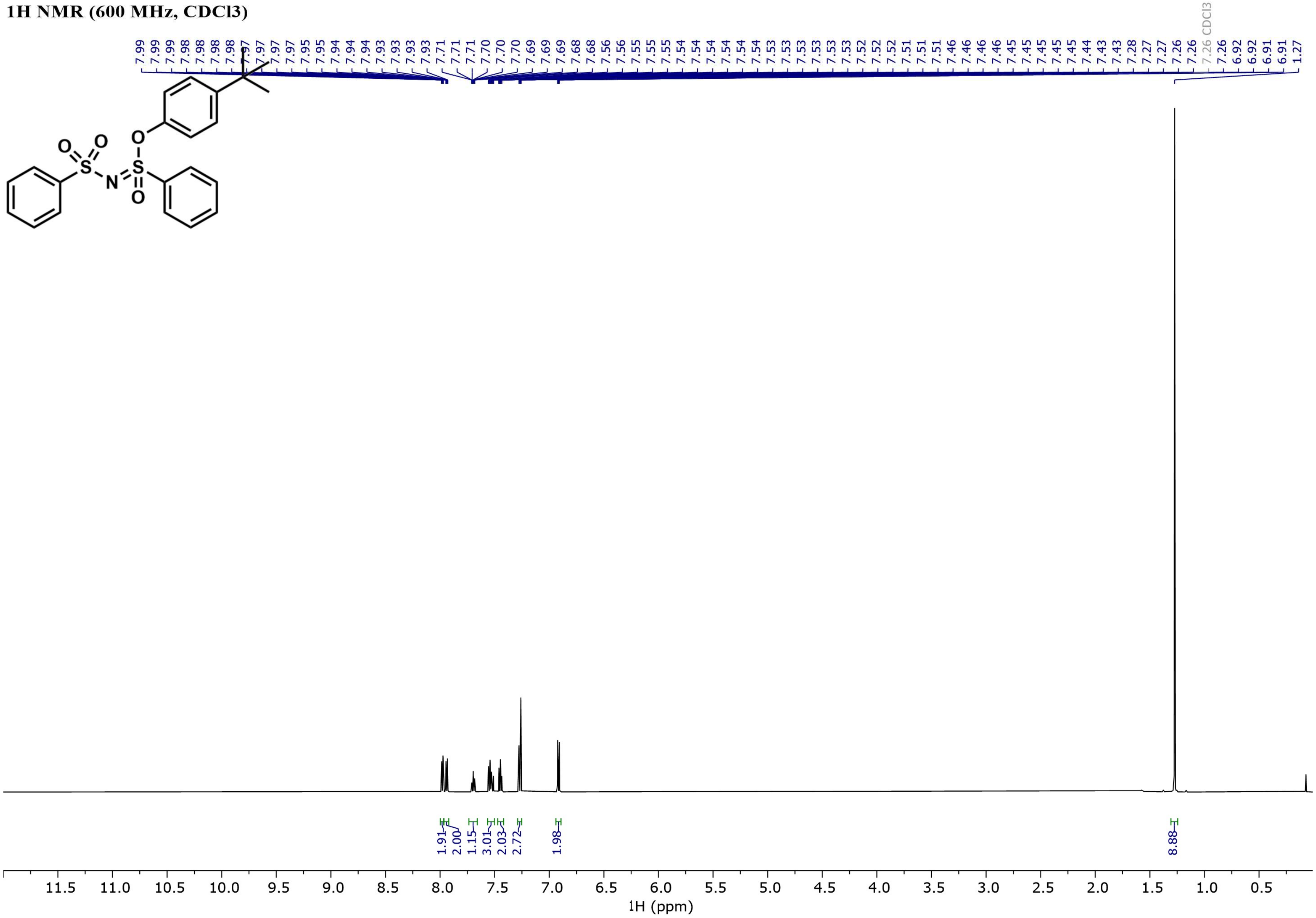

13C NMR (150 MHz, CDCl3)

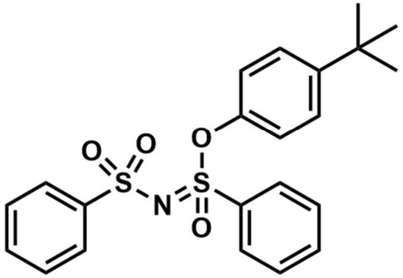

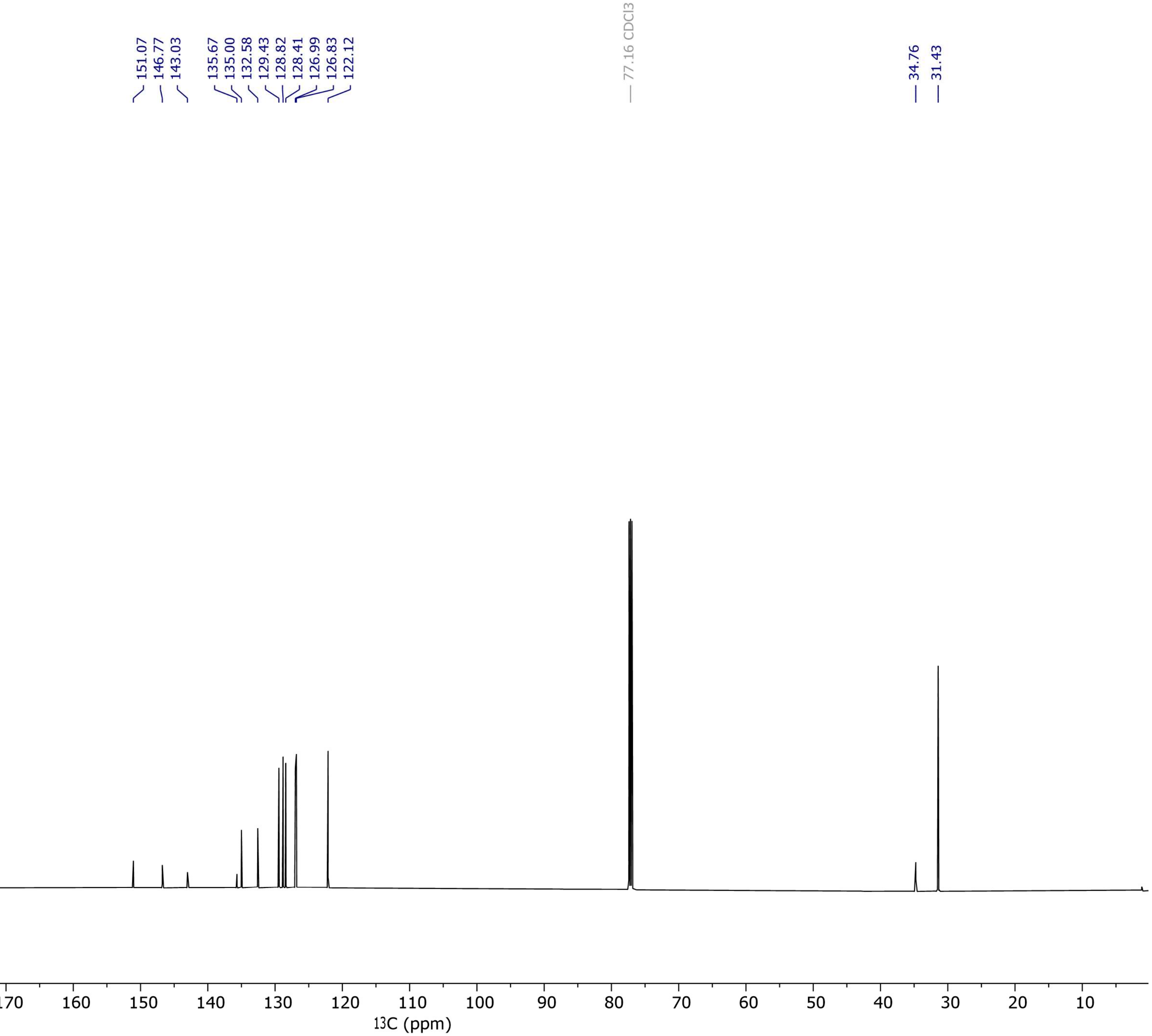

N-phenyl-N-(phenylsulfonyl)benzenesulfonamide (2b)

1H NMR (600 MHz, CDCl3)

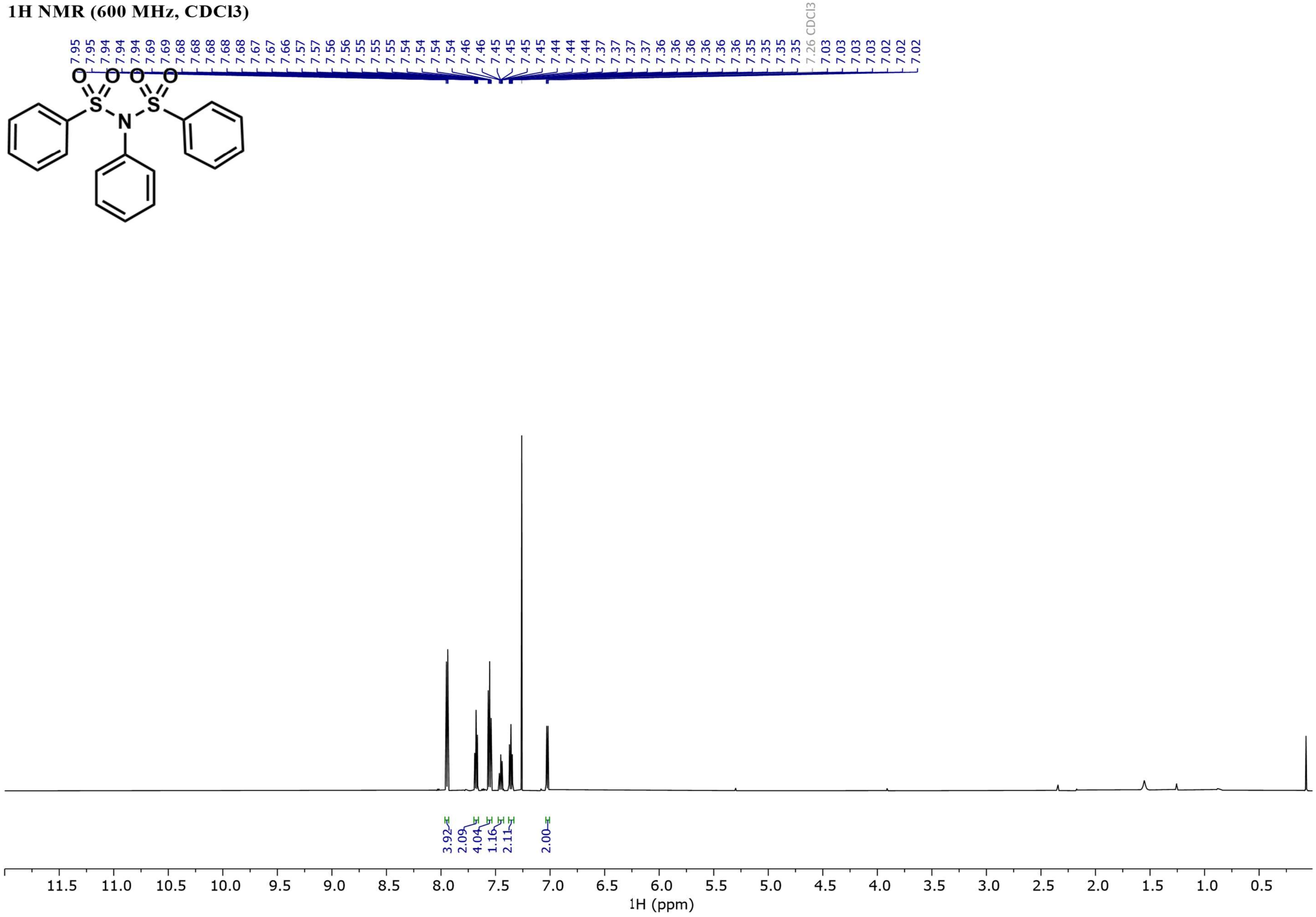

13C NMR (150 MHz, CDCl3)

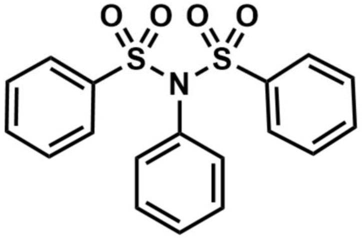

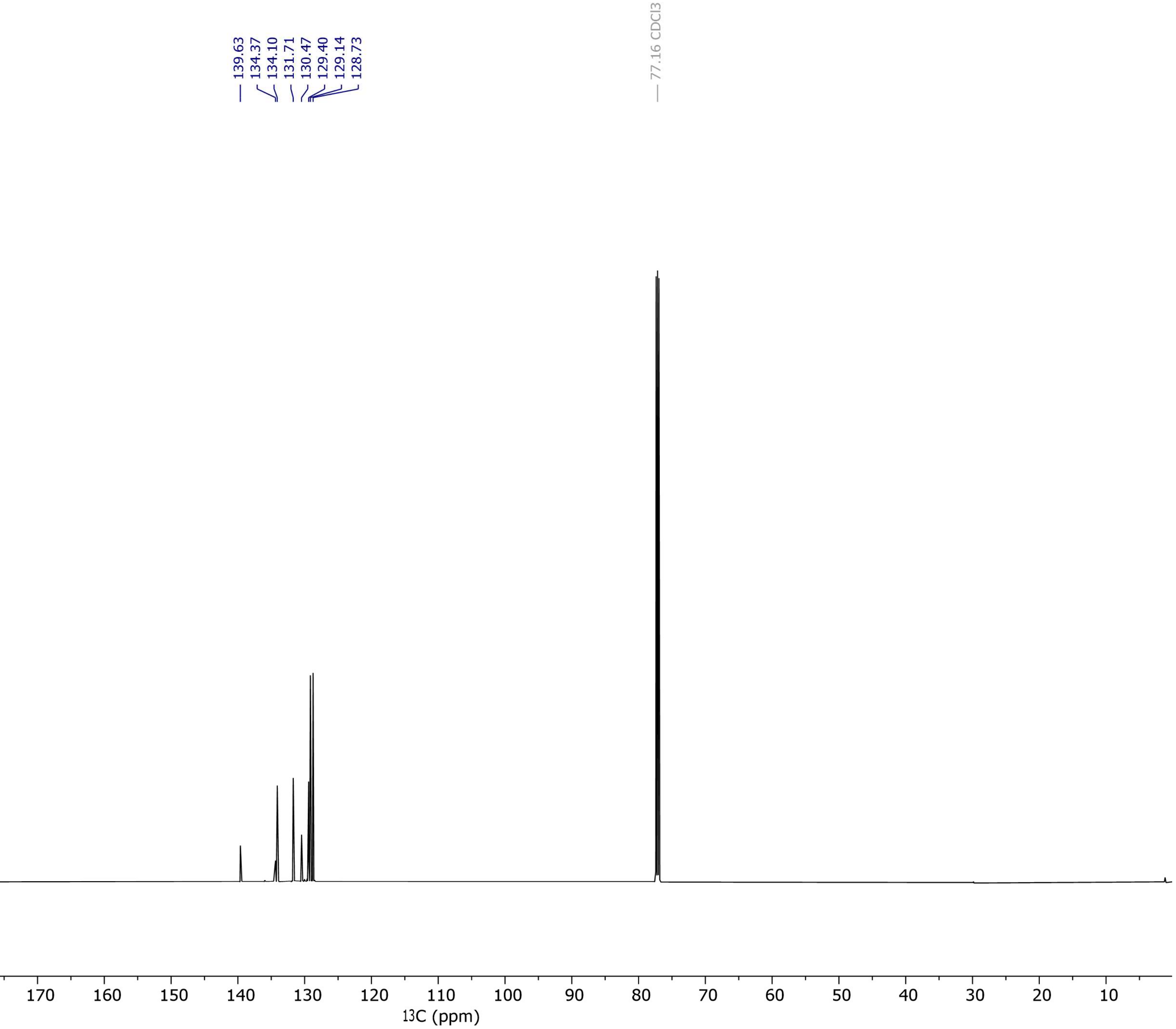

phenyl N-(phenylsulfonyl)benzenesulfonimidate (3b)

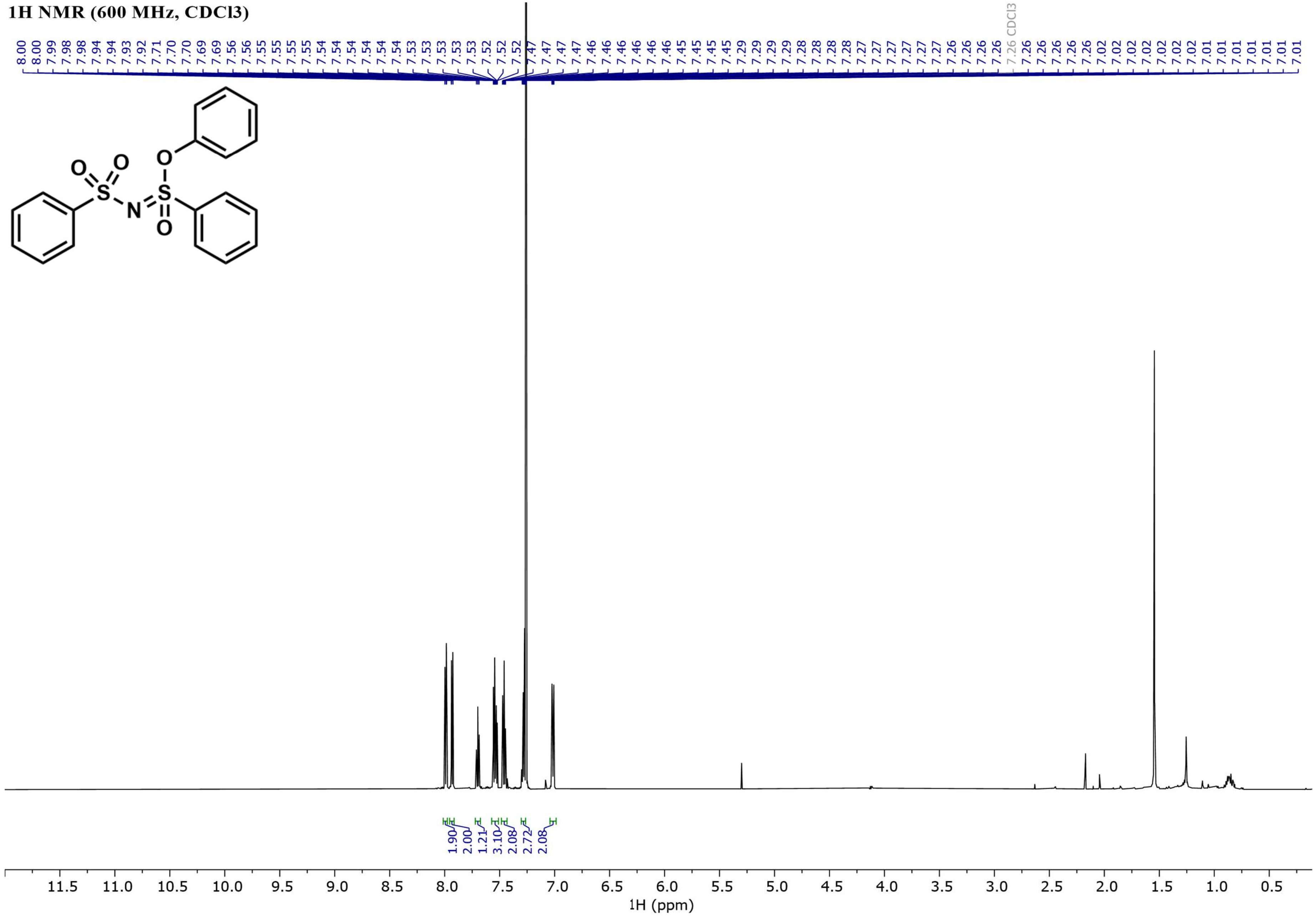

13C NMR (150 MHz, CDCl3)

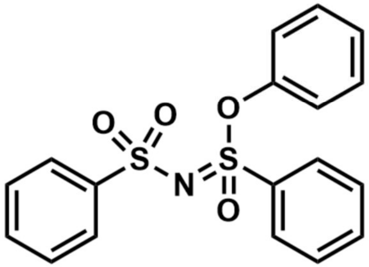

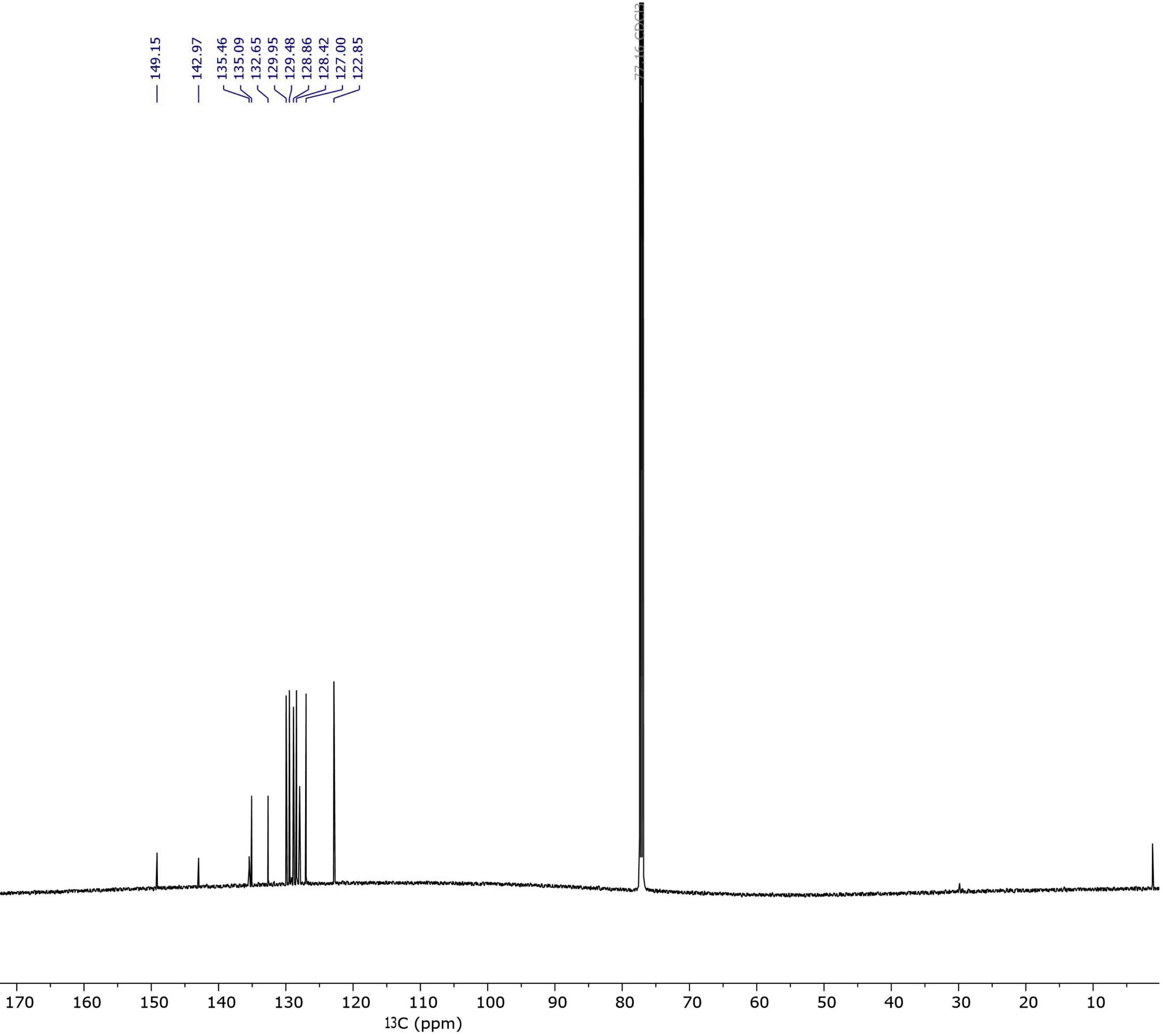

N-(3,5-dimethylphenyl)-N-(phenylsulfonyl)benzenesulfonamide (2c)

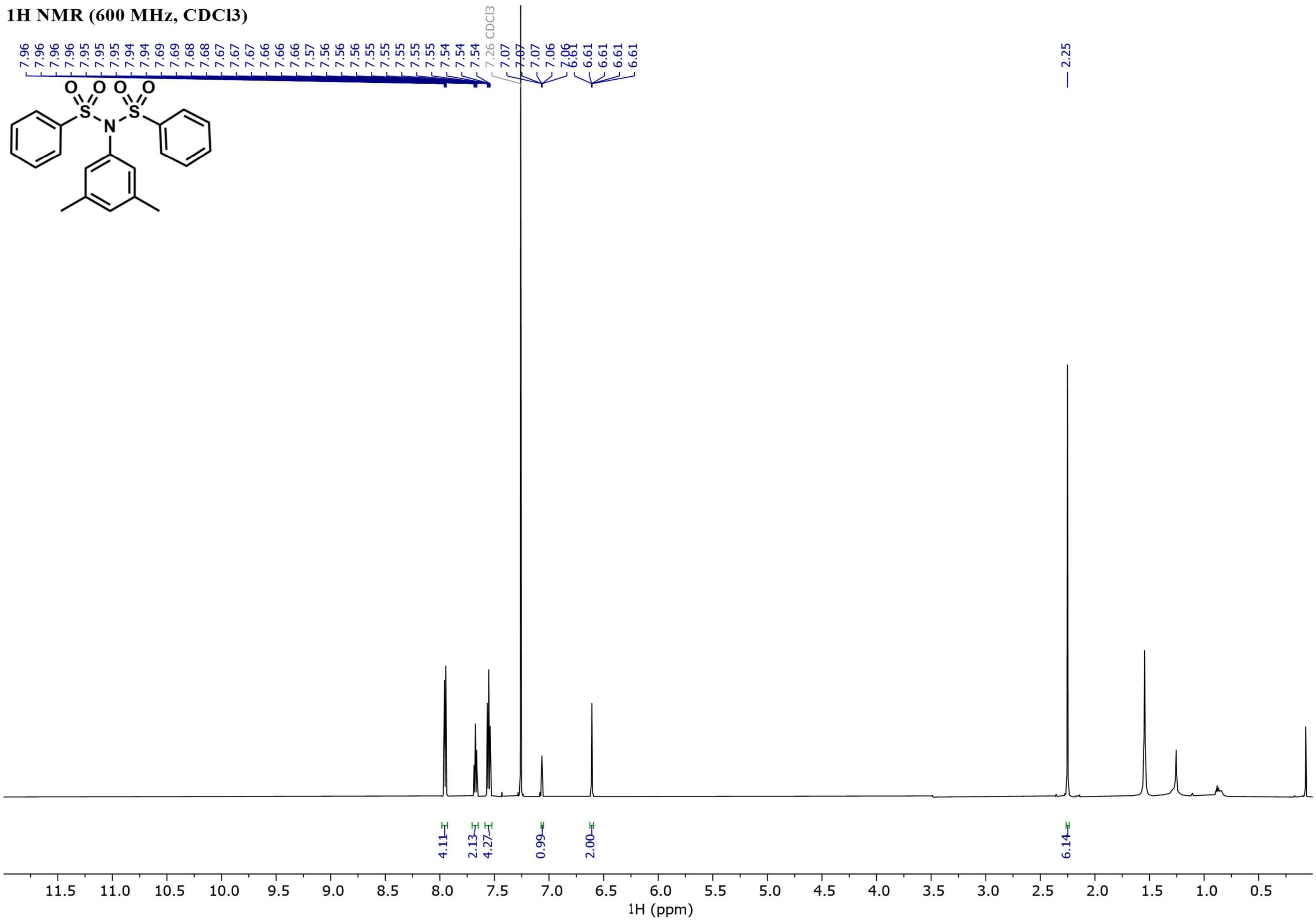

13C NMR (150 MHz, CDCl3)

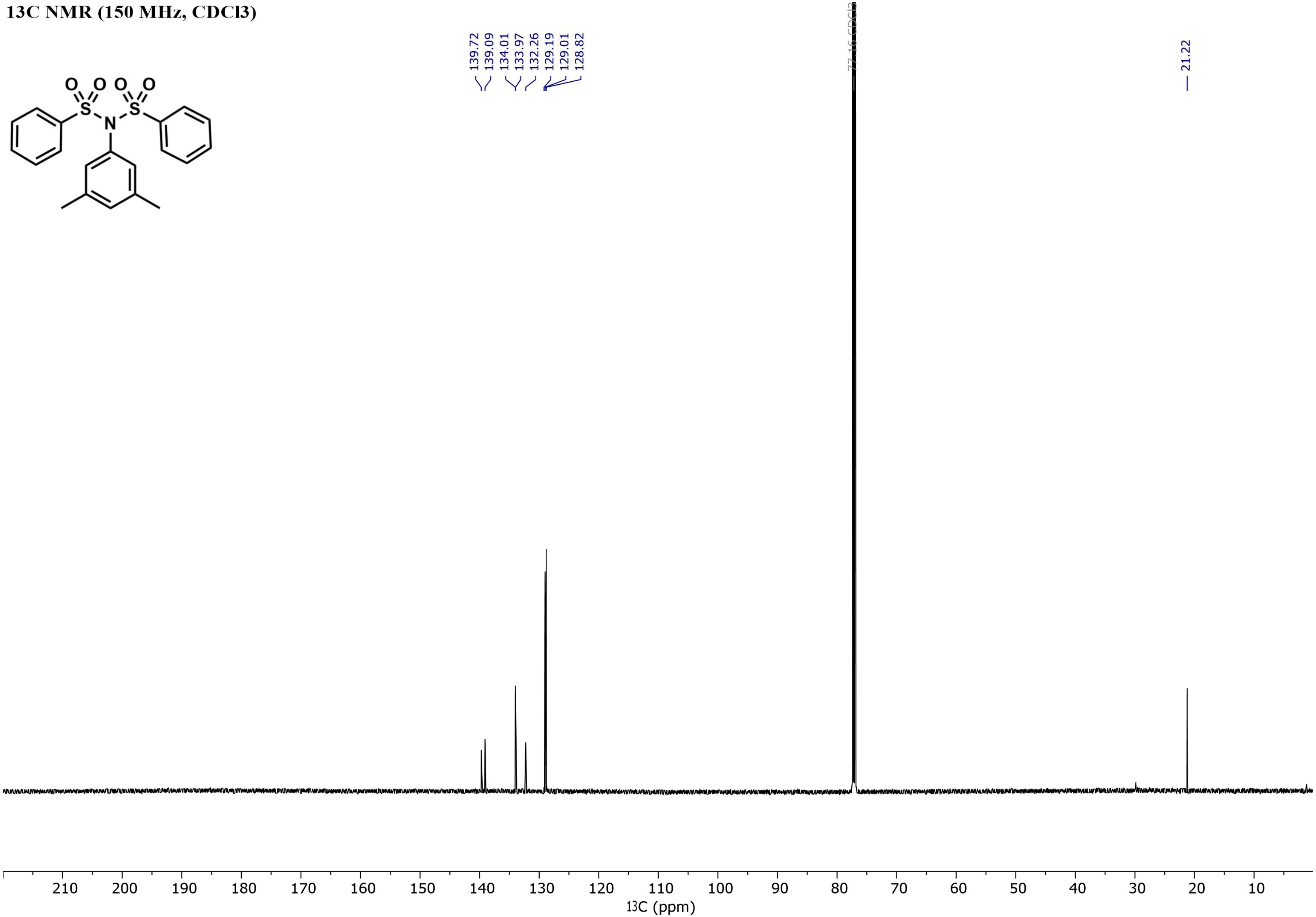

3,5-dimethylphenyl N-(phenylsulfonyl)benzenesulfonimidate (3c)

1H NMR (600 MHz, CDCl3)

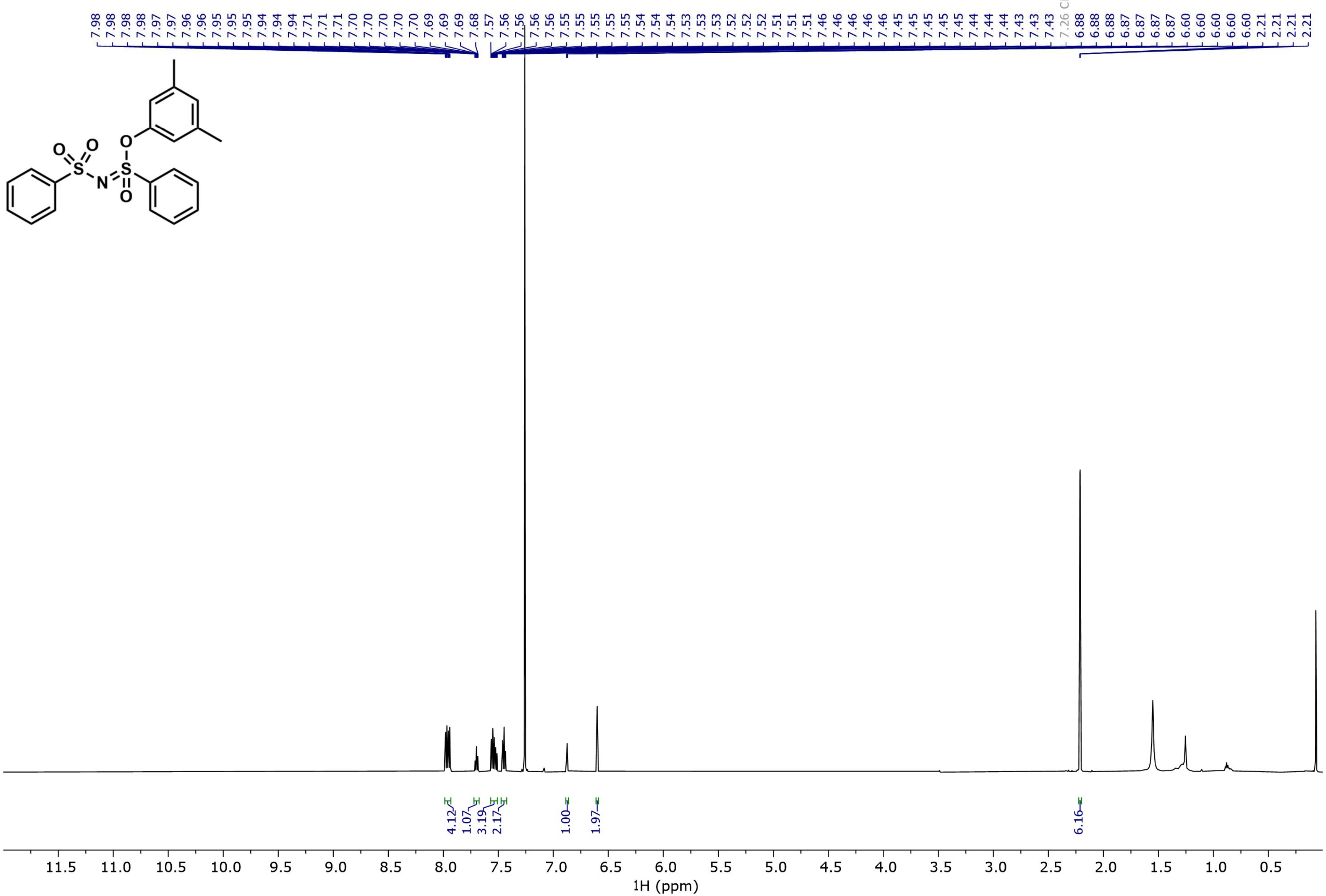

13C NMR (150 MHz, CDCl3)

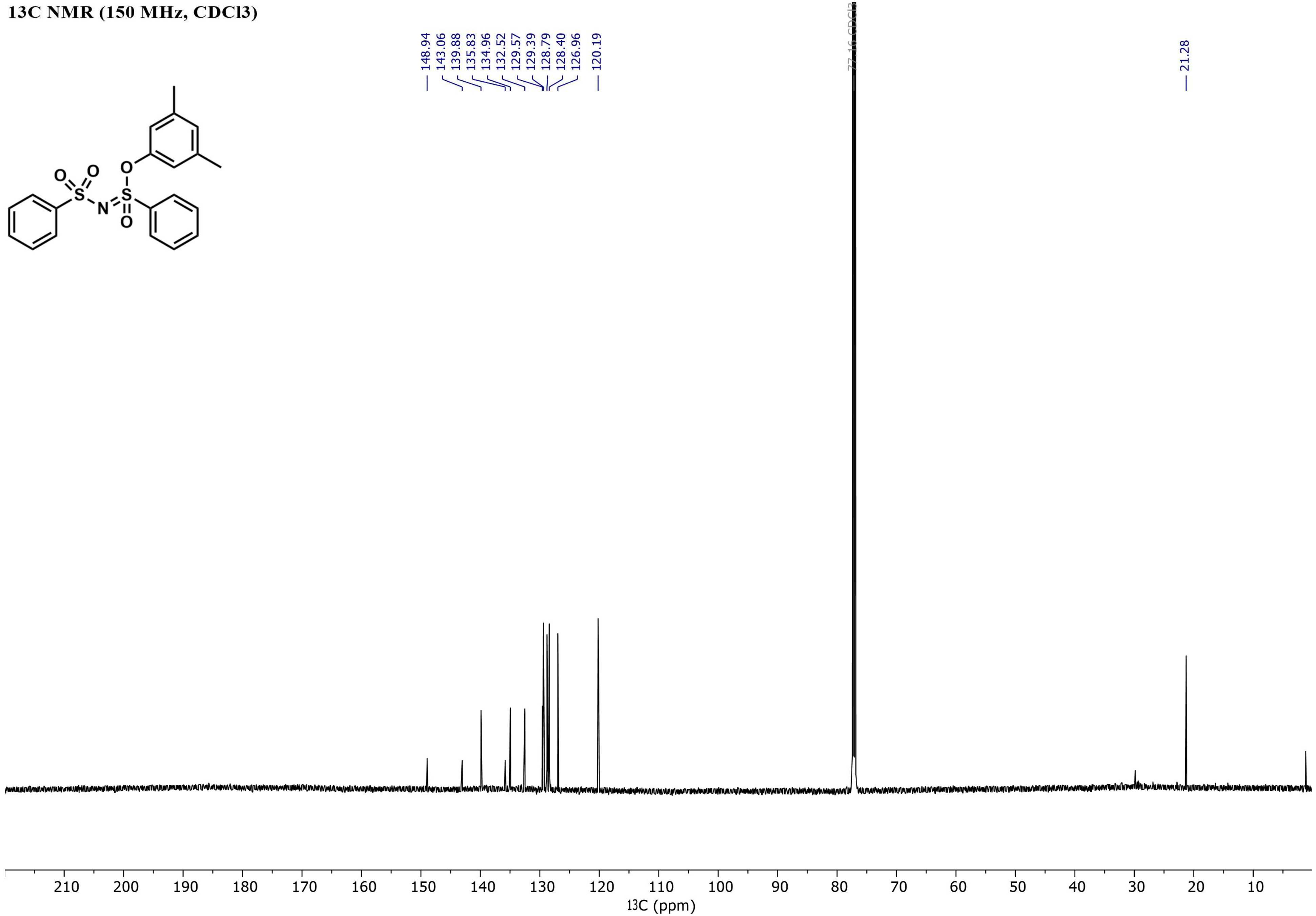

N-(3-methoxyphenyl)-N-(phenylsulfonyl)benzenesulfonamide (2d)

1H NMR (600 MHz, CDCl3)

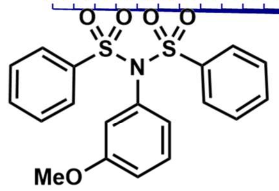

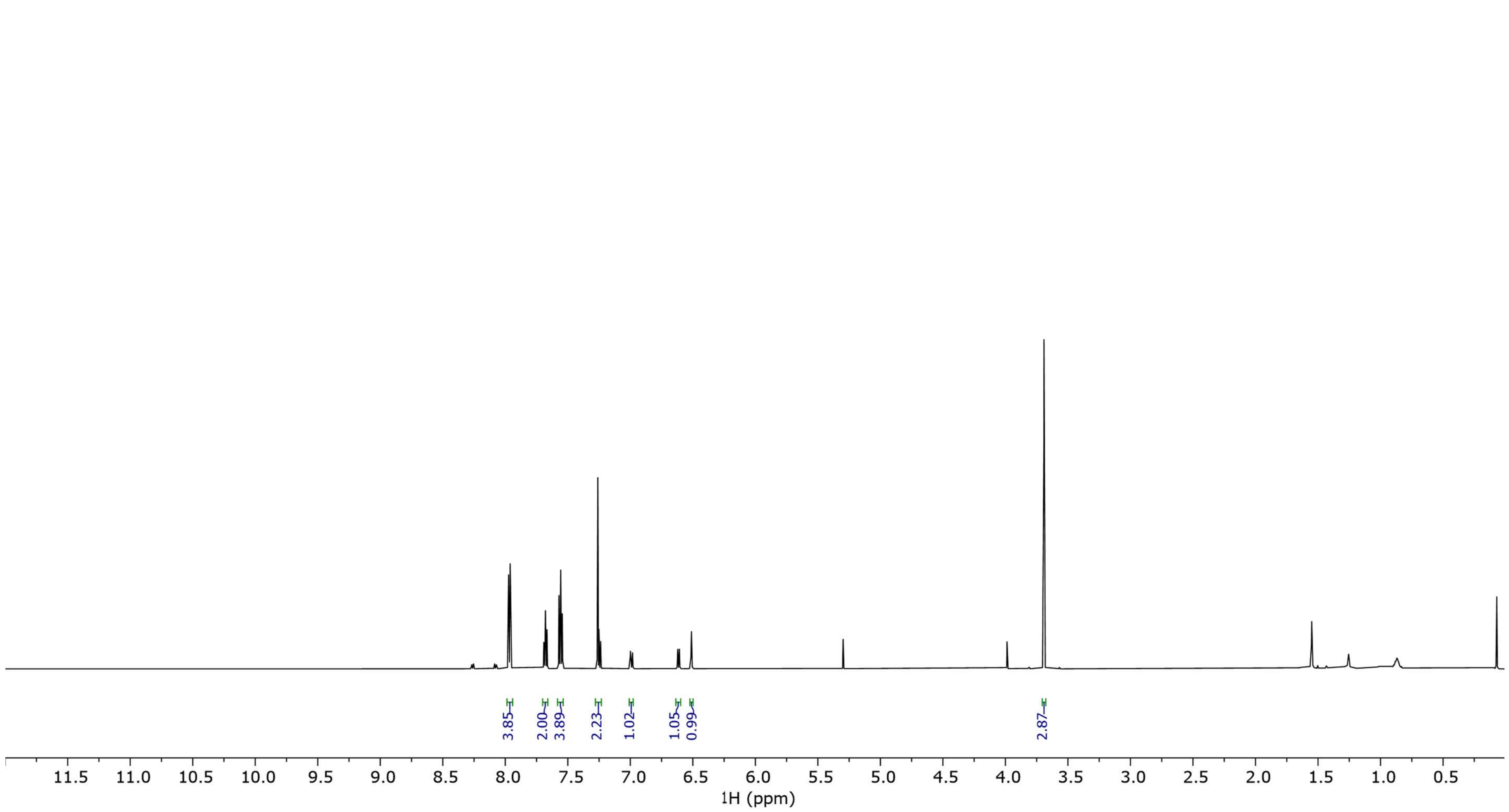

13C NMR (150 MHz, CDCl3)

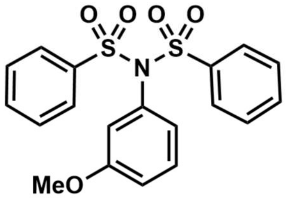

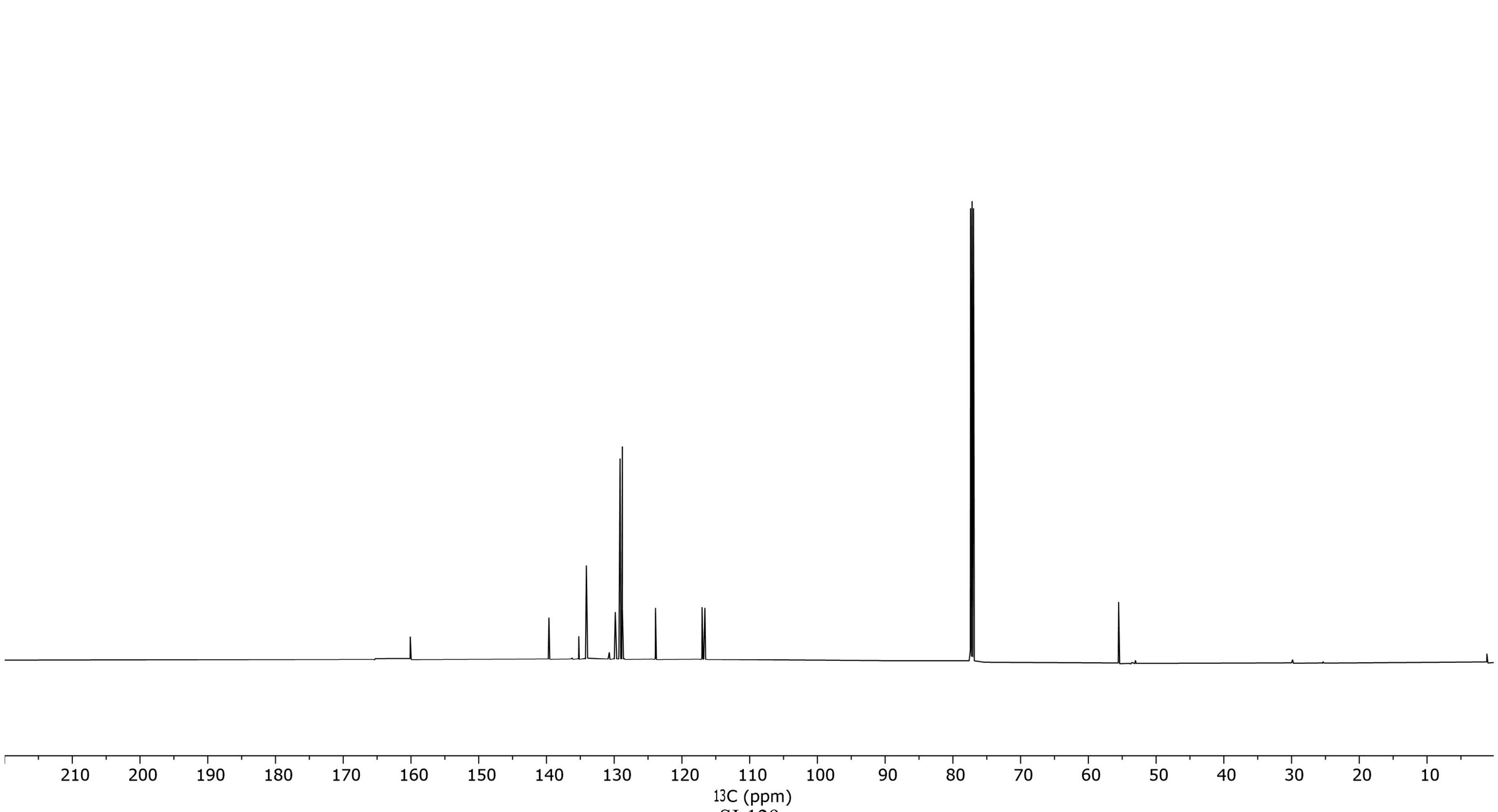

3-methoxyphenyl N-(phenylsulfonyl)benzenesulfonimidate (3d)

1H NMR (600 MHz, CDCl3)

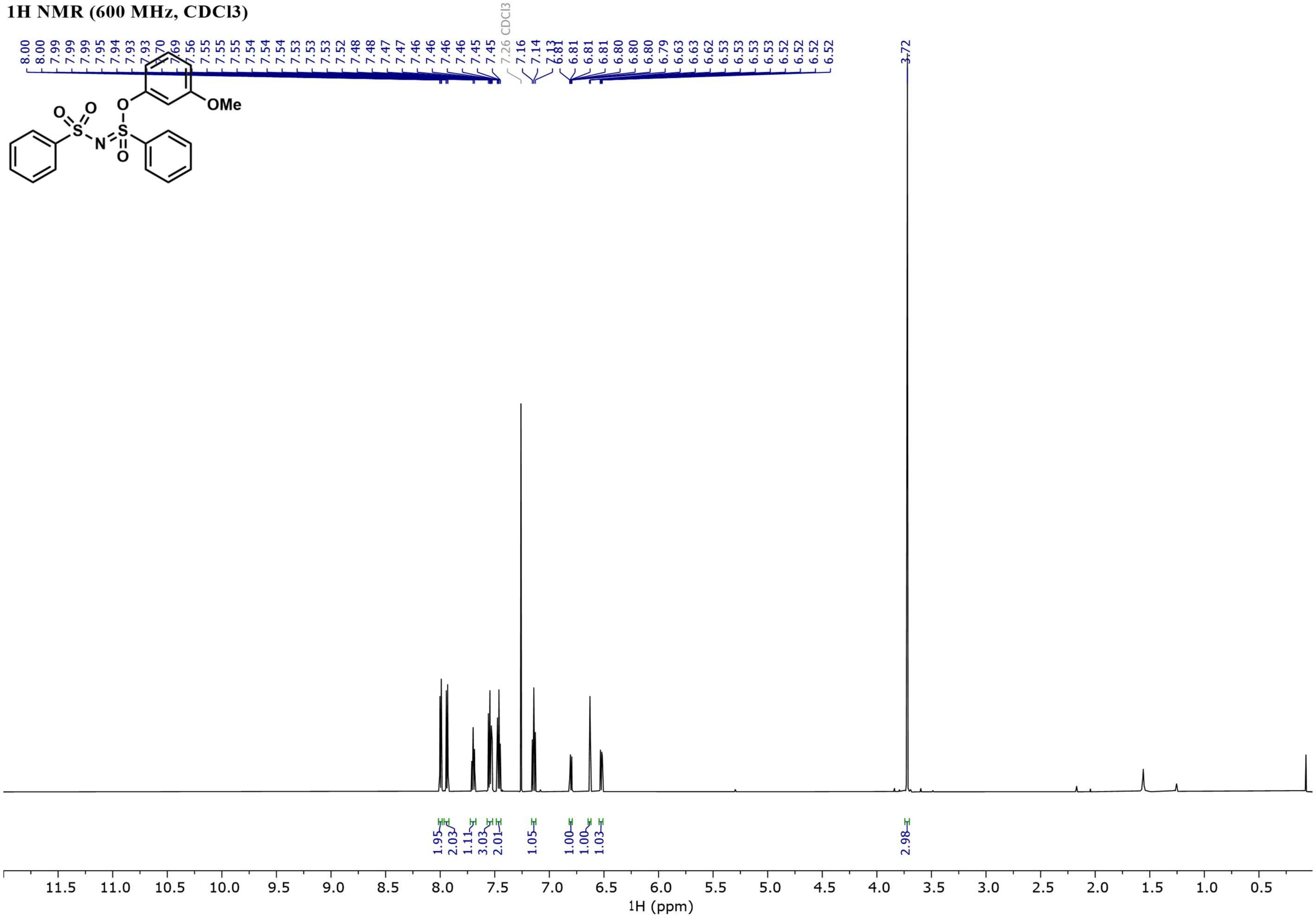

13C NMR (150 MHz, CDCl3)

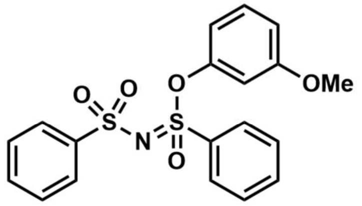

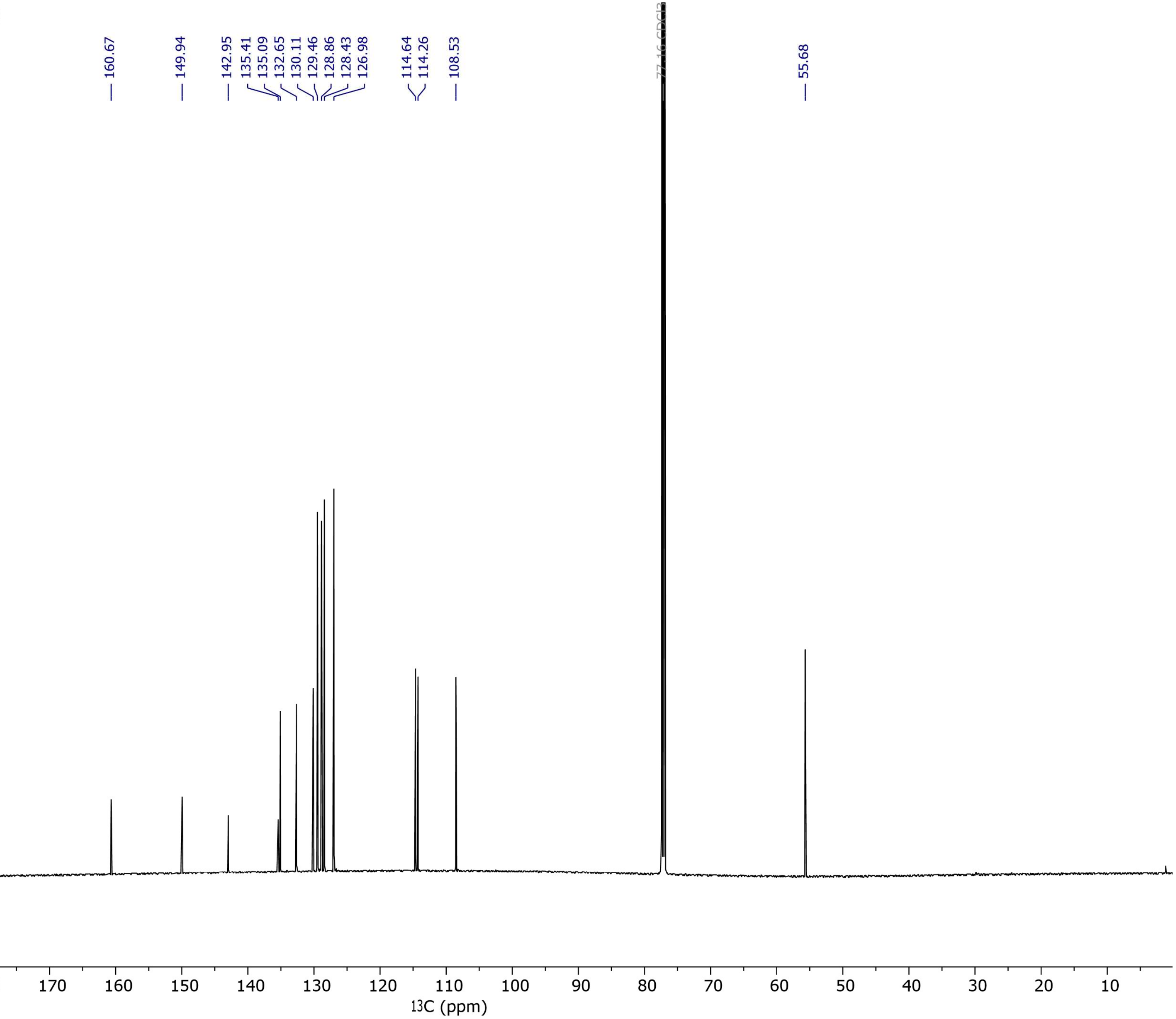

N-(4-methoxyphenyl)-N-(phenylsulfonyl)benzenesulfonamide (2e)

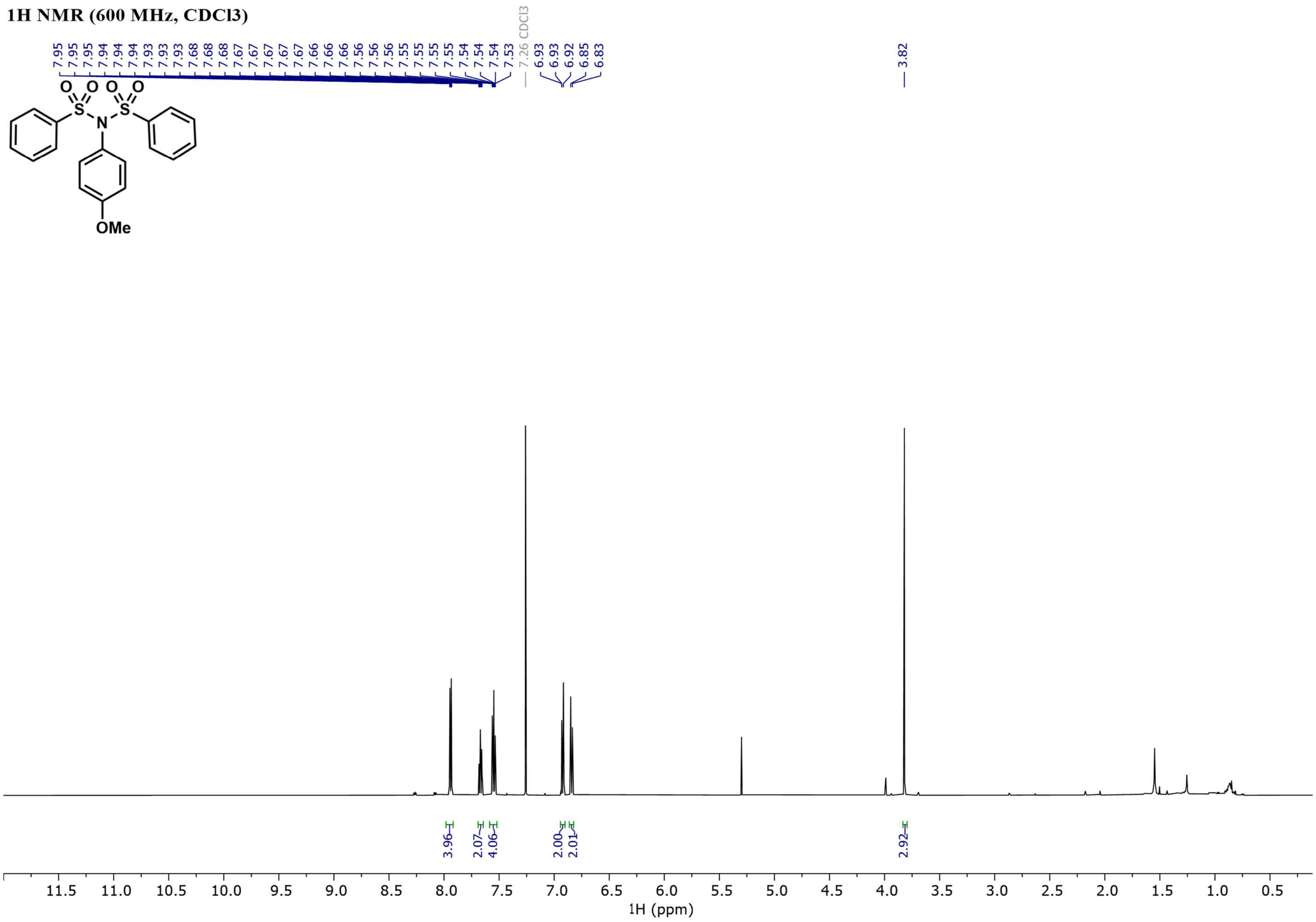

13C NMR (150 MHz, CDCl3)

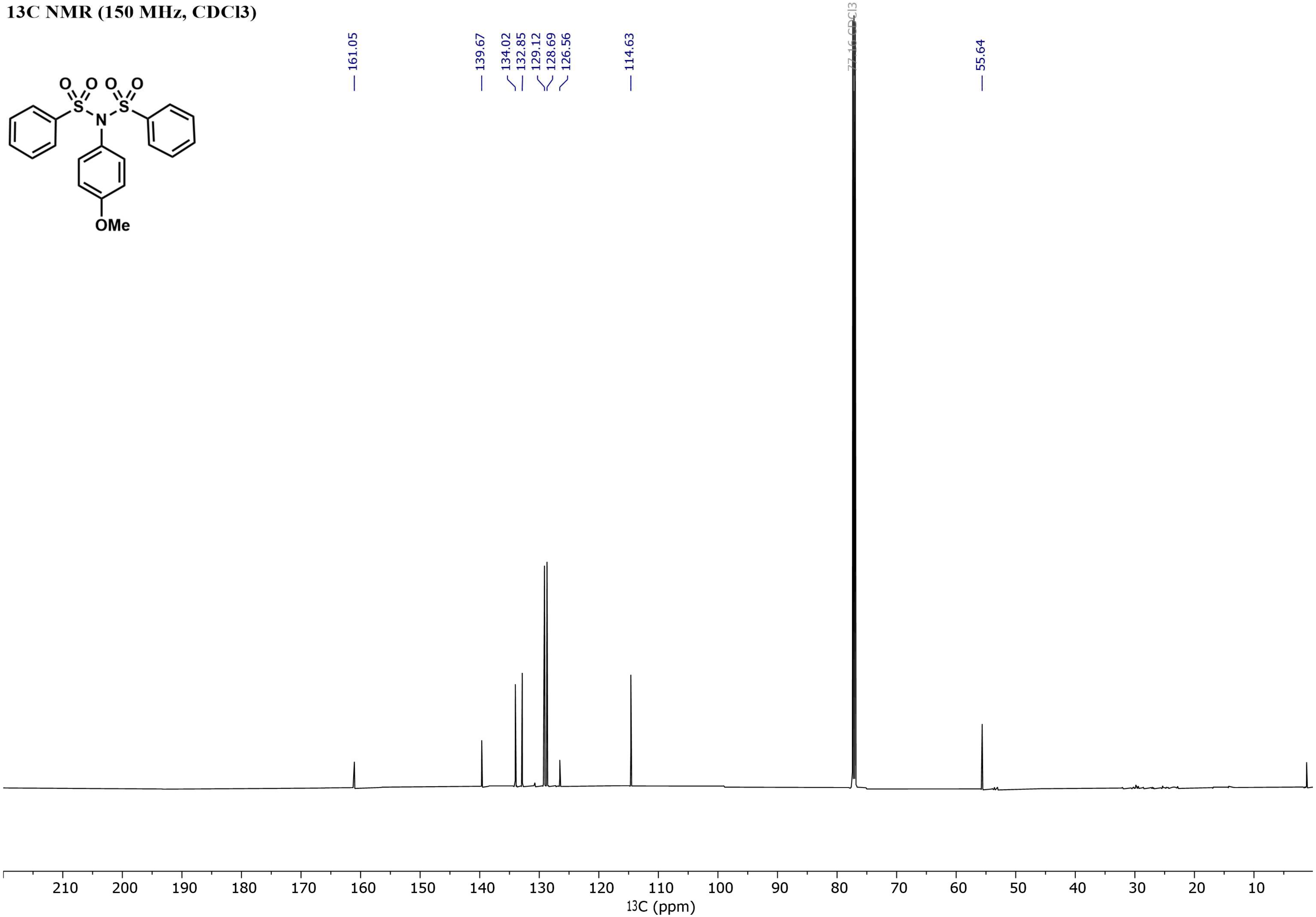

4-methoxyphenyl N-(phenylsulfonyl)benzenesulfonimidate (3e)

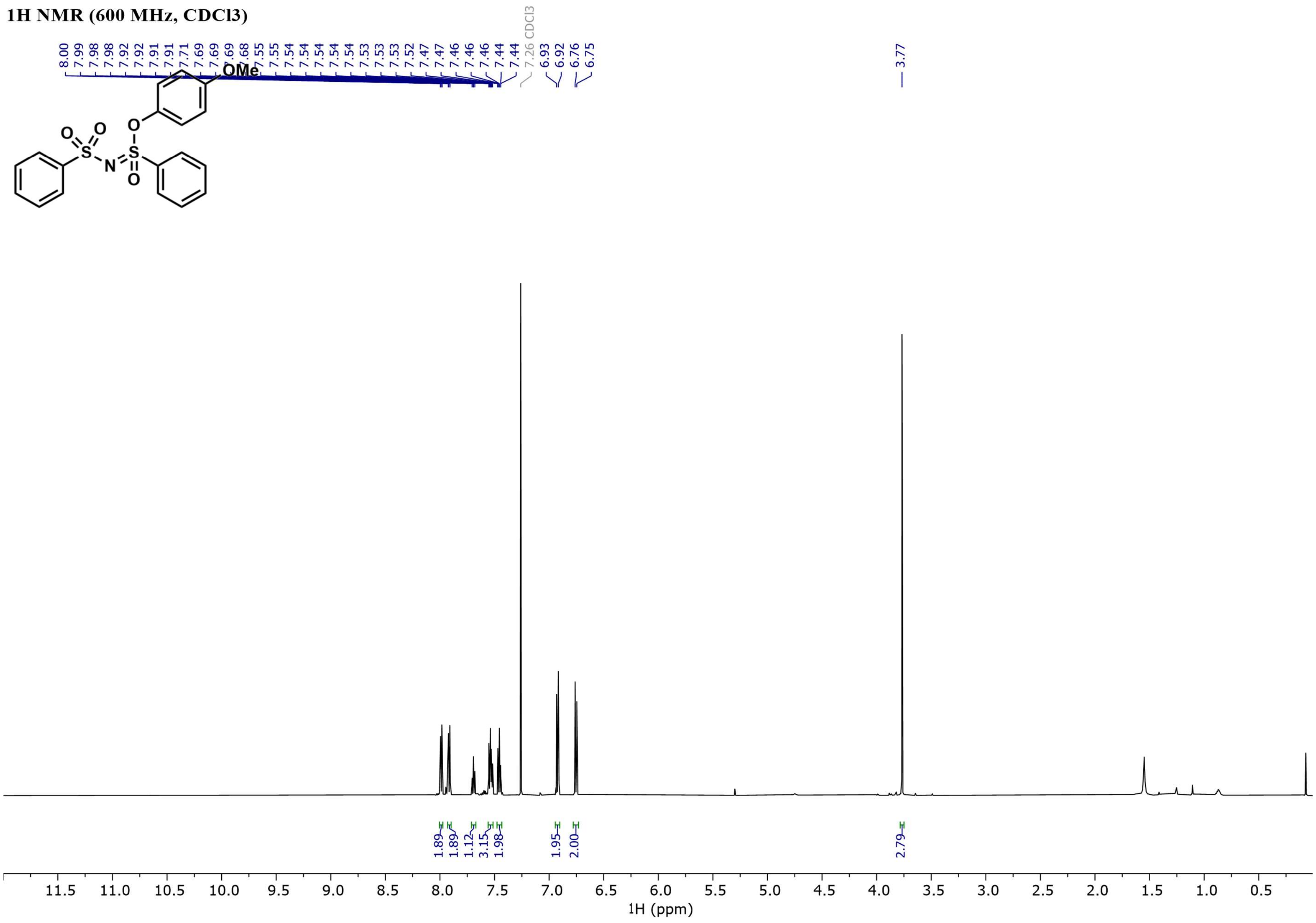

13C NMR (150 MHz, CDCl3)

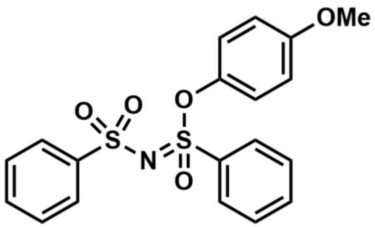

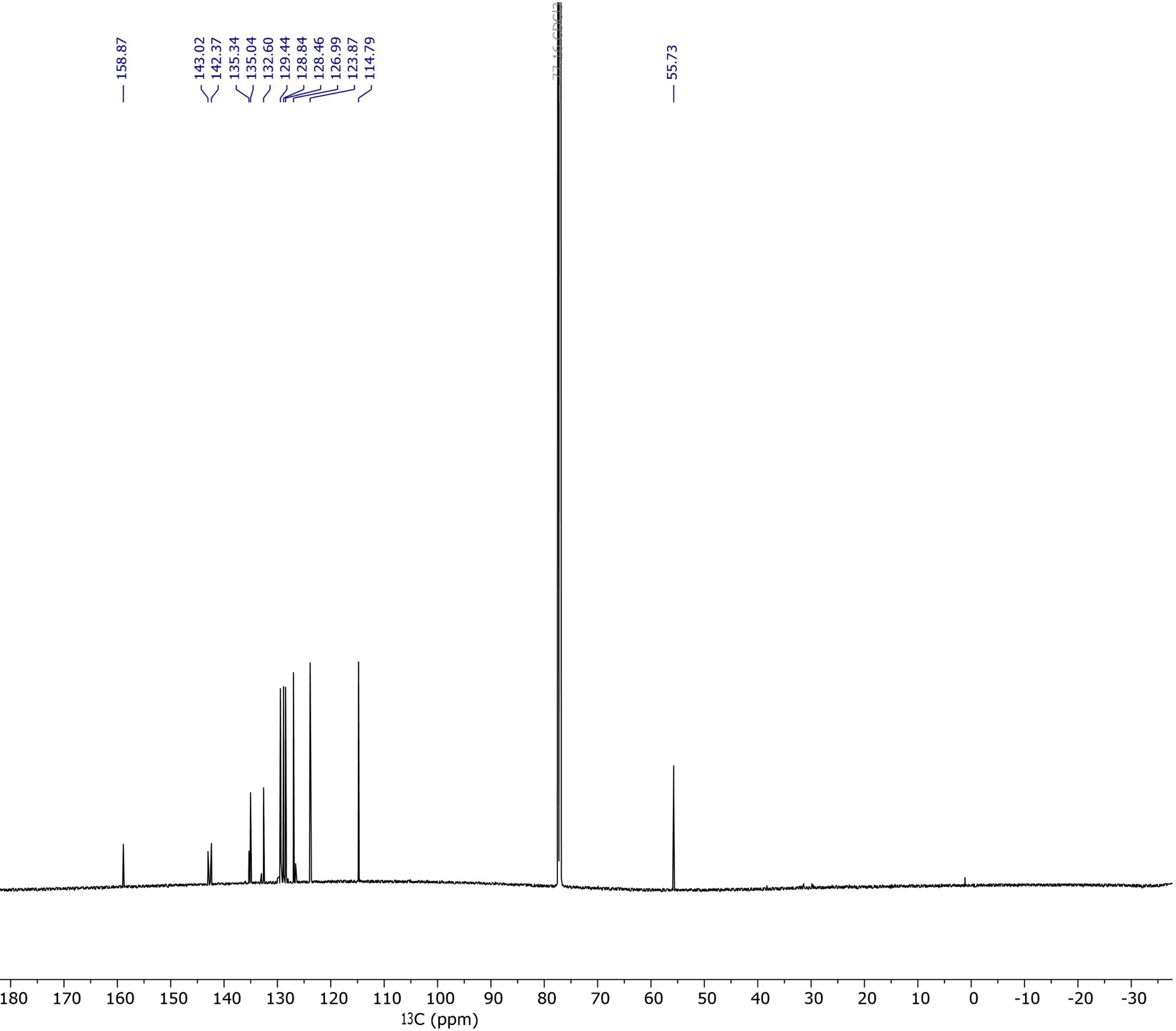

N-(4-fluorophenyl)-N-(phenylsulfonyl)benzenesulfonamide (2f)

1H NMR (600 MHz, CDCl3)

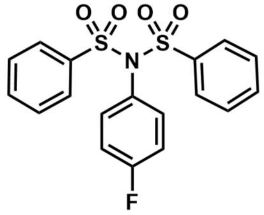

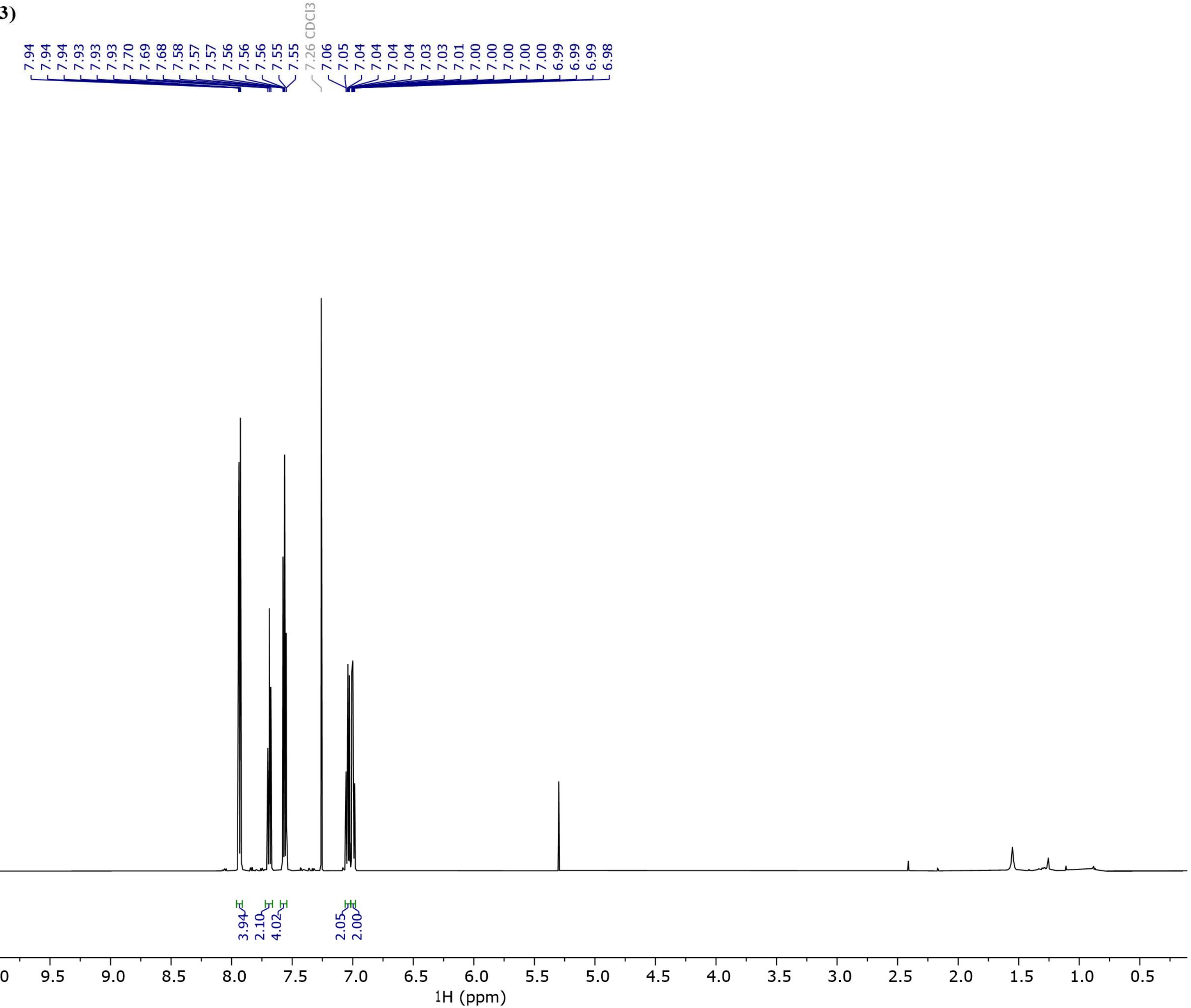

13C NMR (150 MHz, CDCl3)

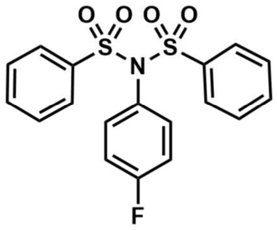

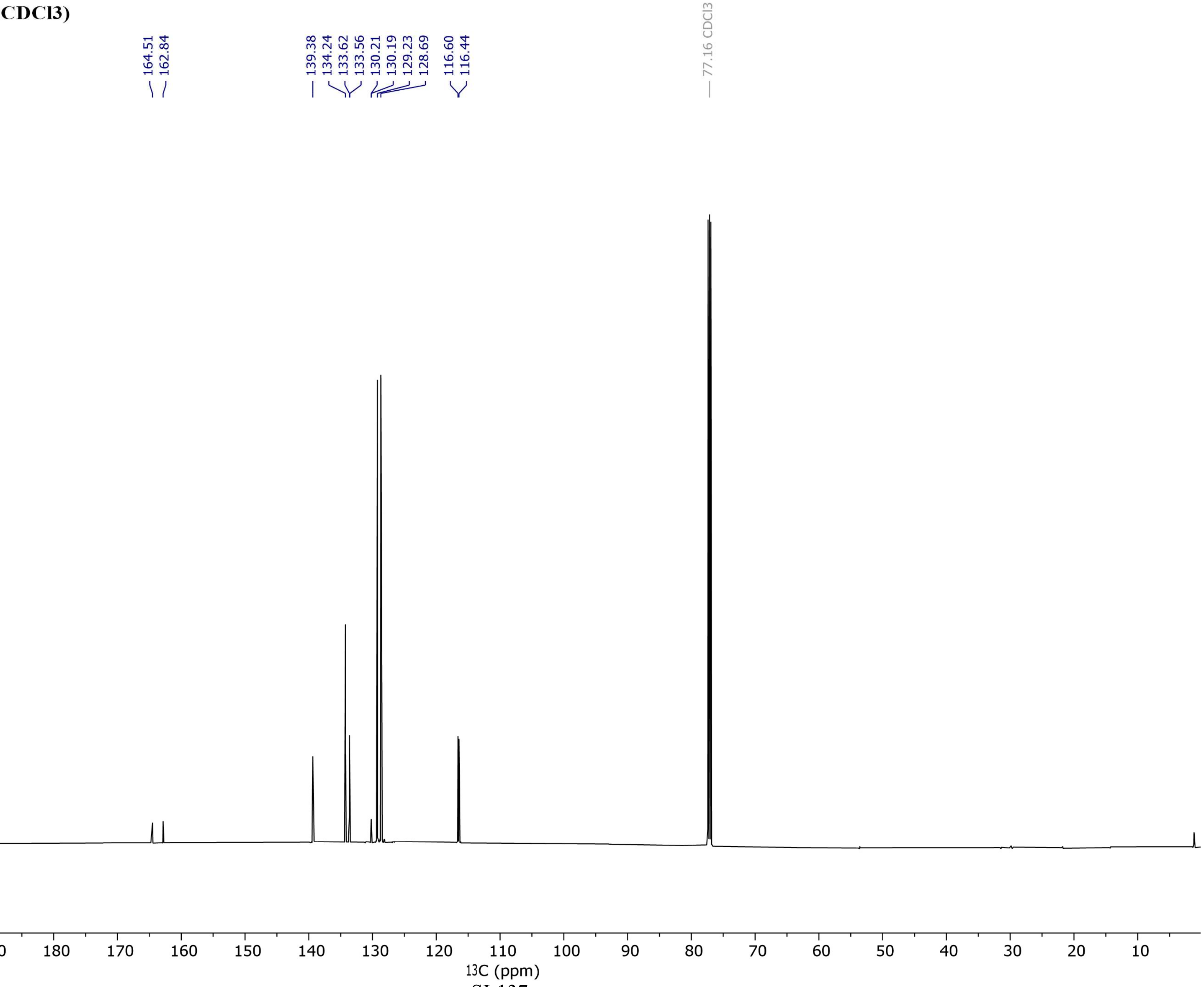

19F NMR (282 MHz, CDCl3)

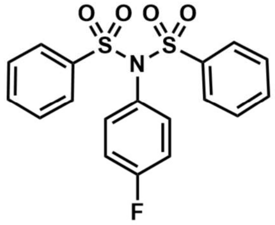

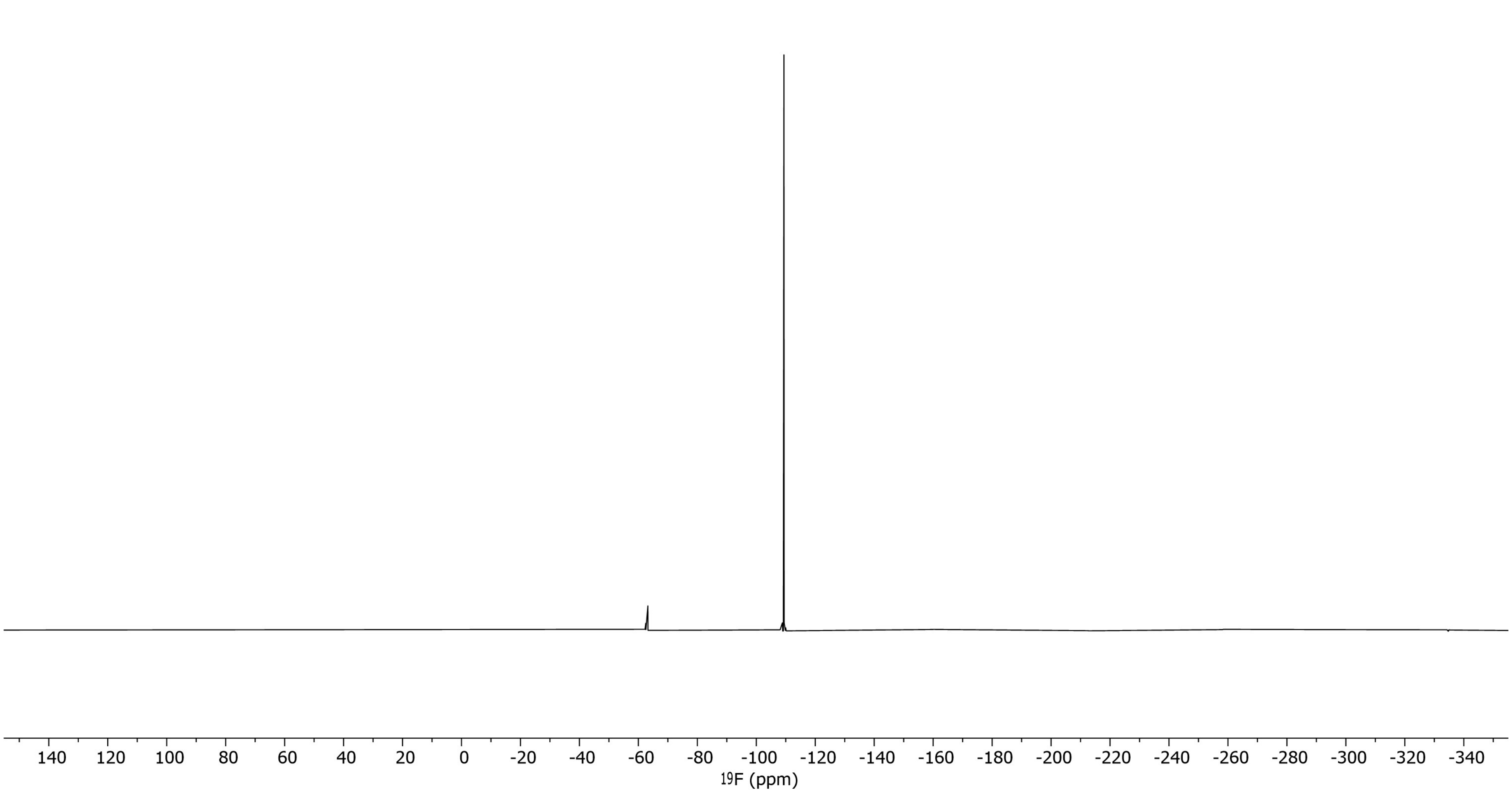

4-fluorophenyl N-(phenylsulfonyl)benzenesulfonimidate (3f)

1H NMR (600 MHz, CDCl3)

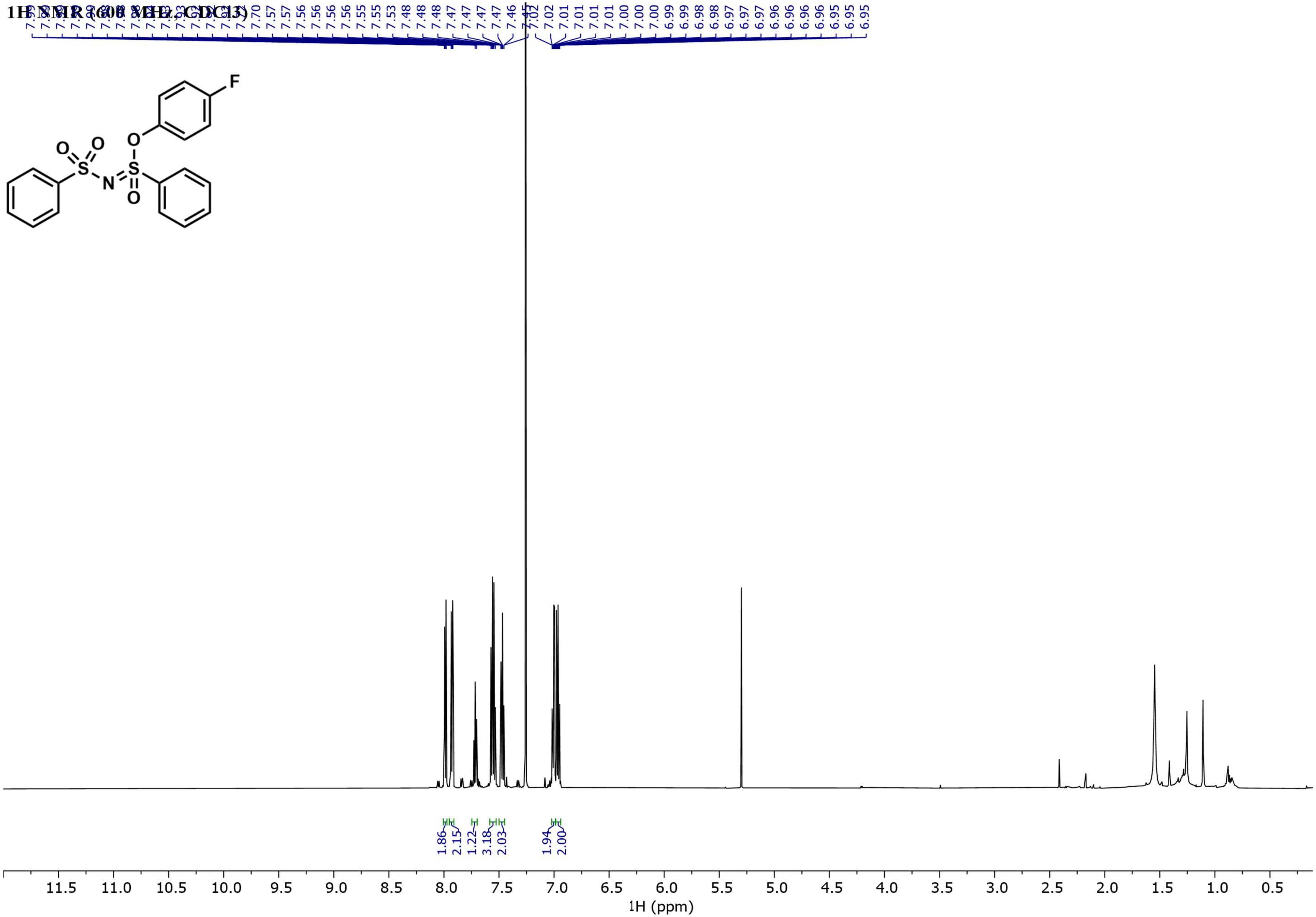

13C NMR (150 MHz, CDCl3)

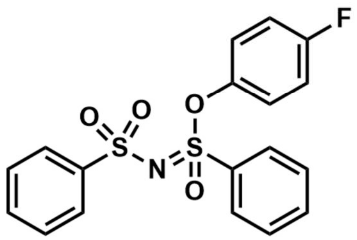

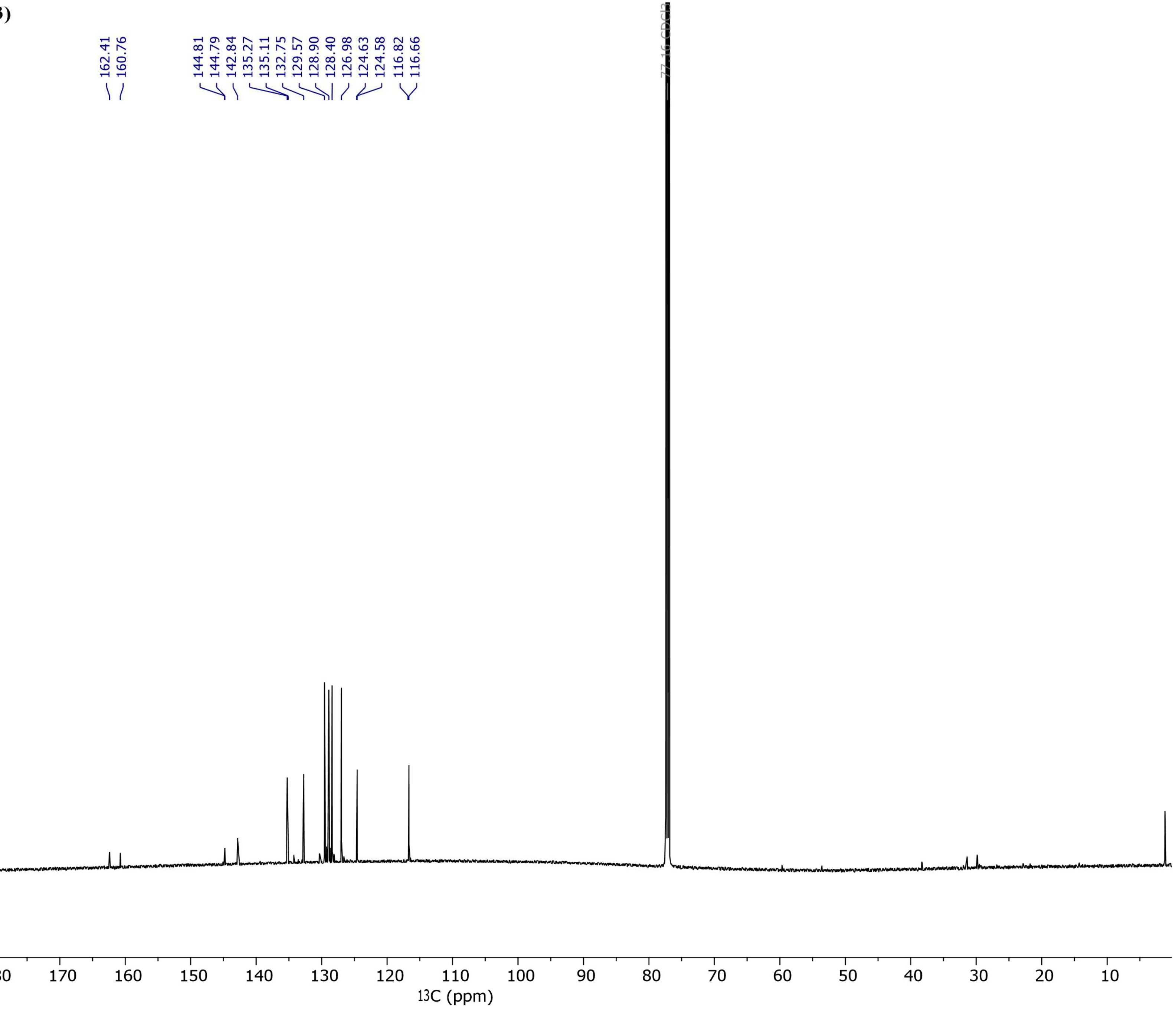

19F NMR (282 MHz, CDCl3)

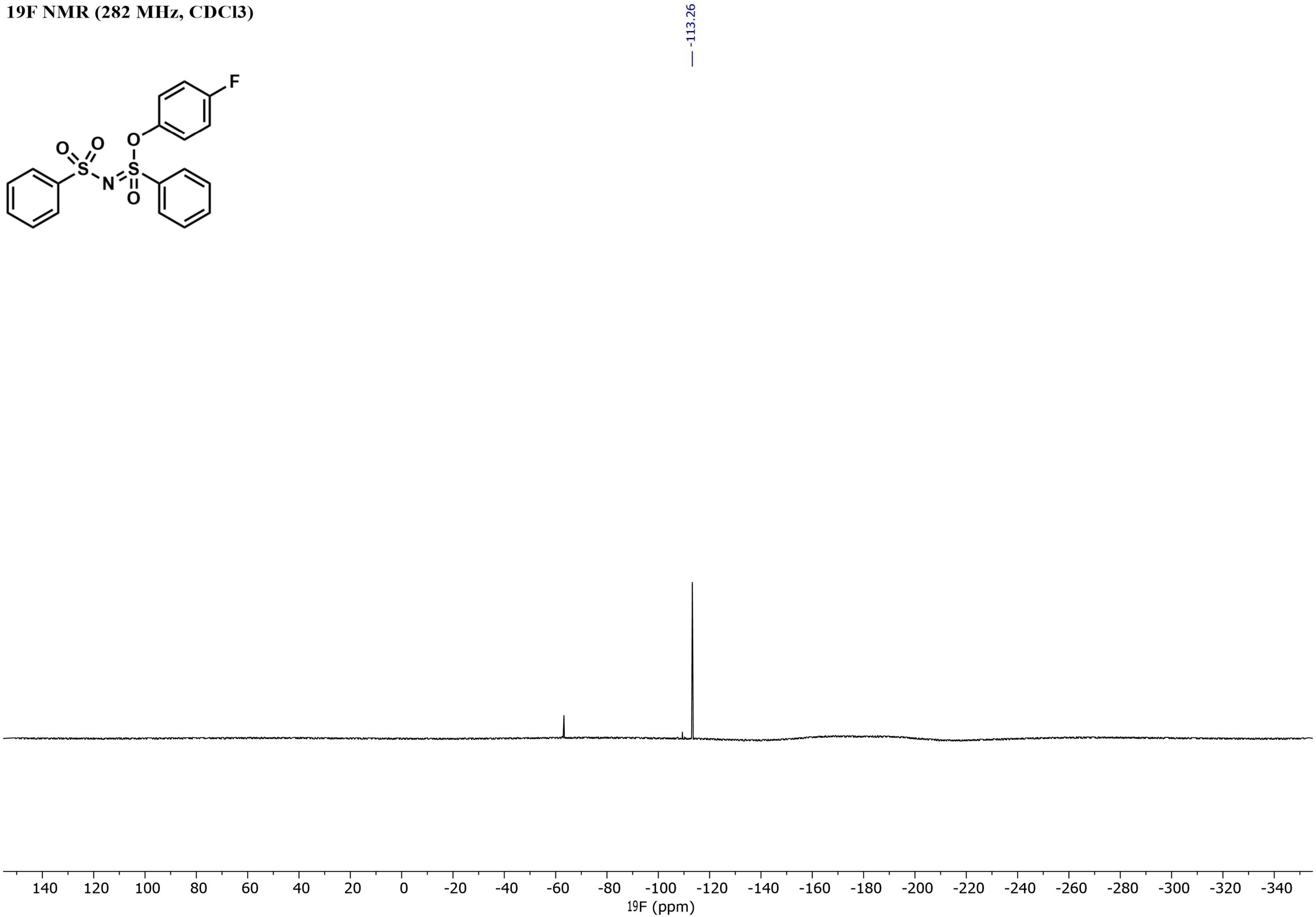

N-(phenylsulfonyl)-N-(4-(trifluoromethoxy)phenyl)benzenesulfonamide (2g)

1H NMR (600 MHz, CDCl3)

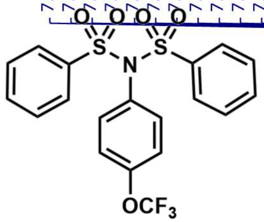

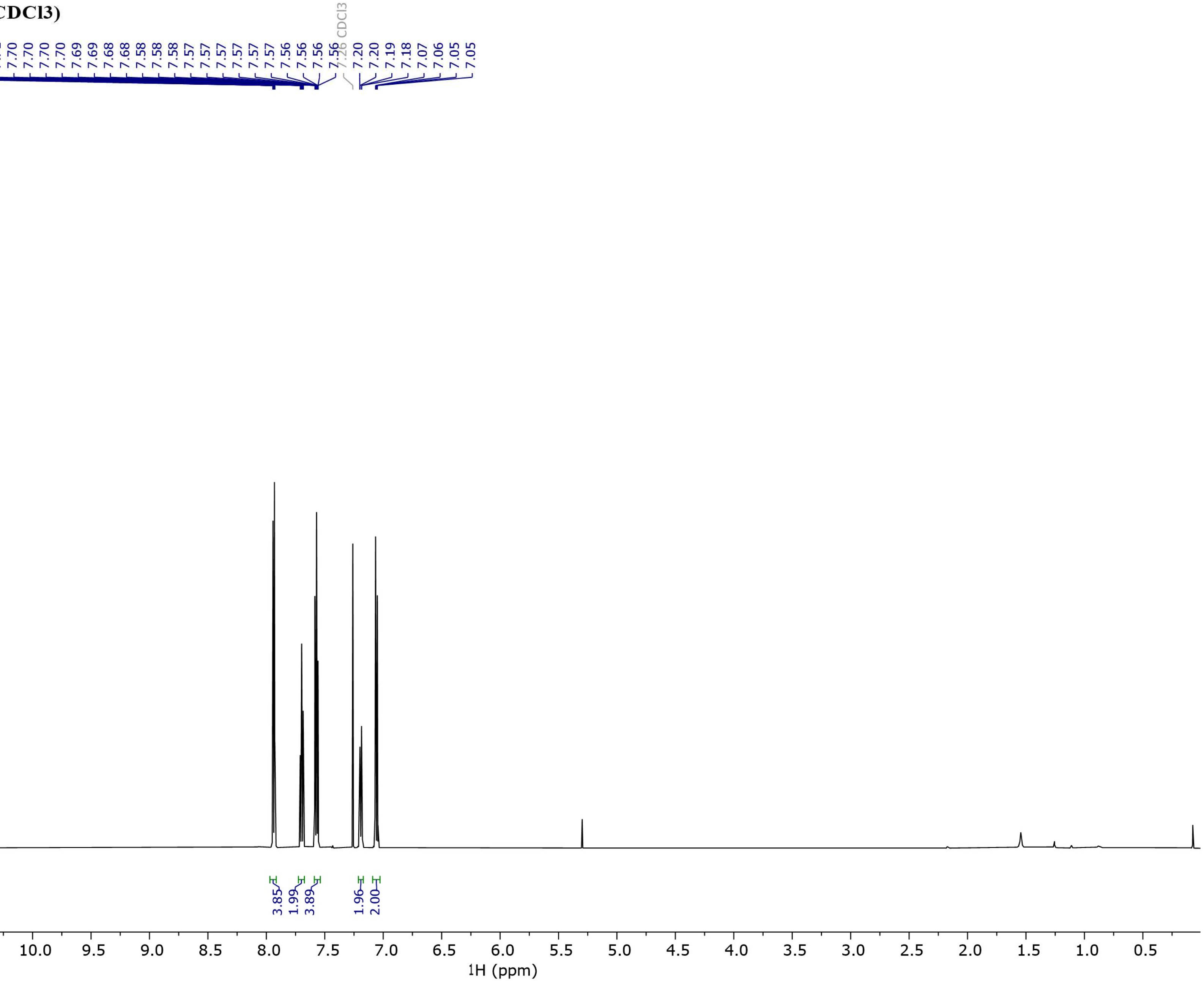

13C NMR (150 MHz, CDCl3)

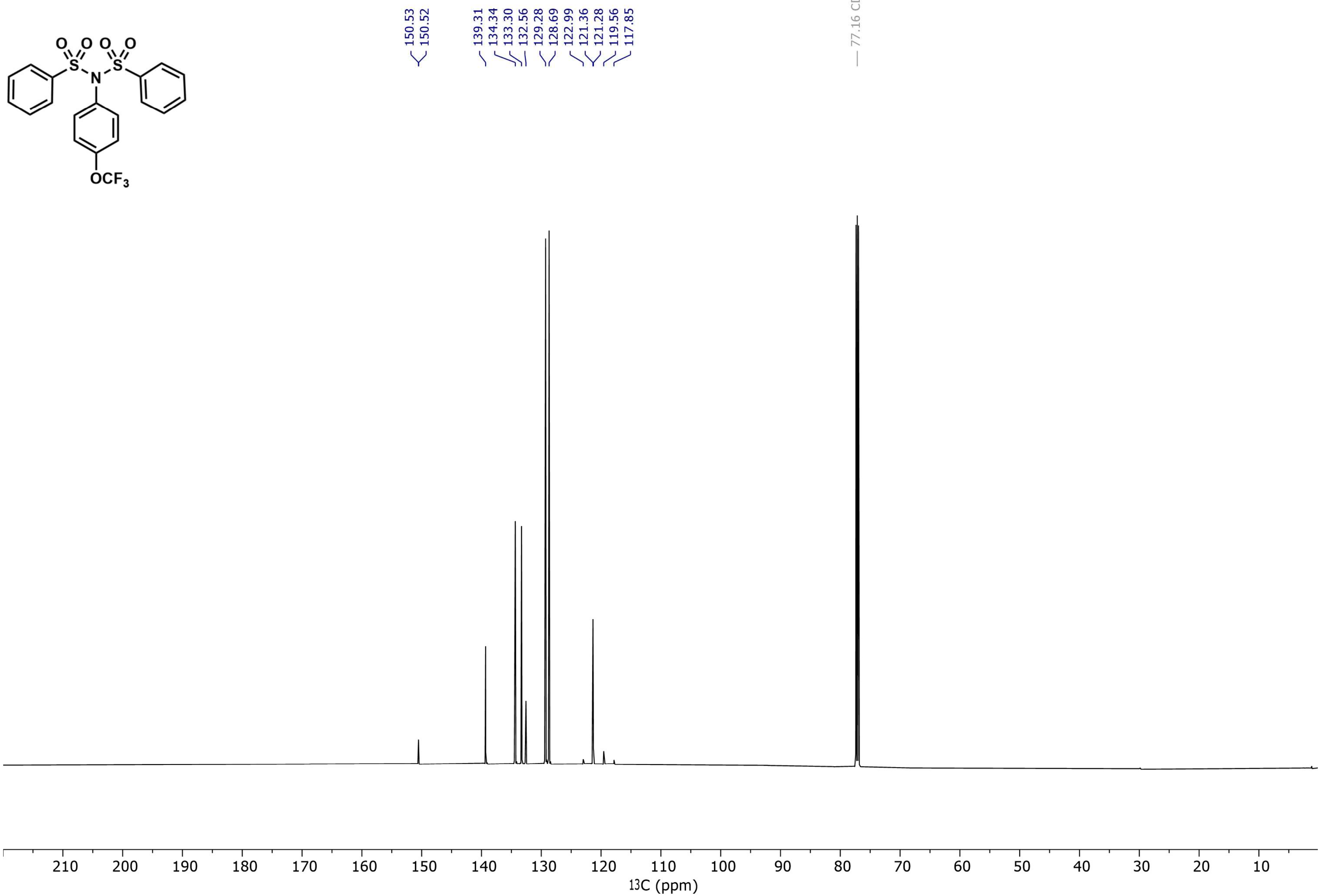

19F NMR (282 MHz, CDCl3)

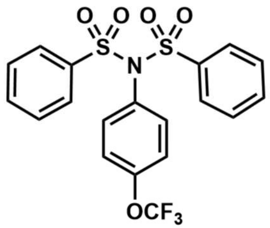

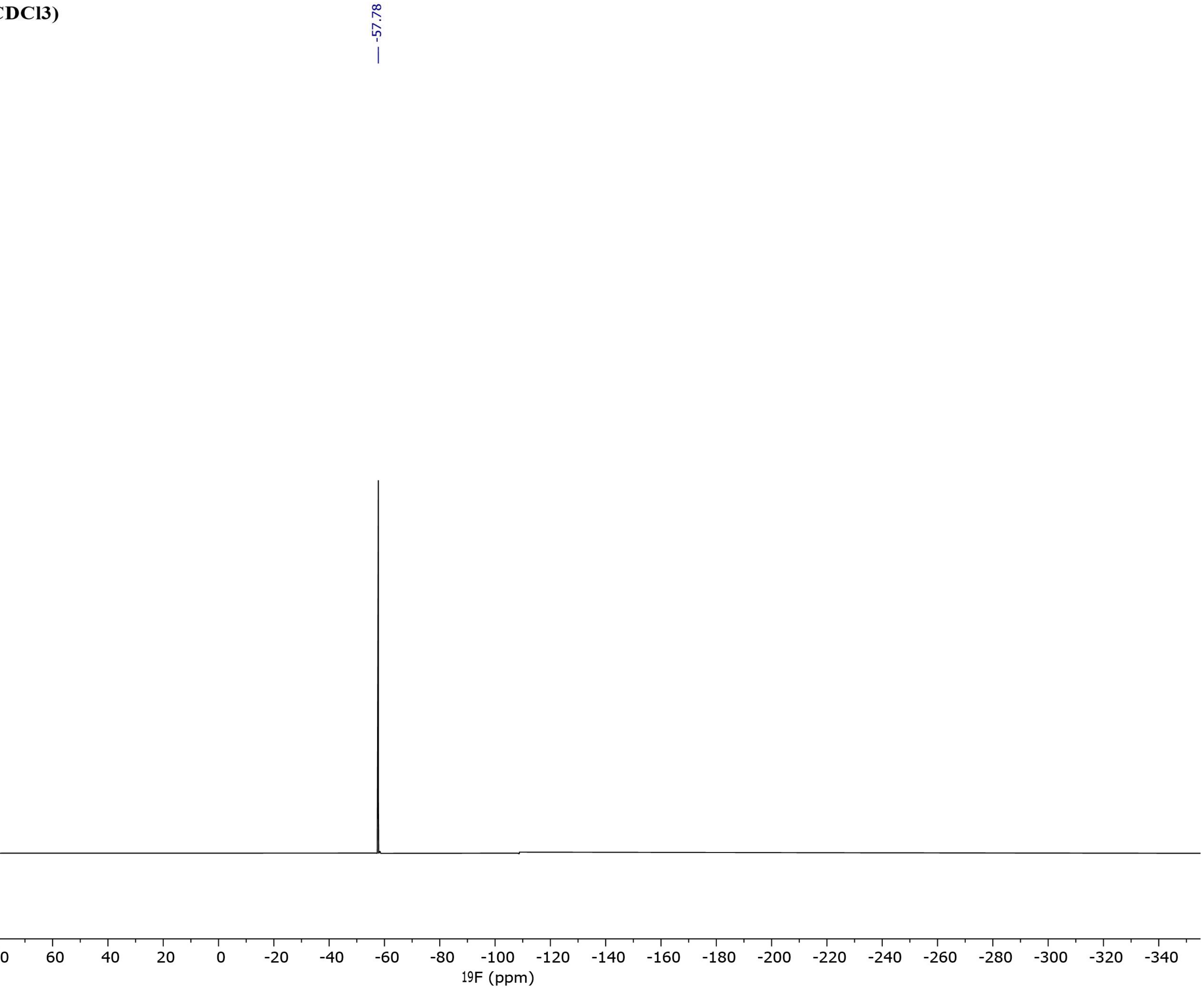

4-(trifluoromethoxy)phenyl N-(phenylsulfonyl)benzenesulfonimidate (3g)

1H NMR (600 MHz, CDCl3)

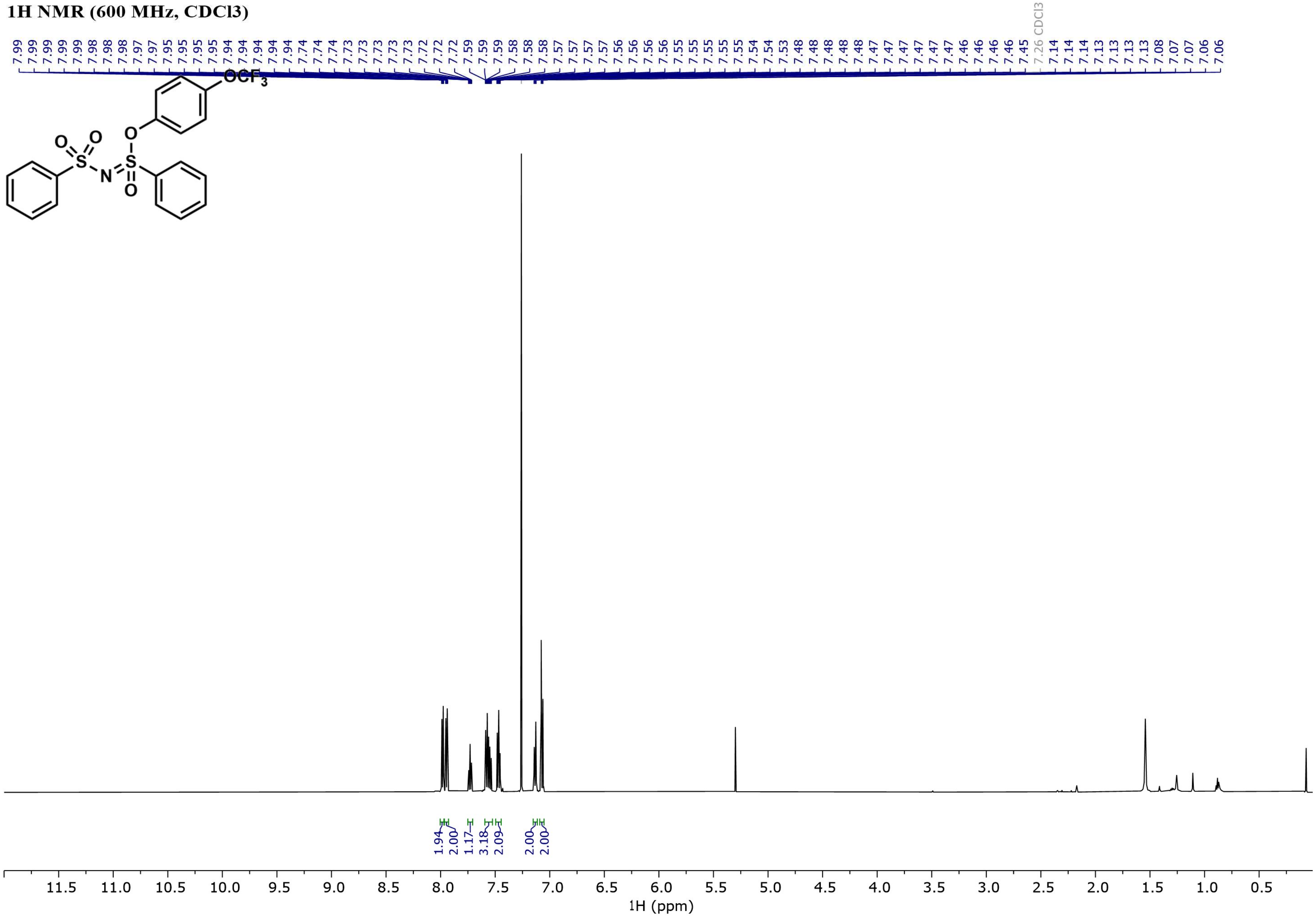

13C NMR (150 MHz, CDCl3)

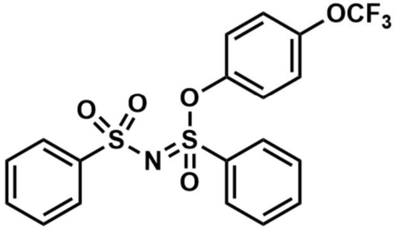

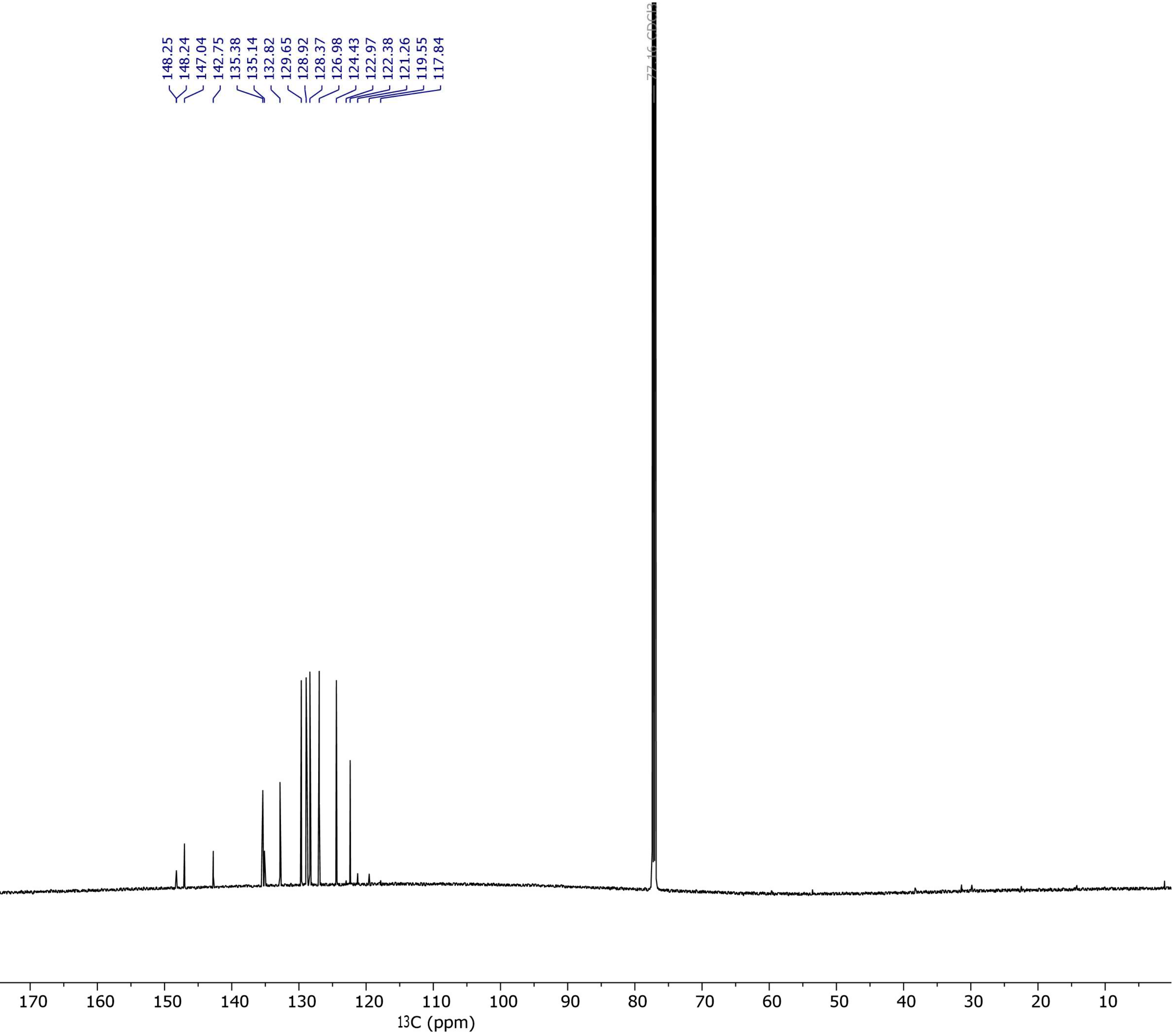

19F NMR (282 MHz, CDCl3)

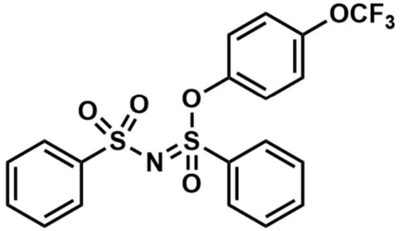

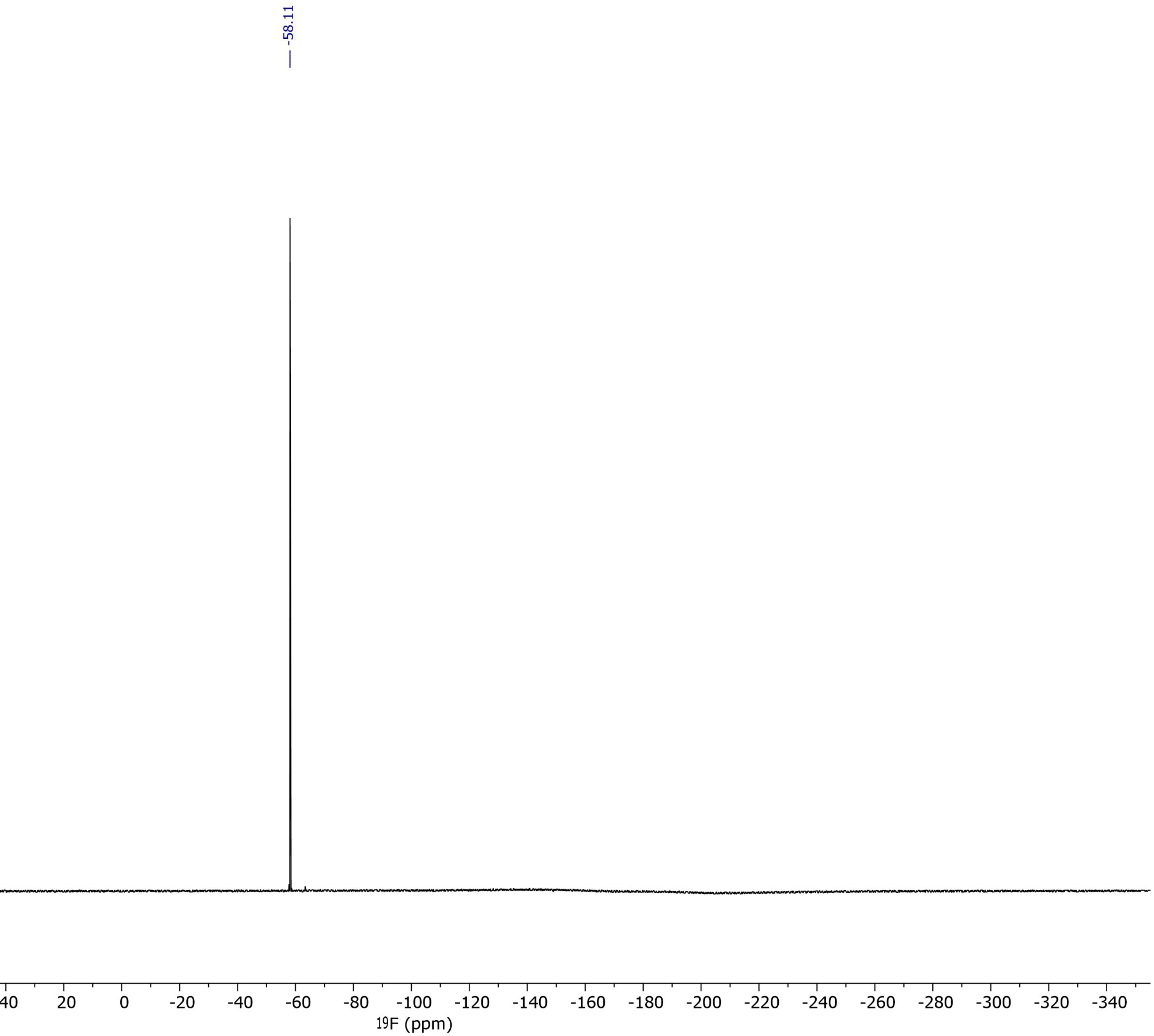

N-(phenylsulfonyl)-N-(4-(trifluoromethyl)phenyl)benzenesulfonamide (2h)

1H NMR (600 MHz, CDCl3)

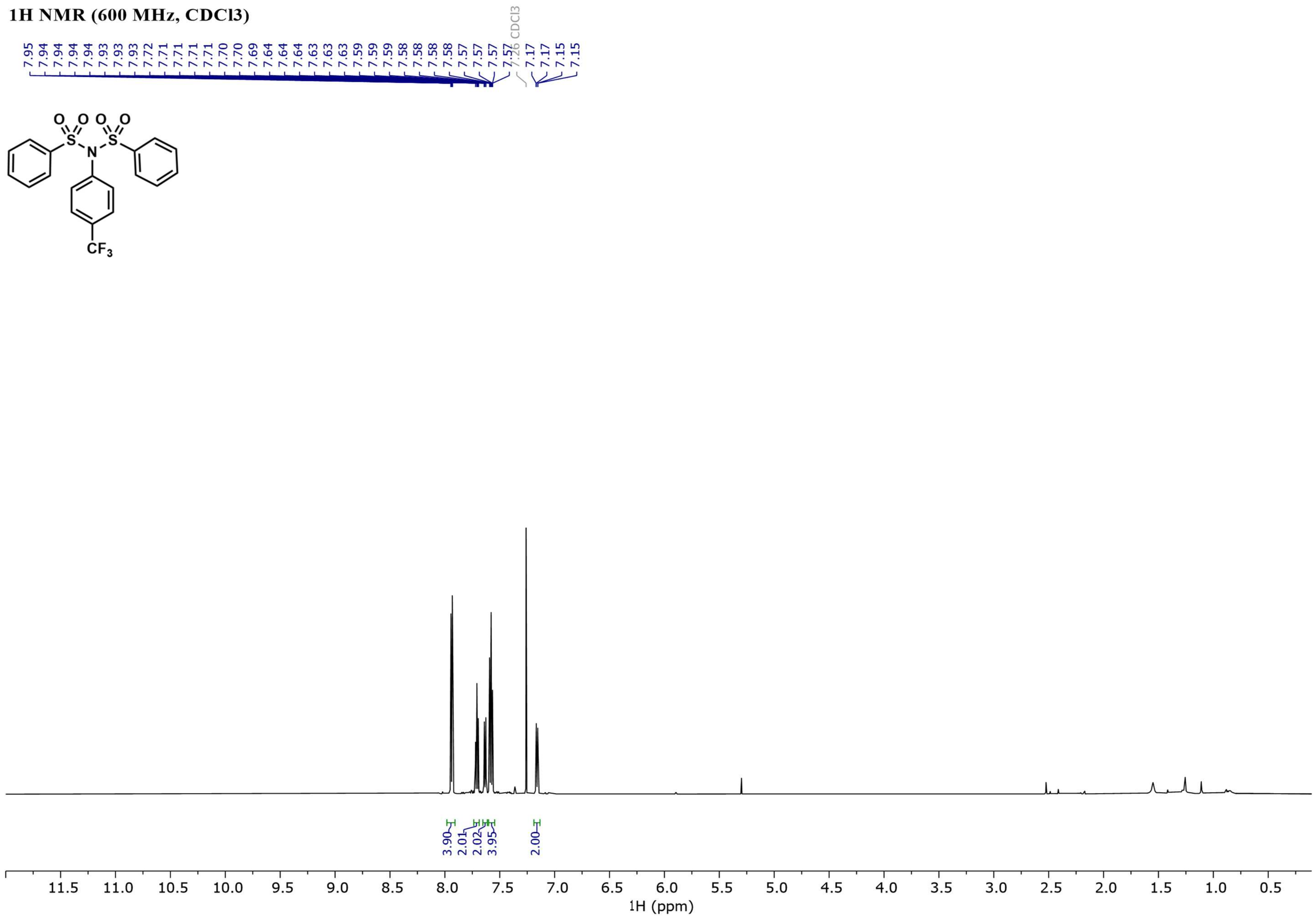

13C NMR (150 MHz, CDCl3)

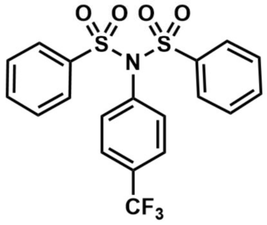

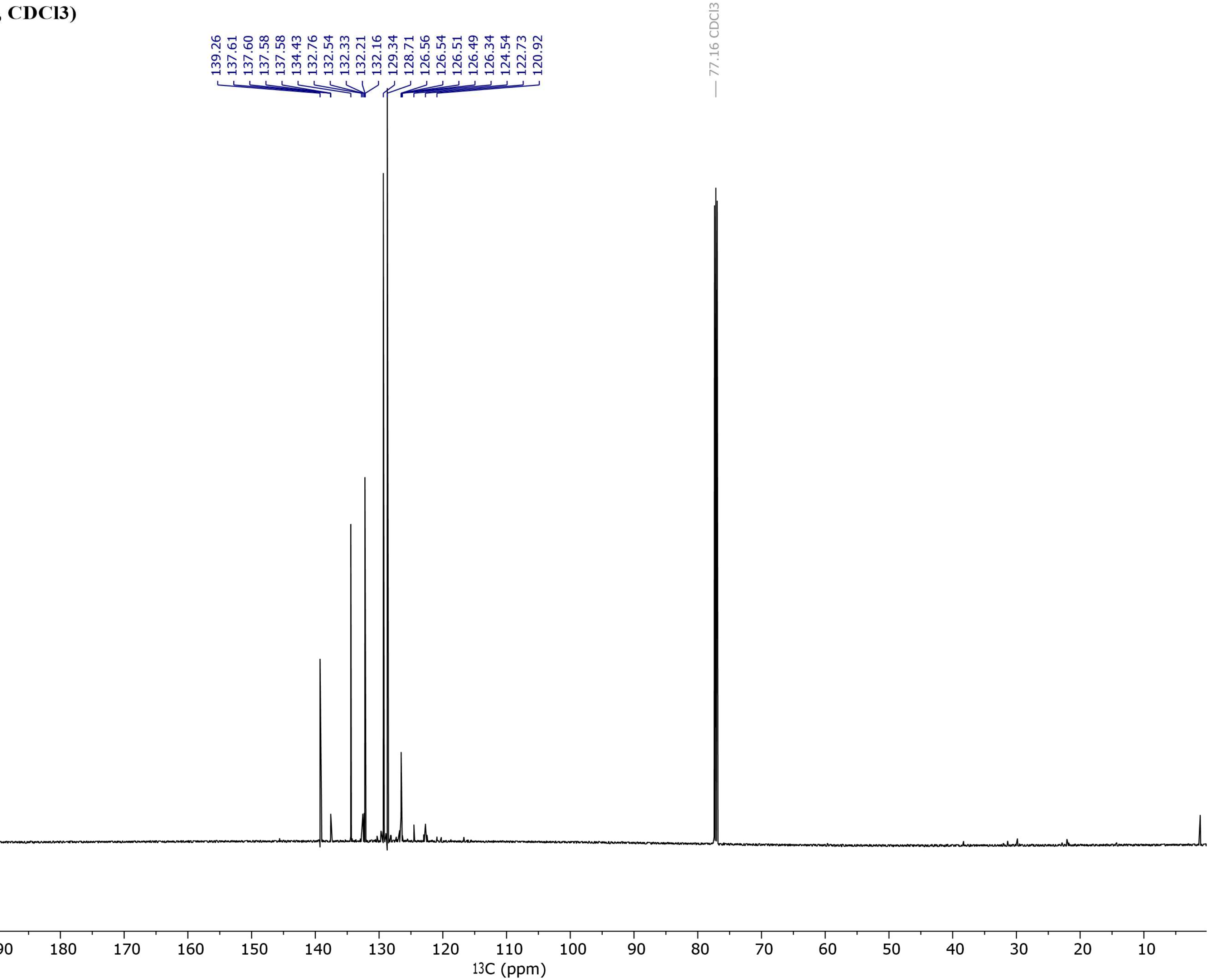

19F NMR (282 MHz, CDCl3)

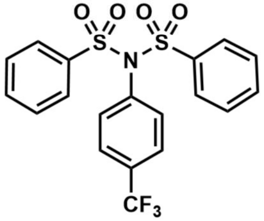

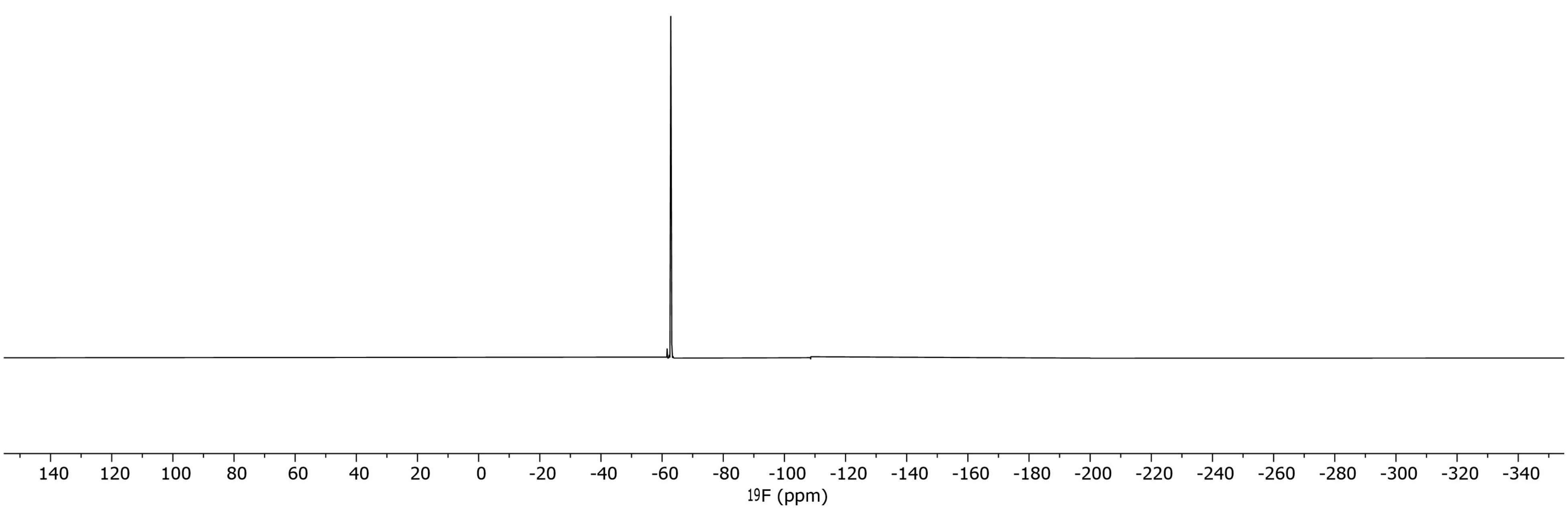

N-(4-(tert-butyl)phenyl)-4-(trifluoromethyl)-N-((4-(trifluoromethyl)phenyl)sulfonyl)benzenesulfonamide (2ab)

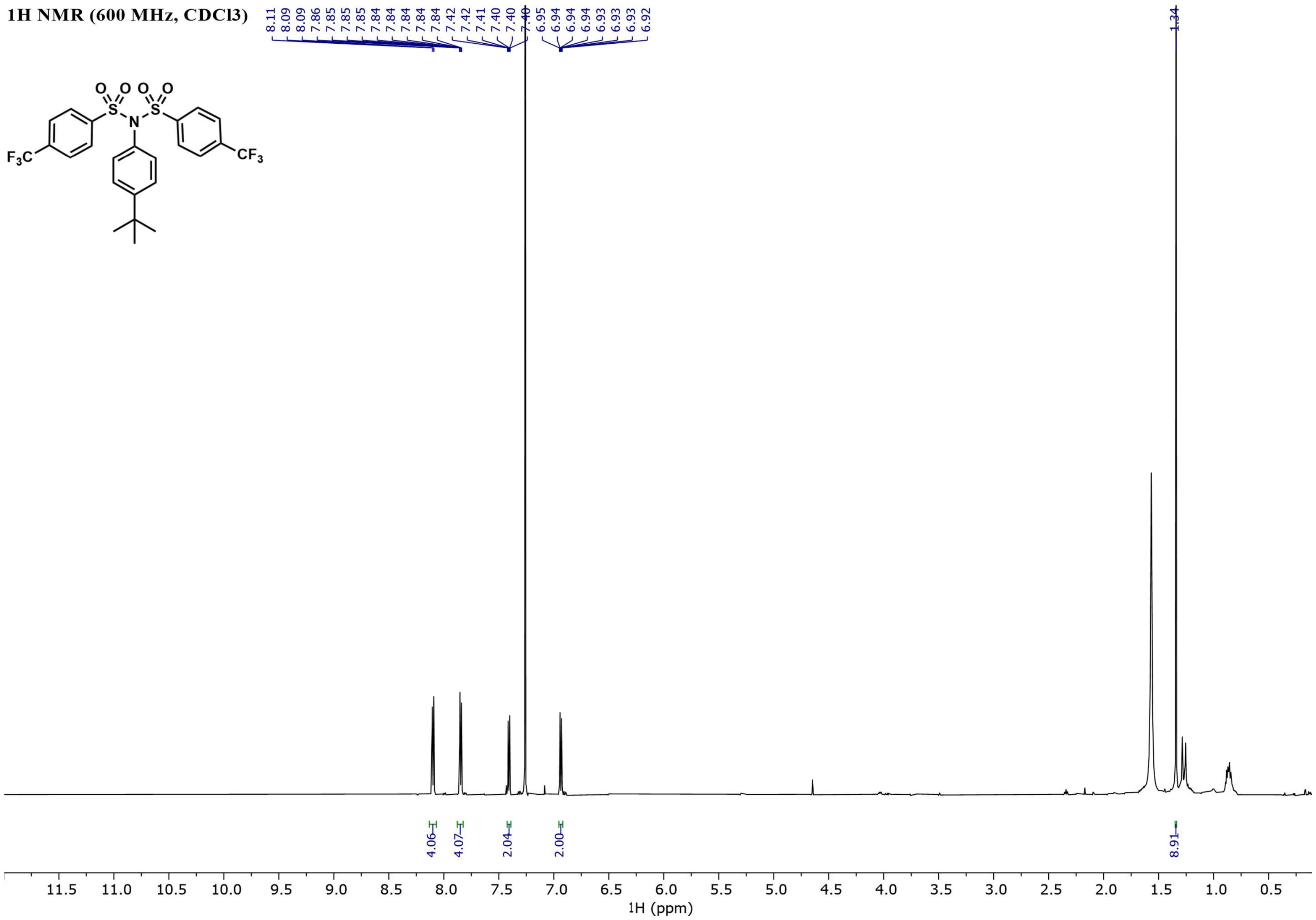

13C NMR (150 MHz, CDCl3)

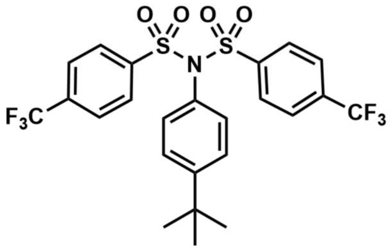

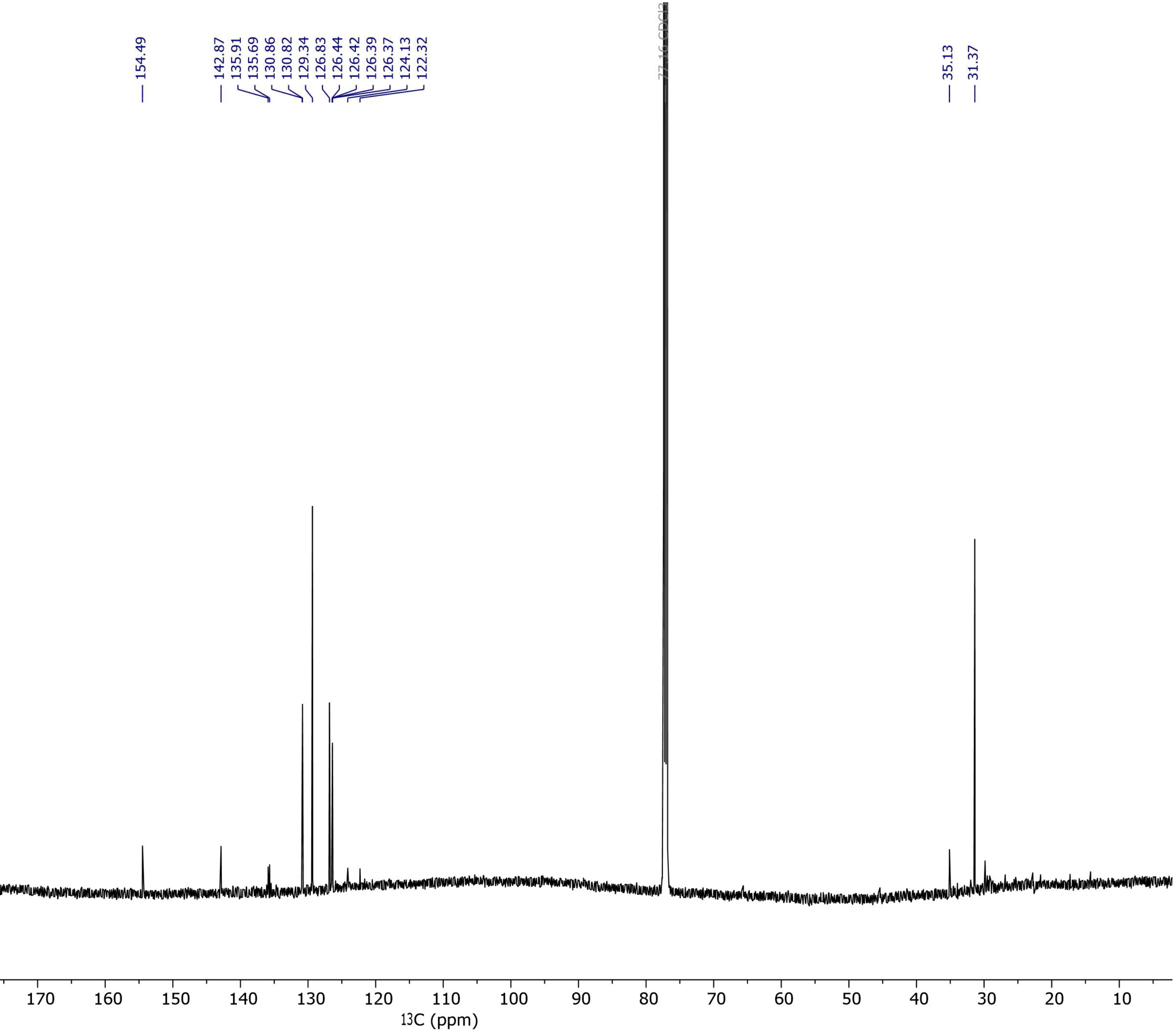

19F NMR (564 MHz, CDCl3)

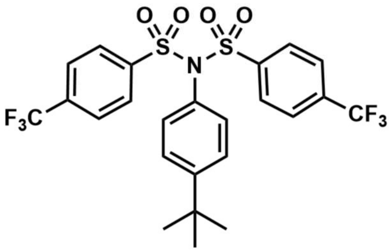

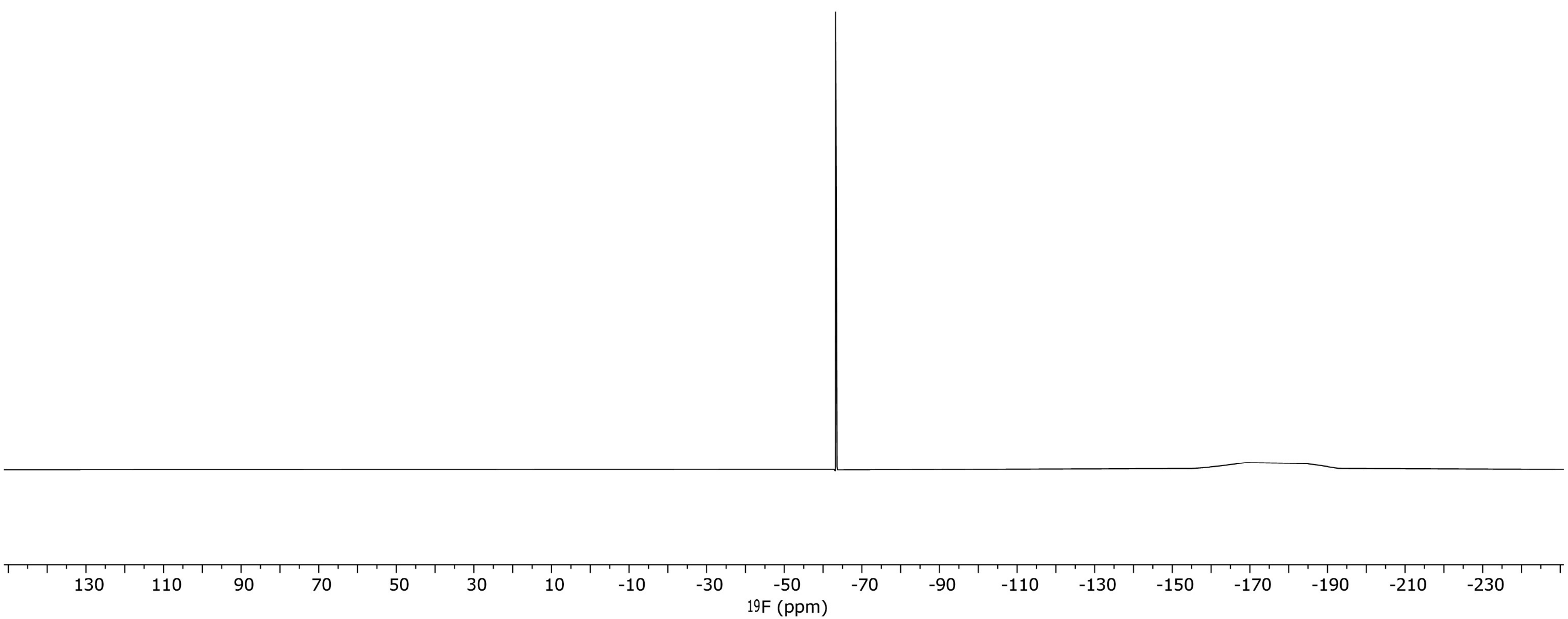

4-(tert-butyl)phenyl 4-(trifluoromethyl)-N-((4-(trifluoromethyl)phenyl)sulfonyl)benzenesulfonimidate (3ab)

1H NMR (600 MHz, CDCl3)

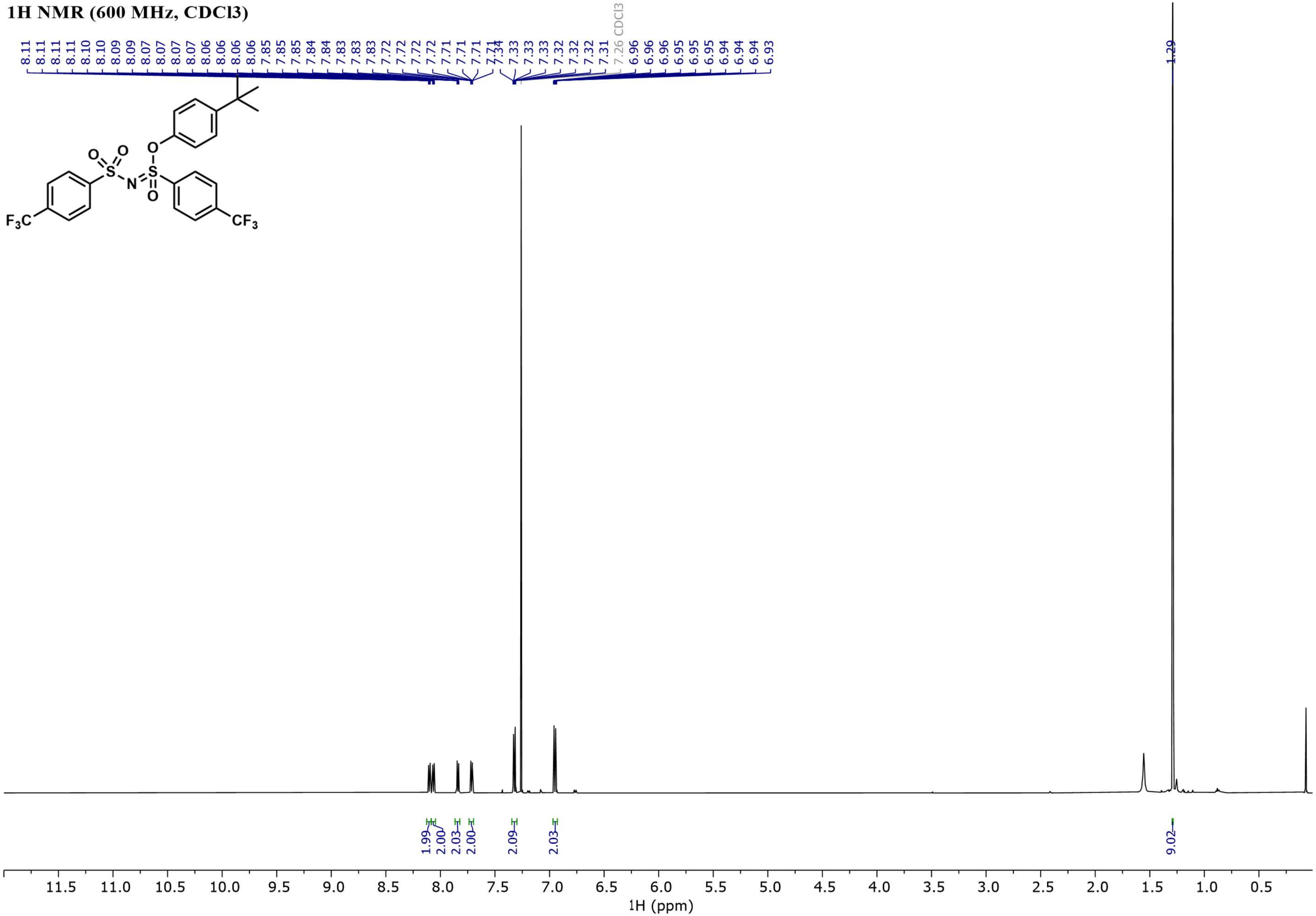

13C NMR (150 MHz, CDCl3)

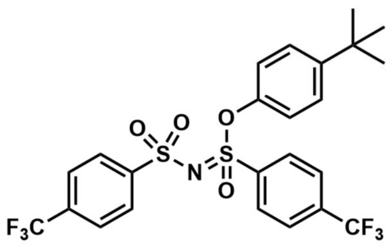

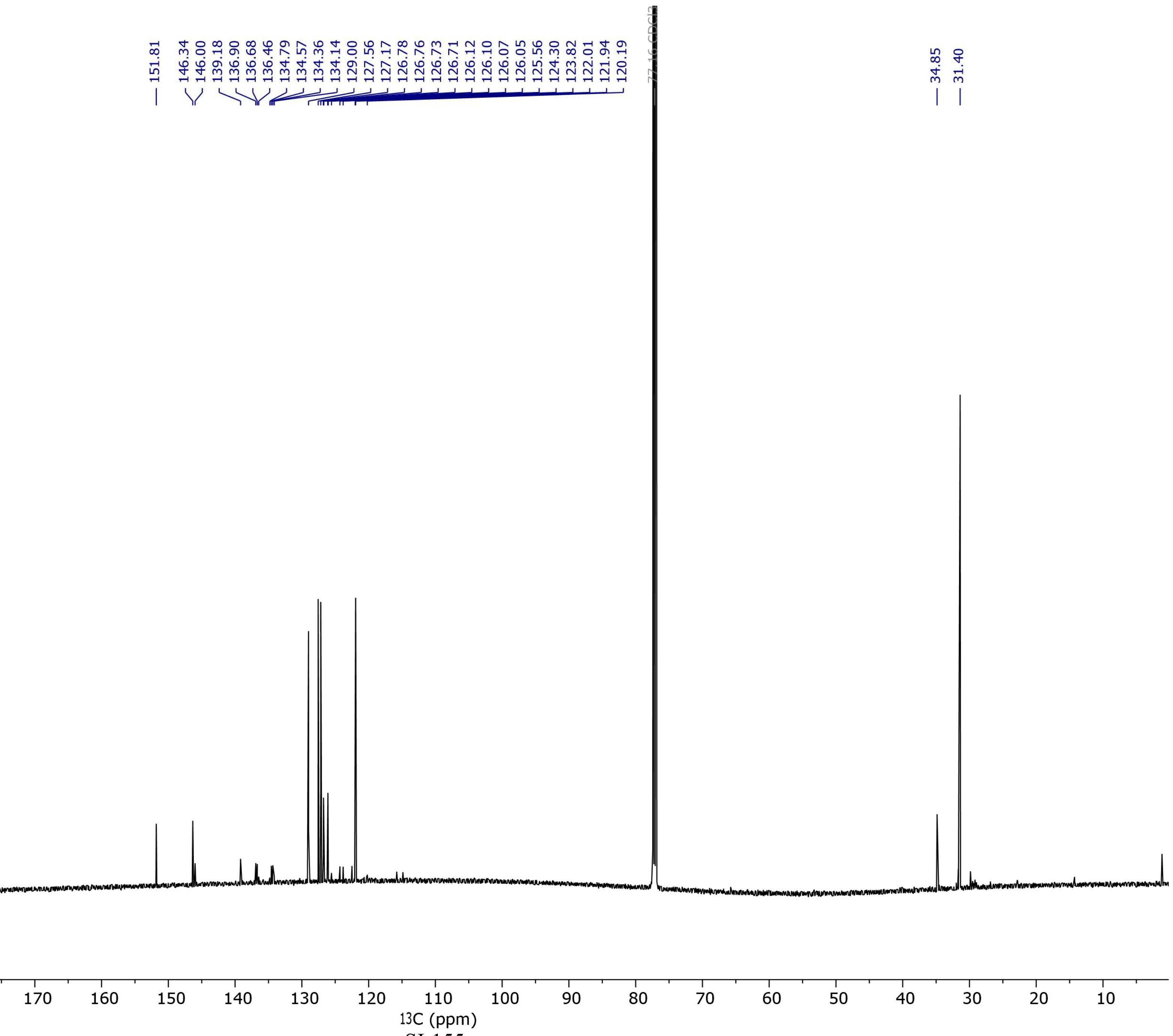

19F NMR (564 MHz, CDCl3)

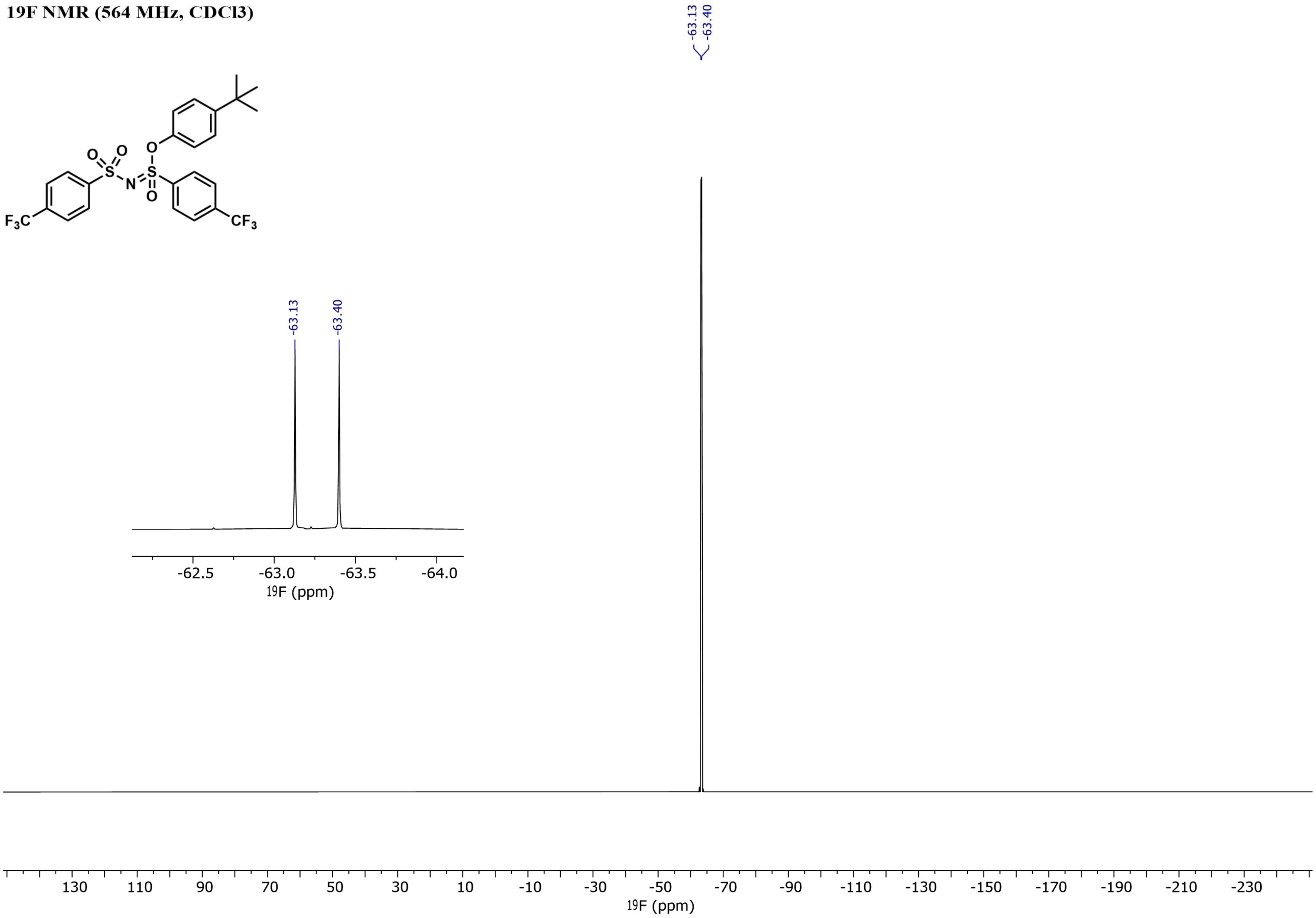

dimethyl 4,4'-(((4-(tert-butyl)phenyl)(hydrosulfonyl)amino)sulfonyl)dibenzoate (2ac)

1H NMR (600 MHz, CDCl3)

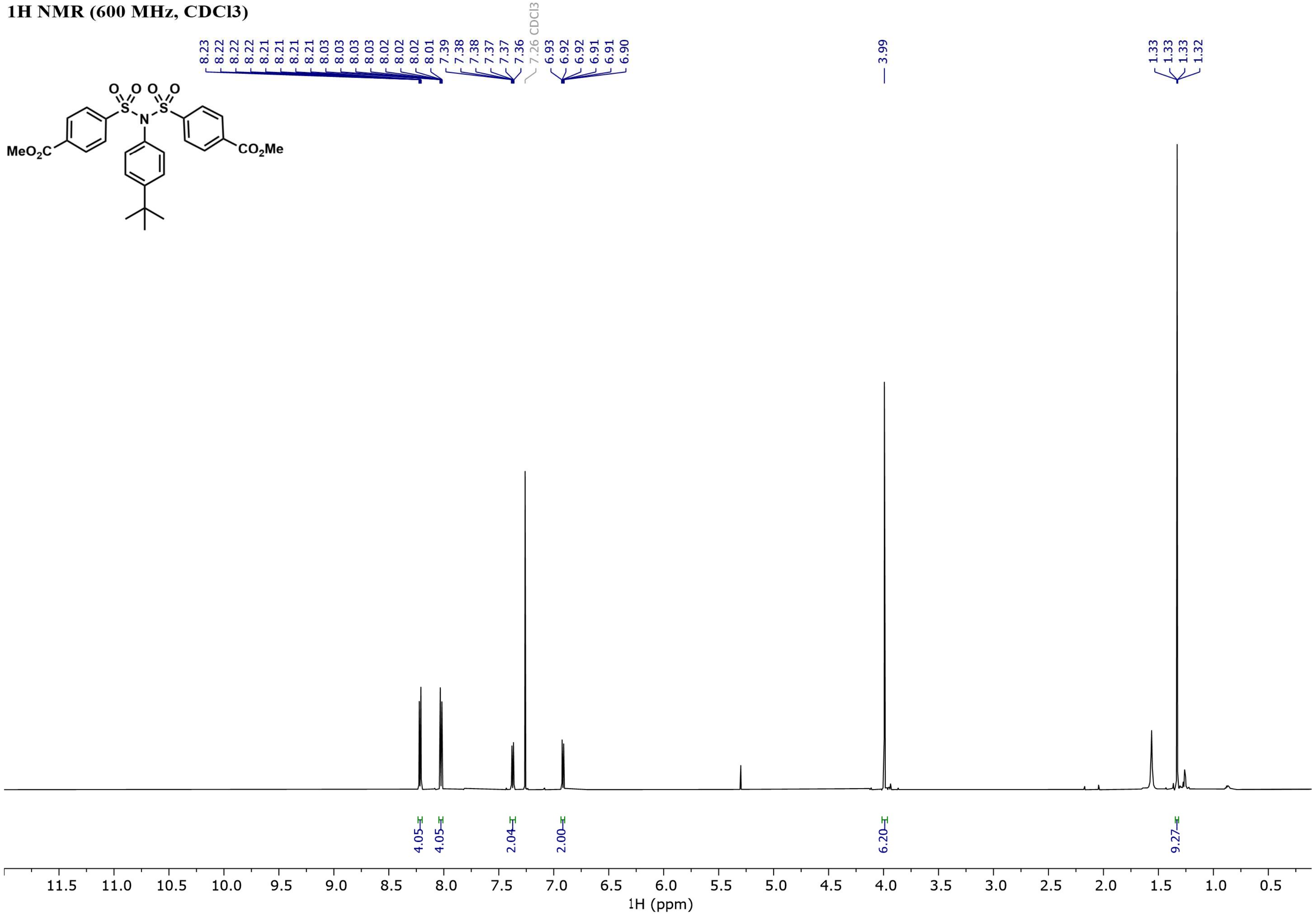

13C NMR (150 MHz, CDCl3)

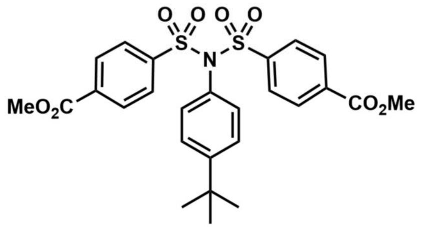

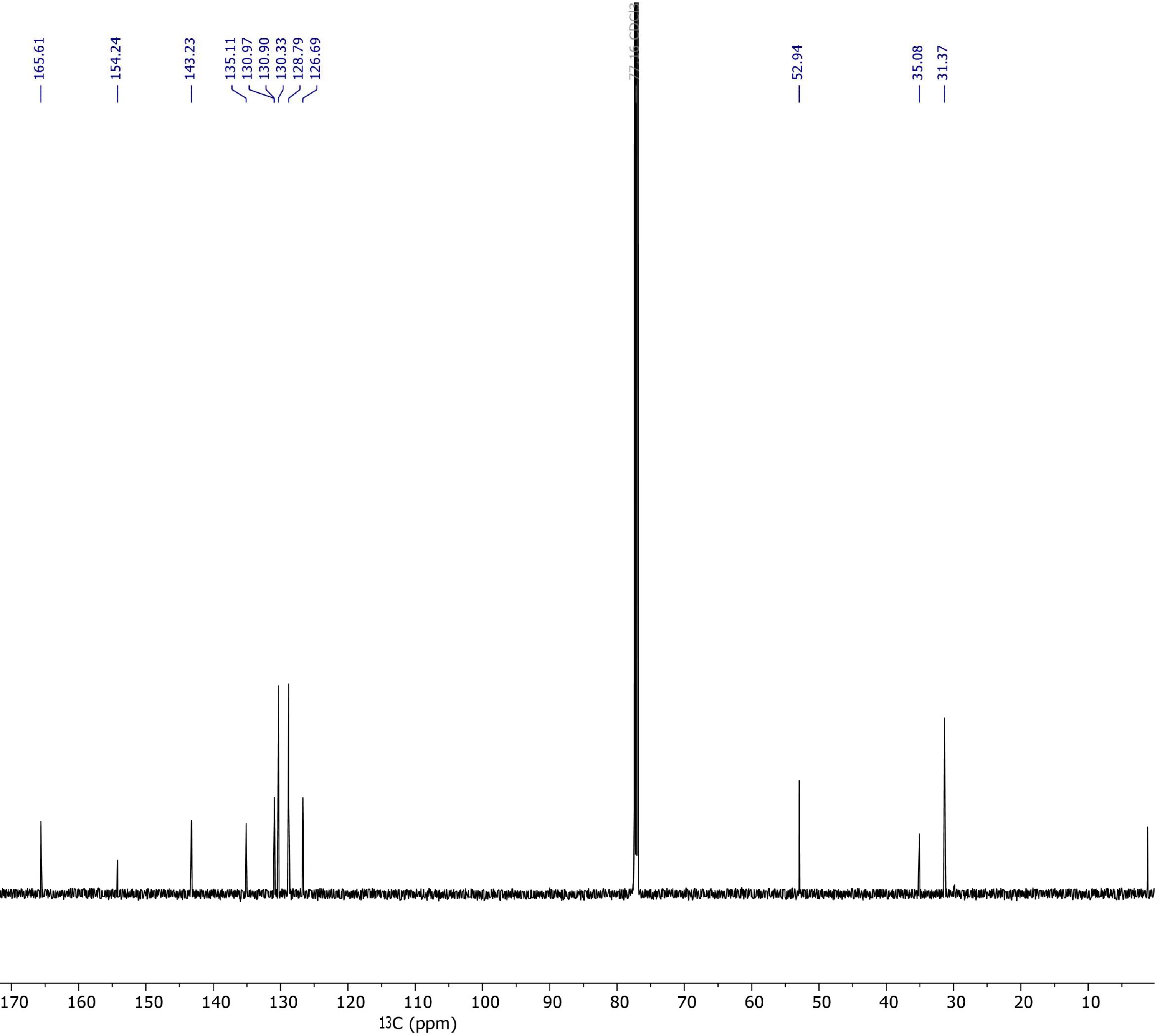

methyl 4-(N-((4-(tert-butyl)phenoxy)(4-(methoxycarbonyl)phenyl)(oxo)-l6-sulfaneylidene)sulfamoyl)benzoate (3ac)

1H NMR (600 MHz, CDCl3)

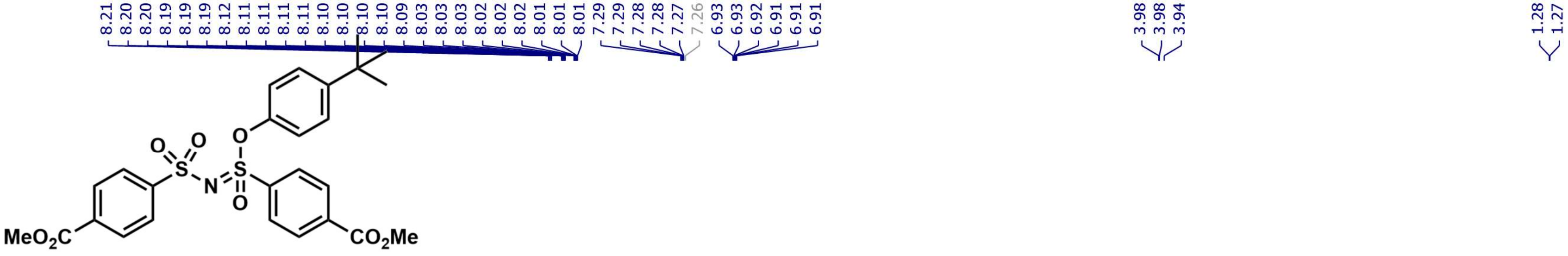

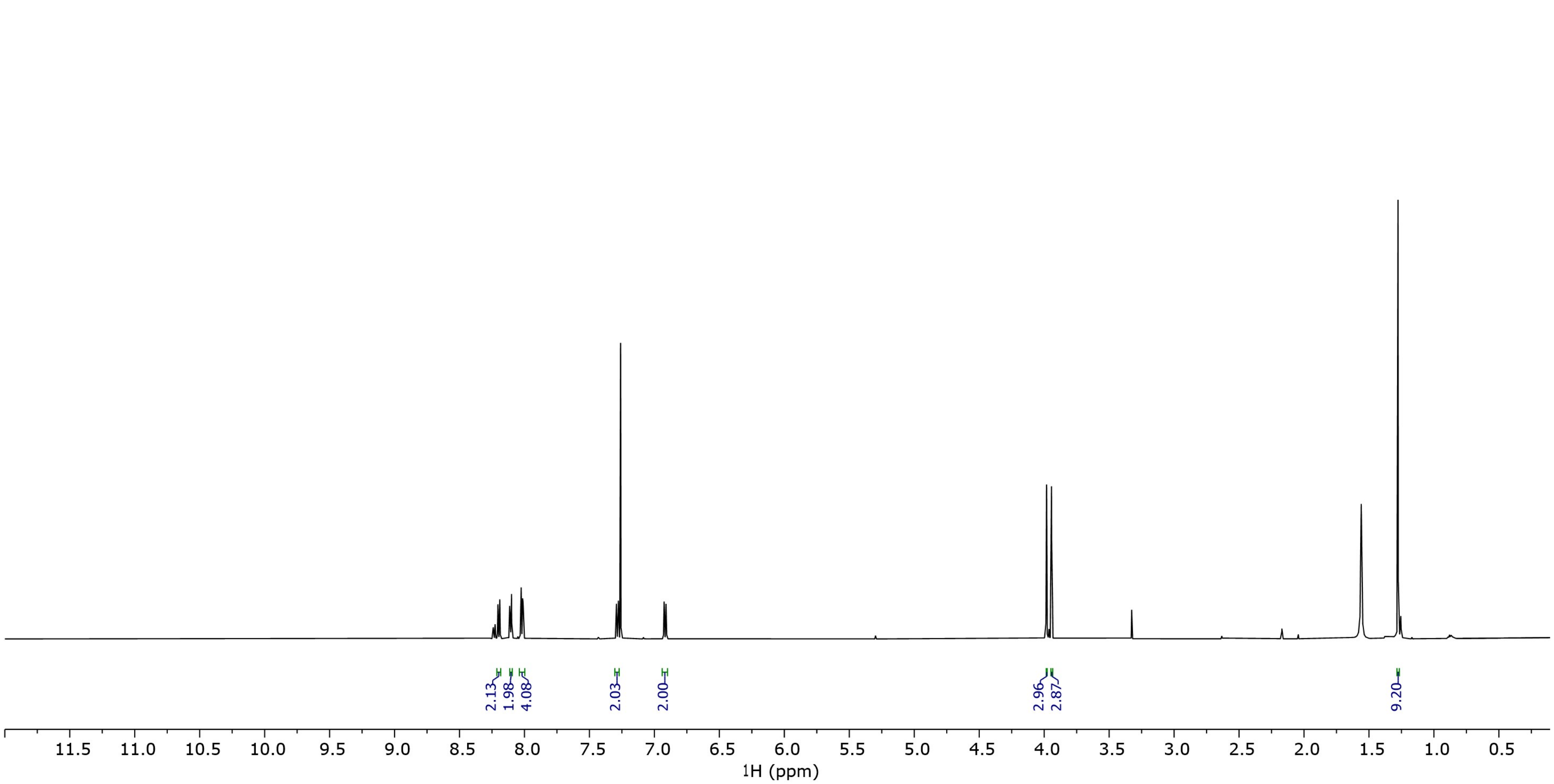

13C NMR (150 MHz, CDCl3)

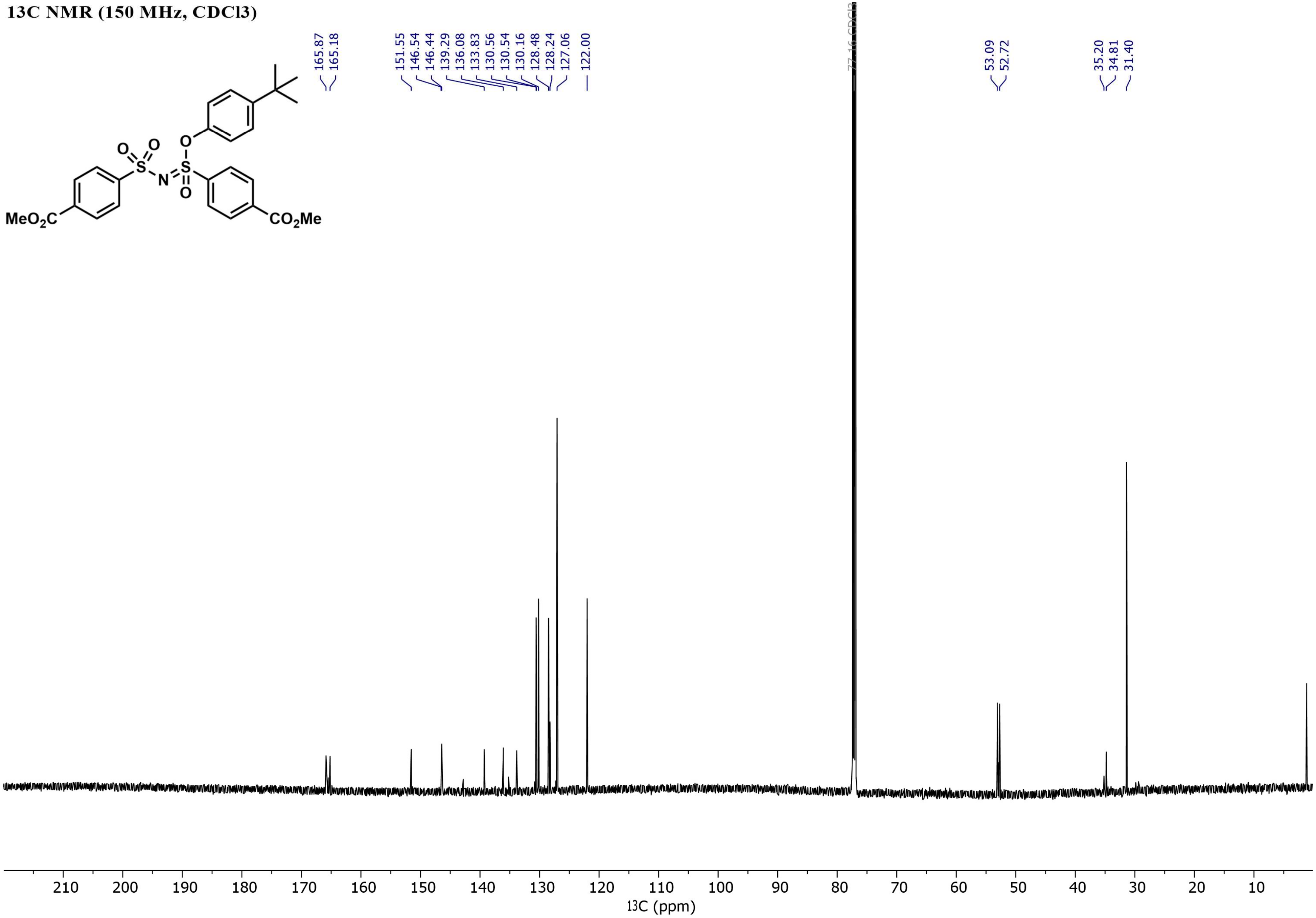

10-(4-(tert-butyl)phenyl)-2-methyl-8-(trifluoromethyl)-10H-dibenzo[b,e][1,4]thiabismine 5,5-dioxide (Bi-1·4-*t*BuPh)

1H NMR (600 MHz, CDCl3)

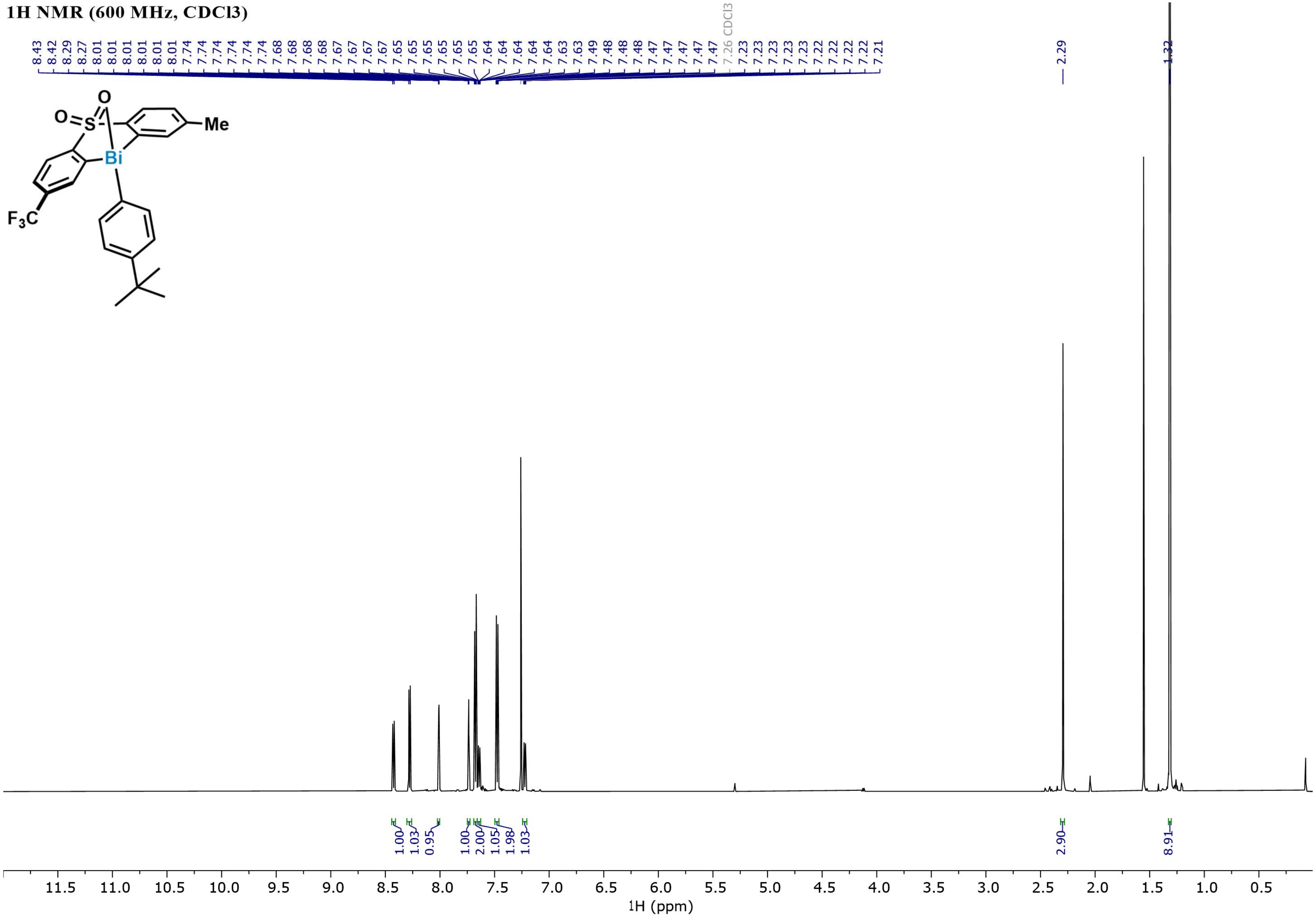

13C NMR (150 MHz, CDCl3)

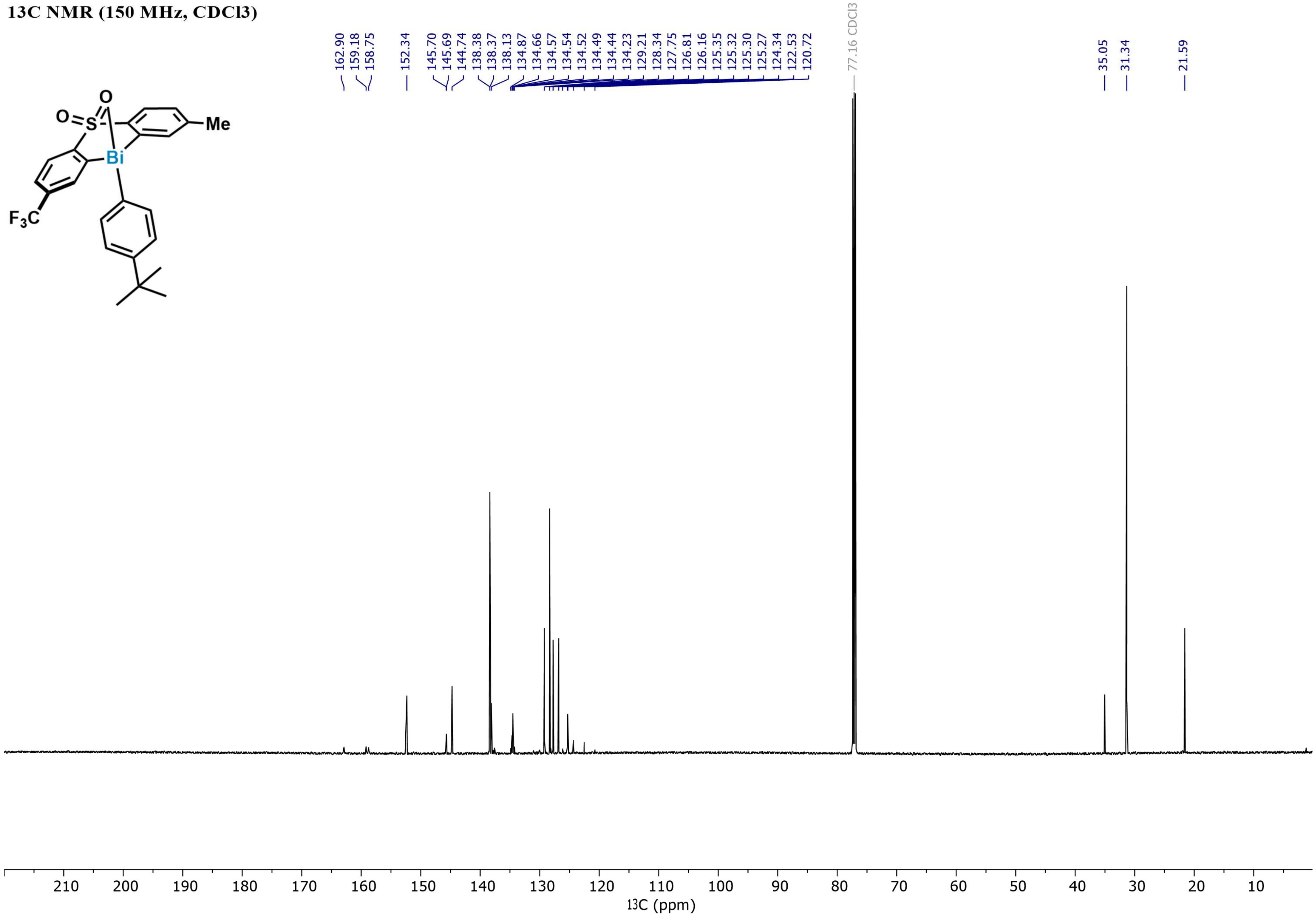

19F NMR (282 MHz, CDCl3)

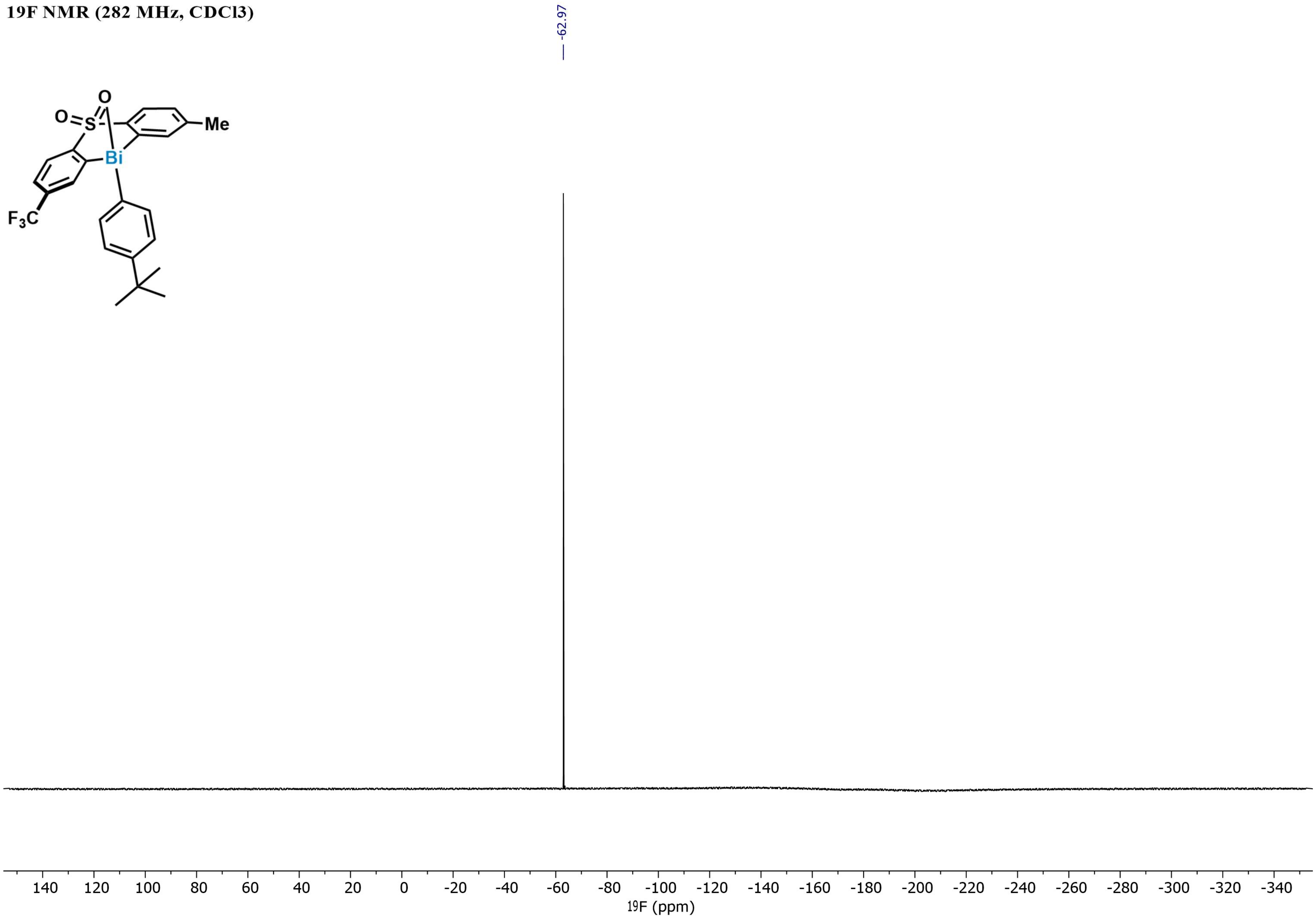

10-(4-(tert-butyl)phenyl)-10,10-difluoro-2-methyl-8-(trifluoromethyl)-10H-10l5-dibenzo[b,e][1,4]thiabismine 5,5-dioxide (6)

1H NMR (300 MHz, CDCl3)

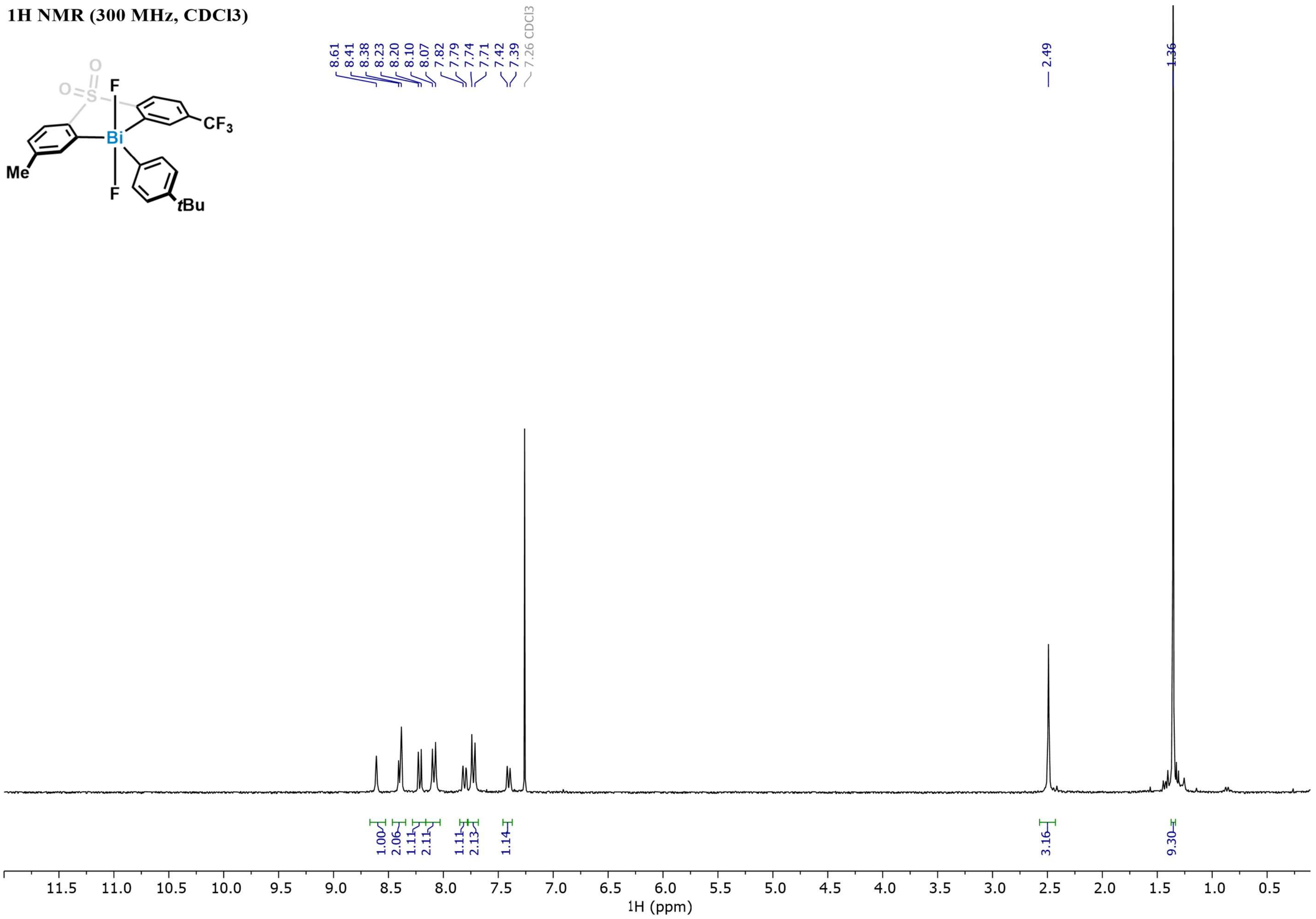

19F NMR (282 MHz, CDCl3)

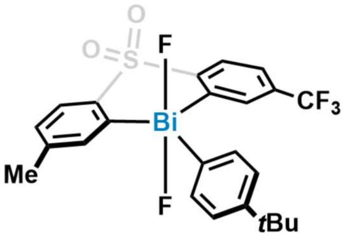

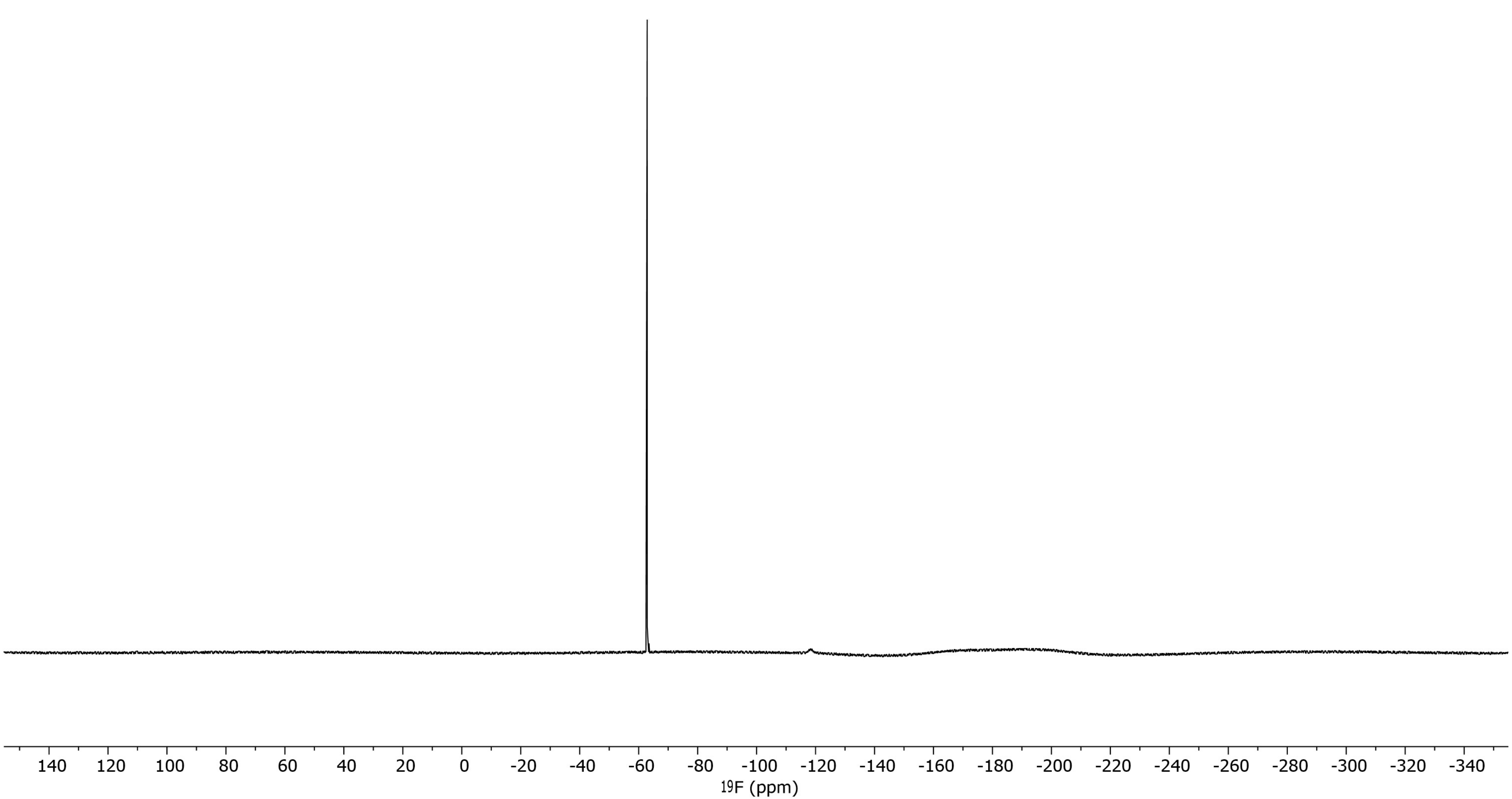

14. References

[1] Planas, O.; Wang, F.; Leutzsch, M.; Cornella, J. Fluorination of arylboronic esters enabled by bismuth redox catalysis. *Science* **2020**, *367*, 313–317.

[2] Magre, M.; Cornella, J. Redox-Neutral Organometallic Elementary Steps at Bismuth: Catalytic Synthesis of Aryl Sulfonyl Fluorides. *J. Am. Chem. Soc.* **2021**, *143*, 21497–21502.

[3] Planas, O.; Peciukenas, V.; Leutzsch, M.; Nöthling, N.; Pantazis, D. A.; Cornella, J. Mechanism of the Aryl–F Bond-Forming Step from Bi(V) Fluorides. *J. Am. Chem. Soc.* **2022**, *144*, 14489–14504.

[4] Faber, T.; Engelhardt, S.; Cornella, J. Aryl Silicon Nucleophiles in Bismuth Catalysis. *Angew. Chem., Int. Ed.* **2025**, *64*, e202424698.

[5] Benjamin, S. L.; Karagiannidis, L.; Levason, W.; Reid, G.; Rogers, M. C. Hybrid Dibismuthines and Distibines: Preparation and Properties of Antimony and Bismuth Oxygen, Sulfur, and Nitrogen Donor Ligands. *Organometallics* **2011**, *30*, 895–904.

[6] Hejda, M.; Jirásko, R.; Růžička, A.; Jambor, R.; Dostál, L. Probing the Limits of Oxidative Addition of C(sp^3)–X Bonds toward Selected *N,C,N*-Chelated Bismuth(I) Compounds. *Organometallics* **2020**, *39*, 4320–4328.

[7] Hernández, J. G.; Ardila-Fierro, K. J.; Barišić, D.; Geneste, H. Multi-Faceted Reactivity of N-Fluorobenzenesulfonimide (NFSI) under Mechanochemical Conditions: Fluorination, Fluorodemethylation, Sulfonylation, and Amidation Reactions. *Beilstein J. Org. Chem.* **2022**, *18*, 182–189.

[8] Shaw, M. M.; Smith, R. G.; Ramsden, C. A. The reaction of xenon difluoride with chloroform. *J. Fluorine Chem.* **2002**, *116*, 71–73

[9] Chiappini, N. D.; Geunes, E. P.; Bodak, E. T.; Knowles, R. R. Organobismuth Compounds as Aryl Radical Precursors via Light-Driven Single-Electron Transfer. *ACS Catal.* **2024**, *14*, 2664–2670.

[10] Shao, Z.; Wang, F.; Shi, J.; Ma, L.; Li, Z. Synergetic copper/TEMPO-catalysed benzylic C–H imidation with *N*-fluorobenzenesulfonimide at room temperature and tandem conversions with alcohols or arenes. *Org. Chem. Front.* **2021**, 8, 3298–3307.

[11] *N*-centered radical *ipso*-substitution of arylboronic acids: Chan, A. Y.; Ghosh, A.; Yarranton, J. T.; Twilton, J.; Jin, J.; Arias-Rotondo, D. M.; Sakai, H. A.; McCusker, J. K.; MacMillan, D. W. C. Exploring the Marcus inverted region for first-row transition metal-based photoredox catalysis. *Science* **2023**, *382*, 191–197.

[12] Buss, J. A.; Vasilopoulos, A.; Golden, D. L.; Stahl, S. S. Copper-Catalyzed Functionalization of Benzylic C–H Bonds with N-Fluorobenzenesulfonimide: Switch from C–N to C–F Bond Formation Promoted by a Redox Buffer and Brønsted Base *Org. Lett.* **2020**, *22*, 5749–5752.

[13] TURBOMOLE V7.5.2 2021, a development of University of Karlsruhe and Forschungszentrum Karlsruhe GmbH, TURBOMOLE GmbH, available from http://www.turbomole.com.

[14] a) Becke, A. D. Density-functional exchange-energy approximation with correct asymptotic behavior. *Phys. Rev. A* **1988**, *38*, 3098-3100; b) Perdew, J. P. Density-functional approximation for the correlation energy of the inhomogeneous electron gas. *Phys. Rev. B* **1986**, *33*, 8822–8824.

[15] Weigend, F.; Ahlrichs, R. Balanced basis sets of split valence, triple zeta valence and quadruple zeta valence quality for H to Rn: Design and assessment of accuracy. *Phys. Chem. Chem. Phys.* **2005**, *7*, 3297–3305.

[16] Caldeweyher, E.; Ehlert, S.; Hansen, A.; Neugebauer, H.; Spicher, S.; Bannwarth, C.; Grimme, S. A generally applicable atomic-charge dependent London dispersion correction. *J. Chem. Phys.* **2019**, *150*, 154122.

[17] Metz, B.; Stoll, H.; Dolg, M. Small-core multiconfiguration-Dirac–Hartree–Fock-adjusted pseudopotentials for post-d main group elements: Application to PbH and PbO. *J. Chem. Phys.* **2000**, *113*, 2563–2569.

[18] Klamt, A.; Schüürmann, G. COSMO : a new approach to dielectric screening in solvents with explicit expressions for the screening energy and its gradient. *J. Chem. Soc., Perkin Trans. 2* **1993**, 799–805.

[19] a) Becke, A. D. Density-functional thermochemistry. III. The role of exact exchange. *J. Chem. Phys.* **1993**, *98*, 5648–5652; b) Lee, C.; Yang, W.; Parr, R. G. Development of the Colle-Salvetti correlation-energy formula into a functional of the electron density. *Phys. Rev. B* **1988**, *37*, 785–789; c) Stephens, P. J.; Devlin, F. J.; Chabalowski, C. F.; Frisch, M. J. Ab Initio Calculation of Vibrational Absorption and Circular Dichroism Spectra Using Density Functional Force Fields. *J. Phys. Chem.* **1994**, *98*, 11623–11627; d) Vosko, S. H.; Wilk, L.; Nusair, M. Accurate spin-dependent electron liquid correlation energies for local spin density calculations: a critical analysis. *Can. J. Phys.* **1980**, *58*, 1200–1211.

[20] a) Deglmann, P.; May, K.; Furche, F.; Ahlrichs, R. Nuclear second analytical derivative calculations using auxiliary basis set expansions. *Chem. Phys. Lett.* **2004**, *384*, 103–107; b) Eichkorn, K.; Treutler, O.; Öhm, H.; Häser, M.; Ahlrichs, R. Auxiliary basis sets to approximate Coulomb potentials. *Chem. Phys. Lett.* **1995**, *240*, 283–290; c) Vahtras, O.; Almlöf, J.; Feyereisen, M. W. Integral approximations for LCAO-SCF calculations. *Chem. Phys. Lett.* **1993**, *213*, 514–518; d) Weigend, F. Accurate Coulomb-fitting basis sets for H to Rn. *Phys. Chem. Chem. Phys.* **2006**, *8*, 1057–1065.

[21] a) Klamt, A. Conductor-like Screening Model for Real Solvents: A New Approach to the Quantitative Calculation of Solvation Phenomena. *J. Phys. Chem.* **1995**, *99*, 2224–2235; b) Klamt, A.; Jonas, V.; Bürger, T.; Lohrenz, J. C. W. Refinement and Parametrization of COSMO-RS. *J. Phys. Chem. A* **1998**, *102*, 5074–5085.

[22] COSMOtherm Version 18.0.0 (Revision 4360), COSMOlogic GmbH & Co KG, available from http://www.cosmologic.de.

[23] Frisch, M. J.; Trucks, G. W.; Schlegel, H. B.; Scuseria, G. E.; Robb, M. A.; Cheeseman, J. R.; Scalmani, G.; Barone, V.; Petersson, G. A.; Nakatsuji, H.; Li, X.; Caricato, M.; Marenich, A. V.; Bloino, J.; Janesko, B. G.; Gomperts, R.; Mennucci, B.; Hratchian, H. P.; Ortiz, J. V.; Izmaylov, A. F.; Sonnenberg, J. L.; Williams; Ding, F.; Lipparini, F.; Egidi, F.; Goings, J.; Peng, B.; Petrone, A.; Henderson, T.; Ranasinghe, D.; Zakrzewski, V. G.; Gao, J.; Rega, N.; Zheng, G.; Liang, W.; Hada, M.; Ehara, M.; Toyota, K.; Fukuda, R.; Hasegawa, J.; Ishida, M.; Nakajima, T.; Honda, Y.; Kitao, O.; Nakai, H.; Vreven, T.; Throssell, K.; Montgomery Jr., J. A.; Peralta, J. E.; Ogliaro, F.; Bearpark, M. J.; Heyd, J. J.; Brothers, E. N.; Kudin, K. N.; Staroverov, V. N.; Keith, T. A.; Kobayashi, R.; Normand, J.; Raghavachari, K.; Rendell, A. P.; Burant, J. C.; Iyengar, S. S.; Tomasi, J.; Cossi, M.; Millam, J. M.; Klene, M.; Adamo, C.; Cammi, R.; Ochterski, J. W.; Martin, R. L.; Morokuma, K.; Farkas, O.; Foresman, J. B.; Fox, D. J. Gaussian 16 Rev. C.01, Wallingford, CT, **2016**.

[24] CYLview20; Legault, C. Y., Université de Sherbrooke, **2020**. http://www.cylview.org.

[25] Zhurko, G. A.; Zhurko, D. A. ChemCraft: Tool for treatment of chemical data **2005**. https://www.chemcraftprog.com.

[26] a) Zimmerman, P. Reliable Transition State Searches Integrated with the Growing String Method. *J. Chem. Theory Comput.* **2013**, *9*, 3043–3050; b) Zimmerman, P. M. Growing string method with interpolation and optimization in internal coordinates: Method and examples. *J. Chem. Phys.* **2013**, *138*, 184102; c) Zimmerman, P. M. Single-ended transition state finding with the growing string method. *J. Comput. Chem.* **2015**, *36*, 601–611.

[27] Geiger, J.; Settels, V.; Deglmann, P.; Schäfer, A.; Bergeler, M. Automated input structure generation for single-ended reaction path optimizations. *J. Comput. Chem.* **2022**, *43*, 1662–1674.

[28] a) Grimme, S. Exploration of Chemical Compound, Conformer, and Reaction Space with Meta-Dynamics Simulations Based on Tight-Binding Quantum Chemical Calculations. *J. Chem. Theory Comput.* **2019**, *15*, 2847–2862; b) Pracht, P.; Bohle, F.; Grimme, S. Automated exploration of the low-energy chemical space with fast quantum chemical methods. *Phys. Chem. Chem. Phys.* **2020**, *22*, 7169–7192.

[29] a) Neese, F. The ORCA program system. *WIREs Comput. Mol. Sci.* **2012**, *2*, 73–78; b) Neese, F. Software update: The ORCA program system—Version 5.0. *WIREs Comput. Mol. Sci.* **2022**, *12*, e1606.

[30] Barone, V.; Cossi, M. Quantum Calculation of Molecular Energies and Energy Gradients in Solution by a Conductor Solvent Model. *J. Phys. Chem. A* **1998**, *102*, 1995–2001.

[31] Jorner, K.; Turcani, L. Morfeus (version 0.7.2), https://github.com/digital-chemistry-laboratory/morfeus.

[32] Reed, A. E.; Weinstock, R. B.; Weinhold, F. Natural population analysis. *J. Chem. Phys.* **1985**, *83*, 735–746.

[33] Hansch, C.; Leo, A.; Taft, R. W. A survey of Hammett substituent constants and resonance and field parameters. *Chem. Rev.* **1991**, *91*, 165–195.

[34] Haas, B. C., Hardy, M. A. SigmanGroup/Get_Properties: Get_Properties_v1.0.3 (v1.0.3), **2024**. https://doi.org/10.5281/zenodo.10651727.

[35] RDKit: Open-source cheminformatics, https://www.rdkit.org.

[36] LeSueur, A.; Peters, E.; Crawford, J.; Howard, J. python-modeling, **2025**. https://github.com/SigmanGroup/python-modeling.

[37] Pedregosa, F.; Varoquaux, G.; Gramfort, A.; Michel, V.; Thirion, B.; Grisel, O.; Blondel, M.; Prettenhofer, P.; Weiss, R.; Dubourg, V.; Vanderplas, J.; Passos, A.; Cournapeau, D.; Brucher, M.; Perrot, M.; Duchesnay, É. Scikit-learn: Machine Learning in Python. *J. Mach. Learn. Res.* **2011**, *12*, 2825−2830.
